# Electron-Transfer-Enabled Concerted Nucleophilic Fluorination of Azaarenes: Selective C–H Fluorination of Quinolines

**DOI:** 10.1021/jacs.3c07119

**Published:** 2023-09-11

**Authors:** Li Zhang, Jiyao Yan, Dilgam Ahmadli, Zikuan Wang, Tobias Ritter

**Affiliations:** † Max-Planck-Institut für Kohlenforschung, Kaiser-Wilhelm-Platz 1, 45470 Mülheim an der Ruhr, Germany; ‡ Institute of Organic Chemistry, RWTH Aachen University, Landoltweg 1, 52074 Aachen, Germany

## Abstract

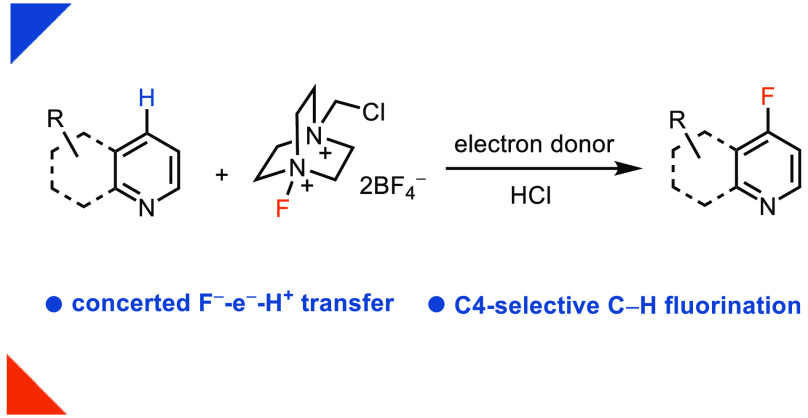

Direct C–H fluorination is an efficient strategy to construct aromatic C–F bonds, but the cleavage of specific C–H bonds in the presence of other functional groups and the high barrier of C–F bond formation make the transformation challenging. Progress for the electrophilic fluorination of arenes has been reported, but a similar transformation for electron-deficient azaarenes has remained elusive due to the high energy of the corresponding Wheland intermediates. Nucleophilic fluorination of electron-deficient azaarenes is difficult owing to the identity of the Meisenheimer intermediate after fluoride attack, from which fluoride elimination to regenerate the substrate is favored over hydride elimination to form the product. Herein, we report a new concept for C–H nucleophilic fluorination without the formation of azaarene Meisenheimer intermediates through a chain process with an asynchronous concerted F^–^-e^–^-H^+^ transfer. The concerted nucleophilic aromatic substitution strategy allows for the first successful nucleophilic oxidative fluorination of quinolines.

Single-step transformations from aromatic C–H bonds to C–F bonds are attractive methodologies because no prefunctionalization is required to obtain valuable fluorinated products, which often exhibit desirable properties and are widely applied in medicinal and materials chemistry.^1−3^ However, selective aromatic C–H bond fluorination of functionalized molecules is far from being established, in part due to a high reaction barrier associated with C–F bond formation that results from the high electronegativity of fluorine and the small ionic radius of fluoride (1.33 Å),^4^ both of which make reductive elimination from metal fluorides difficult. Therefore, aromatic C–H fluorination is a comparatively underdeveloped area with just a few successful approaches so far.^5−11^ Fluorination of six-membered azaarenes is even more challenging, and there is currently no method for a C4-selective fluorination at all.^12,13^ The sp^2^-hybridized nitrogen atom on electron-deficient azaarenes renders electrophilic aromatic substitution (S_E_Ar) difficult due to a highly unstable potential Wheland intermediate (Figure 1a).^6,13^ Radical aromatic substitution of azaarenes such as Minisci-type reactions can afford C–C, C–B and C–Si bonds^14−18^ but not C–F bonds.^19^ Nucleophilic fluorination is challenging because, after fluoride addition to form a Meisenheimer intermediate, a hydride must be removed for rearomatization. Moreover, formation of the fluoride Meisenheimer intermediate is endergonic for azaarenes (Figure 1b), which would require a facile hydride elimination so that the overall barrier is not prohibitively large. Elimination of hydride from the fluoride Meisenheimer intermediate is challenging because the high-lying HOMO upon fluoride attack has overlap with the low-lying antibonding orbital of the C–F bond (σ*_C–F_), which results in the weakening of the C–F bond and facilitates heterolytic cleavage back to the starting material (Figure 1b, bottom). Modern nucleophilic aromatic substitution (S_N_Ar) strategies to selectively attach a linchpin such as a phosphonium substituent at the C4 position of azaarenes for subsequent functionalization have found broad applications in C–H functionalization, but the strategy has not yet been extended to fluorination.^20,21^ So far, only a C2-selective fluorination was realized through a AgF_2_-mediated Chichibabin-type reaction (Figure 1c).^10^ While the C4 position is the most electrophilic site of six-membered azaarenes,^12,13^ C–H fluorination at this position has not been accomplished.^22−26^ Here we report a new approach to overcome the fundamental challenge for cleaving the strong C–H bond without the formation of high-energy Meisenheimer intermediates and successfully apply the concept to the C–H fluorination of quinolines (Figure 1d).

**Figure 1:**
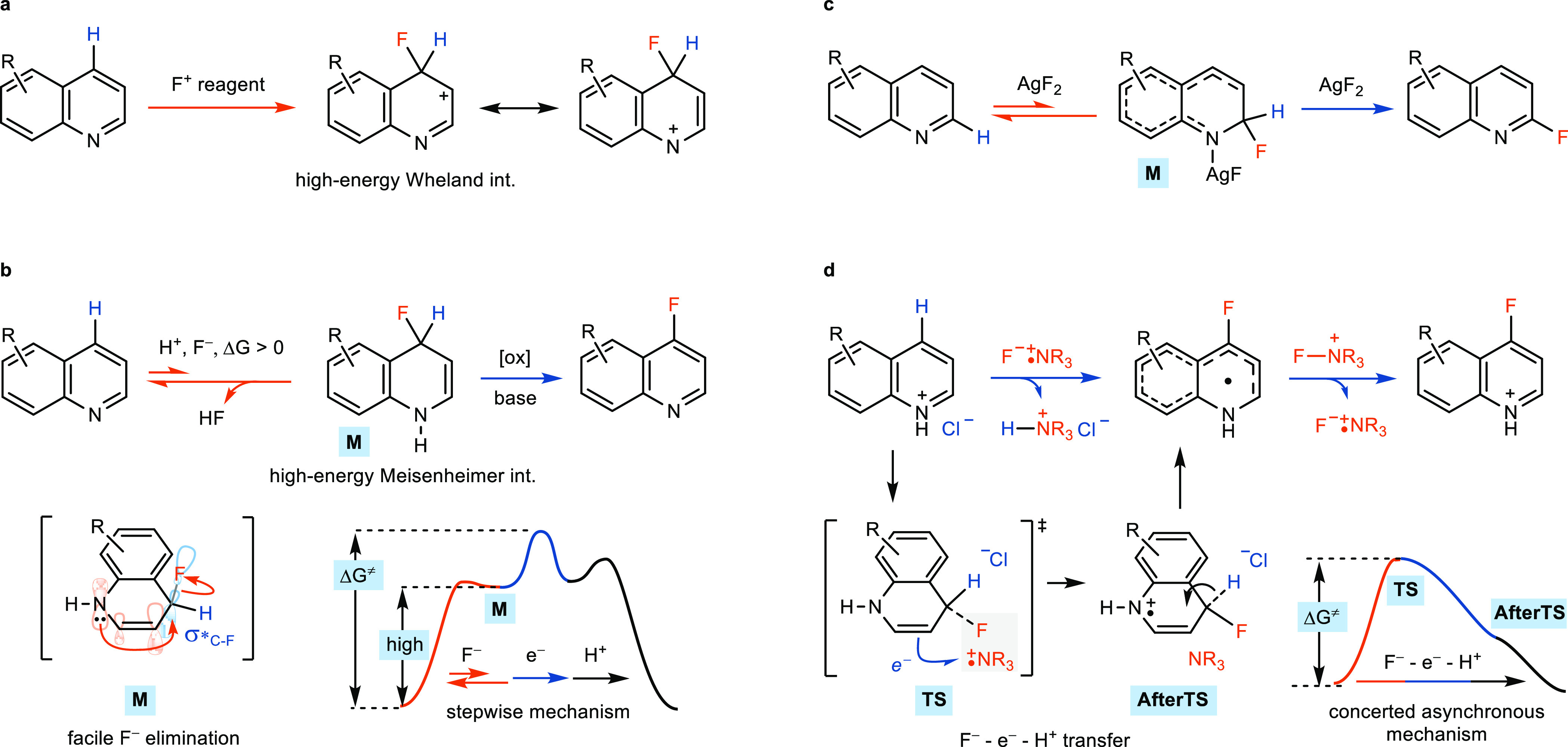
(a) High-energy Wheland intermediate in the S_E_Ar mechanism. (b) High-energy fluoride Meisenheimer intermediate in the S_N_Ar mechanism. (c) C2-selective fluorination of azaarenes with AgF_2_. (d) This work: fluorination of azaarenes via concerted nucleophilic substitution. M, Meisenheimer complex; TS, transition state.

Meisenheimer complexes are commonly considered intermediates in S_N_Ar reactions. However, in concerted nucleophilic aromatic substitutions (CS_N_Ar),^24,27,28^ Meisenheimer complexes are transition states, in which case the formation of high-energy intermediates is avoided. Therefore, a CS_N_Ar fluorination may provide the opportunity to avoid the high barriers that are associated with elimination of hydride from an already high-energy Meisenheimer complex to achieve azaarene C–H fluorination. We envisioned that when the fluoride attack on a protonated azaarene was coupled to an electron transfer, C–H bond heterolytic cleavage of a radical cation to release H^+^ would be more facile and allow a concerted process. Whether presumed F^–^, e^–^, and H^+^ transfer are concerted depends on the driving forces of both electron transfer (ET) and proton transfer (PT) as well as the distance between the fluoride Meisenheimer complexes and e^–^ and H^+^ acceptors.^29,30^ For example, preassociation of reactants can play an important role in concerted proton-coupled electron transfer.^29,30^ Therefore, we attempted to generate an ion pair TEDA^2+•^F^–^ (TEDA, *N*-(chloromethyl)triethylenediamine)^31−35^ in proximity to the protonated quinoline for potential concerted F^–^-e^–^ transfer, simultaneous with deprotonation (Figure 2a, chain propagation). We have previously made use of the high electron affinity of the doubly cationic radical TEDA^2+•^ (12.4 eV)^31^ for a charge-transfer-directed radical substitution. We therefore envisioned that TEDA^2+•^F^–^ may support an F^–^-e^–^-H^+^ transfer (Figure 2a, **A → D**) and then e^–^ transfer (**D → A**) chain for C–H fluorination upon further single-electron oxidation of the intermediate after F^–^ attack and C–H cleavage. In this case, the formation of the protonated Meisenheimer intermediate could be avoided, and the challenging fluorination could become feasible. The overall two-electron reaction Ar-H + Selectfluor → Ar-F + TEDA-H^2+^ would thus be achieved via two single-electron redox processes, and ET to sustain the chain process.

**Figure 2:**
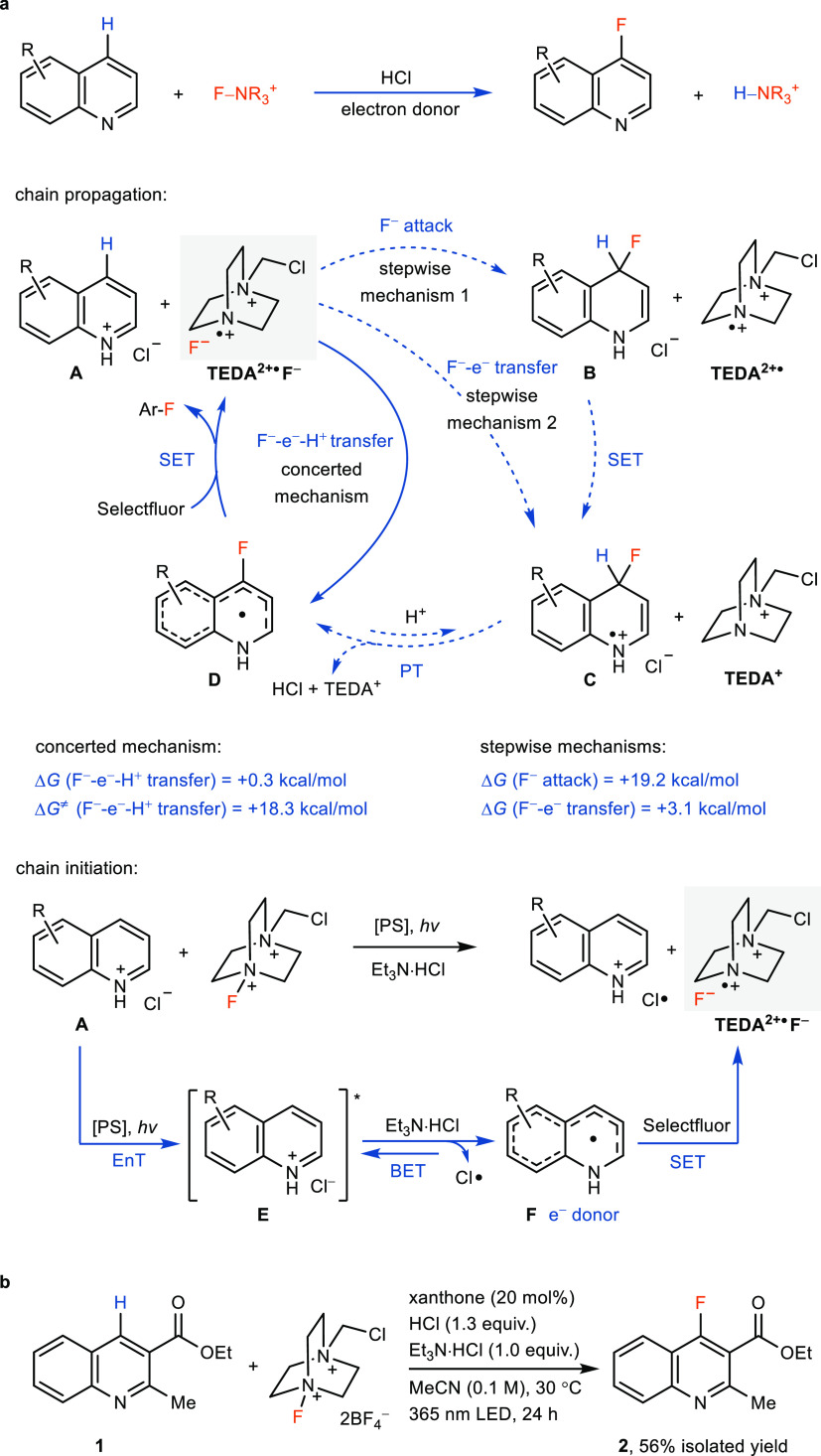
(a) Nucleophilic fluorination of azaarene via a radical chain mechanism; Δ*G*_solv298K_ in acetonitrile.^42^ (b) Representative fluorination reaction of quinoline. PS, photosensitizer; BET, back electron transfer; and PT, proton transfer.

Chain initiation from Selectfluor by single-electron reduction to yield the TEDA^2+•^F^–^ ion pair for chain propagation requires an electron donor that fulfills the following criteria: the electron donor is strong enough to reduce Selectfluor under acidic conditions for a sufficiently high concentration of TEDA^2+•^; and the electron donor should not engage in other side reactions with Selectfluor. An appropriate species that meets both requirements could be the reduced state of protonated azaarene itself, the *N*-heterocyclic π-radical **F**(36,37) (Figure 2a, chain initiation). Our group has reported that the excitation of an ion pair acridine-H^+^Cl^–^ followed by reductive quenching of its counteranion Cl^–^ can produce a *N*-heterocyclic π-radical,^38^ and we intended to apply the Cl^–^ quenching process to excited-state protonated azaarene **E** to generate a π-radical **F**. The electron donor **F** can thus donate an electron to Selectfluor to generate TEDA^2+•^F^–^ for chain propagation. The excited-state protonated azaarene **E** could originate from an energy transfer (EnT)^39^ between the ground-state protonated azaarene and a triplet-state photosensitizer (PS). For EnT to occur effectively, we chose xanthone as a photosensitizer due to its high triplet-state energy of *E*_T_ = 73.8 kcal/mol^40^ and a quinoline derivative as the substrate (*E*_T_ = 57.7 kcal/mol^41^ for protonated quinoline) to evaluate the fluorination of **1**. When HCl and Et_3_N·HCl were used as H^+^ and Cl^–^ donors, respectively, C4-fluorinated quinoline derivative **2** was obtained in 56% isolated yield upon irradiation with a 365 nm LED (Figure 2b).

## Concerted Mechanism

Upon initiation to generate TEDA^2+•^F^–^, fluoride-coupled electron transfer to protonated quinoline **A**, simultaneous with deprotonation, to generate **D** is associated with an activation energy of 18.3 kcal/mol (Figure 2a, chain propagation). The electron-rich π-radical **D** can be oxidized by Selectfluor to form the fluorinated product and regenerate TEDA^2+•^F^–^ to complete the chain, which is exergonic by 46.2 kcal/mol.

## Stepwise Mechanism 1

**Figure 3:**
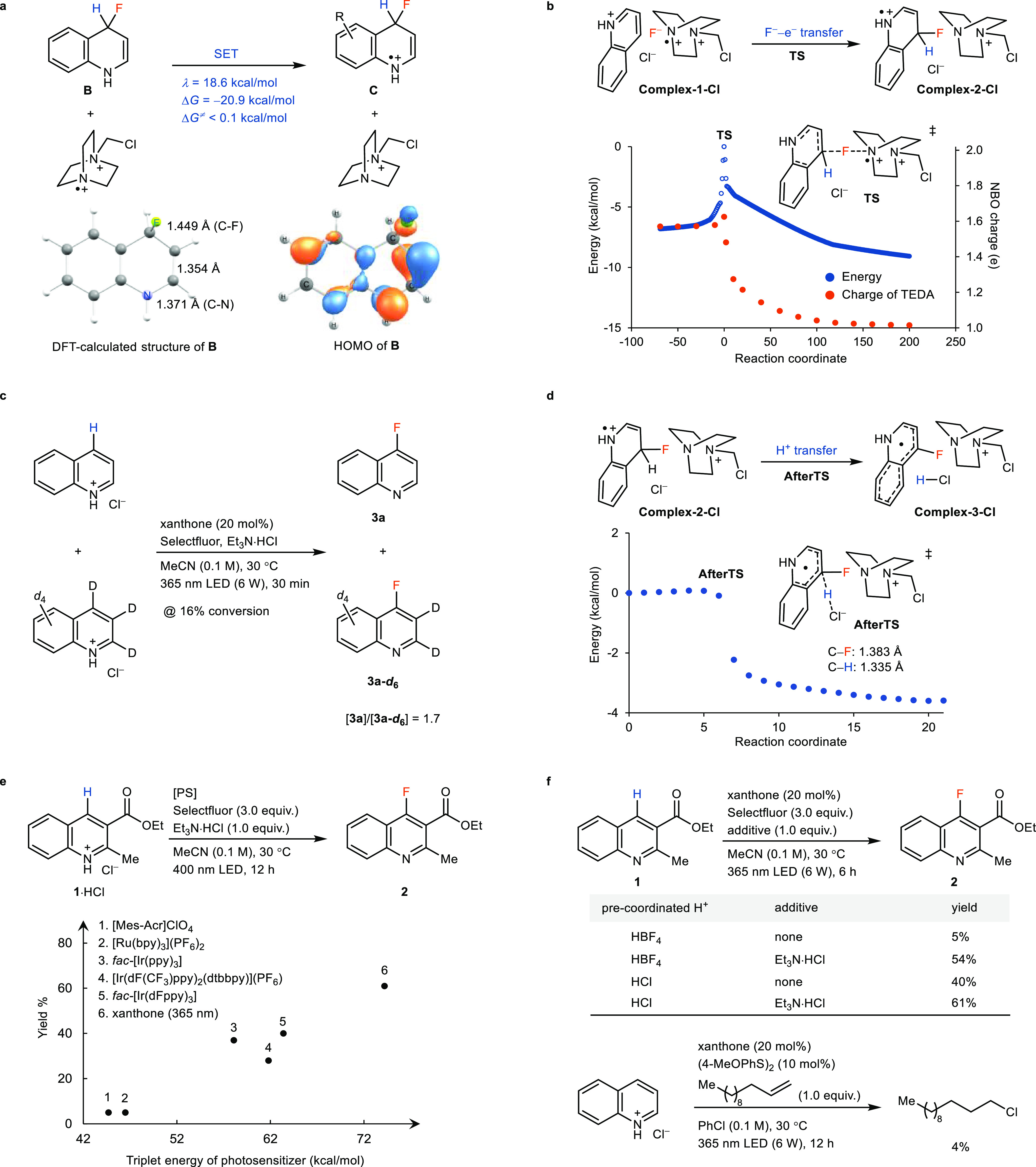
Mechanistic investigation. (a) Estimation of the Δ*G*^⧧^_SET_. DFT-calculated geometry and HOMO of **B**; isosurface value = 0.06. (b) Reaction coordinate of the fluoride-coupled electron transfer. (c) KIE from an intramolecular competition reaction. (d) Reaction coordinate of the PT. (e) Relationship between the yield of fluorination and *E*_T_ of photosensitizers. (f) Effect of chloride and a chlorine-radical-trapping reaction.

The fluoride of TEDA^2+•^F^–^ could attack quinoline hydrochloride **A** to form Meisenheimer intermediate **B** and TEDA^2+•^, followed by ET-PT or HAT of **B** (**A → B → D**). Estimation of the ET activation energy for the reaction between **B** and TEDA^2+•^ shows that there is no significant barrier (Figure 3a, Δ*G*^⧧^ < 0.1 kcal/mol), which indicates that it is unlikely for the Meisenheimer intermediate **B** and TEDA^2+•^ to form a stable complex. An internal reaction coordinate (IRC) analysis revealed that the transition state of TEDA^2+•^F^–^ attacking quinoline hydrochloride **A** directly leads to the product after ET, which excludes the existence of additional maxima along the reaction path (Figure 3b). Natural bond orbital analysis (NBO) of the transition state indicates a significant charge transfer process during the C–F bond formation, suggesting that an ET is coupled with the fluoride attack (Figure 3b). The calculated HOMO (Figure 3a, bottom) of intermediate **B** shows that the fluoride and enamine parts have the major contribution to the HOMO, which is remote from the proton donor. The lack of HOMO contribution from the C–H bond indicates that the C–H cleavage is difficult for a traditional HAT pathway,^43,44^ which would require the H^+^ and e^–^ to originate from the same donor group.^29,30,45^

## Stepwise Mechanism 2

A fluoride-coupled electron transfer mechanism to form a complex of dihydroquinoline radical cation^46,47^**C** and TEDA^+^, followed by a second step of deprotonation (**A → C → D**), could be feasible as well. Yet, we determined a kinetic isotope effect (KIE) of 1.7 from an intermolecular competition experiment with quinoline and quinoline-*d*_7_ (Figure 3c), which indicates a PT equilibrium before the product-formation ET (**D** → ArF), which is consistent with a concerted F^–^-e^–^-H^+^ mechanism (**A → D**) or a stepwise mechanism (**A → C → D**) with a reversible deprotonation. From the calculation, when Cl^–^ or TEDA^+^ were used as proton acceptors, the deprotonation proceeds without a significant energy barrier (Figure 3d, **AfterTS**, Δ*G*^⧧^ < 0.1 kcal/mol), which is consistent with an asynchronous concerted F^–^-e^–^-H^+^ transfer mechanism^48^ from **A** to **D**.

## Chain Initiation

The fluorination reaction efficiency correlates with the *E*_T_ of the photosensitizers but not with their reduction potentials, which is in agreement with an EnT process to form the triplet state of protonated quinoline (Figure 3e).^39^ Control experiments show the important role of the Cl^–^ counteranion and the Et_3_N·HCl additive to improve the reaction yield (Figure 3f), which could indicate a further reductive quenching process of the triplet state of protonated quinoline by Cl^–^. The observation of 1-chlorododecane in an intermolecular radical-trapping experiment with 1-dodecene is consistent with the formation of Cl·.^38,49^ According to an electrochemistry study, the additive Et_3_N·HCl (*E*_*p*__1/2_ = +0.94 V vs SCE) cannot be oxidized by ground-state protonated quinoline (*E*_*p*__1/2_ = −1.03 V vs SCE). However, the reduction of excited-state protonated quinoline (*E*_0–0_ = 2.65 V,^50^*E*_*p*__1/2_* = +1.62 V vs SCE) by Et_3_N·HCl is feasible. The quantum yield for fluorination of 3.5% is consistent with a back ET (BET) between *N*-heterocyclic π-radical **F** and Cl·.

**Figure 4:**
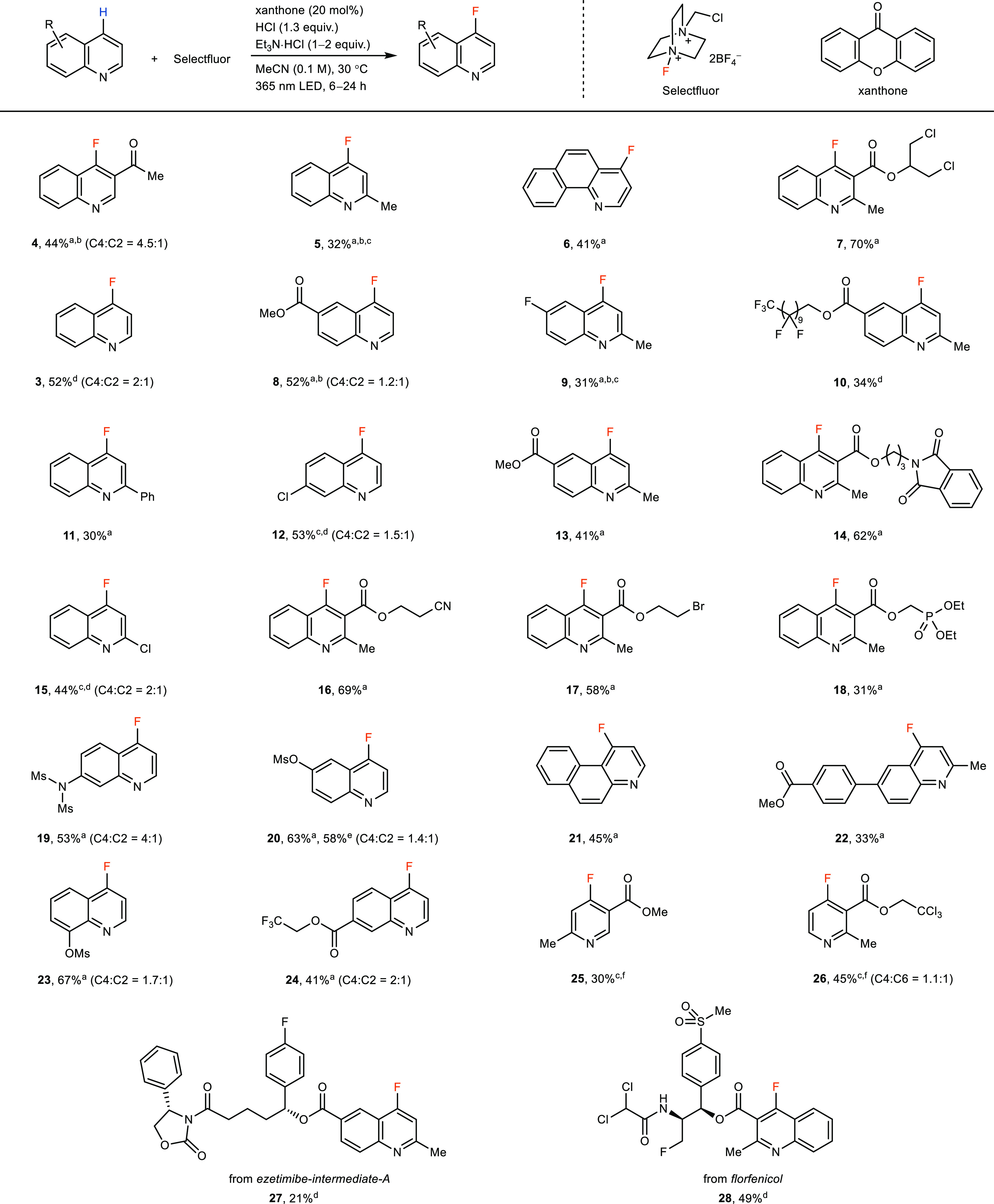
Substrate scope. ^a^Azaarene (0.4 mmol), HCl (1.3 equiv); then Selectfluor (3–4 equiv), xanthone (20 mol %), Et_3_N·HCl (1 equiv), MeCN (0.1 M), 365 nm LED (12 W), 30 °C, 24 h. ^b^Yield BRSM is more than 80%. ^c^Yields were determined by NMR. ^d^0.1 mmol scale. ^e^1 mmol scale. ^f^280 nm LED (10 W) and a quartz reaction vessel. All isomers of products or their hydrochloride salts have been isolated and characterized as analytically pure samples.

The fluorination reaction can be applied to small molecules with quinoline scaffolds, as shown in Figure 4. The conditions enable C–H fluorination in the presence of a range of functional groups, including esters (**7**, **8**, **13**), halogens (**9**, **12**, **17**), ketone (**4**), cyano (**16**), phosphoryl (**18**), alkyls (**5**, **7**), fluoroalkyls (**10**, **24**), amide (**20**), imide (**14**), carbamate (**27**), sulfonamide (**19**), sulfonates (**20**, **23**) and sulfone substituents (**28**). Substrates with electron-deficient or -neutral aryl groups (**14**, **19**, **22**, **28**) can be tolerated, but substrates with electron-rich aryl groups result in lower yields (for unsuccessful examples and examples with lower yields, see Table S2).^31−35^ Some substrates (**4**, **5**, **8**, **9**) exhibit yields based on recovered starting material (BRSM) greater than 80% but have only moderate yields of isolated pure products due to the incomplete reaction, likely due to product inhibition (see Supporting Information, p S15). Fluorination of unsubstituted quinoline (**3**) resulted in a 2:1 ratio of C4 and C2 products, slightly favoring the more electrophilic C4 site. A 4.5:1 of C4 and C2 fluorinated products was obtained in the case of 3-acetylquinoline (**4**), owing to enhanced C4 reactivity caused by electronic effects. The C4 selectivity is inconsistent with the fluorine atom transfer mechanism due to the polarity mismatch.^14^ The DFT-calculated LUMOs of protonated quinoline derivatives show that C4 has the largest contribution to the LUMO, suggesting that the site selectivity of the fluorination is consistent with the quinoline in the role of the electrophile (Supporting Information, p S73). In general, the selectivity for the C4 position is moderate but can be increased if Lewis acids are used instead of protic acid, presumably to coordinate to the quinoline nitrogen, and thereby sterically disfavor the reaction at C2 (see Supporting Information, pp S22–S23). Benzoquinolines such as **6** and **21** can be fluorinated successfully. Pyridine derivatives are not suitable substrates for the energy transfer because their *E*_T_ values (79.0 kcal/mol for pyridine)^40^ are too high for common photosensitizers such as xanthone (*E*_T_ = 73.8 kcal/mol).^40^ However, when direct excitation conditions were applied to access triplet-state pyridine–HCl complexes, several pyridine derivatives (**25**, **26**) could be fluorinated successfully.

The strategy of electron-transfer-enabled concerted nucleophilic aromatic substitution provides a conceptually new approach to azaarene C–H fluorination. Our work demonstrates how ion-coupled electron transfer avoids the formation of high-energy Meisenheimer intermediates and, therefore, can provide a hypothesis for the design of efficient catalysis by coupling ion transfer with redox processes, which may serve as a novel mechanistic basis for other nucleophilic aromatic C–H functionalization.

## Supporting Information Available

Experimental procedures and spectral data (PDF)

## References

[ref1] Hiyama T.; Yamamoto H. Organofluorine Building Blocks. In Organofluorine Compounds; Springer: Berlin, 2000; pp 77–118.

[ref2] de la Torre B. G.; Albericio F. The Pharmaceutical Industry in 2021. An Analysis of FDA Drug Approvals from the Perspective of Molecules. Molecules 2022, 27, 1075 10.3390/molecules27031075. 35164339 PMC8839730

[ref3] Li J.; Zhang X.; Jin H.; Fan J.; Flores H.; Perlmutter J. S.; Tu Z. Synthesis of fluorine-containing phosphodiesterase 10A (PDE10A) inhibitors and the in vivo evaluation of F-18 labeled PDE10A PET tracers in rodent and nonhuman primate. J. Med. Chem. 2015, 58, 8584–8600. 10.1021/acs.jmedchem.5b01205. 26430878 PMC4697445

[ref4] Shannon R. D. Revised effective ionic radii and systematic studies of interatomic distances in halides and chalcogenides. Acta Crystallogr. A 1976, 32, 751–767. 10.1107/S0567739476001551.

[ref5] Szpera R.; Moseley D. F.; Smith L. B.; Sterling A. J.; Gouverneur V. The Fluorination of C-H bonds: developments and perspectives. Angew. Chem., Int. Ed. 2019, 58, 14824–14848. 10.1002/anie.201814457. 30759327

[ref6] Zhang L.; Ritter T. A perspective on late-stage aromatic C-H bond functionalization. J. Am. Chem. Soc. 2022, 144, 2399–2414. 10.1021/jacs.1c10783. 35084173 PMC8855345

[ref7] Yamamoto K.; Li J.; Garber J. A.; Rolfes J. D.; Boursalian G. B.; Borghs J. C.; Genicot C.; Jacq J.; van Gastel M.; Neese F.; Ritter T. Palladium-catalysed electrophilic aromatic C-H fluorination. Nature 2018, 554, 511–514. 10.1038/nature25749. 29469096

[ref8] Li J.; Chen J.; Sang R.; Ham W. S.; Plutschack M. B.; Berger F.; Chabbra S.; Schnegg A.; Genicot C.; Ritter T. Photoredox catalysis with aryl sulfonium salts enables site-selective late-stage fluorination. Nat. Chem. 2020, 12, 56–62. 10.1038/s41557-019-0353-3. 31767996

[ref9] Chen W.; Huang Z.; Tay N. E.; Giglio B.; Wang M.; Wang H.; Wu Z.; Nicewicz D. A.; Li Z. Direct arene C-H fluorination with 18F- via organic photoredox catalysis. Science 2019, 364, 1170–1174. 10.1126/science.aav7019. 31221856 PMC6680023

[ref10] Fier P. S.; Hartwig J. F. Selective C-H fluorination of pyridines and diazines inspired by a classic amination reaction. Science 2013, 342, 956–960. 10.1126/science.1243759. 24264986

[ref11] Fier P. S.; Hartwig J. F. Synthesis and late-stage functionalization of complex molecules through C-H fluorination and nucleophilic aromatic substitution. J. Am. Chem. Soc. 2014, 136, 10139–10147. 10.1021/ja5049303. 24918484 PMC4227713

[ref12] Murakami K.; Yamada S.; Kaneda T.; Itami K. C-H functionalization of azines. Chem. Rev. 2017, 117, 9302–9332. 10.1021/acs.chemrev.7b00021. 28445033

[ref13] Zhou F. Y.; Jiao L. Recent developments in transition-metal-free functionalization and derivatization reactions of pyridines. Synlett 2021, 32, 159–178. 10.1055/s-0040-1706552.

[ref14] Proctor R. S.; Phipps R. J. Recent advances in Minisci-type reactions. Angew. Chem., Int. Ed. 2019, 58, 13666–13699. 10.1002/anie.201900977. 30888102

[ref15] Holmberg-Douglas N.; Nicewicz D. A. Photoredox-catalyzed C-H functionalization reactions. Chem. Rev. 2022, 122, 1925–2016. 10.1021/acs.chemrev.1c00311. 34585909 PMC8939264

[ref16] Fujiwara Y.; Dixon J. A.; O’Hara F.; Funder E. D.; Dixon D. D.; Rodriguez R. A.; Baxter R. D.; Herlé B.; Sach N.; Collins M. R.; Ishihara Y.; Baran P. S. Practical and innate carbon-hydrogen functionalization of heterocycles. Nature 2012, 492, 95–99. 10.1038/nature11680. 23201691 PMC3518649

[ref17] Kim J. H.; Constantin T.; Simonetti M.; Llaveria J.; Sheikh N. S.; Leonori D. A radical approach for the selective C-H borylation of azines. Nature 2021, 595, 677–683. 10.1038/s41586-021-03637-6. 34015802

[ref18] Liu S.; Pan P.; Fan H.; Li H.; Wang W.; Zhang Y. Photocatalytic C-H silylation of heteroarenes by using trialkylhydrosilanes. Chem. Sci. 2019, 10, 3817–3825. 10.1039/C9SC00046A. 31015923 PMC6457191

[ref19] Josephitis C. M.; Nguyen H. M.; McNally A. Late-Stage C-H Functionalization of Azines. Chem. Rev. 2023, 123, 7655–7691. 10.1021/acs.chemrev.2c00881. 37134187 PMC10631472

[ref20] Zhang X.; Nottingham K. G.; Patel C.; Alegre-Requena J. V.; Levy J. N.; Paton R. S.; McNally A. Phosphorus-mediated sp2-sp3 couplings for C-H fluoroalkylation of azines. Nature 2021, 594, 217–222. 10.1038/s41586-021-03567-3. 33910228

[ref21] Hilton M. C.; Zhang X.; Boyle B. T.; Alegre-Requena J. V.; Paton R. S.; McNally A. Heterobiaryl synthesis by contractive C-C coupling via P(V) intermediates. Science 2018, 362, 799–804. 10.1126/science.aas8961. 30442804 PMC6814017

[ref22] Campbell M. G.; Ritter T. Modern carbon-fluorine bond forming reactions for aryl fluoride synthesis. Chem. Rev. 2015, 115, 612–633. 10.1021/cr500366b. 25474722

[ref23] Watson D. A.; Su M.; Teverovskiy G.; Zhang Y.; García-Fortanet J.; Kinzel T.; Buchwald S. L. Formation of ArF from LPdAr (F): catalytic conversion of aryl triflates to aryl fluorides. Science 2009, 325, 1661–1664. 10.1126/science.1178239. 19679769 PMC3038120

[ref24] Neumann C. N.; Hooker J. M.; Ritter T. Concerted nucleophilic aromatic substitution with ^19^F- and ^18^F-. Nature 2016, 534, 369–373. 10.1038/nature17667. 27487221

[ref25] Ghiazza C.; Faber T.; Gómez-Palomino A.; Cornella J. Deaminative chlorination of aminoheterocycles. Nat. Chem. 2022, 14, 78–84. 10.1038/s41557-021-00812-0. 34916597 PMC8755540

[ref26] See Y. Y.; Morales-Colón M. T.; Bland D. C.; Sanford M. S. Development of S_N_Ar nucleophilic fluorination: a fruitful academia-industry collaboration. Acc. Chem. Res. 2020, 53, 2372–2383. 10.1021/acs.accounts.0c00471. 32969213

[ref27] Kwan E. E.; Zeng Y.; Besser H. A.; Jacobsen E. N. Concerted nucleophilic aromatic substitutions. Nat. Chem. 2018, 10, 917–923. 10.1038/s41557-018-0079-7. 30013193 PMC6105541

[ref28] Rohrbach S.; Smith A. J.; Pang J. H.; Poole D. L.; Tuttle T.; Chiba S.; Murphy J. A. Concerted nucleophilic aromatic substitution reactions. Angew. Chem., Int. Ed. 2019, 58, 16368–16388. 10.1002/anie.201902216. PMC6899550 30990931

[ref29] Tyburski R.; Liu T.; Glover S. D.; Hammarström L. Proton-coupled electron transfer guidelines, fair and square. J. Am. Chem. Soc. 2021, 143, 560–576. 10.1021/jacs.0c09106. 33405896 PMC7880575

[ref30] Weinberg D. R.; Gagliardi C. J.; Hull J. F.; Murphy C. F.; Kent C. A.; Westlake B. C.; Paul A.; Ess D. H.; McCafferty D. G.; Meyer T. J. Proton-coupled electron transfer. Chem. Rev. 2012, 112, 4016–4093. 10.1021/cr200177j. 22702235

[ref31] Boursalian G. B.; Ham W. S.; Mazzotti A. R.; Ritter T. Charge-transfer-directed radical substitution enables para-selective C-H functionalization. Nat. Chem. 2016, 8, 810–815. 10.1038/nchem.2529. 27442288 PMC4957710

[ref32] Serpier F.; Pan F.; Ham W. S.; Jacq J.; Genicot C.; Ritter T. Selective Methylation of Arenes: A Radical C-H Functionalization/Cross-Coupling Sequence. Angew. Chem., Int. Ed. 2018, 57, 10697–10701. 10.1002/anie.201804628. 29893494

[ref33] Aguilar Troyano F. J.; Merkens K.; Gómez-Suárez A. Selectfluor^®^ Radical Dication (TEDA^2+.^)-A Versatile Species in Modern Synthetic Organic Chemistry. Asian J. Org. Chem. 2020, 9, 992–1007. 10.1002/ajoc.202000196.

[ref34] Xiang M.; Xin Z. K.; Chen B.; Tung C. H.; Wu L. Z. Exploring the reducing ability of organic dye (Acr^+^-Mes) for fluorination and oxidation of benzylic C (sp^3^)-H bonds under visible light irradiation. Org. Lett. 2017, 19, 3009–3012. 10.1021/acs.orglett.7b01270. 28530821

[ref35] Ventre S.; Petronijevic F. R.; MacMillan D. W. Decarboxylative fluorination of aliphatic carboxylic acids via photoredox catalysis. J. Am. Chem. Soc. 2015, 137, 5654–5657. 10.1021/jacs.5b02244. 25881929 PMC4862610

[ref36] Rössler S. L.; Jelier B. J.; Magnier E.; Dagousset G.; Carreira E. M.; Togni A. Pyridinium Salts as Redox-Active Functional Group Transfer Reagents. Angew. Chem., Int. Ed. 2020, 59, 9264–9280. 10.1002/anie.201911660. 31600423

[ref37] Wang G.; Zhang H.; Zhao J.; Li W.; Cao J.; Zhu C.; Li S. Homolytic cleavage of a B-B bond by the cooperative catalysis of two Lewis bases: computational design and experimental verification. Angew. Chem., Int. Ed. 2016, 55, 5985–5989. 10.1002/anie.201511917. 27061603

[ref38] Kim J.; Sun X.; van der Worp B. A.; Ritter T. Anti-Markovnikov hydrochlorination and hydronitrooxylation of α-olefins. Nat. Catal. 2023, 6, 196–203. 10.1038/s41929-023-00914-7.

[ref39] Strieth-Kalthoff F.; James M. J.; Teders M.; Pitzer L.; Glorius F. Energy transfer catalysis mediated by visible light: principles, applications, directions. Chem. Soc. Rev. 2018, 47, 7190–7202. 10.1039/C8CS00054A. 30088504

[ref40] Morov S. L.; Carmicheal I.; Hug G. L. Handbook of Photochemistry; Marcel Dekker, Inc.: New York, 1993.

[ref41] Ma J.; Chen S.; Bellotti P.; Guo R.; Schäfer F.; Heusler A.; Zhang X.; Daniliuc C.; Brown M. K.; Houk K. N.; Glorius F. Photochemical intermolecular dearomative cycloaddition of bicyclic azaarenes with alkenes. Science 2021, 371, 1338–1345. 10.1126/science.abg0720. 33766881 PMC7610643

[ref42] Neese F. The ORCA program system. WIREs Comput. Mol. Sci. 2012, 2, 73–78. 10.1002/wcms.81.

[ref43] Pitts C. R.; Ling B.; Woltornist R.; Liu R.; Lectka T. Triethylborane-initiated radical chain fluorination: a synthetic method derived from mechanistic insight. J. Org. Chem. 2014, 79, 8895–8899. 10.1021/jo501520e. 25137438

[ref44] Xia J. B.; Zhu C.; Chen C. Visible light-promoted metal-free C-H activation: diarylketone-catalyzed selective benzylic mono-and difluorination. J. Am. Chem. Soc. 2013, 135, 17494–17500. 10.1021/ja410815u. 24180320 PMC3874084

[ref45] Markle T. F.; Darcy J. W.; Mayer J. M. A new strategy to efficiently cleave and form C-H bonds using proton-coupled electron transfer. Sci. Adv. 2018, 4, eaat5776 10.1126/sciadv.aat5776. 30027119 PMC6044737

[ref46] Zhu X. Q.; Yang Y.; Zhang M.; Cheng J. P. First estimation of C4-H bond dissociation energies of NADH and its radical cation in aqueous solution. J. Am. Chem. Soc. 2003, 125, 15298–15299. 10.1021/ja0385179. 14664567

[ref47] Zhu X. Q.; Li H. R.; Li Q.; Ai T.; Lu J. Y.; Yang Y.; Cheng J. P. Determination of the C4-H Bond Dissociation Energies of NADH Models and Their Radical Cations in Acetonitrile. Chem. Eur. J. 2003, 9, 871–880. 10.1002/chem.200390108. 12584702

[ref48] Labet V.; Morell C.; Toro-Labbé A.; Grand A. Is an elementary reaction step really elementary? Theoretical decomposition of asynchronous concerted mechanisms. Phys. Chem. Chem. Phys. 2010, 12, 4142–4151. 10.1039/b924589h. 20379505

[ref49] Poulet G.; Laverdet G.; Jourdain J. L.; Le Bras G. Kinetic study of the reactions of acetonitrile with chlorine (Cl) and hydroxyl radicals. J. Phys. Chem. 1984, 88, 6259–6263. 10.1021/j150669a041.

[ref50] Komura A.; Uchida K.; Yagi M.; Higuchi J. Electron spin resonance and phosphorescence of quinoline, isoquinoline and their protonated cations in the phosphorescent triplet states. J. Photochem. Photobiol., A 1988, 42, 293–300. 10.1016/1010-6030(88)80072-8.

