# Supplementary material for: Electron-Transfer-Enabled Concerted Nucleophilic Fluorination of Azaarenes: Selective C–H Fluorination of Quinolines
Source: J Am Chem Soc. 2023 Sep 11;145(37):20182–8. doi: 10.1021/jacs.3c07119 (PMC10515641; doi:10.1021/jacs.3c07119)
Supplement: Supplementary file 1 — ja3c07119_si_001.pdf [file ja3c07119_si_001.pdf]

SUPPLEMENTARY INFORMATION

Electron-transfer-enabled concerted nucleophilic fluorination of azaarenes: selective C–H fluorination of quinolines

Li Zhang[1], Jiyao Yan[1,2], Dilgam Ahmadli[1,2], Zikuan Wang[1], Tobias Ritter[1]*

[1] Max-Planck-Institute für Kohlenforschung, Kaiser-Wilhelm Platz 1, D-45470 Mülheim an der Ruhr, Germany.

[2] Institute of Organic Chemistry, RWTH Aachen University, Landoltweg 1, 52074 Aachen, Germany.

*E-mail: ritter@kofo.mpg.de

TABLE OF CONTENTS

TABLE OF CONTENTS 2

MATERIALS AND METHODS 10

EXPERIMENTAL DATA 11

General procedure and reaction optimization for the fluorination reaction 11

General procedure for the fluorination reaction 11

Table S1. Reaction condition optimization 13

Fluorination of azaarenes 16

Ethyl 4-fluoro-2-methylquinoline-3-carboxylate (**2**) 16

3-Acetyl-4-fluoroquinoline (**4a**) and 3-acetyl-2-fluoroquinoline (**4b**) 17

4-Fluoro-2-methylquinoline (**5**) 18

4-Fluorobenzo[*h*]quinoline (**6**) 19

Chloroalkyl 4-fluoro-2-methylquinoline-3-carboxylate **7** 20

4-Fluoroquinoline (**3a**) and 2-fluoroquinoline (**3b**) 21

Methyl 4-fluoroquinoline-6-carboxylate (**8a**) and methyl 2-fluoroquinoline-6-carboxylate (**8b**) 24

4,6-Difluoro-2-methylquinoline (**9**) 25

Polyfluoroalkyl 4-fluoro-2-methylquinoline-6-carboxylate **10** 26

4-Fluoro-2-phenylquinoline (**11**) 27

7-Chloro-4-fluoroquinoline (**12a**) and 7-chloro-2-fluoroquinoline (**12b**) 29

Methyl 4-fluoro-2-methylquinoline-6-carboxylate (**13**) 30

3-(Phthalimidyl)propyl 4-fluoro-2-methylquinoline-3-carboxylate (**14**) 31

4-Fluoro-2-chloroquinoline (**15**) 32

2-Cyanoethyl 4-fluoro-2-methylquinoline-3-carboxylate (**16**) 33

2-Bromoethyl 4-fluoro-2-methylquinoline-3-carboxylate (**17**) 34

Phosphorylmethyl 4-fluoro-2-methylquinoline-3-carboxylate **18** 35

4-Fluoro-7-sulfonamidylquinoline **19a** and 2-fluoro-7-sulfonamidylquinoline **19b** 36

4-Fluoro-6-sulfonyloxyquinoline **20a** and 2-fluoro-6-sulfonyloxyquinoline **20b** 38

4-Fluorobenzo[*f*]quinoline (**21**) 39

Methyl 4-(4-fluoro-2-methylquinolin-6-yl)benzoate (**22**) 40

4-Fluoro-8-sulfonyloxyquinoline **23a** and 2-fluoro-8-sulfonyloxyquinoline **23b** 41

Trifluoroethyl 4-fluoro-quinoline-7-carboxylate (**24a**) and trifluoroethyl 4-fluoro-quinoline-7-carboxylate (**24b**) 42

Methyl 4-fluoro-6-methylnicotinate (**25**) 43

Trichloroethyl 4-fluoro-2-methylnicotinate (**26a**) and trichloroethyl 6-fluoro-2-methylnicotinate (**26b**) 45

Fluoroezetimibe-intermediate-A derivative **27** 46

Fluoroflorfenicol derivative **28** 47

Table S2. Other tested substrates for photo-initiated conditions 48
2-Chloro-4-fluoronicotinic amide **29** 49
Preparation of starting materials 50
Chloroalkyl 2-methylquinoline-3-carboxylate **S7** 50
Polyfluoroalkyl 2-methylquinoline-6-carboxylate **S10** 51
3-(Phthalimidyl)propyl 2-methylquinoline-3-carboxylate (**S14**) 52
2-Cyanoethyl 2-methylquinoline-3-carboxylate (**S16**) 53
2-Bromoethyl 2-methylquinoline-3-carboxylate (**S17**) 54
Phosphorylmethyl 2-methylquinoline-3-carboxylate **S18** 54
7-Sulfonamidylquinoline **S19** 55
6-Sulfonyloxyquinoline **S20** 56
Methyl 4-(2-methylquinolin-6-yl)benzoate (**S22**) 57
8-Sulfonyloxyquinoline **S23** 57
Trifluoroethyl quinoline-7-carboxylate (**S24**) 58
Trichloroethyl 2-methylnicotinate (**S26**) 59
Ezetimibe-intermediate-A derivative **S27** 60
Florfenicol derivative **S28** 61
MECHANISTIC STUDIES 62
Kinetic isotope effect 62
KIE determined from intermolecular competition kinetics 62
KIE determined from parallel reactions 63
X-Ray Crystallographic Data 65
3-Acetylquinoline hydrochloride (CCDC 2212696) 65
Table S3. Crystallographic data 66
DFT Calculation 67
Methods 67
Discussion 67
Table S4. Conformation analysis of Complex-2 71
Calculated coordinates 74
Stern–Volmer luminescence quenching experiments 105
General Procedure 105
Table S5. Quenching rate coefficients 106
Intermolecular radical-trapping experiment 107
1-Chlorododecane 107

Quantum yield measurement 109
Photon flux measurement 109
Table S6. Calculation of photon flux 109
Quantum yield of azaarene fluorination 110
Quantum yield of hydrochlorination 110
Electrochemistry study 112
General Procedure 112
SPECTROSCOPIC DATA 114
^{1}H NMR of **2** 114
^{13}C NMR of **2** 115
^{19}F NMR of **2** 116
^{1}H NMR of **4a** 117
^{13}C NMR of **4a** 118
^{19}F NMR of **4a** 119
^{1}H NMR of **4b** 120
^{13}C NMR of **4b** 121
^{19}F NMR of **4b** 122
^{1}H NMR of **5·HCl** 123
^{13}C NMR of **5·HCl** 124
^{19}F NMR of **5·HCl** 125
^{1}H NMR of **6** 126
^{13}C NMR of **6** 127
^{19}F NMR of **6** 128
^{1}H NMR of **7** 129
^{13}C NMR of **7** 130
^{19}F NMR of **7** 131
^{1}H NMR of **3a·HCl** 132
^{13}C NMR of **3a·HCl** 133
^{19}F NMR of **3a·HCl** 134

^{1}H NMR of **3b** 135

^{13}C NMR of **3b** 136

^{19}F NMR of **3b** 137

^{1}H NMR of **8a** 138

^{13}C NMR of **8a** 139

^{19}F NMR of **8a** 140

^{1}H NMR of **8b** 141

^{13}C NMR of **8b** 142

^{19}F NMR of **8b** 143

^{1}H NMR of **9·HCl** 144

^{13}C NMR of **9·HCl** 145

^{19}F NMR of **9·HCl** 146

^{1}H NMR of **10** 147

^{13}C NMR of **10** 148

^{19}F NMR of **10** 149

^{1}H NMR of **11** 150

^{13}C NMR of **11** 151

^{19}F NMR of **11** 152

^{1}H NMR of **12a** 153

^{13}C NMR of **12a** 154

^{19}F NMR of **12a** 155

^{1}H NMR of **12b** 156

^{13}C NMR of **12b** 157

^{19}F NMR of **12b** 158

^{1}H NMR of **13** 159

^{13}C NMR of **13** 160

^{19}F NMR of **13** 161

^{1}H NMR of **14** 162

^{13}C NMR of **14** 163

^{19}F NMR of **14** 164
^{1}H NMR of **15** 165
^{13}C NMR of **15** 166
^{19}F NMR of **15** 167
^{1}H NMR of **16** 168
^{13}C NMR of **16** 169
^{19}F NMR of **16** 170
^{1}H NMR of **17** 171
^{13}C NMR of **17** 172
^{19}F NMR of **17** 173
^{1}H NMR of **18** 174
^{13}C NMR of **18** 175
^{19}F NMR of **18** 176
^{31}P NMR of **18** 177
^{1}H NMR of **19a** 178
^{13}C NMR of **19a** 179
^{19}F NMR of **19a** 180
^{1}H NMR of **19b** 181
^{13}C NMR of **19b** 182
^{19}F NMR of **19b** 183
^{1}H NMR of **20a** 184
^{13}C NMR of **20a** 185
^{19}F NMR of **20a** 186
^{1}H NMR of **20b** 187
^{13}C NMR of **20b** 188
^{19}F NMR of **20b** 189
^{1}H NMR of **21** 190
^{13}C NMR of **21** 191
^{19}F NMR of **21** 192

^{1}H NMR of **22** 193
^{13}C NMR of **22** 194
^{19}F NMR of **22**........ 195
^{1}H NMR of **23a** 196
^{13}C NMR of **23a** 197
^{19}F NMR of **23a**........ 198
^{1}H NMR of **23b** 199
^{13}C NMR of **23b** 200
^{19}F NMR of **23b** 201
^{1}H NMR of **24a** 202
^{13}C NMR of **24a** 203
^{19}F NMR of **24a**........ 204
^{1}H NMR of **24b** 205
^{13}C NMR of **24b** 206
^{19}F NMR of **24b** 207
^{1}H NMR of **25** 208
^{13}C NMR of **25** 209
^{19}F NMR of **25**........ 210
^{1}H NMR of **26a** 211
^{13}C NMR of **26a** 212
^{19}F NMR of **26a**........ 213
^{1}H NMR of **26b** 214
^{13}C NMR of **26b** 215
^{19}F NMR of **26b** 216
^{1}H NMR of **27** 217
^{13}C NMR of **27** 218
^{19}F NMR of **27**........ 219
^{1}H NMR of **28** 220
^{13}C NMR of **28** 221

^{19}F NMR of **28** ... 222
^{1}H NMR of **29** ... 223
^{13}C NMR of **29** ... 224
^{19}F NMR of **29** ... 225
^{1}H NMR of **S7** ... 226
^{13}C NMR of **S7** ... 227
^{1}H NMR of **S10** ... 228
^{13}C NMR of **S10** ... 229
^{19}F NMR of **S10** ... 230
^{1}H NMR of **S14** ... 231
^{13}C NMR of **S14** ... 232
^{1}H NMR of **S16** ... 233
^{13}C NMR of **S16** ... 234
^{1}H NMR of **S17** ... 235
^{13}C NMR of **S17** ... 236
^{1}H NMR of **S18** ... 237
^{13}C NMR of **S18** ... 238
^{31}P NMR of **S18** ... 239
^{1}H NMR of **S19** ... 240
^{13}C NMR of **S19** ... 241
^{1}H NMR of **S20** ... 242
^{13}C NMR of **S20** ... 243
^{1}H NMR of **S22** ... 244
^{13}C NMR of **S22** ... 245
^{1}H NMR of **S23** ... 246
^{13}C NMR of **S23** ... 247
^{1}H NMR of **S24** ... 248
^{13}C NMR of **S24** ... 249
^{19}F NMR of **S24** ... 250

^{1}H NMR of **S26** 251
^{13}C NMR of **S26** 252
^{1}H NMR of **S27** 253
^{13}C NMR of **S27** 254
^{19}F NMR of **S27** 255
^{1}H NMR of **S28** 256
^{13}C NMR of **S28** 257
^{19}F NMR of **S28** 258
REFERENCES 259

MATERIALS AND METHODS

All reactions were carried out under ambient atmosphere unless otherwise stated and monitored by thin-layer chromatography (TLC). High-resolution mass spectra were obtained using *Q Exactive Plus* from *Thermo*. Concentration under reduced pressure was performed by rotary evaporation at 25–40 °C at an appropriate pressure. Purified compounds were further dried under vacuum (10^{-6} – 10^{-3} bar). Yields refer to purified and spectroscopically pure compounds, unless otherwise stated.

Solvents

Anhydrous solvents were obtained from Phoenix Solvent Drying Systems. All deuterated solvents were purchased from Euriso-Top®.

Chromatography

Thin layer chromatography (TLC) was performed using EMD TLC plates pre-coated with 250 μm thickness silica gel 60 F_{254} plates and visualized by fluorescence quenching under UV light and $KMnO_4$ stain. Flash column chromatography was performed using silica gel (40–63 µm particle size) purchased from Geduran®. Preparative HPLC was performed on a Shimadzu SPD-20A/LC-20AP/FRC-10A.

Spectroscopy and Instruments

NMR spectra were recorded on a Bruker *Ascend*™ 500 spectrometer operating at 500 MHz, 471 MHz, 203 MHz, and 126 MHz, for ^{1}H, ^{19}F, ^{31}P, and ^{13}C acquisitions, respectively; or on a Varian Unity/Inova 600 spectrometer operating at 600 MHz, 565 MHz, and 151 MHz for ^{1}H, ^{19}F, and ^{13}C acquisitions, respectively. Chemical shifts are reported in ppm with the solvent residual peak as the internal standard. For ^{1}H NMR: $CDCl_3$, δ 7.26, CD_3CN, δ 1.96. For ^{13}C NMR: $CDCl_3$, δ 77.16, CD_3CN, δ 1.79[1]. ^{19}F NMR spectra were referenced using a unified chemical shift scale based on the ^{1}H resonance of tetramethylsilane (1% (v/v) solution in the respective solvent). Data is reported as follows: s = singlet, d = doublet, t = triplet, q = quartet, m = multiplet, br = broad; coupling constants in Hz; integration. NMR spectra were viewed and processed by Mestrenova.

Starting materials

All substrates were used as received from commercial suppliers, unless otherwise stated. Chemicals were purchased from *Sigma-Aldrich*, *TCI*, *Alfa Aesar, Apollo Scientific, Abcr, BLDpharm or Fluorochem*. Substrates **S6**, **S10**, **S14, S16, S17, S18, S28** were prepared according to a published procedure[2].

EXPERIMENTAL DATA

General procedure and reaction optimization for the fluorination reaction

General procedure for the fluorination reaction

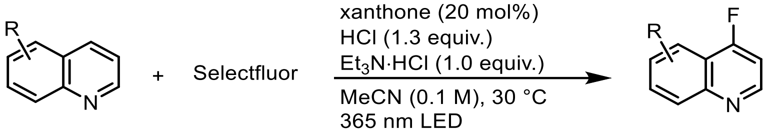

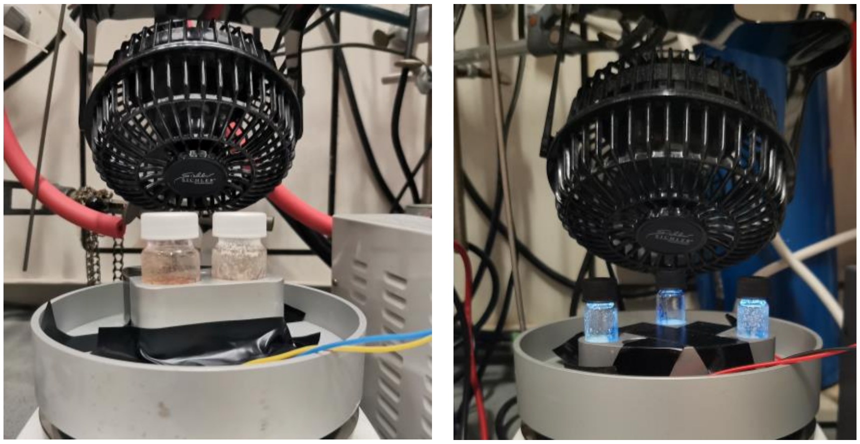

Figure S1. Photo of the irradiation setup for the fluorination of fused-azaarene derivatives (left: 0.4 mmol and 1 mmol scale; right: 0.1 mmol scale)

Under an ambient atmosphere, a 20-mL borosilicate vial equipped with a magnetic stir bar was charged with azaarene (0.400 mmol, 1.00 equiv.), followed by dry DCM (4.0 mL, c = 0.1 M). Subsequently, 4.0 M HCl solution in dioxane (133 μL, 0.52 mmol, 1.3 equiv.) was added. The mixture was stirred at ambient temperature for 10 min. After that, the solvent was removed by a rotary evaporator. Under a nitrogen atmosphere, to the vial were then added xanthone (15.6 mg, 80 μmol, 20 mol%), Selectfluor (424 mg, 1.20 mmol, 3.00 equiv.), Et_3N·HCl (54.8 mg, 0.400 mmol, 1.00 equiv.), and dry acetonitrile (4.0 mL, c = 0.1 M). The vial was sealed with a septum cap. Then, the vial was placed onto an aluminum plate fitted with a 12 W high-power single LED plate (EpiLED Chip, size: 10 × 10 mm, λ_{max} = 365 nm, 1.2 A, 9–11 V). Another aluminum block was utilized to hold the vial. The temperature was kept at approximately 30 °C through the use of a cooling fan. The reaction mixture was stirred and irradiated for 24 hours, then, 2 mL of saturated aqueous Na_2CO_3 were added. To obtain purified material, extraction followed by column chromatography on silica gel was conducted. Where necessary, further purification was accomplished by preparative HPLC.

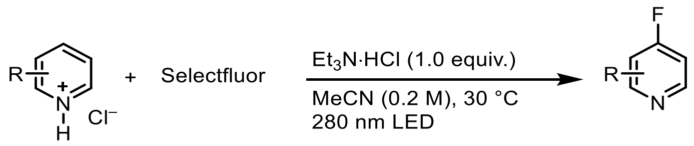

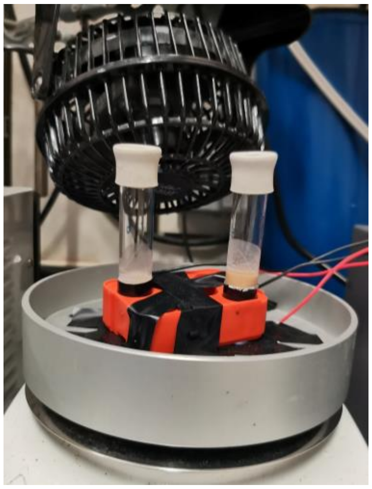

Figure S2. Photo of the irradiation setup for the fluorination of pyridine derivatives

Under a nitrogen atmosphere, a 5-mL quartz vial equipped with a magnetic stir bar was charged with azaarene•HCl (0.400 mmol, 1.00 equiv.), Selectfluor (424 mg, 1.20 mmol, 3.00 equiv.), $Et_3N·HCl$ (54.8 mg, 0.400 mmol, 1.00 equiv.), and dry acetonitrile (2.0 mL, c = 0.2 M). The vial was then sealed with a rubber septum. Then the vial was placed onto an aluminum plate fitted with a 10 W high-power single LED plate (Avonec LED chip, size: 6.8 × 6.8 mm, λ_{max} = 280 nm), which is driven by a constant current power supply (22–36 V, 350 mA). Another plastic block was utilized to hold the quartz vial. The temperature was kept at approximately 30 °C through the use of a cooling fan. The reaction mixture was stirred and irradiated for 24 hours, then, 2 mL of saturated aqueous Na_2CO_3 were added. To obtain purified material, extraction followed by column chromatography on silica gel was conducted. Where necessary, further purification was accomplished by preparative HPLC.

Table S1. Reaction condition optimization

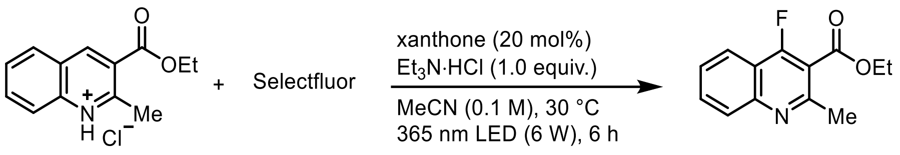

1·HCl **2**

entry	Change of reaction conditions	yield of product[b]
1	None	61%
2	No light	0%
3	No HCl pre-coordination	22%
4	No xanthone	30%
5	No $Et_3N \cdot HCl$	40%
6	With KF (2 equiv.)	56%
7	With KF (2 equiv.), no Selectfluor	0%
8	Selectfluor (4 equiv.), xanthone (40 mol%)	67%
9	Change $Et_3N \cdot HCl$ to Et_3N (1 equiv.)	30%
10	Change HCl to $Zn(OTf)_2$ (1 equiv.)	29%
11	Change HCl to HBF_4 pre-coordination	54%
12	Change HCl to HBF_4 pre-coordination, no No $Et_3N \cdot HCl$	5%
13	Change HCl to BF_3 pre-coordination	51%
14	Change HCl to BF_3 pre-coordination, no No $Et_3N \cdot HCl$	7%
15	Change Selectfluor to NFSI (3 equiv.)	17%
16	Change MeCN to HFIP	<5%
17	Change xanthone to $[Acr\text{-}Mes]ClO_4$ (2 mol%)[c]	5%
18	Change xanthone to $[Ru(bpy)_3](PF_6)_2$ (2 mol%)[c]	5%
19	Change xanthone to *fac*-$[Ir(ppy)_3]$ (2 mol%)[c]	37%
20	Change xanthone to $[Ir(dF(CF_3)ppy)_2(dtbbpy)](PF_6)$ (2 mol%)[c]	28%
21	Change xanthone to *fac*-$[Ir(dFppy)_3]$ (2 mol%)[c]	40%

[a] Ethyl 2-methylquinoline-3-carboxylate hydrochloride (0.100 mmol, 1.00 equiv.), Selectfluor (3.00 equiv.), xanthone (20 mol%), $Et_3N \cdot HCl$ (1.00 equiv.), MeCN (0.1 M), 365 nm LED (6 W), 30 °C, 6 h. [b] NMR yield with 2-fluorotoluene as internal standard. [c] 400 nm LED (10 W), 30 °C, 12 h.

Discussion:

^{1}H NMR analysis of the reaction mixture: The fluorination product was obtained in 61% yield. Chlorination of quinoline was not observed, and the major "byproduct" of the reaction is the unreacted starting material in 34%. The photosensitizer, xanthone, can be recovered after the reaction in >95% yield (determined by GC-FID and ^{1}H NMR), and it is compatible with Selectfluor at room temperature.

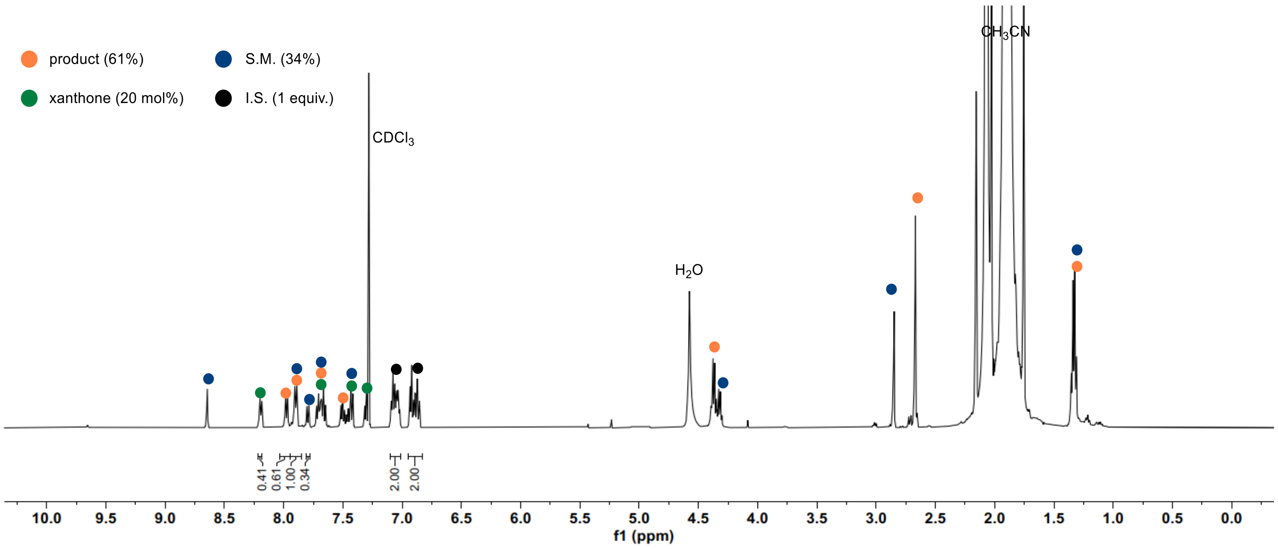

Figure S3. Crude ^{1}H NMR of the reaction mixture of **1•HCl**

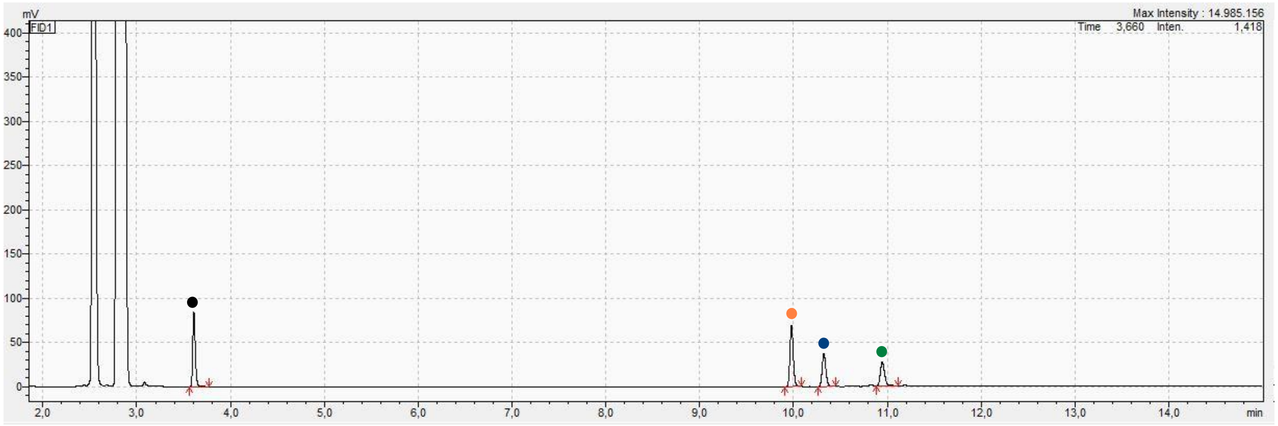

Figure S4. GC-FID of the reaction mixture of **1•HCl**

Product inhibition of the fluorination reaction: We have observed significant product inhibition for the azaarene fluorination reaction. When 50 mol% fluorinated quinoline derivative was subjected to the azaarene fluorination reaction, the yields decreased from 60% to 29%. Therefore, the accumulation of the fluorinated product could inhibit the ongoing reaction and cause incomplete reactions. The phenomenon can also explain why the yield of the reaction did not increase significantly when more Selectfluor was added (Table S1, entry 8).

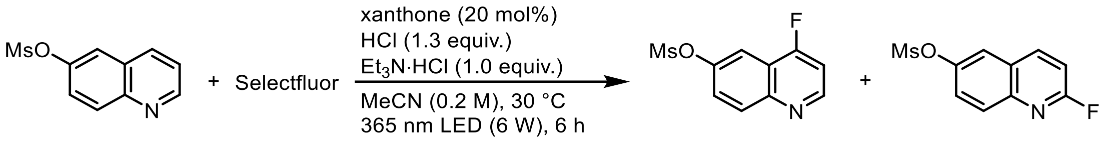

w/o additional **20b**:	**20a**, 35%	**20b**, 25%
w 50 mol% **20b**:	16%	13%

Background reaction: The background reaction without a photosensitizer is due to the direct excitation of protonated quinoline. It was reported that the triplet state of quinoline could be accessible via direct excitation by 365 nm light due to the quinoline chromophore[2]. After the formation of triplet state, the radical initiation and propagation are the same as that without a photosensitizer.

Difference in fluorinating reagents: The sulfonimidyl radical produced from NFSI exhibits substantial electrophilicity. As an illustration, the substrate scope applicable for radical aromatic substitution using Selectfluor and NFSI displays similarity[3,4]. Consequently, it is conceivable that the fluoride-coupled electron transfer mechanism might be operable with NFSI as well. However, the process may encounter significantly higher energy barriers, leading to a diminished yield.

Fluorination of azaarenes

Ethyl 4-fluoro-2-methylquinoline-3-carboxylate (2)

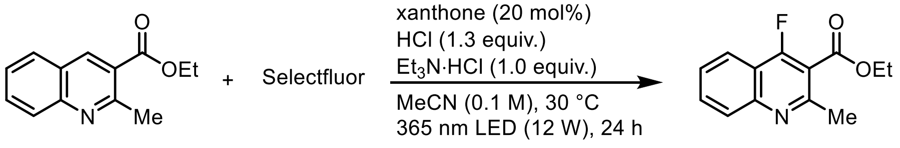

Under an ambient atmosphere, a 20-mL borosilicate vial equipped with a magnetic stir bar was charged with ethyl 2-methylquinoline-3-carboxylate (86.0 mg, 0.400 mmol, 1.00 equiv.), followed by dry DCM (4.0 mL, *c* = 0.1 M). Subsequently, 4.0 M HCl solution in dioxane (133 µL, 0.52 mmol, 1.3 equiv.) was added. The mixture was stirred at ambient temperature for 10 min. After that, the solvent was removed by a rotary evaporator. Under a nitrogen atmosphere, to the vial were then added xanthone (15.6 mg, 80 µmol, 20 mol%), Selectfluor (566 mg, 1.60 mmol, 4.00 equiv.), Et_3N·HCl (54.8 mg, 0.400 mmol, 1.00 equiv.), and dry acetonitrile (4.0 mL, *c* = 0.1 M). The vial was sealed with a septum cap. Then the vial was placed onto an aluminum plate fitted with a 12 W high-power single LED plate (EpiLED Chip, size: 10 × 10 mm, λ_{max} = 365 nm, 1.2 A, 9–11 V). Another aluminum block was utilized to hold the vial. The temperature was kept at approximately 30 °C through the use of a cooling fan. The reaction mixture was stirred and irradiated for 24 hours, then, 2 mL of saturated aqueous Na_2CO_3 were added. The mixture was then diluted with 5 mL of ethyl acetate, the layers were separated, and the aqueous layer was extracted twice with ethyl acetate (approx. 5 mL each time). Then the combined organic layer was dried over Na_2SO_4, and the solvent was removed *in vacuo*. The residue was purified by flash column chromatography on silica gel eluting with hexanes/ethyl acetate (20:1 + 1% Et_3N, v:v:v) to obtain a mixture. The mixture was further purified by normal phase HPLC (YMC PVA-Sil, 250 × 30.0 mm, 5 µm) with an eluent mixture of *i*-hexane/MTBE (85:15, v:v) at a flow rate of 42.5 mL/min to afford the title compound **2** (52.0 mg, 223 µmol, 56%) as a colorless oil.

R_f = 0.40 (pentane/EtOAc, 5:1, v/v (UV))

NMR Spectroscopy:

^{1}H NMR (500 MHz, $CDCl_3$, 25 °C, δ): 8.08 (dd, *J* = 8.4, 1.5 Hz, 1H), 8.06 – 8.00 (m, 1H), 7.80 (ddd, *J* = 8.5, 6.9, 1.4 Hz, 1H), 7.58 (ddd, *J* = 8.2, 6.9, 1.1 Hz, 1H), 4.50 (q, *J* = 7.1 Hz, 2H), 2.82 (s, 3H), 1.45 (t, *J* = 7.2 Hz, 3H).

^{13}C {^{1}H} NMR (126 MHz, $CDCl_3$, 25 °C, δ): 164.8, 163.4 (d, *J* = 273.6 Hz), 157.9 (d, *J* = 3.1 Hz), 149.6 (d, *J* = 6.0 Hz), 132.0, 128.5 (d, *J* = 3.6 Hz), 126.9, 121.2 (d, *J* = 5.4 Hz), 117.4 (d, *J* = 14.1 Hz), 113.0 (d, *J* = 9.6 Hz), 62.2, 24.3 (d, *J* = 3.4 Hz), 14.3.

^{19}F NMR (471 MHz, $CDCl_3$, 25 °C, δ): –112.7 (s).

HRMS GC-EI (m/z) calc'd for $C_{13}H_{12}NO_2F^+$ [M]$^+$, 233.0847; found, 233.0850. Deviation: –1.3 ppm.

3-Acetyl-4-fluoroquinoline (4a) and 3-acetyl-2-fluoroquinoline (4b)

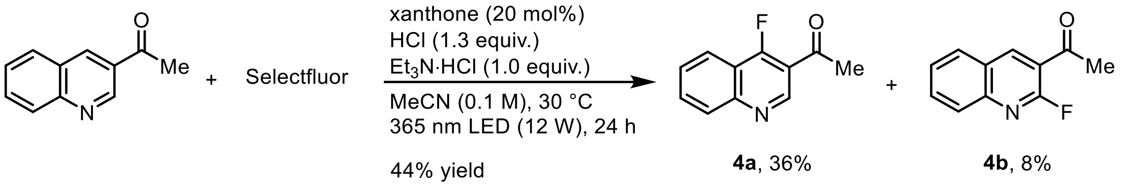

4a, 36% **4b**, 8%

Under an ambient atmosphere, a 20-mL borosilicate vial equipped with a magnetic stir bar was charged with 3-acetyl quinoline (68.4 mg, 0.400 mmol, 1.00 equiv.), followed by dry DCM (4.0 mL, *c* = 0.1 M). Subsequently, 4.0 M HCl solution in dioxane (133 µL, 0.52 mmol, 1.3 equiv.) was added. The mixture was stirred at ambient temperature for 10 min. After that, the solvent was removed by a rotary evaporator. Under a nitrogen atmosphere, to the vial were then added xanthone (15.6 mg, 80 µmol, 20 mol%), Selectfluor (566 mg, 1.60 mmol, 4.00 equiv.), $Et_3N \cdot HCl$ (54.8 mg, 0.400 mmol, 1.00 equiv.), and dry acetonitrile (4.0 mL, *c* = 0.1 M). The vial was sealed with a septum cap. Then the vial was placed onto an aluminum plate fitted with a 12 W high-power single LED plate (EpiLED Chip, size: 10 × 10 mm, λ_{max} = 365 nm, 1.2 A, 9–11 V). Another aluminum block was utilized to hold the vial. The temperature was kept at approximately 30 °C through the use of a cooling fan. The reaction mixture was stirred and irradiated for 24 hours, then, 2 mL of saturated aqueous Na_2CO_3 were added. The mixture was then diluted with 5 mL of ethyl acetate, the layers were separated, and the aqueous layer was extracted twice with ethyl acetate (approx. 5 mL each time). Then the combined organic layer was dried over Na_2SO_4, and the solvent was removed *in vacuo*. The residue was purified by flash column chromatography on silica gel eluting with hexanes/ethyl acetate (from 20:1 to 10:1 + 1% Et_3N, v:v:v) to afford 3-acetyl quinoline (30.8 mg, 180 µmol, 45%) and the title compounds **4a** (27.1 mg, 144 µmol, 36%) and **4b** (6.0 mg, 32 µmol, 8%) as colorless oils.

3-Acetyl-4-fluoroquinoline (**4a**)

R*f* = 0.15 (pentane/EtOAc, 5:1, v/v (UV))

NMR Spectroscopy:

^{1}H NMR (500 MHz, $CDCl_3$, 25 °C, δ): 9.27 (d, *J* = 10.3 Hz, 1H), 8.20 (dd, *J* = 8.2, 1.5 Hz, 1H), 8.13 (dt, *J* = 8.5, 1.1 Hz, 1H), 7.86 (ddd, *J* = 8.4, 6.9, 1.4 Hz, 1H), 7.66 (t, *J* = 7.8 Hz, 1H), 2.76 (d, *J* = 4.5 Hz, 3H).

^{13}C {^{1}H} NMR (126 MHz, $CDCl_3$, 25 °C, δ): 194.6 (d, *J* = 3.0 Hz), 165.8 (d, *J* = 278.0 Hz), 152.1 (d, *J* = 5.5 Hz), 151.5 (d, *J* = 3.0 Hz), 132.9, 129.5 (d, *J* = 3.4 Hz), 128.0, 122.0 (d, *J* = 7.0 Hz), 118.7 (d, *J* = 14.7 Hz), 115.5 (d, *J* = 7.4 Hz), 32.0 (d, *J* = 6.3 Hz).

^{19}F NMR (471 MHz, $CDCl_3$, 25 °C, δ): –108.6 (s).

HRMS GC-EI (m/z) calc'd for $C_{11}H_8NOF^+$ [M]$^+$, 189.0584; found, 189.0587. Deviation: –1.4 ppm.

3-Acetyl-2-fluoroquinoline (**4b**)

R*f* = 0.35 (EtOAc/pentane, 1:5, v/v (UV))

NMR Spectroscopy:

^{1}H NMR (500 MHz, $CDCl_3$, 25 °C, δ): 8.86 (d, *J* = 9.5 Hz, 1H), 7.98 (ddd, *J* = 8.0, 2.5, 1.2 Hz, 2H), 7.85 (ddd, *J* = 8.4, 6.9, 1.5 Hz, 1H), 7.61 (ddd, *J* = 8.2, 6.8, 1.1 Hz, 1H), 2.70 (d, *J* = 4.7 Hz, 3H).

^{13}C {^{1}H} NMR (151 MHz, $CDCl_3$, 25 °C, δ): 194.8 (d, *J* = 7.5 Hz), 158.4 (d, *J* = 244.9 Hz), 147.4 (d, *J* = 17.7 Hz), 144.4 (d, *J* = 5.2 Hz), 133.2, 129.4, 128.0 (d, *J* = 1.7 Hz), 127.2 (d, *J* = 2.4 Hz), 126.7 (d, *J* = 2.3 Hz), 120.4 (d, *J* = 34.4 Hz), 31.1 (d, *J* = 7.9 Hz).

^{19}F NMR (471 MHz, $CDCl_3$, 25 °C, δ): –60.3 (s).

HRMS GC-EI (m/z) calc'd for $C_{11}H_8NOF^+$ $[M]^+$, 189.0584; found, 189.0585. Deviation: –0.3 ppm.

4-Fluoro-2-methylquinoline (5)

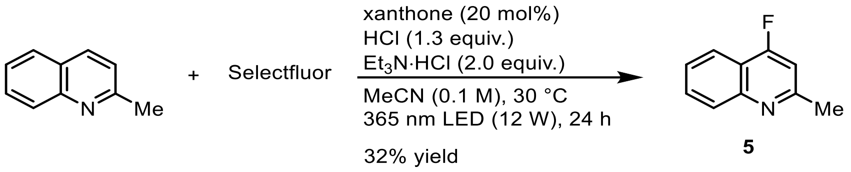

Under an ambient atmosphere, a 20-mL borosilicate vial equipped with a magnetic stir bar was charged with 2-methylquinoline (56 µL, 56 mg, 0.40 mmol, 1.0 equiv.), followed by dry DCM (4.0 mL, *c* = 0.1 M). Subsequently, 4.0 M HCl solution in dioxane (133 µL, 0.52 mmol, 1.3 equiv.) was added. The mixture was stirred at ambient temperature for 10 min. After that, the solvent was removed by a rotary evaporator. Under a nitrogen atmosphere, to the vial were then added xanthone (15.6 mg, 80 µmol, 20 mol%), Selectfluor (425 mg, 1.20 mmol, 3.00 equiv.), $Et_3N\cdot HCl$ (109.6 mg, 0.800 mmol, 2.00 equiv.), and dry acetonitrile (4.0 mL, *c* = 0.1 M). The vial was sealed with a septum cap. Then the vial was placed onto an aluminum plate fitted with a 12 W high-power single LED plate (EpiLED Chip, size: 10 × 10 mm, λ_{max} = 365 nm, 1.2 A, 9–11 V). Another aluminum block was utilized to hold the vial. The temperature was kept at approximately 30 °C through the use of a cooling fan. The reaction mixture was stirred and irradiated for 24 hours. After that, 2-fluorotoluene (44 µL, 0.40 mmol, 1.0 equiv.) was added as an internal standard, followed by 1 mL of saturated aqueous Na_2CO_3. An aliquot (ca. 0.1 mL) of the organic phase was taken and diluted with $CDCl_3$ (0.5 mL), and the yield of product (32%; **5**: δ 2.68 (s) ppm, Figure S5) and remained starting material (63%; 2-methylquinoline: δ 2.69 (s) ppm, Figure S5) were determined by ^{1}H NMR integration relative to the internal standard. To isolate the fluorinated product, an identical reaction without added standard was diluted with 2 mL of ethyl acetate, the layers were separated, and the aqueous layer was extracted twice with ethyl acetate (approx. 2 mL each time). Then the combined organic layer was dried over Na_2SO_4, and the solvent was removed *in vacuo*. The residue was purified by flash column chromatography on silica gel eluting with hexanes/ethyl acetate (50:1, v:v). To decrease the volatility of the product, the eluate was treated with two

drops of 4.0 M HCl solution in dioxane before concentration. The hydrochloride salt of the title compound **5·HCl** was obtained as a colorless solid.

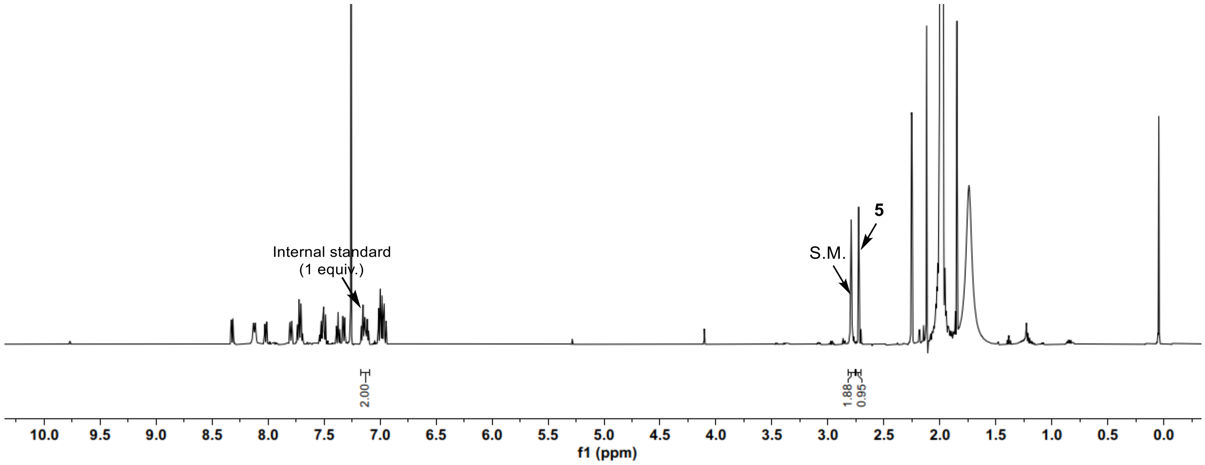

Figure S5. ^{1}H NMR of the crude mixture for product **5**

The following data were obtained from the reaction mixture:

R*f* = 0.45 (hexanes/EtOAc, 5:1, v/v (UV))

The following data were obtained from the pure product:

4-Fluoro-2-methylquinoline hydrogen chloride (**5·HCl**)

NMR Spectroscopy:

^{1}**H NMR** (500 MHz, CD_3CN, 25 °C, δ): 8.82 (d, *J* = 8.7 Hz, 1H), 8.31 (dd, *J* = 8.4, 1.4 Hz, 1H), 8.16 (ddd, *J* = 8.5, 7.0, 1.4 Hz, 1H), 7.94 (t, *J* = 7.7 Hz, 1H), 7.56 (d, *J* = 9.3 Hz, 1H), 3.09 (s, 3H).

^{13}**C {^{1}H} NMR** (151 MHz, CD_3CN, 25 °C, δ): 170.3 (d, *J* = 280.1 Hz), 162.9 (d, *J* = 14.9 Hz), 142.8 (d, *J* = 8.0 Hz), 136.6, 130.9 (d, *J* = 1.6 Hz), 123.0 (d, *J* = 4.3 Hz), 122.2, 119.8 (d, *J* = 16.9 Hz), 110.4 (d, *J* = 20.6 Hz), 21.5 (d, *J* = 1.9 Hz).

^{19}**F NMR** (471 MHz, CD_3CN, 25 °C, δ): –96.2 (brs).

HRMS ESIpos (m/z) calc'd for $C_{10}H_9NF^+$ $[M+H]^+$, 162.0714; found, 162.0713. Deviation: +0.3 ppm.

4-Fluorobenzo[*h*]quinoline (6)

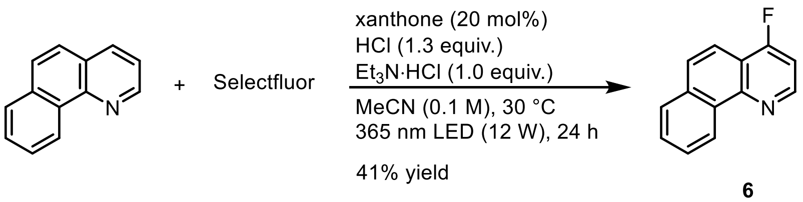

6

Under an ambient atmosphere, a 20-mL borosilicate vial equipped with a magnetic stir bar was charged with benzo[*h*]quinoline (71.6 mg, 0.400 mmol, 1.00 equiv.), followed by dry DCM (4.0 mL, *c* = 0.1 M).

Subsequently, 4.0 M HCl solution in dioxane (133 μL, 0.52 mmol, 1.3 equiv.) was added. The mixture was stirred at ambient temperature for 10 min. After that, the solvent was removed by a rotary evaporator. Under a nitrogen atmosphere, to the vial were then added xanthone (15.6 mg, 80 μmol, 20 mol%), Selectfluor (425 mg, 1.20 mmol, 3.00 equiv.), $Et_3N{\cdot}HCl$ (54.8 mg, 0.400 mmol, 1.00 equiv.), and dry acetonitrile (4.0 mL, *c* = 0.1 M). The vial was sealed with a septum cap. Then the vial was placed onto an aluminum plate fitted with a 12 W high-power single LED plate (EpiLED Chip, size: 10 × 10 mm, λ_{max} = 365 nm, 1.2 A, 9–11 V). Another aluminum block was utilized to hold the vial. The temperature was kept at approximately 30 °C through the use of a cooling fan. The reaction mixture was stirred and irradiated for 24 hours, then, 2 mL of saturated aqueous Na_2CO_3 were added. The mixture was then diluted with 5 mL of ethyl acetate, the layers were separated, and the aqueous layer was extracted twice with ethyl acetate (approx. 5 mL each time). Then the combined organic layers were dried over Na_2SO_4, and the solvent was removed *in vacuo*. The residue was purified by flash column chromatography on silica gel eluting with hexanes/ethyl acetate (100:1 + 1% Et_3N, v:v:v) to afford the title compounds **6** (32.3 mg, 41%) as a colorless solid. Further purification by normal phase HPLC (YMC PVA-Sil, 250 × 30.0 mm, 5 μm) with an eluent mixture of *i*-hexane/2-propanol (99.5:0.5, v:v) at a flow rate of 42.5 mL/min to provide the title compound **6** as a colorless solid.

R_f = 0.60 (pentane/EtOAc, 10:1, v/v (UV))

NMR Spectroscopy:

^{1}H NMR (500 MHz, $CDCl_3$, 25 °C, δ): 9.27 (dd, *J* = 7.7, 1.7 Hz, 1H), 8.94 (dd, *J* = 8.4, 5.1 Hz, 1H), 7.98 (d, *J* = 9.0 Hz, 1H), 7.94 (dd, *J* = 8.0, 1.5 Hz, 1H), 7.88 (d, *J* = 9.0 Hz, 1H), 7.79 – 7.70 (m, 2H), 7.24 (dd, *J* = 9.0, 5.1 Hz, 1H).

^{13}C {^{1}H} NMR (126 MHz, $CDCl_3$, 25 °C, δ): 165.5 (d, *J* = 266.3 Hz), 150.1 (d, *J* = 7.9 Hz), 149.2 (d, *J* = 4.7 Hz), 133.9, 131.0, 128.9, 128.3 (d, *J* = 1.8 Hz), 128.2, 127.7, 124.8, 117.3 (d, *J* = 6.0 Hz), 117.0 (d, *J* = 12.4 Hz), 107.4 (d, *J* = 15.3 Hz).

^{19}F NMR (471 MHz, $CDCl_3$, 25 °C, δ): –112.6 (brs).

HRMS ESIpos (m/z) calc'd for $C_{13}H_9NF^+$ $[M+H]^+$, 198.0713; found, 198.0715. Deviation: –0.6 ppm.

Chloroalkyl 4-fluoro-2-methylquinoline-3-carboxylate 7

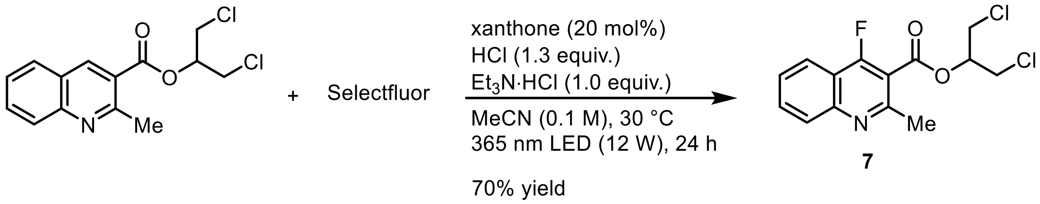

Under an ambient atmosphere, a 20-mL borosilicate vial equipped with a magnetic stir bar was charged with 1,3-dichloropropan-2-yl 2-methylquinoline-3-carboxylate (119.2 mg, 0.400 mmol, 1.00 equiv.), followed by dry DCM (4.0 mL, *c* = 0.1 M). Subsequently, 4.0 M HCl solution in dioxane (133 μL, 0.52 mmol, 1.3 equiv.) was added. The mixture was stirred at ambient temperature for 10 min. After that, the solvent was removed by a

rotary evaporator. Under a nitrogen atmosphere, to the vial were then added xanthone (15.6 mg, 80 µmol, 20 mol%), Selectfluor (425 mg, 1.20 mmol, 3.00 equiv.), $Et_3N·HCl$ (54.8 mg, 0.400 mmol, 1.00 equiv.), and dry acetonitrile (4.0 mL, *c* = 0.1 M). The vial was sealed with a septum cap. Then the vial was placed onto an aluminum plate fitted with a 12 W high-power single LED plate (EpiLED Chip, size: 10 × 10 mm, λ_{max} = 365 nm, 1.2 A, 9–11 V). Another aluminum block was utilized to hold the vial. The temperature was kept at approximately 30 °C through the use of a cooling fan. The reaction mixture was stirred and irradiated for 24 hours, then 2 mL of saturated aqueous Na_2CO_3 were added. The mixture was then diluted with 5 mL of ethyl acetate, the layers were separated, and the aqueous layer was extracted twice with ethyl acetate (approx. 5 mL each time). Then the combined organic layers were dried over Na_2SO_4, and the solvent was removed *in vacuo*. The residue was purified by flash column chromatography on silica gel eluting with hexanes/ethyl acetate (20:1 + 1% Et_3N, v:v:v) to obtain a mixture. The mixture was further purified by normal phase HPLC (YMC PVA-Sil, 250 × 30.0 mm, 5 µm) with an eluent mixture of *i*-hexane/2-propanol (98:2, v:v) at a flow rate of 42.5 mL/min to provide the title compound **7** (88.2 mg, 279 µmol, 70%) as a colorless solid.

R_f = 0.40 (pentane/EtOAc, 5:1, v/v (UV))

NMR Spectroscopy:

^{1}H NMR (500 MHz, $CDCl_3$, 25 °C, δ): 8.12 – 8.08 (m, 1H), 8.05 (dd, *J* = 8.6, 1.8 Hz, 1H), 7.83 (ddd, *J* = 8.5, 7.0, 1.5 Hz, 1H), 7.61 (ddd, *J* = 8.1, 6.9, 1.1 Hz, 1H), 5.56 (p, *J* = 5.2 Hz, 1H), 3.92 (dd, *J* = 8.0, 5.2 Hz, 4H), 2.85 (s, 3H).

^{13}C {^{1}H} NMR (126 MHz, $CDCl_3$, 25 °C, δ): 163.9 (d, *J* = 274.9 Hz), 163.7, 157.8 (d, *J* = 2.6 Hz), 149.8 (d, *J* = 6.2 Hz), 132.4, 128.5 (d, *J* = 3.6 Hz), 127.1, 121.3 (d, *J* = 5.6 Hz), 117.3 (d, *J* = 13.9 Hz), 111.6 (d, *J* = 8.8 Hz), 73.6, 43.4, 24.5 (d, *J* = 3.2 Hz).

^{19}F NMR (471 MHz, $CDCl_3$, 25 °C, δ): –110.7 (s).

HRMS GC-EI (m/z) calc'd for $C_{14}H_{12}NO_2FCl_2^+$ $[M]^+$, 315.0224; found, 315.0229. Deviation: –1.8 ppm.

4-Fluoroquinoline (3a) and 2-fluoroquinoline (3b)

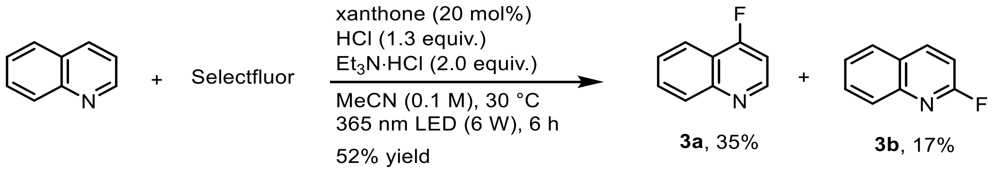

3a, 35% **3b**, 17%

entry	Change of reaction conditions	yield of products[b]	C4:C2
1	None	52%	2:1
2	Change HCl to $BF_3·Et_2O$ (1.3 equiv.)	41%	2:1
3	Change HCl to $Zn(OTf)_2$ (1.3 equiv.)	11%	>10:1
4	Change HCl to $Zn(BF_4)_2$ (1.3 equiv.)	13%	>10:1
5	Change HCl to $Y(OTf)_2$ (1.3 equiv.)	26%	6:1

6	Change HCl to $Ga(OTf)_3$ (1.3 equiv.)	6%	--
7	Change HCl to $Bi(OTf)_3$ (1.3 equiv.)	<5%	--
8	Change HCl to TMSOTf (1.3 equiv.)	<5%	--
9	With 1.0 equiv. of 2-fluoroanisole	40%[c]	1.5:1

[a] Quinoline (0.100 mmol, 1.00 equiv.), HCl (1.3 equiv.); then Selectfluor (3.00 equiv.), xanthone (20 mol%), $Et_3N{\cdot}HCl$ (2.00 equiv.), MeCN (0.1 M), 365 nm LED (6 W), 30 °C, 6 h. [b] NMR yield with 2-fluorotoluene as internal standard. [c] TEDAylation product of 2-fluoroanisole was observed in <1% yield.

Discussion:

Site-selectivity: Owing to the increased steric hindrance on C2 by changing HCl to Lewis acids, higher C4 selectivity could be observed.

The TEDAylation reaction: We did not observe an efficient TEDAylation reaction[4] of 2-fluoroanisole (<1% yield) when 2-fluoroanisole was employed as an additive to the quinoline fluorination reaction (entry 9). We believe the protonated quinoline, Cl^-, $TEDA^{2+\bullet}$, and F^- could form an ion pair and hydrogen bonding network, which leads to the success of the fluorination reaction. However, arenes lack the opportunity of ion pairing and hydrogen bonding interactions with $TEDA^{2+\bullet}$. The lack of the previously-observed reactivity could be another hint as to the concertedness of the reaction.

Under an ambient atmosphere, a 4-mL borosilicate vial equipped with a magnetic stir bar was charged with quinoline (13 µL, 13 mg, 0.10 mmol, 1.0 equiv.), followed by dry DCM (1.0 mL, c = 0.1 M). Subsequently, 4.0 M HCl solution in dioxane (33 µL, 0.13 mmol, 1.3 equiv.) was added. The mixture was stirred at ambient temperature for 10 min. After that, the solvent was removed by a rotary evaporator. Under a nitrogen atmosphere, to the vial were then added xanthone (3.9 mg, 20 µmol, 20 mol%), Selectfluor (106.2 mg, 0.300 mmol, 3.00 equiv.), $Et_3N{\cdot}HCl$ (27.4 mg, 0.200 mmol, 2.00 equiv.), and dry acetonitrile (1.0 mL, c = 0.1 M). The vial was sealed with a septum cap. Then the vial was placed onto an aluminum plate fitted with a 6 W high-power single LED plate (EpiLED Chip, size: 10 × 10 mm, λ_{max} = 365 nm, 600 mA, 9–11 V). Another aluminum block was utilized to hold the vial. The temperature was kept at approximately 30 °C through the use of a cooling fan. The reaction mixture was stirred and irradiated for 6 hours. After that, 2-fluorotoluene (11 µL, 0.10 mmol, 1.0 equiv.) was added as an internal standard, followed by 1 mL of saturated aqueous Na_2CO_3. An aliquot (0.1 mL) of the organic phase was taken and diluted with $CDCl_3$ (0.5 mL), and the yield was determined by ^{19}F NMR integration relative to the internal standard (52% yield, **3a**:**3b** = 2:1; standard: δ –118.6 (m) ppm, **3a**: δ –113.2 (t, J = 9.1 Hz) ppm, and **3b**: δ –62.7 (d, J = 8.7 Hz) ppm, Figure S6). To isolate the fluorinated product, another reaction with 2 mol% [$Ir(dFppy)_3$] instead of xanthone as photosensitizer was carried out (42%, **3a**:**3b** = 1.5:1), because it is difficult to separate **3b** and xanthone by flash column chromotography. The reaction mixture was transferred to a separatory funnel. The mixture was then diluted with 2 mL of ethyl acetate, the layers were separated, and the aqueous layer was extracted twice with ethyl acetate (approx. 2 mL each time). Then the combined organic layers were dried over Na_2SO_4, and the solvent was removed *in vacuo*. The residue was purified by flash column chromatography

on silica gel eluting with hexanes/ethyl acetate (100:1 to 50:1, v:v). To decrease the volatility of the product, the eluate was treated with two drops of 4.0 M HCl solution in dioxane before concentration. The hydrochloride salt of the title compound **3a·HCl** was obtained as a colorless solid and **3b** as colorless oil (protonation of **3b** by HCl or HTFA was not observed).

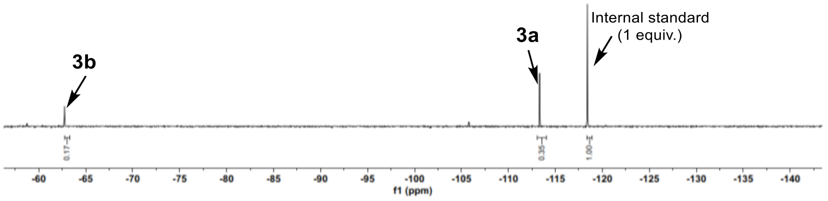

Figure S6. ^{19}F NMR of the reaction mixture for products **3a** and **3b**

The following data were obtained from the reaction mixture:

R*f* = 0.40 (hexanes/EtOAc, 5:1, v/v (UV)) (**3a**)

R*f* = 0.60 (hexanes/EtOAc, 5:1, v/v (UV)) (**3b**)

The following data were obtained from the pure product:

4-Fluoroquinoline hydrochloride salt (**3a·HCl**):

NMR Spectroscopy:

^{1}H NMR (500 MHz, CD_3CN, 25 °C, δ): 9.06 (t, *J* = 6.0 Hz, 1H), 8.78 (ddd, *J* = 8.7, 1.8, 0.9 Hz, 1H), 8.39 (ddd, *J* = 8.3, 1.3, 0.7 Hz, 1H), 8.21 (ddd, *J* = 8.6, 7.0, 1.3 Hz, 1H), 8.01 (ddd, *J* = 8.2, 6.9, 1.0 Hz, 1H), 7.73 (dd, *J* = 8.6, 6.2 Hz, 1H).

^{13}C {^{1}H} NMR (151 MHz, CD_3CN, 25 °C, δ): 171.2 (d, *J* = 281.7 Hz), 148.4 (d, *J* = 14.1 Hz), 143.1, 136.9, 131.7 (d, *J* = 1.7 Hz), 123.1 (d, *J* = 4.4 Hz), 122.7 (d, *J* = 2.1 Hz), 121.3 (d, *J* = 16.2 Hz), 109.2 (d, *J* = 20.8 Hz).

^{19}F NMR (471 MHz, CD_3CN, 25 °C, δ): –93.8 (brs).

HRMS ESIpos (m/z) calc'd for $C_9H_7NF^+$ $[M+H]^+$, 148.0557; found, 148.0555. Deviation: +1.2 ppm.

2-Fluoroquinoline (**3b**):

NMR Spectroscopy:

^{1}H NMR (500 MHz, CD_3CN, 25 °C, δ): 8.45 (t, *J* = 8.6 Hz, 1H), 8.00 (dd, *J* = 8.0, 1.5 Hz, 1H), 7.94 – 7.91 (m, 1H), 7.81 (ddd, *J* = 8.4, 6.8, 1.5 Hz, 1H), 7.63 (ddd, *J* = 8.1, 6.8, 1.2 Hz, 1H), 7.21 (dd, *J* = 8.8, 2.7 Hz, 1H).

^{13}C {^{1}H} NMR (151 MHz, CD_3CN, 25 °C, δ): 162.7 (d, *J* = 239.3 Hz), 147.1 (d, *J* = 17.4 Hz), 144.1 (d, *J* = 10.2 Hz), 132.2, 129.4, 129.2 (d, *J* = 1.5 Hz), 128.5 (d, *J* = 1.9 Hz), 127.7 (d, *J* = 2.7 Hz), 111.5 (d, *J* = 43.4 Hz).

^{19}F NMR (471 MHz, CD_3CN, 25 °C, δ): –62.7 (d, *J* = 8.7 Hz).

HRMS ESIpos (m/z) calc'd for $C_9H_7NF^+$ $[M+H]^+$, 148.0557; found, 148.0555. Deviation: +1.2 ppm.

Methyl 4-fluoroquinoline-6-carboxylate (8a) and methyl 2-fluoroquinoline-6-carboxylate (8b)

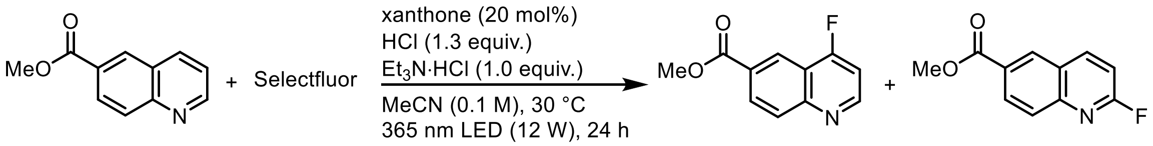

8a, 27% **8b**, 25%

Under an ambient atmosphere, a 20-mL borosilicate vial equipped with a magnetic stir bar was charged with methyl quinoline-6-carboxylate (74.8 mg, 0.400 mmol, 1.00 equiv.), followed by dry DCM (4.0 mL, *c* = 0.1 M). Subsequently, 4.0 M HCl solution in dioxane (133 µL, 0.52 mmol, 1.3 equiv.) was added. The mixture was stirred at ambient temperature for 10 min. After that, the solvent was removed by a rotary evaporator. Under a nitrogen atmosphere, to the vial were then added xanthone (15.6 mg, 80 µmol, 20 mol%), Selectfluor (566 mg, 1.60 mmol, 4.00 equiv.), $Et_3N{\cdot}HCl$ (54.8 mg, 0.400 mmol, 1.00 equiv.), and dry acetonitrile (4.0 mL, c = 0.1 M). The vial was sealed with a septum cap. Then the vial was placed onto an aluminum plate fitted with a 12 W high-power single LED plate (EpiLED Chip, size: 10 × 10 mm, λ_{max} = 365 nm, 1.2 A, 9–11 V). Another aluminum block was utilized to hold the vial. The temperature was kept at approximately 30 °C through the use of a cooling fan. The reaction mixture was stirred and irradiated for 24 hours, then, 2 mL of saturated aqueous Na_2CO_3 were added. The mixture was then diluted with 5 mL of ethyl acetate, the layers were separated, and the aqueous layer was extracted twice with ethyl acetate (approx. 5 mL each time). Then the combined organic layer was dried over Na_2SO_4, and the solvent was removed *in vacuo*. The residue was purified by flash column chromatography on silica gel eluting with hexanes/ethyl acetate (20:1 + 1% Et_3N, v:v:v) to afford methyl quinoline-6-carboxylate (26.9 mg, 144 µmol, 36%) and the title compounds **8a** (22.0 mg, 107 µmol, 27%) and **8b** (20.0 mg, 98 µmol, 25%) as colorless solids.

Methyl 4-fluoroquinoline-6-carboxylate (**8a**)

R_f = 0.20 (pentane/EtOAc, 1:5, v/v (UV))

NMR Spectroscopy:

^{1}H NMR (500 MHz, $CDCl_3$, 25 °C, δ): 8.90 (dd, *J* = 8.2, 5.1 Hz, 1H), 8.81 (d, *J* = 1.9 Hz, 1H), 8.31 (dd, *J* = 8.9, 2.0 Hz, 1H), 8.14 (dd, *J* = 8.9, 1.8 Hz, 1H), 7.12 (dd, *J* = 9.3, 5.1 Hz, 1H), 3.95 (3H).

^{13}C {^{1}H} NMR (126 MHz, $CDCl_3$, 25 °C, δ): 166.4, 166.0 (d, *J* = 270.0 Hz), 153.9 (d, *J* = 8.3 Hz), 152.2 (d, *J* = 3.5 Hz), 130.3, 129.8 (d, *J* = 3.7 Hz), 128.7 (d, *J* = 1.3 Hz), 124.0 (d, *J* = 5.2 Hz), 119.1 (d, *J* = 13.0 Hz), 106.6 (d, *J* = 14.3 Hz), 52.7.

^{19}F NMR (471 MHz, $CDCl_3$, 25 °C, δ): –111.2 (brs).

HRMS GC-EI (m/z) calc'd for $C_{11}H_8NO_2F^+$ $[M]^+$, 205.0534; found, 205.0537. Deviation: –1.8 ppm.

Methyl 2-fluoroquinoline-6-carboxylate (**8b**)

R*f* = 0.37 (pentane/EtOAc, 5:1, v/v (UV))

NMR Spectroscopy:

^{1}H NMR (500 MHz, $CDCl_3$, 25 °C, δ): 8.60 (d, *J* = 1.9 Hz, 1H), 8.40 – 8.29 (m, 2H), 7.99 (d, *J* = 8.8 Hz, 1H), 7.16 (dd, *J* = 8.8, 2.7 Hz, 1H), 4.00 (s, 3H)

^{13}C {^{1}H} NMR (151 MHz, $CDCl_3$, 25 °C, δ): 166.5, 162.6 (d, *J* = 245.7 Hz), 148.3 (d, *J* = 17.4 Hz), 143.3 (d, *J* = 10.4 Hz), 130.6, 130.5, 128.6 (d, *J* = 1.6 Hz), 128.1 (d, *J* = 2.5 Hz), 126.2 (d, *J* = 1.9 Hz), 111.3 (d, *J* = 42.3 Hz), 52.7.

^{19}F NMR (471 MHz, $CDCl_3$, 25 °C, δ): –58.1 (d, *J* = 8.2 Hz).

HRMS GC-EI (m/z) calc'd for $C_{11}H_8NO_2F^+$ $[M]^+$, 205.0534; found, 205.0536. Deviation: –1.3 ppm.

4,6-Difluoro-2-methylquinoline (9)

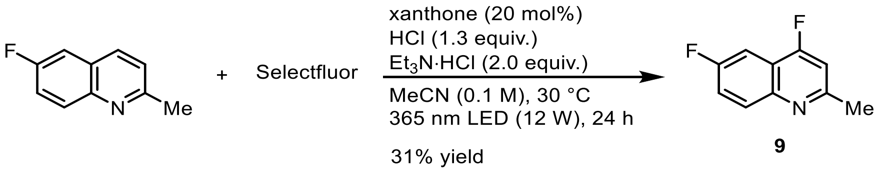

Under an ambient atmosphere, a 20-mL borosilicate vial equipped with a magnetic stir bar was charged with 6-fluoroquinoline (64 µL, 64 mg, 0.40 mmol, 1. 0 equiv.), followed by dry DCM (4.0 mL, *c* = 0.1 M). Subsequently, 4.0 M HCl solution in dioxane (133 µL, 0.52 mmol, 1.3 equiv.) was added. The mixture was stirred at ambient temperature for 10 min. After that, the solvent was removed by a rotary evaporator. Under a nitrogen atmosphere, to the vial were then added xanthone (15.6 mg, 80 µmol, 20 mol%), Selectfluor (425 mg, 1.20 mmol, 3.00 equiv.), $Et_3N·HCl$ (109.6 mg, 0.800 mmol, 2.00 equiv.), and dry acetonitrile (4.0 mL, *c* = 0.1 M). The vial was sealed with a septum cap. Then the vial was placed onto an aluminum plate fitted with a 12 W high-power single LED plate (EpiLED Chip, size: 10 × 10 mm, λ_{max} = 365 nm, 1.2 A, 9–11 V). Another aluminum block was utilized to hold the vial. The temperature was kept at approximately 30 °C through the use of a cooling fan. The reaction mixture was stirred and irradiated for 24 hours. After that, 2-fluorotoluene (44 µL, 0.40 mmol, 1.0 equiv.) was added as an internal standard, followed by 1 mL of saturated aqueous Na_2CO_3. An aliquot (ca. 0.1 mL) of the organic phase was taken and diluted with $CDCl_3$ (0.5 mL), and the yield of product (31%; **9**: δ 2.65 (s), ppm, Figure S7) and remained starting material (62%; **9**: δ 2.66 (s), ppm, Figure S7) were determined by ^{1}H NMR integration relative to the internal standard. To isolate the fluorinated product, an identical reaction without added standard was transferred to a separatory funnel. The mixture was then diluted with 2 mL of ethyl acetate, the layers were separated, and the aqueous layer was extracted twice with ethyl acetate (approx. 2 mL each time). Then the combined organic layer was dried over Na_2SO_4, and the solvent was removed *in vacuo*. The residue was purified by flash column chromatography on silica gel eluting with hexanes/ethyl acetate (20:1 + 1% Et_3N, v:v:v) to obtain a mixture.

The mixture was further purified by normal phase HPLC (YMC PVA-Sil, 250 × 30.0 mm, 5 µm) with an eluent mixture of *i*-hexane/2-propanol (90:10, v:v) at a flow rate of 42.5 mL/min. To decrease the volatility of the product, the eluate was treated with two drops of 4.0 M HCl solution in dioxane before concentration. The hydrochloride salt of the title compound **9·HCl** was obtained as a colorless solid.

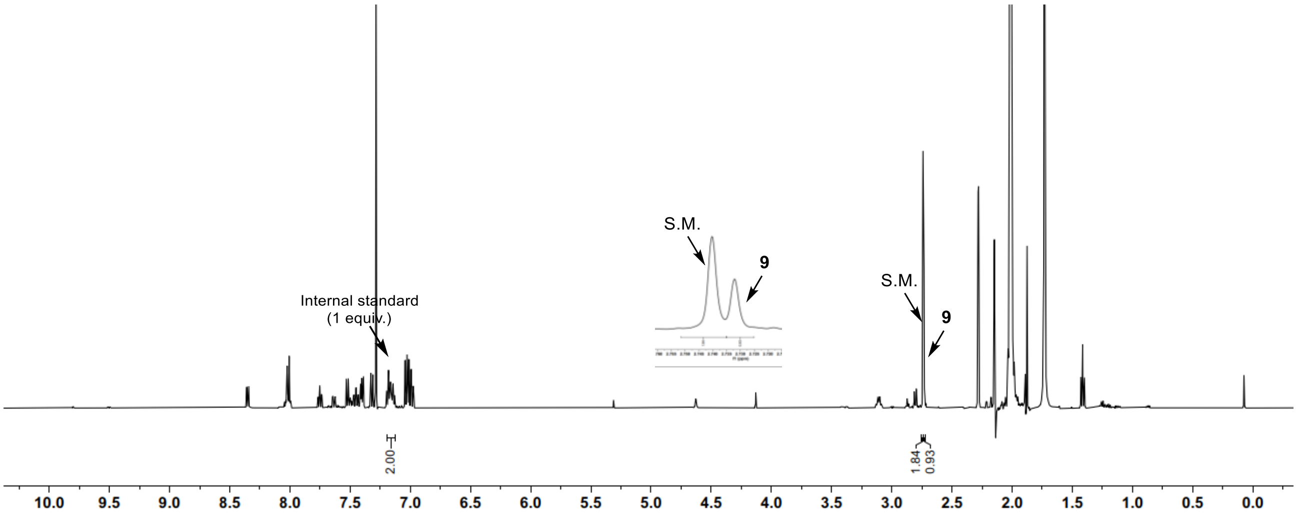

Figure S7. ^{1}H NMR of the reaction mixture for product **9**

The following data were obtained from the reaction mixture:

R*f* = 0.45 (hexanes/EtOAc, 5:1, v/v (UV))

The following data were obtained from the pure product:

4,6-Difluoro-2-methylquinoline hydrogen chloride (**9·HCl**)

NMR Spectroscopy:

^{1}H NMR (500 MHz, CD_3CN, 25 °C, δ): 8.95 (ddd, *J* = 9.4, 4.6, 1.7 Hz, 1H), 8.01 – 7.91 (m, 2H), 7.61 (d, *J* = 9.3 Hz, 1H), 3.09 (s, 3H).

^{13}C {^{1}H} NMR (126 MHz, CD_3CN, 25 °C, δ): 169.6 (d, *J* = 277.5 Hz), 163.0 (d, *J* = 252.2 Hz), 162.5 (d, *J* = 13.5 Hz), 139.9, 126.6 (d, *J* = 28.3 Hz), 125.4, 121.2 (dd, *J* = 17.3, 11.0 Hz), 110.2 (d, *J* = 19.8 Hz), 107.5 (dd, *J* = 25.1, 4.2 Hz), 21.3 (d, *J* = 1.8 Hz).

^{19}F NMR (471 MHz, $CDCl_3$, 25 °C, δ): –96.7 (brs, 1F), –109.5 (m, 1F).

HRMS ESIpos (m/z) calc'd for $C_{10}H_8NF_2^+$ [M+H]$^+$, 180.0619; found, 180.0620. Deviation: –0.3 ppm.

Polyfluoroalkyl 4-fluoro-2-methylquinoline-6-carboxylate 10

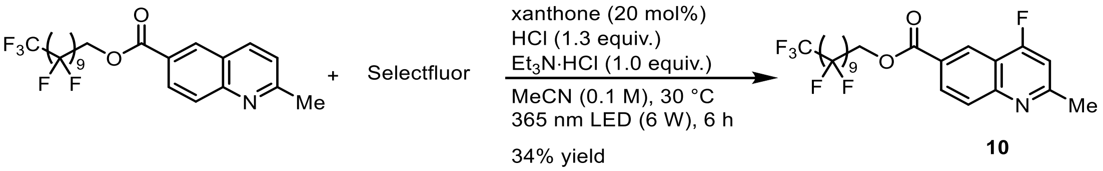

Under an ambient atmosphere, a 4-mL borosilicate vial equipped with a magnetic stir bar was charged with polyfluoroalkyl 2-methylquinoline-6-carboxylate (71.9 mg, 0.100 mmol, 1.00 equiv.), followed by dry DCM (1.0 mL, *c* = 0.1 M). Subsequently, 4.0 M HCl solution in dioxane (33 µL, 0.13 mmol, 1.3 equiv.) was added. The mixture was stirred at ambient temperature for 10 min. After that, the solvent was removed by a rotary evaporator. Under a nitrogen atmosphere, to the vial were then added xanthone (3.9 mg, 20 µmol, 20 mol%), Selectfluor (106.2 mg, 0.300 mmol, 3.00 equiv.), $Et_3N·HCl$ (13.7 mg, 0.100 mmol, 1.00 equiv.), and dry acetonitrile (1.0 mL, *c* = 0.1 M). The vial was sealed with a septum cap. Then the vial was placed onto an aluminum plate fitted with a 6 W high-power single LED plate (EpiLED Chip, size: 10 × 10 mm, λ_{max} = 365 nm, 600 mA, 9–11 V). Another aluminum block was utilized to hold the vial. The temperature was kept at approximately 30 °C through the use of a cooling fan. The reaction mixture was stirred and irradiated for 6 hours, then 1 mL of saturated aqueous Na_2CO_3 were added. The mixture was then diluted with 2 mL of ethyl acetate, the layers were separated, and the aqueous layer was extracted twice with ethyl acetate (approx. 2 mL each time). Then the combined organic layer was dried over Na_2SO_4, and the solvent was removed *in vacuo*. The residue was purified by flash column chromatography on silica gel eluting with hexanes/ethyl acetate (10:1 + 1% Et_3N, v:v:v) to obtain a mixture. The mixture was further purified by normal phase HPLC (YMC PVA-Sil, 250 × 30.0 mm, 5 µm) with an eluent mixture of *i*-hexane/2-propanol (95:5, v:v) at a flow rate of 42.5 mL/min to provide the title compound **10** (24.9 mg, 34 µmol, 34%) as a colorless solid.

R_f = 0.30 (pentane/EtOAc, 5:1, v/v (UV))

NMR Spectroscopy:

^{1}H NMR (500 MHz, $CDCl_3$, 25 °C, δ): 8.83 (dd, *J* = 2.0, 0.6 Hz, 1H), 8.32 (dd, *J* = 8.9, 1.9 Hz, 1H), 8.11 (d, *J* = 9.4 Hz, 1H), 7.09 (d, *J* = 10.1 Hz, 1H), 4.90 (t, *J* = 13.2 Hz, 1H), 2.79 (s, 3H).

^{13}C {^{1}H} NMR (151 MHz, $CDCl_3$, 25 °C, δ): 166.08 (d, *J* = 271.2 Hz), 164.4, 164.3, 151.9, 130.2, 129.3, 125.9, 124.8 (d, *J* = 5.0 Hz), 117.6 (d, *J* = 13.7 Hz), 107.4 (d, *J* = 14.1 Hz), 118.7 – 107.1 (m, 10C), 60.5 (t, *J* = 27.4 Hz), 26.1 (d, *J* = 2.9 Hz).

^{19}F NMR (471 MHz, $CDCl_3$, 25 °C, δ): –80.7 (t, *J* = 9.9 Hz, 3F), –113.2 (m, 1F), –119.2 (m, 2F), –121.7 (m, 6F), –121.8 (m, 4F), –122.6 (m, 2F), –123.1 (m, 2F), 126.1 (m, 2F).

HRMS ESIpos (m/z) calc'd for $C_{22}H_{10}NF_{22}O_2^+$ $[M+H]^+$, 738.0355; found, 738.0353. Deviation: +0.3 ppm.

4-Fluoro-2-phenylquinoline (11)

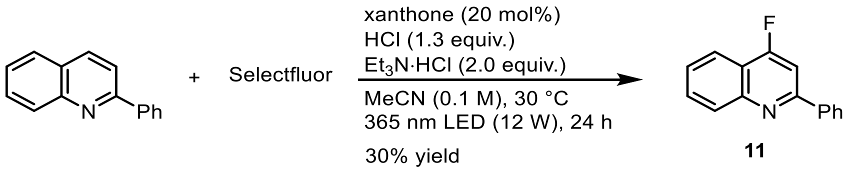

Under an ambient atmosphere, a 20-mL borosilicate vial equipped with a magnetic stir bar was charged with 2-phenylquinoline (82.0 mg, 0.400 mmol, 1.00 equiv.), followed by dry DCM (4.0 mL, *c* = 0.1 M).

Subsequently, 4.0 M HCl solution in dioxane (133 µL, 0.52 mmol, 1.3 equiv.) was added. The mixture was stirred at ambient temperature for 10 min. After that, the solvent was removed by a rotary evaporator. Under a nitrogen atmosphere, to the vial were then added xanthone (15.6 mg, 80 µmol, 20 mol%), Selectfluor (425 mg, 1.20 mmol, 3.00 equiv.), $Et_3N{\cdot}HCl$ (109.6 mg, 0.800 mmol, 2.00 equiv.), and dry acetonitrile (4.0 mL, *c* = 0.1 M). The vial was sealed with a septum cap. Then the vial was placed onto an aluminum plate fitted with a 12 W high-power single LED plate (EpiLED Chip, size: 10 × 10 mm, λ_{max} = 365 nm, 1.2 A, 9–11 V). Another aluminum block was utilized to hold the vial. The temperature was kept at approximately 30 °C through the use of a cooling fan. The reaction mixture was stirred and irradiated for 24 hours. After that, 2-fluorotoluene (44 µL, 0.40 mmol, 1.0 equiv.) was added as an internal standard, followed by 1 mL of saturated aqueous Na_2CO_3. An aliquot (ca. 0.1 mL) of the organic phase was taken and diluted with $CDCl_3$ (0.5 mL), and the yield was determined by ^{19}F NMR integration relative to the internal standard (30% yield; standard: δ –118.6 (m) ppm, **11**: δ –113.4 (d, *J* = 11.3 Hz) ppm, Figure S8). To isolate the fluorinated product, an identical reaction without added standard was transferred to a separatory funnel. The mixture was then diluted with 2 mL of ethyl acetate, the layers were separated, and the aqueous layer was extracted twice with ethyl acetate (approx. 2 mL each time). Then the combined organic layer was dried over Na_2SO_4, and the solvent was removed *in vacuo*. The residue was purified by flash column chromatography on silica gel eluting with hexanes/ethyl acetate (50:1 + 1% Et_3N, v:v:v) to obtain a mixture. The mixture was further purified by normal phase HPLC (Multokrom 100-3 Si, 250 × 20.0 mm, 3 µm) with an eluent mixture of *i*-hexane/MTBE (98:2 (v:v)) at a flow rate of 15.0 mL/min to provide the title compound **11** as a colorless oil.

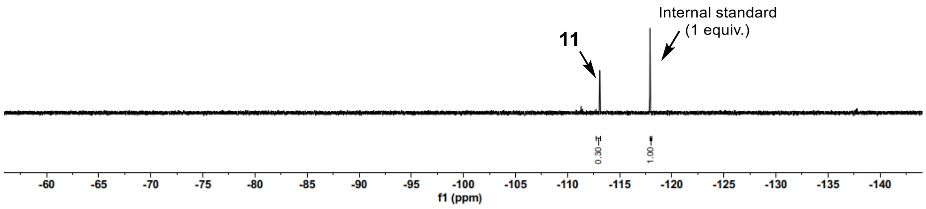

Figure S8. ^{19}F NMR of the reaction mixture for product **11**

$\mathbf{R}_f$ = 0.60 (pentane/EtOAc, 5:1, v/v (UV))

NMR Spectroscopy:

^{1}H NMR (500 MHz, $CDCl_3$, 25 °C, δ): 8.22 (d, *J* = 8.6 Hz, 1H), 8.19 – 8.14 (m, 2H), 8.12 (dd, *J* = 8.4, 1.4 Hz, 1H), 7.81 (ddd, *J* = 8.5, 6.9, 1.5 Hz, 1H), 7.63 – 7.49 (m, 5H).

^{13}C {^{1}H} NMR (126 MHz, $CDCl_3$, 25 °C, δ): 166.0 (d, *J* = 266.6 Hz), 159.0 (d, *J* = 8.3 Hz), 150.2, 138.9, 130.8, 129.9, 129.4 (d, *J* = 4.0 Hz), 128.9 (2C), 127.5 (2C), 126.6, 120.4 (d, *J* = 4.8 Hz), 118.5 (d, *J* = 13.7 Hz), 103.4 (d, *J* = 15.9 Hz).

^{19}F NMR (471 MHz, $CDCl_3$, 25 °C, δ): –113.4 (d, *J* = 11.3 Hz).

HRMS ESIpos (m/z) calc'd for $C_{15}H_{11}NF^+$ $[M+H]^+$, 224.0870; found, 224.0869. Deviation: –0.3 ppm.

7-Chloro-4-fluoroquinoline (12a) and 7-chloro-2-fluoroquinoline (12b)

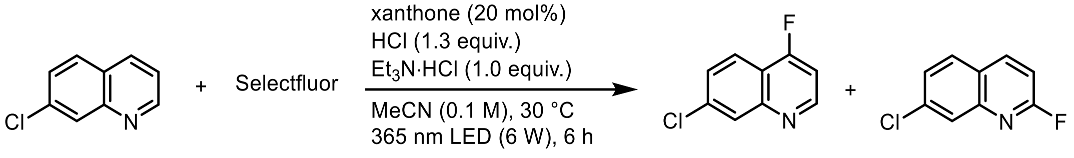

Under an ambient atmosphere, a 4-mL borosilicate vial equipped with a magnetic stir bar was charged with 7-chloroquinoline (16.3 mg, 0.100 mmol, 1.00 equiv.), followed by dry DCM (1.0 mL, *c* = 0.1 M). Subsequently, 4.0 M HCl solution in dioxane (33 µL, 0.13 mmol, 1.30 equiv.) was added. The mixture was stirred at ambient temperature for 10 min. After that, the solvent was removed by a rotary evaporator. Under a nitrogen atmosphere, to the vial were then added xanthone (3.9 mg, 20 µmol, 20 mol%), Selectfluor (106.2 mg, 0.300 mmol, 3.00 equiv.), Et_3N·HCl (13.7 mg, 0.100 mmol, 1.00 equiv.), and dry acetonitrile (1.0 mL, *c* = 0.1 M). The vial was sealed with a septum cap. Then the vial was placed onto an aluminum plate fitted with a 6 W high-power single LED plate (EpiLED Chip, size: 10 × 10 mm, λ_{max} = 365 nm, 600 mA, 9–11 V). Another aluminum block was utilized to hold the vial. The temperature was kept at approximately 30 °C through the use of a cooling fan. The reaction mixture was stirred and irradiated for 6 hours. After that, 2-fluorotoluene (11 µL, 0.10 mmol, 1.0 equiv.) was added as an internal standard, followed by 1 mL of saturated aqueous Na_2CO_3. An aliquot (0.1 mL) of the organic phase was taken and diluted with $CDCl_3$ (0.5 mL), and the yield was determined by ^{19}F NMR integration relative to the internal standard (53% yield, **12a**:**12b** = 1.5:1; standard: δ –118.6 (m) ppm, **12a**: δ –112.30 (t, *J* = 9.0 Hz) ppm, **12b**: δ –61.2 (d, *J* = 7.4 Hz) ppm, Figure S9). To isolate the fluorinated product, an identical reaction without added standard was transferred to a separatory funnel. The mixture was then diluted with 2 mL of ethyl acetate, the layers were separated, and the aqueous layer was extracted twice with ethyl acetate (approx. 2 mL each time). Then the combined organic layer was dried over Na_2SO_4, and the solvent was removed *in vacuo*. The residue was purified by flash column chromatography on silica gel eluting with hexanes/ethyl acetate (100:1 to 50:1 + 1% Et_3N, v:v:v) to afford a mixture. The mixture was further purified by normal phase HPLC (YMC PVA-Sil, 250 × 30.0 mm, 5 µm) with an eluent mixture of *i*-hexane/MTBE (98:2, v:v) at a flow rate of 42.5 mL/min to provide the title compounds **12a** and **12b** as colorless oils.

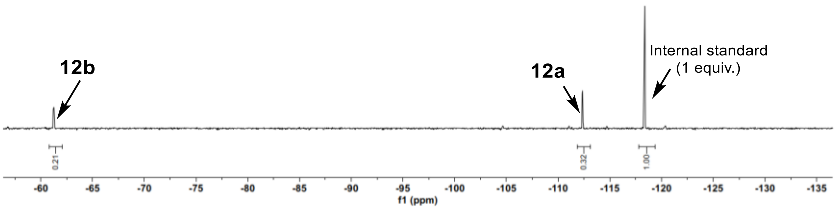

Figure S9. ^{19}F NMR of the reaction mixture for products **12a** and **12b**

7-Chloro-4-fluoroquinoline (**12a**):

R*f* = 0.40 (hexanes/EtOAc, 5:1, v/v (UV))

NMR Spectroscopy:

^{1}H NMR (500 MHz, $CDCl_3$, 25 °C, δ): 8.87 (dd, *J* = 8.2, 5.0 Hz, 1H), 8.14 (t, *J* = 1.9 Hz, 1H), 8.05 (d, *J* = 8.8 Hz, 1H), 7.57 (dd, *J* = 8.9, 2.0 Hz, 1H), 7.11 (dd, *J* = 9.5, 5.0 Hz, 1H).

^{13}C {^{1}H} NMR (151 MHz, $CDCl_3$, 25 °C, δ): 165.3 (d, *J* = 269.9 Hz), 152.7 (d, *J* = 8.3 Hz), 150.8 (d, *J* = 4.2 Hz), 136.8, 128.3 (d, *J* = 3.6 Hz), 128.1, 122.0 (d, *J* = 4.8 Hz), 118.0 (d, *J* = 13.6 Hz), 106.0 (d, *J* = 14.5 Hz).

^{19}F NMR (471 MHz, $CDCl_3$, 25 °C, δ): –112.3 (t, *J* = 9.0 Hz).

HRMS ESIpos (m/z) calc'd for $C_9H_6NClF^+$ $[M+H]^+$, 182.0167; found, 182.0167. Deviation: 0.0 ppm.

7-Chloro-2-fluoroquinoline (**12b**):

R*f* = 0.60 (hexanes/EtOAc, 5:1, v/v (UV))

NMR Spectroscopy:

^{1}H NMR (500 MHz, $CDCl_3$, 25 °C, δ): 8.23 (t, *J* = 8.5 Hz, 1H), 7.95 (d, *J* = 2.1 Hz, 1H), 7.78 (d, *J* = 8.6 Hz, 1H), 7.50 (dd, *J* = 8.6, 2.1 Hz, 1H), 7.09 (dd, *J* = 8.8, 2.7 Hz, 1H).

^{13}C {^{1}H} NMR (126 MHz, $CDCl_3$, 25 °C, δ): 161.9 (d, *J* = 243.8 Hz), 146.5 (d, *J* = 17.3 Hz), 141.9 (d, *J* = 10.2 Hz), 136.8, 128.8, 127.5, 127.5 (d, *J* = 2.5 Hz), 125.4 (d, *J* = 1.8 Hz), 110.5 (d, *J* = 42.3 Hz).

^{19}F NMR (471 MHz, $CDCl_3$, 25 °C, δ): –61.2 (d, *J* = 7.4 Hz).

HRMS ESIpos (m/z) calc'd for $C_9H_6NClF^+$ $[M+H]^+$, 182.0467; found, 182.0168. Deviation: –0.2 ppm.

Methyl 4-fluoro-2-methylquinoline-6-carboxylate (13)

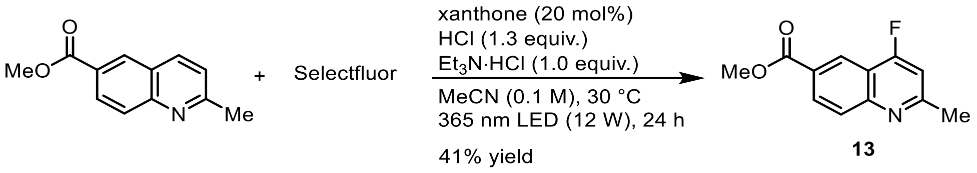

Under an ambient atmosphere, a 20-mL borosilicate vial equipped with a magnetic stir bar was charged with methyl 2-methylquinoline-6-carboxylate (80.4 mg, 0.100 mmol, 1.00 equiv.), followed by dry DCM (4.0 mL, *c* = 0.1 M). Subsequently, 4.0 M HCl solution in dioxane (133 µL, 0.52 mmol, 1.3 equiv.) was added. The mixture was stirred at ambient temperature for 10 min. After that, the solvent was removed by a rotary evaporator. Under a nitrogen atmosphere, to the vial were then added xanthone (15.6 mg, 80 µmol, 20 mol%), Selectfluor (566 mg, 1.60 mmol, 4.00 equiv.), $Et_3N·HCl$ (54.8 mg, 0.400 mmol, 1.00 equiv.), and dry acetonitrile (4.0 mL, *c* = 0.1 M). The vial was sealed with a septum cap. Then the vial was placed onto an aluminum plate fitted with a 12 W high-power single LED plate (EpiLED Chip, size: 10 × 10 mm,

λ_{max} = 365 nm, 1.2 A, 9–11 V). Another aluminum block was utilized to hold the vial. The temperature was kept at approximately 30 °C through the use of a cooling fan. The reaction mixture was stirred and irradiated for 24 hours, then, 2 mL of saturated aqueous Na_2CO_3 were added. The mixture was then diluted with 5 mL of ethyl acetate, the layers were separated, and the aqueous layer was extracted twice with ethyl acetate (approx. 5 mL each time). Then the combined organic layer was dried over Na_2SO_4, and the solvent was removed *in vacuo*. The residue was purified by flash column chromatography on silica gel eluting with hexanes/ethyl acetate (50:1 to 10:1 + 1% Et_3N, v:v:v) to afford a mixture. The mixture was further purified by normal phase HPLC (YMC PVA-Sil, 250 × 30.0 mm, 5 µm) with an eluent mixture of *i*-hexane/2-propanol (98:2, v:v) at a flow rate of 45.2 mL/min to the title compound **13** (36.1 mg, 164 µmol, 41%) as a colorless oil.

R*f* = 0.30 (pentane/EtOAc, 5:1, v/v (UV))

NMR Spectroscopy:

^{1}H NMR (500 MHz, $CDCl_3$, 25 °C, δ): 8.78 (d, *J* = 1.9 Hz, 1H), 8.31 (dd, *J* = 8.9, 2.0 Hz, 1H), 8.05 (dd, *J* = 8.9, 1.9 Hz, 1H), 7.04 (d, *J* = 10.2 Hz, 1H), 3.99 (s, 3H), 2.75 (s, 3H).

^{13}C {^{1}H} NMR (126 MHz, $CDCl_3$, 25 °C, δ): 166.3, 165.8 (d, *J* = 269.7 Hz), 163.5 (d, *J* = 7.8 Hz), 151.6 (d, *J* = 3.6 Hz), 130.2, 128.7 (d, *J* = 3.6 Hz), 127.6, 123.7 (d, *J* = 5.2 Hz), 117.4 (d, *J* = 13.7 Hz), 106.9 (d, *J* = 14.3 Hz), 52.5, 25.9 (d, *J* = 3.0 Hz).

^{19}F NMR (471 MHz, $CDCl_3$, 25 °C, δ): –113.0 (d, *J* = 10.4 Hz).

HRMS ESIpos (m/z) calc'd for $C_{12}H_{11}NO_2F^+$ $[M+H]^+$, 220.0768; found, 220.0769. Deviation: –0.3 ppm.

3-(Phthalimidyl)propyl 4-fluoro-2-methylquinoline-3-carboxylate (14)

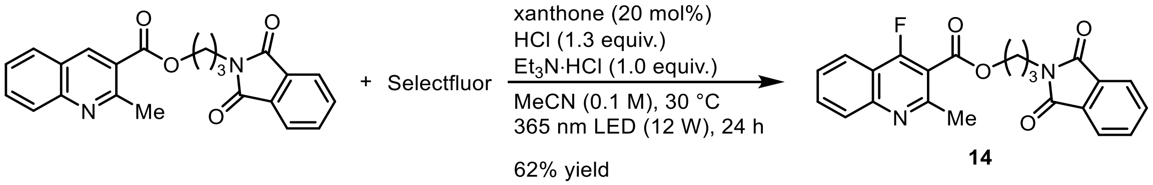

Under an ambient atmosphere, a 20-mL borosilicate vial equipped with a magnetic stir bar was charged with 3-(phthalimidyl)propyl 2-methylquinoline-3-carboxylate (149.6 mg, 0.400 mmol, 1.00 equiv.), followed by dry DCM (4.0 mL, *c* = 0.1 M). Subsequently, 4.0 M HCl solution in dioxane (133 µL, 0.52 mmol, 1.3 equiv.) was added. The mixture was stirred at ambient temperature for 10 min. After that, the solvent was removed by rotary evaporator. Under a nitrogen atmosphere, to the vial were then added xanthone (15.6 mg, 80 µmol, 20 mol%), Selectfluor (425 mg, 1.20 mmol, 3.00 equiv.), Et_3N·HCl (54.8 mg, 0.400 mmol, 1.00 equiv.), and dry acetonitrile (4.0 mL, *c* = 0.1 M). The vial was sealed with a septum cap. Then the vial was placed onto an aluminum plate fitted with a 12 W high-power single LED plate (EpiLED Chip, size: 10 × 10 mm, λ_{max} = 365 nm, 1.2 A, 9–11 V). Another aluminum block was utilized to hold the vial. The temperature was kept at approximately 30 °C through the use of a cooling fan. The reaction mixture was stirred and irradiated for 24 hours, then 2 mL of saturated aqueous Na_2CO_3 were added. The mixture was then diluted by 5 mL of

ethyl acetate, the layers were separated, and the aqueous layer was extracted twice with ethyl acetate (approx. 5 mL each time). Then the combined organic layer was dried over Na_2SO_4, and the solvent was removed *in vacuo*. The residue was purified by flash column chromatography on silica gel eluting with hexanes/ethyl acetate (5:1 + 1% Et_3N, v:v:v) to afford a mixture. The mixture was further purified by normal phase HPLC (YMC PVA-Sil, 250 × 30.0 mm, 5 µm) with an eluent mixture of *i*-hexane/2-propanol (98:2, v:v) at a flow rate of 42.5 mL/min to provide the title compound **14** (97.5 mg, 248 µmol, 62%) as a colorless solid.

R_f = 0.30 (pentane:EtOAc, 2:1, v/v (UV))

NMR Spectroscopy:

^{1}H NMR (500 MHz, $CDCl_3$, 25 °C, δ): 7.95 – 7.90 (m, 2H), 7.72 (ddd, *J* = 8.5, 6.9, 1.5 Hz, 1H), 7.64 (dd, *J* = 5.4, 3.1 Hz, 2H), 7.51 – 7.47 (m, 1H), 7.42 (dd, *J* = 5.4, 3.0 Hz, 2H), 4.42 (t, *J* = 6.0 Hz, 2H), 3.84 (t, *J* = 6.7 Hz, 2H), 2.74 (s, 3H), 2.15 (p, *J* = 6.3 Hz, 2H).

^{13}C {^{1}H} NMR (126 MHz, $CDCl_3$, 25 °C, δ): 168.3, 164.4, 163.4 (d, *J* = 274.9 Hz), 158.1 (d, *J* = 3.0 Hz), 149.5 (d, *J* = 4.3 Hz), 133.8, 131.93 (d, *J* = 4.2 Hz), 128.36 (d, *J* = 3.8 Hz), 126.7, 123.0, 121.2 (d, *J* = 5.4 Hz), 117.2 (d, *J* = 14.3 Hz), 112.2 (d, *J* = 9.0 Hz), 64.0, 35.4, 27.5, 24.3 (d, *J* = 3.5 Hz).

^{19}F NMR (471 MHz, $CDCl_3$, 25 °C, δ): –112.7 (s).

HRMS ESIpos (m/z) calc'd for $C_{22}H_{18}N_2O_4F^+$ $[M+H]^+$, 393.1245; found, 393.1247. Deviation: –0.5 ppm.

4-Fluoro-2-chloroquinoline (15)

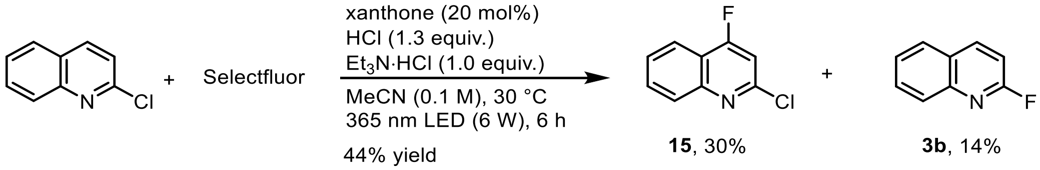

Under an ambient atmosphere, a 4-mL borosilicate vial equipped with a magnetic stir bar was charged with 2-cholroquinoline (16.3 mg, 0.100 mmol, 1.00 equiv.), followed by dry DCM (1.0 mL, *c* = 0.1 M). Subsequently, 4 M HCl solution in dioxane (33 µL, 0.13 mmol, 1.3 equiv.) was added. The mixture was stirred at ambient temperature for 10 min. After that, the solvent was removed by a rotary evaporator. Under a nitrogen atmosphere, to the vial were then added xanthone (3.9 mg, 0.020 mmol, 20 mol%), Selectfluor (106.2 mg, 0.300 mmol, 3.00 equiv.), Et_3N·HCl (13.7 mg, 0.100 mmol, 1.00 equiv.), and dry acetonitrile (1.0 mL, *c* = 0.1 M). The vial was sealed with a septum cap. Then the vial was placed onto an aluminum plate fitted with a 6 W high-power single LED plate (EpiLED Chip, size: 10 × 10 mm, λ_{max} = 365 nm, 600 mA, 9–11 V). Another aluminum block was utilized to hold the vial. The temperature was kept at approximately 30 °C through the use of a cooling fan. The reaction mixture was stirred and irradiated for 6 hours. After that, 2-fluorotoluene (11 µL, 0.10 mmol, 1.0 equiv.) was added as an internal standard, followed by 1 mL of saturated aqueous Na_2CO_3. An aliquot (ca. 0.1 mL) of the organic phase was taken and diluted with $CDCl_3$ (0.5 mL), and the yield was determined by ^{19}F NMR integration relative to the internal standard (44% yield;

15:**3b** = 2:1; standard: δ –118.6 (m) ppm, **15**: δ –109.8 (d, *J* = 8.8 Hz) ppm, and **3b**: δ –62.7 (d, *J* = 8.7 Hz) ppm, Figure S10). TLC of the reaction showed that the C2 fluorinated product overlapped with xanthone, and the production of **3b** was confirmed by the comparison with an authentic sample. To isolate the C4-fluorinated product, an identical reaction without added standard was diluted by 2 mL of ethyl acetate, the layers were separated, and the aqueous layer was extracted twice with ethyl acetate (approx. 2 mL each time). Then the combined organic layer was dried over Na_2SO_4, and the solvent was removed *in vacuo*. The residue was purified by flash column chromatography on silica gel eluting with hexanes/ethyl acetate (20:1 + 1% Et_3N, v:v:v) to afford a mixture. The mixture was further purified by normal phase HPLC (YMC PVA-Sil, 250 × 30.0 mm, 5 µm) with an eluent mixture of *i*-hexane/MTBE (98:2, v:v) at a flow rate of 42.5 mL/min to provide the title compound **15** as a colorless oil.

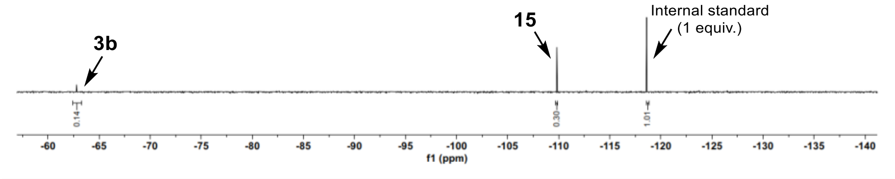

Figure S10. ^{19}F NMR of the reaction mixture for product **15**

R*f* = 0.65 (EtOAc/pentane, 1:5, v/v (UV))

NMR Spectroscopy:

^{1}H NMR (500 MHz, $CDCl_3$, 25 °C, δ): 8.08 (t, *J* = 8.4 Hz, 2H), 7.83 (ddd, *J* = 8.5, 7.0, 1.5 Hz, 1H), 7.65 (ddd, *J* = 8.2, 6.9, 1.1 Hz, 1H), 7.17 (d, *J* = 8.9 Hz, 1H).

^{13}C {^{1}H} NMR (126 MHz, $CDCl_3$, 25 °C, δ): 161.9 (d, *J* = 247.8 Hz), 150.8 (d, *J* = 11.1 Hz), 149.2 (d, *J* = 5.8 Hz), 131.8, 128.4 (d, *J* = 3.6 Hz), 127.3, 120.7 (d, *J* = 4.7 Hz), 118.5 (d, *J* = 13.8 Hz), 107.3 (d, *J* = 19.2 Hz).

^{19}F NMR (471 MHz, $CDCl_3$, 25 °C, δ): –109.8 (d, *J* = 9.0 Hz).

HRMS GC-EI (m/z) calc'd for $C_9H_5NFCl^+$ $[M]^+$, 181.0089; found, 181.0090. Deviation: –0.3 ppm.

2-Cyanoethyl 4-fluoro-2-methylquinoline-3-carboxylate (16)

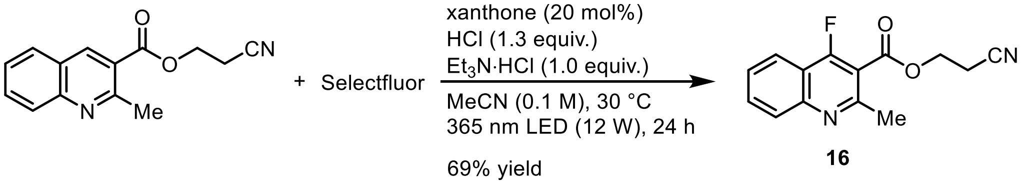

16

Under an ambient atmosphere, a 20-mL borosilicate vial equipped with a magnetic stir bar was charged with 2-cyanoethyl 2-methylquinoline-3-carboxylate (96.0 mg, 0.400 mmol, 1.00 equiv.), followed by dry DCM

(4.0 mL, c = 0.1 M). Subsequently, 4.0 M HCl solution in dioxane (133 μL, 0.52 mmol, 1.3 equiv.) was added. The mixture was stirred at ambient temperature for 10 min. After that, the solvent was removed by a rotary evaporator. Under a nitrogen atmosphere, to the vial were then added xanthone (15.6 mg, 80 μmol, 20 mol%), Selectfluor (425 mg, 1.20 mmol, 3.00 equiv.), $Et_3N·HCl$ (54.8 mg, 0.400 mmol, 1.00 equiv.), and dry acetonitrile (4.0 mL, c = 0.1 M). The vial was sealed with a septum cap. Then the vial was placed onto an aluminum plate fitted with a 12 W high-power single LED plate (EpiLED Chip, size: 10 × 10 mm, λ_{max} = 365 nm, 1.2 A, 9–11 V). Another aluminum block was utilized to hold the vial. The temperature was kept at approximately 30 °C through the use of a cooling fan. The reaction mixture was stirred and irradiated for 24 hours, then, 2 mL of saturated aqueous Na_2CO_3 were added. The mixture was then diluted with 5 mL of ethyl acetate, the layers were separated, and the aqueous layer was extracted twice with ethyl acetate (approx. 5 mL each time). Then the combined organic layer was dried over Na_2SO_4, and the solvent was removed *in vacuo*. The residue was purified by flash column chromatography on silica gel eluting with hexanes/ethyl acetate (5:1 + 1% Et_3N, v:v:v) to afford a mixture. The mixture was further purified by normal phase HPLC (YMC PVA-Sil, 250 × 30.0 mm, 5 μm) with an eluent mixture of *i*-hexane/2-propanol (90:10, v:v) at a flow rate of 42.5 mL/min to provide the title compound **16** (71.2 mg, 276 μmol, 69%) as a colorless oil.

R_f = 0.30 (pentane/EtOAc, 2:1, v/v (UV))

NMR Spectroscopy:

^{1}H NMR (500 MHz, $CDCl_3$, 25 °C, δ): 8.09 (ddd, J = 8.4, 1.4, 0.7 Hz, 1H), 8.03 (ddd, J = 8.6, 1.9, 0.9 Hz, 1H), 7.82 (ddd, J = 8.5, 6.9, 1.4 Hz, 1H), 7.59 (ddd, J = 8.2, 6.9, 1.2 Hz, 1H), 4.64 (t, J = 6.3 Hz, 2H), 2.90 (t, J = 6.3 Hz, 2H), 2.84 (s, 3H).

^{13}C {^{1}H} NMR (126 MHz, $CDCl_3$, 25 °C, δ): 164.0 (d, J = 275.8 Hz), 164.1, 157.9 (d, J = 2.7 Hz), 149.8 (d, J = 6.4 Hz), 132.4, 128.5 (d, J = 3.6 Hz), 127.0, 121.2 (d, J = 5.5 Hz), 117.2 (d, J = 13.9 Hz), 116.5, 111.2 (d, J = 8.4 Hz), 60.0, 24.5 (d, J = 3.5 Hz), 18.1.

^{19}F NMR (471 MHz, $CDCl_3$, 25 °C, δ): –111.5 (s).

HRMS GC-EI (m/z) calc'd for $C_{14}H_{11}N_2O_2F^+$ $[M]^+$, 258.0799; found, 258.0802. Deviation: –1.2 ppm.

2-Bromoethyl 4-fluoro-2-methylquinoline-3-carboxylate (17)

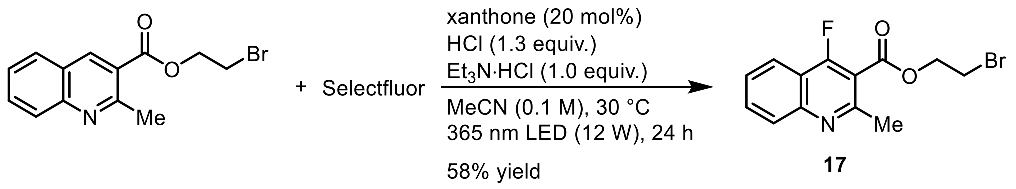

Under an ambient atmosphere, a 20-mL borosilicate vial equipped with a magnetic stir bar was charged with 2-bromoethyl 2-methylquinoline-6-carboxylate (117.6 mg, 0.400 mmol, 1.00 equiv.), followed by dry DCM (4.0 mL, c = 0.1 M). Subsequently, 4.0 M HCl solution in dioxane (133 μL, 0.52 mmol, 1.3 equiv.) was added. The mixture was stirred at ambient temperature for 10 min. After that, the solvent was removed by a rotary

evaporator. Under a nitrogen atmosphere, to the vial were then added xanthone (15.6 mg, 80 μmol, 20 mol%), Selectfluor (425 mg, 1.20 mmol, 3.00 equiv.), $Et_3N{\cdot}HCl$ (54.8 mg, 0.400 mmol, 1.00 equiv.), and dry acetonitrile (4.0 mL, *c* = 0.1 M). The vial was sealed with a septum cap. Then the vial was placed onto an aluminum plate fitted with a 12 W high-power single LED plate (EpiLED Chip, size: 10 × 10 mm, λ_{max} = 365 nm, 1.2 A, 9–11 V). Another aluminum block was utilized to hold the vial. The temperature was kept at approximately 30 °C through the use of a cooling fan. The reaction mixture was stirred and irradiated for 24 hours, then, 2 mL of saturated aqueous Na_2CO_3 were added. The mixture was then diluted with 5 mL of ethyl acetate, the layers were separated, and the aqueous layer was extracted twice with ethyl acetate (approx. 5 mL each time). Then the combined organic layer was dried over Na_2SO_4, and the solvent was removed *in vacuo*. The residue was purified by flash column chromatography on silica gel eluting with hexanes/ethyl acetate (20:1 + 1% Et_3N, v:v:v) to afford a mixture. The mixture was further purified by normal phase HPLC (YMC PVA-Sil, 250 × 30.0 mm, 5 μm) with an eluent mixture of *i*-hexane/2-propanol (98:2, v:v) at a flow rate of 42.5 mL/min to provide the title compound **17** (72.2 mg, 232 μmol, 58%) as a colorless oil.

R_f = 0.30 (pentane/EtOAc, 5:1, v/v (UV))

NMR Spectroscopy:

^{1}H NMR (500 MHz, $CDCl_3$, 25 °C, δ): 8.12 (dd, *J* = 8.5, 1.6 Hz, 1H), 8.06 (d, *J* = 8.5 Hz, 1H), 7.84 (ddd, *J* = 8.2, 7.0, 1.3 Hz, 1H), 7.62 (ddd, *J* = 8.3, 6.9, 1.2 Hz, 1H), 4.77 (t, *J* = 6.1 Hz, 2H), 3.70 (t, *J* = 6.1 Hz, 2H), 2.87 (s, 3H).

^{13}C {^{1}H} NMR (126 MHz, $CDCl_3$, 25 °C, δ): 164.2, 163.6 (d, *J* = 273.9 Hz), 157.9 (d, *J* = 2.7 Hz), 149.7 (d, *J* = 6.0 Hz), 132.2, 128.5 (d, *J* = 3.5 Hz), 126.9, 121.2 (d, *J* = 5.6 Hz), 117.3 (d, *J* = 14.2 Hz), 111.9 (d, *J* = 8.9 Hz), 65.2, 28.1, 24.5 (d, *J* = 3.4 Hz).

^{19}F NMR (471 MHz, $CDCl_3$, 25 °C, δ): –112.2 (s).

HRMS GC-EI (m/z) calc'd for $C_{13}H_{11}NO_2FBr^+$ $[M]^+$, 310.9955; found, 310.9952. Deviation: –1.1 ppm.

Phosphorylmethyl 4-fluoro-2-methylquinoline-3-carboxylate 18

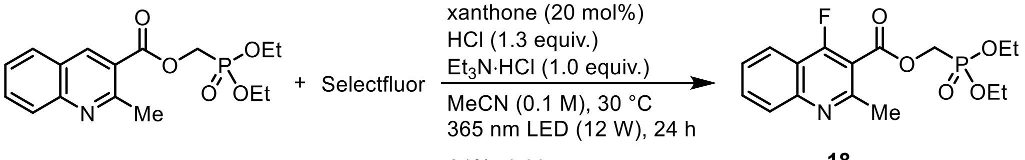

18

Under an ambient atmosphere, a 20-mL borosilicate vial equipped with a magnetic stir bar was charged with phosphorylmethyl 2-methylquinoline-3-carboxylate (134.8 mg, 0.400 mmol, 1.00 equiv.), followed by dry DCM (4.0 mL, *c* = 0.1 M). Subsequently, 4.0 M HCl solution in dioxane (133 μL, 0.52 mmol, 1.3 equiv.) was added. The mixture was stirred at ambient temperature for 10 min. After that, the solvent was removed by a rotary evaporator. Under a nitrogen atmosphere, to the vial were then added xanthone (15.6 mg, 80 μmol, 20 mol%), Selectfluor (425 mg, 1.20 mmol, 3.00 equiv.), $Et_3N{\cdot}HCl$ (54.8 mg, 0.400 mmol, 1.00 equiv.), and dry

acetonitrile (4.0 mL, c = 0.1 M). The vial was sealed with a septum cap. Then the vial was placed onto an aluminum plate fitted with a 12 W high-power single LED plate (EpiLED Chip, size: 10 × 10 mm, λ_{max} = 365 nm, 1.2 A, 9–11 V). Another aluminum block was utilized to hold the vial. The temperature was kept at approximately 30 °C through the use of a cooling fan. The reaction mixture was stirred and irradiated for 24 hours, then, 2 mL of saturated aqueous Na_2CO_3 were added. The mixture was then diluted with 5 mL of ethyl acetate, the layers were separated, and the aqueous layer was extracted twice with ethyl acetate (approx. 5 mL each time). Then the combined organic layers were dried over Na_2SO_4, and the solvent was removed *in vacuo*. The residue was purified by flash column chromatography on silica gel eluting with hexanes/ethyl acetate (from 50:1 to 1:1 + 1% Et_3N, v:v:v) to afford a mixture. The mixture was further purified by normal phase HPLC (YMC PVA-Sil, 250 × 30.0 mm, 5 µm) with an eluent mixture of *i*-hexane/2-propanol (95:5, v:v) at a flow rate of 42.5 mL/min to provide the title compound **18** (44.0 mg, 124 µmol, 31%) as a colorless oil.

R*f* = 0.30 (EtOAc/pentane, 1:1, v/v (UV))

NMR Spectroscopy:

^{1}H NMR (500 MHz, $CDCl_3$, 25 °C, δ): 8.10 (dd, J = 8.2, 1.4 Hz, 1H), 8.08 – 7.99 (m, 1H), 7.83 (ddd, J = 8.5, 6.9, 1.4 Hz, 1H), 7.61 (ddd, J = 8.1, 6.9, 1.1 Hz, 1H), 4.73 (d, J = 8.7 Hz, 2H), 4.25 (dq, J = 8.2, 7.1 Hz, 4H), 2.85 (s, 3H), 1.39 (t, J = 7.1 Hz, 6H).

^{13}C {^{1}H} NMR (126 MHz, $CDCl_3$, 25 °C, δ): 163.9 (d, J = 275.0 Hz), 163.8 (d, J = 8.3 Hz), 157.9 (d, J = 2.4 Hz), 149.8 (d, J = 5.9 Hz), 132.3, 128.5 (d, J = 3.6 Hz), 127.0, 121.2 (d, J = 5.6 Hz), 117.2 (d, J = 14.2 Hz), 111.4 (d, J = 8.6 Hz), 63.0 (d, J = 6.4 Hz), 57.8 (d, J = 168.0 Hz), 24.4 (d, J = 3.3 Hz), 16.4 (d, J = 5.9 Hz).

^{19}F NMR (471 MHz, $CDCl_3$, 25 °C, δ): –111.5 (s).

^{31}P NMR (203 MHz, $CDCl_3$, 25 °C, δ): 18.0.

HRMS GC-EI (m/z) calc'd for $C_{16}H_{19}NO_5FP^+$ $[M]^+$, 355.0979; found, 355.0981. Deviation: –0.6 ppm.

4-Fluoro-7-sulfonamidylquinoline 19a and 2-fluoro-7-sulfonamidylquinoline 19b

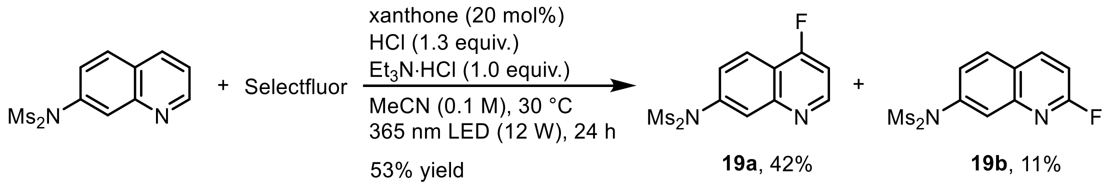

Under an ambient atmosphere, a 20-mL borosilicate vial equipped with a magnetic stir bar was charged with 7-sulfonamidylquinoline (120.0 mg, 0.400 mmol, 1.00 equiv.), followed by dry DCM (4.0 mL, c = 0.1 M). Subsequently, 4.0 M HCl solution in dioxane (133 µL, 0.52 mmol, 1.3 equiv.) was added. The mixture was stirred at ambient temperature for 10 min. After that, the solvent was removed by a rotary evaporator. Under a nitrogen atmosphere, to the vial were then added xanthone (15.6 mg, 80 µmol, 20 mol%), Selectfluor

(425 mg, 1.20 mmol, 3.00 equiv.), $Et_3N{\cdot}HCl$ (54.8 mg, 0.400 mmol, 1.00 equiv.), and dry acetonitrile (4.0 mL, *c* = 0.1 M). The vial was sealed with a septum cap. Then the vial was placed onto an aluminum plate fitted with a 12 W high-power single LED plate (EpiLED Chip, size: 10 × 10 mm, λ_{max} = 365 nm, 1.2 A, 9–11 V). Another aluminum block was utilized to hold the vial. The temperature was kept at approximately 30 °C through the use of a cooling fan. The reaction mixture was stirred and irradiated for 24 hours, then, 2 mL of saturated aqueous Na_2CO_3 were added. The mixture was then diluted with 5 mL of ethyl acetate, the layers were separated, and the aqueous layer was extracted twice with ethyl acetate (approx. 5 mL each time). Then the combined organic layer was dried over Na_2SO_4, and the solvent was removed *in vacuo*. The residue was purified by flash column chromatography on silica gel eluting with hexanes/ethyl acetate (from 10:1 to 2:1 + 1% Et_3N, v:v:v) to afford the title compounds **19a** (51.1 mg, 42%) and **19b** (13.9 mg, 11%) as colorless solids. Further isolation by normal phase HPLC (Multokrom 100-5 Si, 250 × 30.0 mm, 5 µm) with an eluent mixture of *i*-hexane/2-propanol (97:3, v:v) at a flow rate of 20.0 mL/min provided the title compounds **19a** and **19b** as colorless solids.

4-Fluoro-7-sulfonamidylquinoline (**19a**)

R_f = 0.40 (pentane/EtOAc, 1:1, v/v (UV))

NMR Spectroscopy:

^{1}H NMR (500 MHz, $CDCl_3$, 25 °C, δ): 8.95 (dd, *J* = 8.1, 5.0 Hz, 1H), 8.25 – 8.17 (m, 2H), 7.56 (dd, *J* = 8.8, 2.1 Hz, 1H), 7.21 (dd, *J* = 9.4, 5.0 Hz, 1H).

^{13}C {^{1}H} NMR (126 MHz, $CDCl_3$, 25 °C, δ): 165.3 (d, *J* = 270.3 Hz), 153.2 (d, *J* = 7.5 Hz), 135.5, 131.8, 128.8, 122.6 (d, *J* = 4.8 Hz), 120.6 (d, *J* = 13.8 Hz), 107.4 (d, *J* = 14.3 Hz), 43.1.

^{19}F NMR (471 MHz, $CDCl_3$, 25 °C, δ): –110.9 (brs).

HRMS GC-EI (m/z) calc'd for $C_{11}H_{11}N_2O_4S_2F^+$ $[M]^+$, 318.0139; found, 318.0143. Deviation: –1.3 ppm.

2-Fluoro-7-sulfonamidylquinoline (**19b**)

R_f = 0.70 (pentane/EtOAc, 1:1, v/v (UV))

NMR Spectroscopy:

^{1}H NMR (500 MHz, $CDCl_3$, 25 °C, δ): 8.31 (t, *J* = 8.4 Hz, 1H), 8.00 (d, *J* = 2.1 Hz, 1H), 7.95 (d, *J* = 8.5 Hz, 1H), 7.50 (dd, *J* = 8.5, 2.1 Hz, 1H), 7.20 (dd, *J* = 8.9, 2.6 Hz, 1H), 3.48 (s, 6H).

^{13}C {^{1}H} NMR (126 MHz, $CDCl_3$, 25 °C, δ): 162.0 (d, *J* = 245.1 Hz), 146.0 (d, *J* = 17.8 Hz), 141.9 (d, *J* = 10.1 Hz), 135.4, 130.7, 129.2, 128.2 (d, *J* = 2.4 Hz), 127.7 (d, *J* = 2.1 Hz), 112.3 (d, *J* = 42.3 Hz), 43.0.

^{19}F NMR (471 MHz, $CDCl_3$, 25 °C, δ): –59.2 (d, *J* = 8.1 Hz).

HRMS GC-EI (m/z) calc'd for $C_{11}H_{11}N_2O_4S_2F^+$ $[M]^+$, 318.0139; found, 318.0140. Deviation: –0.3 ppm.

4-Fluoro-6-sulfonyloxyquinoline 20a and 2-fluoro-6-sulfonyloxyquinoline 20b

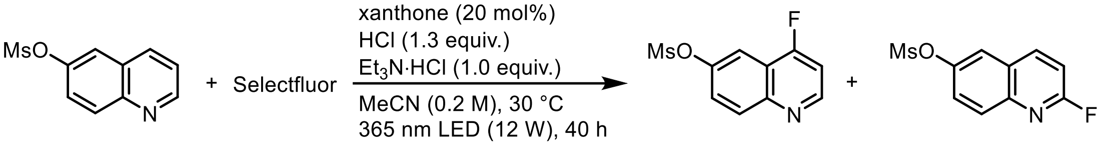

20a, 34% **20b**, 24%

Under an ambient atmosphere, a 20-mL borosilicate vial equipped with a magnetic stir bar was charged with 6-sulfonyloxyquinoline (223.0 mg, 1.00 mmol, 1.00 equiv.), followed by dry DCM (10.0 mL, *c* = 0.1 M). Subsequently, 4.0 M HCl solution in dioxane (333 µL, 1.3 mmol, 1.3 equiv.) was added. The mixture was stirred at ambient temperature for 10 min. After that, the solvent was removed by a rotary evaporator. Under a nitrogen atmosphere, to the vial were then added xanthone (39.0 mg, 0.200 mmol, 20 mol%), Selectfluor (1416 mg, 4.00 mmol, 4.00 equiv.), $Et_3N\cdot HCl$ (137.0 mg, 1.00 mmol, 1.00 equiv.), and dry acetonitrile (5.0 mL, *c* = 0.2 M). The vial was sealed with a septum cap. Then the vial was placed onto an aluminum plate fitted with a 12 W high-power single LED plate (EpiLED Chip, size: 10 × 10 mm, λ_{max} = 365 nm, 1.2 A, 9–11 V). Another aluminum block was utilized to hold the vial. The temperature was kept at approximately 30 °C through the use of a cooling fan. The reaction mixture was stirred and irradiated for 40 hours, then, 4 mL of saturated aqueous Na_2CO_3 were added. The mixture was then diluted with 10 mL of ethyl acetate, the layers were separated, and the aqueous layer was extracted twice with ethyl acetate (approx. 10 mL each time). Then the combined organic layer was dried over Na_2SO_4, and the solvent was removed *in vacuo*. The residue was purified by flash column chromatography on silica gel eluting with hexanes/ethyl acetate (from 10:1 to 4:1 + 1% Et_3N, v:v:v) to afford the title compounds **20a** (81.9 mg, 339 µmol, 34%) and **20b** (57.8 mg, 239 µmol, 24%) as colorless oils.

4-Fluoro-6-sulfonyloxyquinoline (**20a**)

R*f* = 0.50 (pentane/EtOAc, 1:1, v/v (UV))

NMR Spectroscopy:

^{1}H NMR (500 MHz, $CDCl_3$, 25 °C, δ): 8.91 (dd, *J* = 8.2, 5.0 Hz, 1H), 8.20 (dd, *J* = 9.3, 1.7 Hz, 1H), 8.00 (d, *J* = 2.6 Hz, 1H), 7.70 (dd, *J* = 9.2, 2.7 Hz, 1H), 7.18 (dd, *J* = 9.5, 5.0 Hz, 1H), 3.26 (s, 3H).

^{13}C {^{1}H} NMR (126 MHz, $CDCl_3$, 25 °C, δ): 165.2 (d, *J* = 270.1 Hz), 152.2 (d, *J* = 7.9 Hz), 148.9 (d, *J* = 4.1 Hz), 147.2, 132.0 (d, *J* = 3.6 Hz), 126.0, 120.1 (d, *J* = 13.6 Hz), 112.9 (d, *J* = 5.3 Hz), 106.7 (d, *J* = 14.3 Hz), 38.1.

^{19}F NMR (471 MHz, $CDCl_3$, 25 °C, δ): –111.2 (t, *J* = 8.0 Hz).

HRMS GC-EI (m/z) calc'd for $C_{10}H_8NO_3FS^+$ $[M]^+$, 241.0203; found, 241.0208. Deviation: –1.9 ppm.

2-Fluoro-6-sulfonyloxyquinoline (**20b**)

R*f* = 0.70 (pentane/EtOAc, 1:1, v/v (UV))

NMR Spectroscopy:

^{1}H NMR (500 MHz, $CDCl_3$, 25 °C, δ): 8.27 (t, *J* = 8.4 Hz, 1H), 8.01 (d, *J* = 9.1 Hz, 1H), 7.81 (d, *J* = 2.7 Hz, 1H), 7.64 (dd, *J* = 9.1, 2.6 Hz, 1H), 7.17 (dd, *J* = 8.9, 2.7 Hz, 1H), 3.23 (s, 3H).

^{13}C {^{1}H} NMR (126 MHz, $CDCl_3$, 25 °C, δ): 161.6 (d, *J* = 243.8 Hz), 146.6 (d, *J* = 2.8 Hz), 144.5 (d, *J* = 17.2 Hz), 142.0 (d, *J* = 10.1 Hz), 130.6, 127.2 (d, *J* = 2.1 Hz), 125.5, 119.7, 111.6 (d, *J* = 42.4 Hz), 37.9.

^{19}F NMR (471 MHz, $CDCl_3$, 25 °C, δ): –60.3 (d, *J* = 7.8 Hz).

HRMS GC-EI (m/z) calc'd for $C_{10}H_8NO_3FS^+$ [M]$^+$, 241.0203; found, 241.0206. Deviation: –1.2 ppm.

4-Fluorobenzo[*f*]quinoline (21)

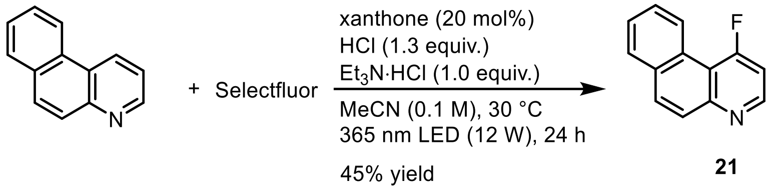

Under an ambient atmosphere, a 20-mL borosilicate vial equipped with a magnetic stir bar was charged with benzo[*f*]quinoline (71.6 mg, 0.400 mmol, 1.00 equiv.), followed by dry DCM (4.0 mL, *c* = 0.1 M). Subsequently, 4.0 M HCl solution in dioxane (133 µL, 0.52 mmol, 1.3 equiv.) was added. The mixture was stirred at ambient temperature for 10 min. After that, the solvent was removed by a rotary evaporator. Under a nitrogen atmosphere, to the vial were then added xanthone (15.6 mg, 80 µmol, 20 mol%), Selectfluor (425 mg, 1.20 mmol, 3.00 equiv.), Et_3N·HCl (54.8 mg, 0.400 mmol, 1.00 equiv.), and dry acetonitrile (4.0 mL, *c* = 0.1 M). The vial was sealed with a septum cap. Then the vial was placed onto an aluminum plate fitted with a 12 W high-power single LED plate (EpiLED Chip, size: 10 × 10 mm, λ_{max} = 365 nm, 1.2 A, 9–11 V). Another aluminum block was utilized to hold the vial. The temperature was kept at approximately 30 °C through the use of a cooling fan. The reaction mixture was stirred and irradiated for 24 hours, then, 2 mL of saturated aqueous Na_2CO_3 were added. The mixture was then diluted with 5 mL of ethyl acetate, the layers were separated, and the aqueous layer was extracted twice with ethyl acetate (approx. 5 mL each time). Then the combined organic layer was dried over Na_2SO_4, and the solvent was removed *in vacuo*. The residue was purified by flash column chromatography on silica gel eluting with hexanes/ethyl acetate (from pentane to 5:1 + 1% Et_3N, v:v:v) to afford the title compounds **21** (35.4 mg, 180 µmol, 45%) as a colorless oil.

R*f* = 0.40 (pentane/EtOAc, 2:1, v/v (UV))

NMR Spectroscopy:

^{1}H NMR (500 MHz, $CDCl_3$, 25 °C, δ): 9.02 (dt, *J* = 8.2, 1.6 Hz, 1H), 8.88 (dd, *J* = 7.7, 5.1 Hz, 1H), 8.06 –

7.98 (m, 2H), 7.97 (dd, *J* = 7.7, 1.7 Hz, 1H), 7.76 – 7.67 (m, 2H), 7.31 (dd, *J* = 12.9, 5.1 Hz, 1H).

^{13}C {^{1}H} NMR (126 MHz, $CDCl_3$, 25 °C, δ): 167.7 (d, *J* = 269.4 Hz), 151.8 (d, *J* = 4.2 Hz), 150.4 (d, *J* = 9.8 Hz), 132.6, 132.2, 128.9, 128.1, 128.0 (d, *J* = 1.4 Hz), 127.9, 127.8 (d, *J* = 2.0 Hz), 127.7 (d, *J* = 4.4 Hz), 116.0 (d, *J* = 6.3 Hz), 108.9 (d, *J* = 19.6 Hz).

^{19}F NMR (471 MHz, $CDCl_3$, 25 °C, δ): –100.2 (brs).

HRMS GC-CI Ammonia (m/z) calc'd for $C_{13}H_8NF^+$ [M]$^+$, 197.0635; found, 197.0634. Deviation: +0.3 ppm.

Methyl 4-(4-fluoro-2-methylquinolin-6-yl)benzoate (22)

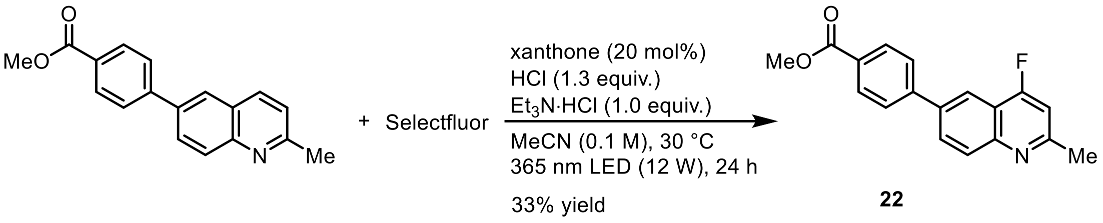

22

Under an ambient atmosphere, a 20-mL borosilicate vial equipped with a magnetic stir bar was charged with methyl 4-(2-methylquinolin-6-yl)benzoate (110.8 mg, 0.400 mmol, 1.00 equiv.), followed by dry DCM (4.0 mL, *c* = 0.1 M). Subsequently, 4.0 M HCl solution in dioxane (133 µL, 0.52 mmol, 1.3 equiv.) was added. The mixture was stirred at ambient temperature for 10 min. After that, the solvent was removed by a rotary evaporator. Under a nitrogen atmosphere, to the vial were then added xanthone (15.6 mg, 80 µmol, 20 mol%), Selectfluor (425 mg, 1.20 mmol, 3.00 equiv.), Et_3N·HCl (54.8 mg, 0.400 mmol, 1.00 equiv.), and dry acetonitrile (4.0 mL, *c* = 0.1 M). The vial was sealed with a septum cap. Then the vial was placed onto an aluminum plate fitted with a 12 W high-power single LED plate (EpiLED Chip, size: 10 × 10 mm, λ_{max} = 365 nm, 1.2 A, 9–11 V). Another aluminum block was utilized to hold the vial. The temperature was kept at approximately 30 °C through the use of a cooling fan. The reaction mixture was stirred and irradiated for 24 hours, then, 2 mL of saturated aqueous Na_2CO_3 were added. The mixture was then diluted with 5 mL of ethyl acetate, the layers were separated, and the aqueous layer was extracted twice with ethyl acetate (approx. 5 mL each time). Then the combined organic layer was dried over Na_2SO_4, and the solvent was removed *in vacuo*. The residue was purified by flash column chromatography on silica gel eluting with hexanes/ethyl acetate (from pentane to 20:1 + 1% Et_3N, v:v:v) to afford the title compounds **22** (38.8 mg, 132 µmol, 33%) as a colorless solid. Further purification by normal phase HPLC (YMC PVA-Sil, 250 × 30.0 mm, 5 µm) with an eluent mixture of *i*-hexane/2-propanol (98:2, v:v) at a flow rate of 20.0 mL/min provided the title compounds **22** as a colorless solid.

R_f = 0.30 (pentane/EtOAc, 5:1, v/v (UV))

NMR Spectroscopy:

^{1}H NMR (500 MHz, $CDCl_3$, 25 °C, δ): 8.26 (d, *J* = 2.1 Hz, 1H), 8.20 – 8.12 (m, 3H), 8.02 (dd, *J* = 8.8, 2.1 Hz, 1H), 7.79 (d, *J* = 8.5 Hz, 2H), 7.04 (d, *J* = 10.4 Hz, 1H), 3.96 (s, 3H), 2.77 (s, 3H).

^{13}C {^{1}H} NMR (126 MHz, $CDCl_3$, 25 °C, δ): 167.0, 165.7 (d, *J* = 268.7 Hz), 161.3 (d, *J* = 8.0 Hz), 149.6, 144.5, 137.7, 130.5, 130.3, 129.7, 129.0, 127.5, 118.7 (d, *J* = 4.6 Hz), 118.4 (d, *J* = 13.4 Hz), 106.9 (d, *J* = 14.7 Hz), 52.4, 25.6.

^{19}F NMR (471 MHz, $CDCl_3$, 25 °C, δ): –113.7 (brs).

HRMS GC-EI (m/z) calc'd for $C_{18}H_{14}NO_2F^+$ $[M+H]^+$, 295.1003; found, 295.1008. Deviation: –1.7 ppm.

4-Fluoro-8-sulfonyloxyquinoline 23a and 2-fluoro-8-sulfonyloxyquinoline 23b

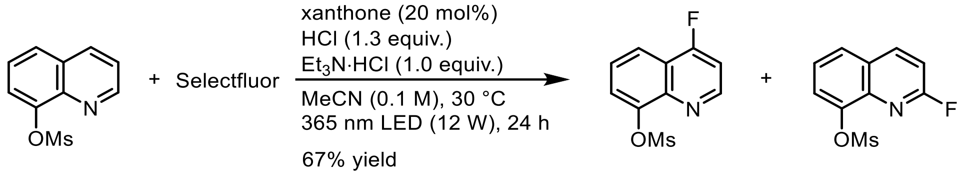

23a, 42% **23b**, 25%

Under an ambient atmosphere, a 20-mL borosilicate vial equipped with a magnetic stir bar was charged with 8-sulfonyloxyquinoline (89.2 mg, 0.400 mmol, 1.00 equiv.), followed by dry DCM (4.0 mL, *c* = 0.1 M). Subsequently, 4.0 M HCl solution in dioxane (133 µL, 0.52 mmol, 1.3 equiv.) was added. The mixture was stirred at ambient temperature for 10 min. After that, the solvent was removed by a rotary evaporator. Under a nitrogen atmosphere, to the vial were then added xanthone (15.6 mg, 80 µmol, 20 mol%), Selectfluor (425 mg, 1.20 mmol, 3.00 equiv.), Et_3N·HCl (54.8 mg, 0.400 mmol, 1.00 equiv.), and dry acetonitrile (4.0 mL, *c* = 0.1 M). The vial was sealed with a septum cap. Then the vial was placed onto an aluminum plate fitted with a 12 W high-power single LED plate (EpiLED Chip, size: 10 × 10 mm, λ_{max} = 365 nm, 1.2 A, 9–11 V). Another aluminum block was utilized to hold the vial. The temperature was kept at approximately 30 °C through the use of a cooling fan. The reaction mixture was stirred and irradiated for 24 hours, then, 2 mL of saturated aqueous Na_2CO_3 were added. The mixture was then diluted with 5 mL of ethyl acetate, the layers were separated, and the aqueous layer was extracted twice with ethyl acetate (approx. 5 mL each time). Then the combined organic layer was dried over Na_2SO_4, and the solvent was removed *in vacuo*. The residue was purified by flash column chromatography on silica gel eluting with hexanes/ethyl acetate (from 10:1 to 4:1 + 1% Et_3N, v:v:v) to afford the title compounds **23a** (40.5 mg, 168 µmol, 42%) and **23b** (24.1 mg, 100 µmol, 25%) as colorless oils.

4-Fluoro-8-sulfonyloxyquinoline (**23a**)

R_f = 0.50 (pentane/EtOAc, 1:1, v/v (UV))

NMR Spectroscopy:

^{1}H NMR (500 MHz, $CDCl_3$, 25 °C, δ): 8.92 (dd, *J* = 8.1, 5.0 Hz, 1H), 8.08 (dd, *J* = 8.4, 1.4 Hz, 1H), 7.78 (dd, *J* = 7.7, 1.4 Hz, 1H), 7.63 (t, *J* = 8.0 Hz, 1H), 7.20 (dd, *J* = 9.5, 5.0 Hz, 1H), 3.46 (s, 3H).

^{13}C {^{1}H} NMR (126 MHz, $CDCl_3$, 25 °C, δ): 165.4 (d, *J* = 270.0 Hz), 152.3 (d, *J* = 8.4 Hz), 145.5 (d, *J* = 5.7 Hz), 143.8 (d, *J* = 4.4 Hz), 127.0, 125.0, 121.5 (d, *J* = 13.9 Hz), 120.1 (d, *J* = 5.3 Hz), 106.9 (d,

J = 14.5 Hz), 39.4.

^{19}F NMR (471 MHz, $CDCl_3$, 25 °C, δ): –110.3 (t, *J* = 8.4 Hz).

HRMS GC-EI (m/z) calc'd for $C_{10}H_8NO_3FS^+$ $[M]^+$, 241.0202; found, 241.0204. Deviation: –0.7 ppm.

2-Fluoro-8-sulfonyloxyquinoline (**23b**)

R*f* = 0.60 (pentane/EtOAc, 1:1, v/v (UV))

NMR Spectroscopy:

^{1}H NMR (500 MHz, $CDCl_3$, 25 °C, δ): 8.32 (dd, *J* = 8.8, 7.8 Hz, 1H), 7.82 (dd, *J* = 8.2, 1.4 Hz, 1H), 7.74 (dd, *J* = 7.7, 1.4 Hz, 1H), 7.56 (t, *J* = 7.9 Hz, 1H), 7.16 (dd, *J* = 8.8, 2.8 Hz, 1H), 3.51 (s, 3H).

^{13}C {^{1}H} NMR (126 MHz, $CDCl_3$, 25 °C, δ): 161.9 (d, *J* = 245.5 Hz), 144.7 (d, *J* = 2.1 Hz), 142.5 (d, *J* = 10.0 Hz), 139.1 (d, *J* = 16.6 Hz), 128.5 (d, *J* = 1.9 Hz), 126.9, 126.3 (d, *J* = 2.4 Hz), 125.2, 111.5 (d, *J* = 42.3 Hz), 39.6.

^{19}F NMR (471 MHz, $CDCl_3$, 25 °C, δ): –59.3 (d, *J* = 7.3 Hz).

HRMS GC-EI (m/z) calc'd for $C_{10}H_8NO_3FS^+$ $[M]^+$, 241.0203; found, 241.0204. Deviation: –0.3 ppm.

Trifluoroethyl 4-fluoro-quinoline-7-carboxylate (24a) and trifluoroethyl 4-fluoro-quinoline-7-carboxylate (24b)

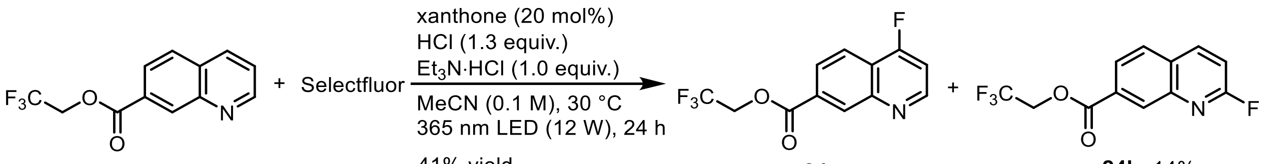

24a, 27% **24b**, 14%

Under an ambient atmosphere, a 20-mL borosilicate vial equipped with a magnetic stir bar was charged with trifluoroethyl quinoline-7-carboxylate (102.0 mg, 0.400 mmol, 1.00 equiv.), followed by dry DCM (4.0 mL, *c* = 0.1 M). Subsequently, 4.0 M HCl solution in dioxane (133 µL, 0.52 mmol, 1.3 equiv.) was added. The mixture was stirred at ambient temperature for 10 min. After that, the solvent was removed by a rotary evaporator. Under a nitrogen atmosphere, to the vial were then added xanthone (15.6 mg, 80 µmol, 20 mol%), Selectfluor (425 mg, 1.20 mmol, 3.00 equiv.), $Et_3N{\cdot}HCl$ (54.8 mg, 0.400 mmol, 1.00 equiv.), and dry acetonitrile (4.0 mL, *c* = 0.1 M). The vial was sealed with a septum cap. Then the vial was placed onto an aluminum plate fitted with a 12 W high-power single LED plate (EpiLED Chip, size: 10 × 10 mm, λ_{max} = 365 nm, 1.2 A, 9–11 V). Another aluminum block was utilized to hold the vial. The temperature was kept at approximately 30 °C through the use of a cooling fan. The reaction mixture was stirred and irradiated for 24 hours, then, 2 mL of saturated aqueous Na_2CO_3 were added. The mixture was then diluted with 5 mL of ethyl acetate, the layers were separated, and the aqueous layer was extracted twice with ethyl acetate (approx. 5 mL each time). Then the combined organic layers were dried over Na_2SO_4, and the solvent was removed *in vacuo*. The residue was purified by flash column chromatography on silica gel eluting with hexanes/ethyl acetate (from

50:1 + 1% Et_3N, v:v:v) to afford the title compounds **24a** (29.4 mg, 27%) and **24b** (16.0 mg, 14%) as colorless oils. Further purification by normal phase HPLC (YMC PVA-Sil, 250 × 30.0 mm, 5 µm) with an eluent mixture of *i*-hexane/MTBE (95:5, v:v) at a flow rate of 20.0 mL/min provided the title compounds **24a** and **24b** as colorless oils.

Trifluoroethyl 4-fluoro-quinoline-7-carboxylate (**24a**)

$\mathbf{R}_f$ = 0.30 (pentane/EtOAc, 5:1, v/v (UV))

NMR Spectroscopy:

^{1}H NMR (500 MHz, $CDCl_3$, 25 °C, δ): 8.97 (dd, *J* = 8.2, 4.9 Hz, 1H), 8.90 (d, *J* = 1.6 Hz, 1H), 8.22 – 8.13 (m, 2H), 7.22 (dd, *J* = 9.3, 5.0 Hz, 1H), 4.79 (q, *J* = 8.3 Hz, 2H).

^{13}C {^{1}H} NMR (126 MHz, $CDCl_3$, 25 °C, δ): 165.19 (d, *J* = 270.5 Hz), 164.4, 152.9 (d, *J* = 7.8 Hz), 149.7 (d, *J* = 4.1 Hz), 132.6 (d, *J* = 3.6 Hz), 130.4, 126.5, 123.1 (q, *J* = 277.2 Hz), 122.7 (d, *J* = 13.6 Hz), 121.6 (d, *J* = 4.8 Hz), 107.8 (d, *J* = 14.5 Hz), 61.4 (q, *J* = 37.0 Hz).

^{19}F NMR (471 MHz, $CDCl_3$, 25 °C, δ): –73.6 (t, *J* = 8.1 Hz, 3F), –111.4 (t, *J* = 8.8 Hz, 1F).

HRMS GC-EI (m/z) calc'd for $C_{12}H_7NO_2F^+$ [M]$^+$, 273.0407; found, 273.0402. Deviation: –1.8 ppm.

Trifluoroethyl 2-fluoro-quinoline-7-carboxylate (**24b**)

$\mathbf{R}_f$ = 0.35 (pentane/EtOAc, 5:1, v/v (UV))

NMR Spectroscopy:

^{1}H NMR (500 MHz, $CDCl_3$, 25 °C, δ): 8.72 – 8.71 (m, 1H), 8.32 (t, *J* = 8.3 Hz, 1H), 8.16 (dd, *J* = 8.5, 1.7 Hz, 1H), 7.95 (d, *J* = 8.5 Hz, 1H), 7.23 (dd, *J* = 8.8, 2.7 Hz, 1H), 4.78 (q, *J* = 8.4 Hz, 2H).

^{13}C {^{1}H} NMR (126 MHz, $CDCl_3$, 25 °C, δ): 164.5, 161.9 (d, *J* = 244.9 Hz), 145.3 (d, *J* = 17.2 Hz), 141.9 (d, *J* = 10.1 Hz), 131.4, 130.3, 129.9, 128.3, 126.0 (d, *J* = 2.4 Hz), 123.1 (q, *J* = 277.1 Hz), 113.0 (d, *J* = 42.4 Hz), 61.3 (q, *J* = 36.9 Hz).

^{19}F NMR (471 MHz, $CDCl_3$, 25 °C, δ): –59.4 (dd, *J* = 7.9, 2.6 Hz, 1F), –73.6 (t, *J* = 8.3 Hz, 3F).

HRMS GC-EI (m/z) calc'd for $C_{12}H_7NO_2F^+$ [M]$^+$, 273.0407; found, 273.0413. Deviation: –2.0 ppm.

Methyl 4-fluoro-6-methylnicotinate (25)

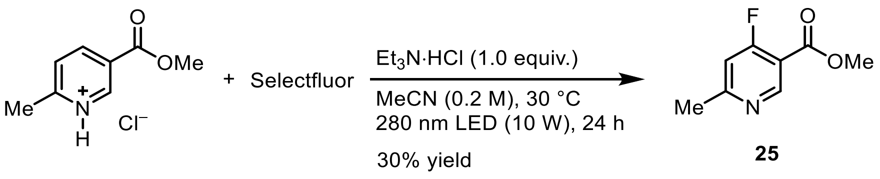

Under a nitrogen atmosphere, a 5-mL quartz vial equipped with a magnetic stri bar was charged with methyl 6-methylnicotinate hydrochloride (74.8 mg, 0.400 mmol, 1.00 equiv.), Selectfluor (566 mg, 1.60 mmol, 4.00 equiv.), $Et_3N{\cdot}HCl$ (54.8 mg, 0.400 mmol, 1.00 equiv.), and dry acetonitrile (2.0 mL, *c* = 0.2 M). The vial

was then sealed with a rubber septum. Then the vial was placed onto an aluminum plate fitted with a 10 W high-power single LED plate (Avonec LED chip, size: 6.8 × 6.8 mm, λ_{max} = 280 nm), which is driven by a constant current power supply (22–36 V, 350 mA). Another plastic block was utilized to hold the quartz vial. The temperature was kept at approximately 30 °C through the use of a cooling fan. The reaction mixture was stirred and irradiated for 24 hours. After that, 2-fluorotoluene (11 µL, 0.10 mmol, 1.0 equiv.) was added as an internal standard, followed by 1 mL of saturated aqueous Na_2CO_3. An aliquot (0.1 mL) of the organic phase was taken and diluted with $CDCl_3$ (0.5 mL), and the yield was determined by ^{19}F NMR integration relative to the internal standard (30% yield, standard: δ –118.6 (m) ppm, **25**: δ –101.5 (t, *J* = 11.0 Hz) ppm, Figure S11). To isolate the fluorinated product, an identical reaction without added standard was transferred to a separatory funnel. The mixture was then diluted with 2 mL of ethyl acetate, the layers were separated, and the aqueous layer was extracted twice with ethyl acetate (approx. 2 mL each time). Then the combined organic layer was dried over Na_2SO_4, and the solvent was removed *in vacuo*. The residue was purified by flash column chromatography on silica gel eluting with hexanes/ethyl acetate (50:1 to 10:1 + 1% Et_3N, v:v:v) to afford a mixture. The mixture was further purified by normal phase HPLC (YMC PVA-Sil, 250 × 30.0 mm, 5 µm) with an eluent mixture of *i*-hexane/*i*-PrOH (98:2, v:v) at a flow rate of 20.0 mL/min to provide the title compounds **25** as a colorless oil.

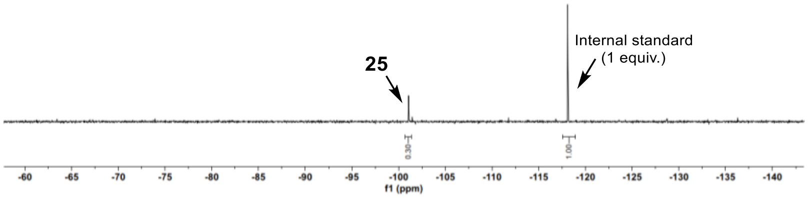

Figure S11. ^{19}F NMR of the reaction mixture for product **25**

R*f* = 0.30 (EtOAc/pentane, 10:1, v/v (UV))

NMR Spectroscopy:

^{1}H NMR (500 MHz, $CDCl_3$, 25 °C, δ): 9.02 (d, *J* = 10.1 Hz, 1H), 6.97 (d, *J* = 11.1 Hz, 1H), 3.95 (s, 3H), 2.62 (s, 3H).

^{13}C {^{1}H} NMR (126 MHz, $CDCl_3$, 25 °C, δ): 169.2, 167.0, 166.2 (d, *J* = 9.0 Hz), 163.5 (d, *J* = 3.1 Hz), 153.6, 111.9 (d, *J* = 17.6 Hz), 52.6, 24.7 (d, *J* = 2.8 Hz).

^{19}F NMR (471 MHz, $CDCl_3$, 25 °C, δ): –101.5 (brs).

HRMS GC-EI (m/z) calc'd for $C_8H_9NO_2F^+$ $[M]^+$, 169.0534; found, 169.0533. Deviation: +0.5 ppm.

Trichloroethyl 4-fluoro-2-methylnicotinate (26a) and trichloroethyl 6-fluoro-2-methylnicotinate (26b)

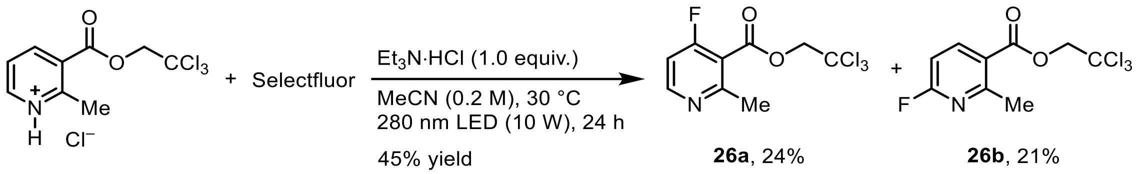

Under a nitrogen atmosphere, a 5-mL quartz vial equipped with a magnetic stri bar was charged with trichloroethyl 2-methylnicotinate hydrochloride (121.6 mg, 0.400 mmol, 1.00 equiv.), Selectfluor (566 mg, 1.60 mmol, 4.00 equiv.), Et_3N·HCl (54.8 mg, 0.400 mmol, 1.00 equiv.), and dry acetonitrile (2.0 mL, *c* = 0.2 M). The vial was then sealed with a rubber septum. Then the vial was placed onto an aluminum plate fitted with a 10 W high-power single LED plate (Avonec LED chip, size: 6.8 × 6.8 mm, λ_{max} = 280 nm), which is driven by a constant current power supply (22–36 V, 350 mA). Another plastic block was utilized to hold the quartz vial. The temperature was kept at approximately 30 °C through the use of a cooling fan. The reaction mixture was stirred and irradiated for 24 hours. After that, 2-fluorotoluene (11 µL, 0.10 mmol, 1.0 equiv.) was added as an internal standard, followed by 1 mL of saturated aqueous Na_2CO_3. An aliquot (0.1 mL) of the organic phase was taken and diluted with $CDCl_3$ (0.5 mL), and the yield was determined by ^{19}F NMR integration relative to the internal standard (45% yield, **26a**:**26b** = 1.1:1; standard: δ –118.6 (m) ppm, **26a**: δ –102.6 (t, *J* = 8.9 Hz) ppm, **26b**: δ –61.9 (d, *J* = 8.0 Hz) ppm, Figure S12). To isolate the fluorinated product, an identical reaction without added standard was transferred to a separatory funnel. The mixture was then diluted with 2 mL of ethyl acetate, the layers were separated, and the aqueous layer was extracted twice with ethyl acetate (approx. 2 mL each time). Then the combined organic layer was dried over Na_2SO_4, and the solvent was removed *in vacuo*. The residue was purified by flash column chromatography on silica gel eluting with hexanes/ethyl acetate (100:1 to 50:1 + 1% Et_3N, v:v:v) to afford the title compound **26b** as a colorless oil, and a mixture of **26a** and trichloroethyl 2-methylnicotinate. The mixture was further purified by normal phase HPLC (Multokrom 100-5 Si, 250 × 30.0 mm, 5 µm) with an eluent mixture of *i*-hexane/MTBE (90:10, v:v) at a flow rate of 20.0 mL/min to provide the title compounds **26a** as a colorless oil.

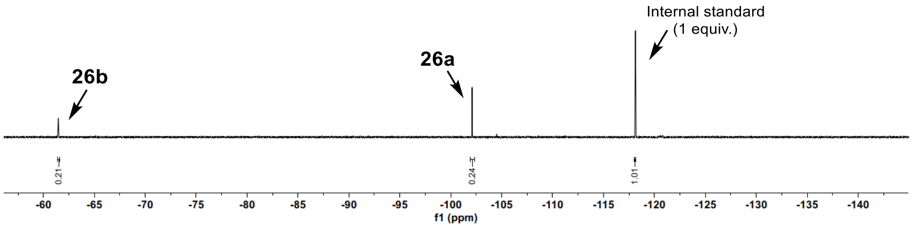

Figure S12. ^{19}F NMR of the reaction mixture for products **26a** and **26b**

Trichloroethyl 4-fluoro-2-methylnicotinate (**26a**)

R*f* = 0.30 (EtOAc/pentane, 10:1, v/v (UV))

NMR Spectroscopy:

^{1}H NMR (500 MHz, $CDCl_3$, 25 °C, δ): 8.59 (dd, *J* = 8.0, 5.7 Hz, 1H), 7.02 (dd, *J* = 9.2, 5.7 Hz, 1H), 5.03 (s, 2H), 2.75 (s, 3H).

^{13}C {^{1}H} NMR (126 MHz, $CDCl_3$, 25 °C, δ): 166.8 (d, *J* = 269.9 Hz), 162.9, 160.7, 153.20 (d, *J* = 8.5 Hz), 115.9 (d, *J* = 10.3 Hz), 109.8 (d, *J* = 17.4 Hz), 94.3, 74.8, 23.7 (d, *J* = 3.4 Hz).

^{19}F NMR (471 MHz, $CDCl_3$, 25 °C, δ): –102.6 (brs).

HRMS ESIpos (m/z) calc'd for $C_9H_8NO_2FCl_3^+$ [M+H]$^+$, 285.9599; found, 285.9599. Deviation: +1.2 ppm.

Trichloroethyl 6-fluoro-2-methylnicotinate (**26b**)

R*f* = 0.70 (EtOAc/pentane, 10:1, v/v (UV))

NMR Spectroscopy:

^{1}H NMR (500 MHz, $CDCl_3$, 25 °C, δ): 8.48 (t, *J* = 8.2 Hz, 1H), 6.91 (dd, *J* = 8.5, 3.4 Hz, 1H), 4.99 (s, 2H), 2.88 (s, 3H).

^{13}C {^{1}H} NMR (126 MHz, $CDCl_3$, 25 °C, δ): 164.4 (d, *J* = 246.9 Hz), 163.7, 162.2 (d, *J* = 15.9 Hz), 144.8 (d, *J* = 9.1 Hz), 121.9 (d, *J* = 4.6 Hz), 107.2 (d, *J* = 37.5 Hz), 94.9, 74.8, 24.9.

^{19}F NMR (471 MHz, $CDCl_3$, 25 °C, δ): –61.9 (d, *J* = 8.0 Hz).

HRMS ESIpos (m/z) calc'd for $C_9H_8NO_2FCl_3^+$ [M+H]$^+$, 285.9599; found, 285.9598. Deviation: +0.4 ppm.

Fluoroezetimibe-intermediate-A derivative 27

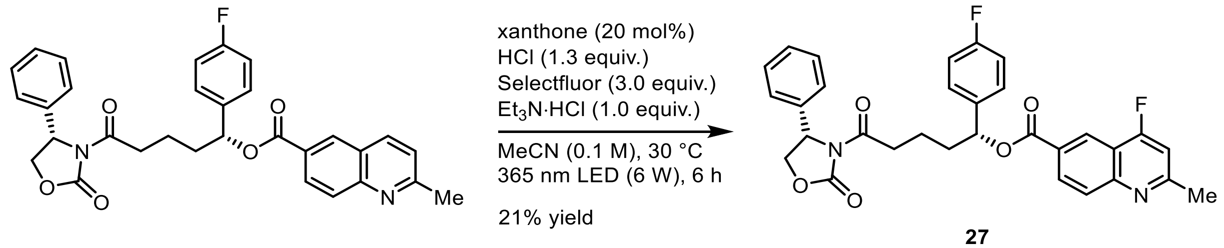

27

Under an ambient atmosphere, a 4-mL borosilicate vial equipped wit-h a magnetic stir bar was charged with ezetimibe intermediate A derivative (52.6 mg, 0.100 mmol, 1.00 equiv.), followed by dry DCM (1.0 mL, *c* = 0.1 M). Subsequently, 4.0 M HCl solution in dioxane (33 μL, 0.13 mmol, 1.3 equiv.) was added. The mixture was stirred at ambient temperature for 10 min. After that, the solvent was removed by a rotary evaporator. Under a nitrogen atmosphere, to the vial were then added xanthone (3.9 mg, 20 μmol, 20 mol%), Selectfluor (106.2 mg, 0.300 mmol, 3.00 equiv.), $Et_3N·HCl$ (13.7 mg, 0.100 mmol, 1.00 equiv.), and dry acetonitrile (1.0 mL, *c* = 0.1 M). The vial was sealed with a septum cap. Then the vial was placed onto an aluminum plate fitted with a 6 W high-power single LED plate (EpiLED Chip, size: 10 × 10 mm, λ_{max} = 365 nm, 600 mA, 9–11

V). Another aluminum block was utilized to hold the vial. The temperature was kept at approximately 30 °C through the use of a cooling fan. The reaction mixture was stirred and irradiated for 6 hours, then, 1 mL of saturated aqueous Na_2CO_3 were added. The mixture was then diluted with 2 mL of ethyl acetate, the layers were separated, and the aqueous layer was extracted twice with ethyl acetate (approx. 2 mL each time). Then the combined organic layer was dried over Na_2SO_4, and the solvent was removed *in vacuo*. The residue was purified by flash column chromatography on silica gel eluting with hexanes/ethyl acetate (from 50:1 to 1:1 + 1% Et_3N, v:v:v) to afford a mixture. The mixture was further purified by normal phase HPLC (YMC PVA-Sil, 250 × 30.0 mm, 5 µm) with an eluent mixture of *i*-hexane/2-propanol (90:10, v:v) at a flow rate of 42.5 mL/min to provide the title compound **27** (11.3 mg, 21 µmol, 21%) as a colorless oil.

R_f = 0.30 (EtOAc/pentane, 1:1, v/v (UV))

NMR Spectroscopy:

^{1}H NMR (500 MHz, $CDCl_3$, 25 °C, δ): 8.79 (d, *J* = 1.9 Hz, 1H), 8.33 (dd, *J* = 8.9, 2.0 Hz, 1H), 8.08 (dd, *J* = 8.9, 1.7 Hz, 1H), 7.46 – 7.29 (m, 7H), 7.09 – 7.02 (m, 3H), 6.01 (dd, *J* = 7.7, 6.0 Hz, 1H), 5.41 (dd, *J* = 8.7, 3.7 Hz, 1H), 4.68 (t, *J* = 8.9 Hz, 1H), 4.29 (dd, *J* = 9.0, 3.7 Hz, 1H), 3.04 (t, *J* = 7.3 Hz, 2H), 2.79 (s, 3H), 2.15 (dddd, *J* = 13.0, 10.6, 7.7, 5.1 Hz, 1H), 1.96 (ddt, *J* = 13.7, 11.1, 5.6 Hz, 1H), 1.85 – 1.75 (m, 1H), 1.73 – 1.63 (m, 1H).

^{13}C {^{1}H} NMR (126 MHz, $CDCl_3$, 25 °C, δ): 172.1, 166.0 (d, *J* = 270.6 Hz), 165.0, 162.5 (d, *J* = 260.8 Hz), 163.5 (d, *J* = 4.7 Hz), 153.7, 151.6 (d, *J* = 3.6 Hz), 139.1, 136.0 (d, *J* = 3.5 Hz), 130.3, 129.2, 128.8, 128.8 (d, *J* = 3.7 Hz), 128.4 (d, *J* = 8.3 Hz), 127.7, 126.0, 123.8 (d, *J* = 4.7 Hz), 117.4 (d, *J* = 13.7 Hz), 115.6 (d, *J* = 21.6 Hz), 107.0 (d, *J* = 14.3 Hz), 76.3, 70.1, 57.6, 35.6, 35.2, 25.9 (d, *J* = 2.9 Hz), 20.2.

^{19}F NMR (471 MHz, $CDCl_3$, 25 °C, δ): –112.9 (brs, 1F), –114.8 (m, 1F).

HRMS ESIpos (m/z) calc'd for $C_{31}H_{27}N_2F_2O_5^+$ [M+H]$^+$, 545.1882; found, 545.1882. Deviation: 0.0 ppm.

Fluoroflorfenicol derivative 28

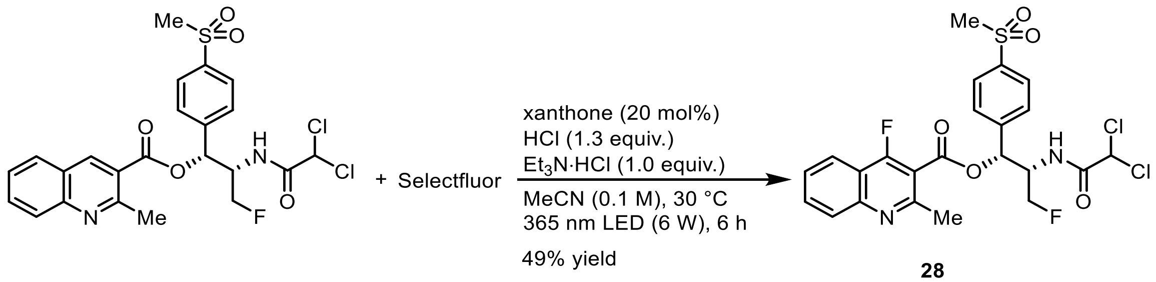

28

Under an ambient atmosphere, a 4-mL borosilicate vial equipped with a magnetic stir bar was charged with florfenicol derivative (52.7 mg, 0.100 mmol, 1.00 equiv.), followed by dry DCM (1.0 mL, *c* = 0.1 M). Subsequently, 4.0 M HCl solution in dioxane (33 µL, 0.13 mmol, 1.3 equiv.) was added. The mixture was stirred at ambient temperature for 10 min. After that, the solvent was removed by a rotary evaporator. Under a nitrogen atmosphere, to the vial were then added xanthone (3.9 mg, 20 µmol, 20 mol%), Selectfluor (106.2

mg, 0.300 mmol, 3.00 equiv.), $Et_3N{\cdot}HCl$ (13.7 mg, 0.100 mmol, 1.00 equiv.), and dry acetonitrile (1.0 mL, *c* = 0.1 M). The vial was sealed with a septum cap. Then the vial was placed onto an aluminum plate fitted with a 6 W high-power single LED plate (EpiLED Chip, size: 10 × 10 mm, λ_{max} = 365 nm, 600 mA, 9–11 V). Another aluminum block was utilized to hold the vial. The temperature was kept at approximately 30 °C through the use of a cooling fan. The reaction mixture was stirred and irradiated for 6 hours, then, 1 mL of saturated aqueous Na_2CO_3 were added. The mixture was then diluted by 2 mL of ethyl acetate, the layers were separated, and the aqueous layer was extracted twice with ethyl acetate (approx. 2 mL each time). Then the combined organic layer was dried over Na_2SO_4, and the solvent was removed *in vacuo*. The residue was purified by flash column chromatography on silica gel eluting with hexanes/ethyl acetate (from 50:1 to 1:1 + 1% Et_3N, v:v:v) to obtain a mixture. The mixture was further purified by normal phase HPLC (YMC PVA-Sil, 250 × 30.0 mm, 5 µm) with an eluent mixture of *i*-hexane/2-propanol (70:30, v:v) at a flow rate of 42.5 mL/min to provide the title compound **28** (26.6 mg, 49 µmol, 49%) as a colorless oil.

$\mathbf{R_f}$ = 0.35 (pentane/EtOAc, 1:1, v/v (UV))

NMR Spectroscopy:

^{1}H NMR (500 MHz, $CDCl_3$, 25 °C, δ): 8.14 (d, *J* = 8.3 Hz, 1H), 8.08 (d, *J* = 8.6 Hz, 1H), 8.06 – 8.03 (m, 2H), 7.88 (ddd, *J* = 8.5, 6.9, 1.4 Hz, 1H), 7.75 (d, *J* = 8.4 Hz, 2H), 7.66 (ddd, *J* = 8.1, 6.9, 1.1 Hz, 1H), 7.10 (d, *J* = 9.3 Hz, 1H), 6.50 (d, *J* = 5.9 Hz, 1H), 5.94 (s, 1H), 4.80 – 4.42 (m, 3H), 3.09 (s, 3H), 2.80 (s, 3H).

^{13}C {^{1}H} NMR (126 MHz, $CDCl_3$, 25 °C, δ): 164.4 (d, *J* = 277.6 Hz), 164.2, 163.4, 158.3 (d, *J* = 3.3 Hz), 149.8, 141.8, 141.4, 132.9 (d, *J* = 4.7 Hz), 128.4, 128.2, 127.9, 127.4 (d, *J* = 3.6 Hz), 121.3 (d, *J* = 5.8 Hz), 117.2 (d, *J* = 14.5 Hz), 110.5 (d, *J* = 7.1 Hz), 81.4 (d, *J* = 174.4 Hz), 74.2 (d, *J* = 3.6 Hz), 66.0, 53.9 (d, *J* = 19.9 Hz), 44.5, 24.6 (d, *J* = 3.8 Hz).

^{19}F NMR (471 MHz, $CDCl_3$, 25 °C, δ): –110.6 (s, 1F), –232.2 (td, *J* = 46.7, 23.4 Hz).

HRMS ESIpos (m/z) calc'd for $C_{23}H_{21}N_2F_2Cl_2O_5S^+$ $[M+H]^+$, 545.0511; found, 545.0507. Deviation: –0.8 ppm.

Table S2. Other tested substrates for photo-initiated conditions

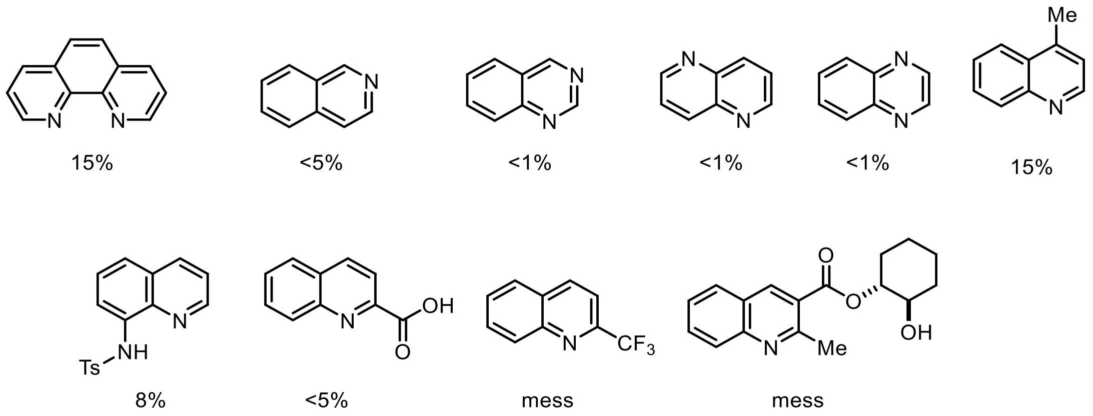

Discussion:

1. C2-selectivity was observed when C4 was blocked. For example, when 4-methylquinoline was employed as substrate, 2-fluoro-4-methylquinoline was observed in 15% yield;

2. In the case of CF_3 substituted quinoline, the reaction mixture is messy. The lack of success in this case may be attributed to an alternative reaction pathway for the *N*-heterocyclic π-radical, which could undergo a spin center shift to the CF_3 group, resulting in side reactions[5].

2-Chloro-4-fluoronicotinic amide 29

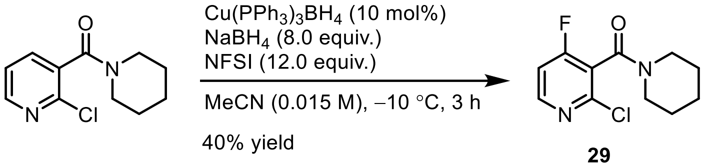

29

entry	Change of reaction conditions	yield of product[b]
1	None	40%
2	BH_3·THF (1.3 equiv.), then Selectfluor (3 equiv.)[c]	8%
3	BH_3·THF (1.3 equiv.), then NFSI (3 equiv.)[c]	14%
4	$NaBH_4$ (2 equiv.), NFSI (4 equiv.)[c]	18%
5	$LiBH_4$ (2 equiv.), NFSI (4 equiv.)[c]	12%
6	HBpin (3 equiv.), NFSI (3 equiv.)[c]	<1%
7	HBcat (1 equiv.), NFSI (3 equiv.)[c]	0%
8	9-BBN dimer (1 equiv.), NFSI (3 equiv.)[c]	0%
9	$NaBH_3CN$ (2 equiv.), NFSI (3 equiv.)[c]	0%
10	$NaBH(OAc)_3$ (2 equiv.), NFSI (3 equiv.)[c]	0%

[a] 2-Chloro-nicotinic amide (0.100 mmol, 1.00 equiv.), $Cu(PPh_3)_3BH_4$ (10 mol%), $NaBH_4$ (8.00 equiv.), NFSI (12.0 equiv.), MeCN (0.015 M), –10 °C, 3 h. [b] NMR yield with 2-fluorotoluene as internal standard. [c] Cu(OAc) (10 mol%), MeCN (0.05 M), –10 °C, 3 h.

Discussion:

The $NaBH_4$//NFSI conditions have the potential to be developed into a C4-selective fluorination reaction of pyridine. However, the high loading of fluorinating reagents poses a challenge in their practical synthetic applications now.

Under inert atmosphere, to a 20-mL vial equipped with a magnetic stir bar were added 2-chloronicotinic amide (22.4 mg, 0.100 mmol, 1.00 equiv.), $Cu(PPh_3)_2BH_4$ (6.0 mg, 10 μmol, 10 mol%), $NaBH_4$ (30.2 mg, 0.800 mmol, 8.00 equiv.), and dry acetonitrile (1.0 mL). The vial was cooled to –10 °C. A solution of *N*-fluorobenzenesulfonimide (378 mg, 1.20 mmol, 12.0 equiv.) in MeCN (6.0 mL) was then added dropwise to

the reaction mixture (caution: gas evolution). The reaction mixture was stirred at –10 °C for 3 h, then warmed to room temperature. After that, 2-fluorotoluene (11 μL, 0.10 mmol, 1.0 equiv.) was added as an internal standard, followed by 1 mL of saturated aqueous Na_2CO_3. An aliquot (ca. 0.1 mL) of the organic phase was taken and diluted with $CDCl_3$ (0.5 mL), and the yield was determined by ^{19}F NMR integration relative to the internal standard (40% yield; standard: δ –118.6 (m) ppm, **29**: δ –102.9 (t, *J* = 8.2 Hz) ppm, Figure S13). To isolate the fluorinated product, an identical reaction without added standard was transferred to a separatory funnel. The mixture was then diluted by 2 mL of ethyl acetate, the layers were separated, and the aqueous layer was extracted twice with ethyl acetate (approx. 2 mL each time). Then the combined organic layer was dried over Na_2SO_4, and the solvent was removed *in vacuo*. The residue was then purified by flash column chromatography on silica gel eluting with hexanes/ethyl acetate (10:1 + 1% Et_3N, v:v:v) to afford the titled compound as a colorless oil.

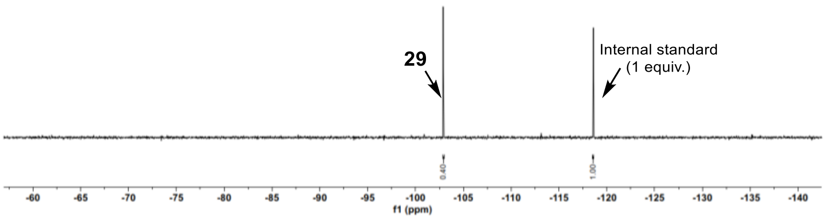

Figure S13. ^{19}F NMR of the crude mixture for product **29**

R*f* = 0.40 (DCM/EtOAc, 4:1, v/v (UV))

NMR Spectroscopy:

^1H NMR (500 MHz, $CDCl_3$, 25 °C, δ): 8.32 (dd, *J* = 8.1, 5.6 Hz, 1H), 7.00 (dd, *J* = 7.7, 5.7 Hz, 1H), 3.71 (dt, *J* = 6.1, 3.2 Hz, 2H), 3.21 – 3.11 (m, 2H), 1.67 – 1.44 (m, 6H).

^{13}C {^1H} NMR (151 MHz, $CDCl_3$, 25 °C, δ): 165.3 (d, *J* = 265.7 Hz), 160.0, 151.3 (d, *J* = 8.0 Hz), 149.5 (d, *J* = 6.3 Hz), 121.80 (d, *J* = 19.5 Hz), 111.32 (d, *J* = 17.4 Hz), 48.0, 43.0, 26.5, 25.5, 24.6.

^{19}F NMR (565 MHz, $CDCl_3$, 25 °C, δ): –102.9 (t, *J* = 7.8 Hz).

HRMS GC/CI isobutan (m/z) calc'd for $C_{11}H_{13}N_2OFCl^+$ $[M]^+$, 243.0694; found, 243.0697. Deviation: –0.8 ppm.

Preparation of starting materials

Chloroalkyl 2-methylquinoline-3-carboxylate S7

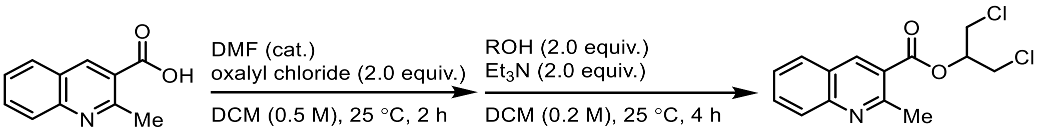

S7, 78%

Under an ambient atmosphere, a 25-mL round bottom flask equipped with a magnetic stir bar was charged

with 2-methylquinoline-3-carboxylic acid (374 mg, 2.00 mmol, 1.00 equiv.), followed by dry DCM (4.0 mL, *c* = 0.5 M). Subsequently, one drop of DMF followed by oxalyl chloride (343 μL, 507 mg, 4.00 mmol, 2.00 equiv.) were added to the flask. The reaction was stirred at 25 °C until gas evolution ceased (ca. 2 hours). The volatiles were removed by a rotary evaporator, then the residue was re-dissolved in dry CH_2Cl_2 (10.0 mL, *c* = 0.2 M). After that, 1,3-dichloropropan-2-ol (368 μL, 512 mg, 4.00 mmol, 2.00 equiv.), and Et_3N (557 μL, 404 mg, 4.00 mmol, 2.00 equiv.) were added. The reaction was stirred at 25 °C for 4 hours. The reaction was quenched with 10 mL water, the layers were separated, and the aqueous layer was extracted twice with CH_2Cl_2 (10 mL each time). Then the combined organic layer was dried over Na_2SO_4, and the solvent was removed *in vacuo*. The residue was then purified by flash column chromatography on silica gel eluting with hexanes/ethyl acetate (20:1 + 1% Et_3N, v:v:v) to obtain the title compound (**S7**, 464 mg, 78%) as a colorless solid.

m. p. = 90 – 92 °C

R_f = 0.40 (pentane/EtOAc, 5:1, v/v (UV))

NMR Spectroscopy:

^{1}H NMR (500 MHz, $CDCl_3$, 25 °C, δ): 8.73 (s, 1H), 7.98 (d, *J* = 8.5 Hz, 1H), 7.83 (dd, *J* = 8.2, 1.4 Hz, 1H), 7.74 (ddd, *J* = 8.4, 6.9, 1.5 Hz, 1H), 7.50 (ddd, *J* = 8.0, 6.9, 1.1 Hz, 1H), 5.43 (p, *J* = 5.1 Hz, 1H), 3.87 (d, *J* = 5.2 Hz, 4H), 2.94 (s, 3H).

^{13}C {^{1}H} NMR (126 MHz, $CDCl_3$, 25 °C, δ): 165.2, 158.5, 148.9, 140.7, 132.2, 128.7, 128.6, 126.8, 125.6, 122.5, 72.6, 42.5, 25.8.

HRMS GC-EI (m/z) calc'd for $C_{14}H_{13}NO_2Cl_2^+$ $[M]^+$, 297.0317; found, 297.0321. Deviation: –1.1 ppm.

Polyfluoroalkyl 2-methylquinoline-6-carboxylate S10

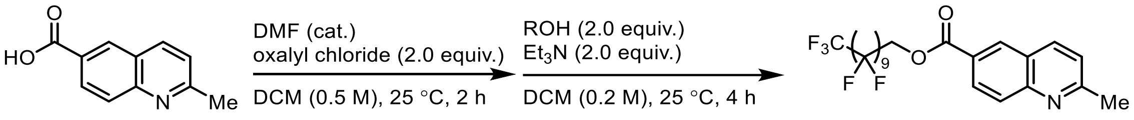

S10, 56%

Under an ambient atmosphere, a 25-mL round bottom flask equipped with a magnetic stir bar was charged with 2-methylquinoline-6-carboxylic acid (187 mg, 1.00 mmol, 1.00 equiv.), followed by dry DCM (2.0 mL, *c* = 0.5 M). Subsequently, one drop of DMF followed by oxalyl chloride (172 μL, 253 mg, 2.00 mmol, 2.00 equiv.) were added to the flask. The reaction was stirred at 25 °C until gas evolution ceased (ca. 2 hours). The volatiles were removed by a rotary evaporator, then the residue was re-dissolved in dry CH_2Cl_2 (5.0 mL, *c* = 0.2 M). After that, heneicosafluoro-1-undecanol (1100 mg, 2.00 mmol, 2.00 equiv.), and Et_3N (278 μL, 202 mg, 2.00 mmol, 2.00 equiv.) were added. The reaction was stirred at 25 °C for 4 hours. The reaction was quenched with 5 mL water, the layers were separated, and the aqueous layer was extracted twice with CH_2Cl_2 (5 mL each time). Then the combined organic layer was dried over Na_2SO_4, and the solvent was removed *in vacuo*. The residue was then purified by flash column chromatography on silica gel eluting with

hexanes/ethyl acetate (10:1 + 1% Et_3N, v:v:v) to obtain the title compound (**S10**, 405 mg, 56%) as a colorless solid.

m. p. = 100 – 102 °C

R*f* = 0.30 (pentane/EtOAc, 5:1, v/v (UV))

NMR Spectroscopy:

^{1}H NMR (500 MHz, $CDCl_3$, 25 °C, δ): 8.61 (d, *J* = 1.9 Hz, 1H), 8.31 (dd, *J* = 8.9, 1.9 Hz, 1H), 8.23 (d, *J* = 8.4 Hz, 1H), 8.17 (d, *J* = 8.9 Hz, 1H), 7.43 (d, *J* = 8.4 Hz, 1H), 4.92 (t, *J* = 13.2 Hz, 2H), 2.84 (s, 3H).

^{13}C {^{1}H} NMR (151 MHz, $CDCl_3$, 25 °C, δ): 164.7, 162.3, 149.7, 138.2, 131.7, 129.4, 129.1, 125.8, 125.4,

118.7 – 107.1 (m, 10C), 60.5 (t, *J* = 27.4 Hz), 25.5.

^{19}F NMR (471 MHz, $CDCl_3$, 25 °C, δ): –80.7 (t, *J* = 9.9 Hz, 3F), –119.2 (m, 2F), –121.7 (m, 6F), –121.8 (m, 4F), –122.6 (m, 2F), –123.1 (m, 2F), 126.1 (m, 2F).

HRMS ESIpos (m/z) calc'd for $C_{22}H_{11}NF_{21}O_2^+$ [M+H]$^+$, 720.0449; found, 720.0450. Deviation: –0.2 ppm.

3-(Phthalimidyl)propyl 2-methylquinoline-3-carboxylate (S14)

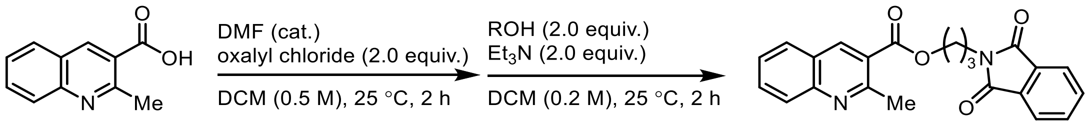

S14, 70%

Under an ambient atmosphere, a 25-mL round bottom flask equipped with a magnetic stir bar was charged with 2-methylquinoline-3-carboxylic acid (374 mg, 2.00 mmol, 1.00 equiv.), followed by dry DCM (4.0 mL, *c* = 0.5 M). Subsequently, one drop of DMF followed by oxalyl chloride (343 µL, 507 mg, 4.00 mmol, 2.00 equiv.) were added to the flask. The reaction was stirred at 25 °C until gas evolution ceased (ca. 2 hours). The volatiles were removed by a rotary evaporator, then the residue was re-dissolved in dry CH_2Cl_2 (10.0 mL, *c* = 0.2 M). After that, *N*-(3-hydroxypropyl)phthalimide (820 mg, 4.00 mmol, 2.00 equiv.), and Et_3N (557 µL, 404 mg, 4.00 mmol, 2.00 equiv.) were added. The reaction was stirred at 25 °C for 2 hours. The reaction was quenched with 10 mL water, the layers were separated, and the aqueous layer was extracted twice with CH_2Cl_2 (10 mL each time). Then the combined organic layer was dried over Na_2SO_4, and the solvent was removed *in vacuo*. The residue was then purified by flash column chromatography on silica gel eluting with hexanes/ethyl acetate (5:1 + 1% Et_3N, v:v:v) to obtain the title compound (**S14**, 523 mg, 70%) as a colorless solid.

m. p. = 107 – 109 °C

R*f* = 0.30 (pentane/EtOAc, 2:1, v/v (UV))

NMR Spectroscopy:

^{1}H NMR (500 MHz, $CDCl_3$, 25 °C, δ): 8.66 (s, 1H), 8.03 (dd, *J* = 8.4, 1.2 Hz, 1H), 7.83 – 7.76 (m, 2H), 7.74 (dd, *J* = 5.4, 3.1 Hz, 2H), 7.56 (dd, *J* = 5.5, 3.0 Hz, 2H), 7.55 – 7.51 (m, 1H), 4.45 (t, *J* = 6.0 Hz, 2H), 3.95 (t, *J* = 6.6 Hz, 2H), 2.99 (s, 3H), 2.26 (p, *J* = 6.4 Hz, 2H).

^{13}C {^{1}H} NMR (126 MHz, $CDCl_3$, 25 °C, δ): 168.3, 166.3, 158.4, 148.6, 139.6, 133.9, 131.9, 131.7, 128.6, 128.5, 126.4, 125.7, 123.6, 123.1, 63.1, 35.4, 27.5, 25.5.

HRMS GC-EI (m/z) calc'd for $C_{22}H_{18}NO_4^+$ $[M]^+$, 374.1261; found, 374.1264. Deviation: –0.8 ppm.

2-Cyanoethyl 2-methylquinoline-3-carboxylate (S16)

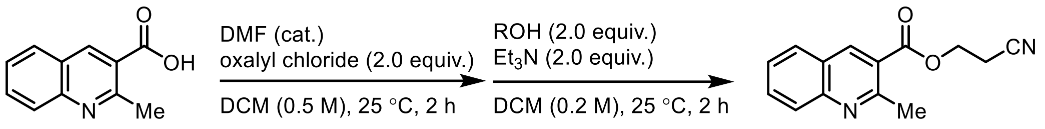

S16, 63%

Under an ambient atmosphere, a 25-mL round bottom flask equipped with a magnetic stir bar was charged with 2-methylquinoline-3-carboxylic acid (374 mg, 2.00 mmol, 1.00 equiv.), followed by dry DCM (4.0 mL, *c* = 0.5 M). Subsequently, one drop of DMF followed by oxalyl chloride (343 μL, 507 mg, 4.00 mmol, 2.00 equiv.) were added to the flask. The reaction was stirred at 25 °C until gas evolution ceased (ca. 2 hours). The volatiles were removed by a rotary evaporator, then the residue was re-dissolved in dry CH_2Cl_2 (10.0 mL, *c* = 0.2 M). After that, 3-hydroxypropionitrile (284 mg, 4.00 mmol, 2.00 equiv.), and Et_3N (557 μL, 404 mg, 4.00 mmol, 2.00 equiv.) were added. The reaction was stirred at 25 °C for 2 hours. The reaction was quenched with 10 mL water, the layers were separated, and the aqueous layer was extracted twice with CH_2Cl_2 (10 mL each time). Then the combined organic layers were dried over Na_2SO_4, and the solvent was removed *in vacuo*. The residue was then purified by flash column chromatography on silica gel eluting with hexanes/ethyl acetate (5:1 + 1% Et_3N, v:v:v) to obtain the title compound (**S16**, 301 mg, 63%) as a colorless solid.

m. p. = 194 – 197 °C

R_f = 0.30 (pentane/EtOAc, 2:1, v/v (UV))

NMR Spectroscopy:

^{1}H NMR (500 MHz, $CDCl_3$, 25 °C, δ): 8.84 (s, 1H), 8.08 (d, *J* = 8.5 Hz, 1H), 7.92 (dd, *J* = 8.3, 1.4 Hz, 1H), 7.84 (ddd, *J* = 8.5, 6.9, 1.5 Hz, 1H), 7.59 (ddd, *J* = 8.1, 6.8, 1.1 Hz, 1H), 4.62 (t, *J* = 6.2 Hz, 2H), 3.04 (s, 3H), 2.93 (t, *J* = 6.2 Hz, 2H).

^{13}C {^{1}H} NMR (126 MHz, $CDCl_3$, 25 °C, δ): 165.7, 158.5, 149.0, 140.7, 132.3, 128.7, 128.6, 126.8, 125.6, 122.3, 116.8, 59.5, 25.7, 18.2.

HRMS GC-EI (m/z) calc'd for $C_{14}H_{12}N_2O_2^+$ $[M]^+$, 240.0893; found, 240.0897. Deviation: –1.5 ppm.

2-Bromoethyl 2-methylquinoline-3-carboxylate (S17)

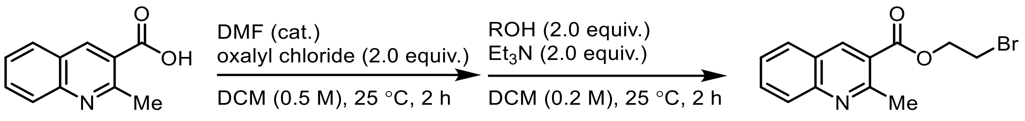

S17, 64%

Under an ambient atmosphere, a 25-mL round bottom flask equipped with a magnetic stir bar was charged with 2-methylquinoline-3-carboxylic acid (374 mg, 2.00 mmol, 1.00 equiv.), followed by dry DCM (4.0 mL, *c* = 0.5 M). Subsequently, one drop of DMF followed by oxalyl chloride (343 µL, 507 mg, 4.00 mmol, 2.00 equiv.) were added to the flask. The reaction was stirred at 25 °C until gas evolution ceased (ca. 2 hours). The volatiles were removed by a rotary evaporator, then the residue was re-dissolved in dry CH_2Cl_2 (10.0 mL, *c* = 0.2 M). After that, 2-bromoethanol (496 mg, 4.00 mmol, 2.00 equiv.), and Et_3N (557 µL, 404 mg, 4.00 mmol, 2.00 equiv.) were added. The reaction was stirred at 25 °C for 2 hours. The reaction was quenched with 10 mL water, the layers were separated, and the aqueous layer was extracted twice with CH_2Cl_2 (10 mL each time). Then the combined organic layers were dried over Na_2SO_4, and the solvent was removed *in vacuo*. The residue was then purified by flash column chromatography on silica gel eluting with hexanes/ethyl acetate (from 20:1 to 10:1 + 1% Et_3N, v:v:v) to obtain the title compound (**S17**, 380 mg, 64%) as a colorless oil.

R*f* = 0.30 (pentane/EtOAc, 5:1, v/v (UV))

NMR Spectroscopy:

^{1}H NMR (500 MHz, $CDCl_3$, 25 °C, δ): 8.83 (s, 1H), 8.07 (dd, *J* = 8.5, 1.1 Hz, 1H), 7.91 (dd, *J* = 8.2, 1.4 Hz, 1H), 7.82 (ddd, *J* = 8.4, 6.9, 1.5 Hz, 1H), 7.58 (ddd, *J* = 8.0, 6.9, 1.1 Hz, 1H), 4.72 (t, *J* = 5.9 Hz, 2H), 3.73 (t, *J* = 5.9 Hz, 2H), 3.04 (s, 3H).

^{13}C {^{1}H} NMR (126 MHz, $CDCl_3$, 25 °C, δ): 165.9, 158.5, 148.8, 140.5, 132.0, 128.6, 128.6, 126.7, 125.7, 123.0, 64.6, 28.8, 25.8.

HRMS GC-EI (m/z) calc'd for $C_{13}H_{12}NO_2Br^+$ $[M]^+$, 293.0046 found, 293.0048. Deviation: –0.6 ppm.

Phosphorylmethyl 2-methylquinoline-3-carboxylate S18

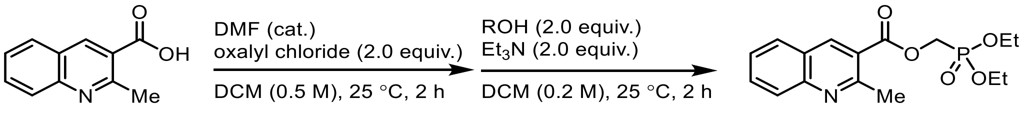

S18, 73%

Under an ambient atmosphere, a 25-mL round bottom flask equipped with a magnetic stir bar was charged with 2-methylquinoline-3-carboxylic acid (374 mg, 2.00 mmol, 1.00 equiv.), followed by dry DCM (4.0 mL, *c* = 0.5 M). Subsequently, one drop of DMF followed by oxalyl chloride (343 µL, 507 mg, 4.00 mmol, 2.00 equiv.) were added to the flask. The reaction was stirred at 25 °C until gas evolution ceased (ca. 2 hours). The

volatiles were removed by a rotary evaporator, then the residue was re-dissolved in dry CH_2Cl_2 (10.0 mL, *c* = 0.2 M). After that, diethyl (hydroxymethyl)phosphonates (672 mg, 4.00 mmol, 2.00 equiv.), and Et_3N (557 µL, 404 mg, 4.00 mmol, 2.00 equiv.) were added. The reaction was stirred at 25 °C for 2 hours. The reaction was quenched with 10 mL water, the layers were separated, and the aqueous layer was extracted twice with CH_2Cl_2 (10 mL each time). Then the combined organic layers were dried over Na_2SO_4, and the solvent was removed *in vacuo*. The residue was then purified by flash column chromatography on silica gel eluting with hexanes/ethyl acetate (2:1 + 1% Et_3N, v:v:v) to obtain the title compound (**S18**, 490 mg, 73%) as a colorless oil.

R*f* = 0.30 (pentane/EtOAc, 1:1, v/v (UV))

NMR Spectroscopy:

^{1}H NMR (500 MHz, $CDCl_3$, 25 °C, δ): 8.82 (s, 1H), 8.08 (d, *J* = 8.5 Hz, 1H), 7.90 (dd, *J* = 8.2, 1.4 Hz, 1H), 7.83 (ddd, *J* = 8.5, 6.9, 1.5 Hz, 1H), 7.59 (ddd, *J* = 8.1, 6.8, 1.2 Hz, 1H), 4.70 (d, *J* = 8.6 Hz, 2H), 4.27 (dq, *J* = 8.3, 7.1 Hz, 4H), 3.03 (s, 3H), 1.40 (t, *J* = 7.1 Hz, 6H).

^{13}C {^{1}H} NMR (126 MHz, $CDCl_3$, 25 °C, δ): 165.4 (d, *J* = 8.3 Hz), 158.5, 148.8, 140.6, 132.2, 128.6, 128.6, 126.8, 125.6, 122.6, 62.9 (d, *J* = 6.4 Hz), 57.4 (d, *J* = 169.1 Hz), 25.6, 16.5 (d, *J* = 5.9 Hz).

^{31}P NMR (203 MHz, $CDCl_3$, 25 °C, δ): 18.9.

HRMS GC-EI (m/z) calc'd for $C_{16}H_{20}NO_5P^+$ $[M]^+$, 337.1074; found, 337.1079. Deviation: –1.5 ppm.

7-Sulfonamidylquinoline S19

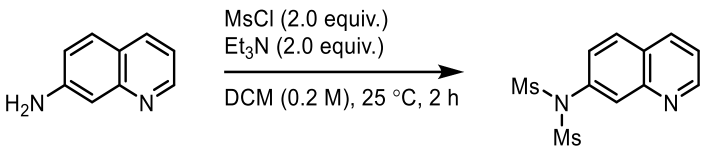

S19, 80%

Under an ambient atmosphere, a 25-mL round bottom flask equipped with a magnetic stir bar was charged with 7-aminoquinoline (288 mg, 2.00 mmol, 1.00 equiv.), followed by dry DCM (10 mL, *c* = 0.2 M). After that, mesyl chloride (456 mg, 4.00 mmol, 2.00 equiv.), and Et_3N (557 µL, 404 mg, 4.00 mmol, 2.00 equiv.) were added. The reaction was stirred at 25 °C for 2 hours. The reaction was quenched with 10 mL water, the layers were separated, and the aqueous layer was extracted twice with CH_2Cl_2 (10 mL each time). Then the combined organic layers were dried over Na_2SO_4, and the solvent was removed *in vacuo*. The residue was then purified by flash column chromatography on silica gel eluting with hexanes/ethyl acetate (2:1 + 1% Et_3N, v:v:v) to obtain the title compound (**S19**, 480 mg, 80%) as a colorless solid.

m. p. = 207 – 209 °C

R*f* = 0.30 (pentane/EtOAc, 1:1, v/v (UV))

NMR Spectroscopy:

^{1}H NMR (500 MHz, $CDCl_3$, 25 °C, δ): 9.01 (dd, *J* = 4.3, 1.7 Hz, 1H), 8.28 (dt, *J* = 8.1, 1.4 Hz, 1H), 8.24 (d, *J* = 2.1 Hz, 1H), 7.95 (d, *J* = 8.6 Hz, 1H), 7.61 – 7.47 (m, 2H), 3.49 (s, 6H).

^{13}C {^{1}H} NMR (126 MHz, $CDCl_3$, 25 °C, δ): 151.3, 147.4, 136.8, 134.6, 131.5, 129.6, 129.1, 128.7, 122.8, 43.1.

HRMS ESIpos (m/z) calc'd for $C_{11}H_{13}N_2O_4S_2^+$ $[M+H]^+$, 301.0311; found, 301.0314. Deviation: –1.0 ppm.

6-Sulfonyloxyquinoline S20

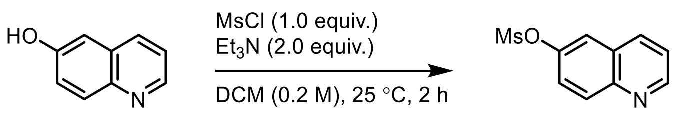

S20, 93%

Under an ambient atmosphere, a 25-mL round bottom flask equipped with a magnetic stir bar was charged with 6-hydroxylquinoline (288 mg, 2.00 mmol, 1.00 equiv.), followed by dry DCM (4.0 mL, *c* = 0.5 M). followed by dry DCM (10 mL, *c* = 0.2 M). After that, mesyl chloride (228 mg, 2.00 mmol, 1.00 equiv.), and Et_3N (557 µL, 404 mg, 4.00 mmol, 2.00 equiv.) were added. The reaction was stirred at 25 °C for 2 hours. The reaction was quenched with 10 mL water, the layers were separated, and the aqueous layer was extracted twice with CH_2Cl_2 (10 mL each time). Then the combined organic layers were dried over Na_2SO_4, and the solvent was removed *in vacuo*. The residue was then purified by flash column chromatography on silica gel eluting with hexanes/ethyl acetate (4:1 + 1% Et_3N, v:v:v) to obtain the title compound (**S20**, 414 mg, 93%) as a colorless solid.

m. p. = 87 – 88 °C

R_f = 0.40 (pentane/EtOAc, 1:1, v/v (UV))

NMR Spectroscopy:

^{1}H NMR (500 MHz, $CDCl_3$, 25 °C, δ): 8.97 (d, *J* = 4.1 Hz, 1H), 8.27 – 8.13 (m, 2H), 7.80 (d, *J* = 2.7 Hz, 1H), 7.65 (dd, *J* = 9.2, 2.7 Hz, 1H), 7.49 (dd, *J* = 8.2, 4.2 Hz, 1H), 3.25 (s, 3H).

^{13}C {^{1}H} NMR (126 MHz, $CDCl_3$, 25 °C, δ): 151.0, 146.9, 146.7, 136.3, 132.0, 128.6, 124.5, 122.2, 119.6, 37.9.

HRMS GC-EI (m/z) calc'd for $C_{10}H_9NO_3S^+$ $[M]^+$, 223.0297; found, 223.0299. Deviation: –0.5 ppm.

Methyl 4-(2-methylquinolin-6-yl)benzoate (S22)

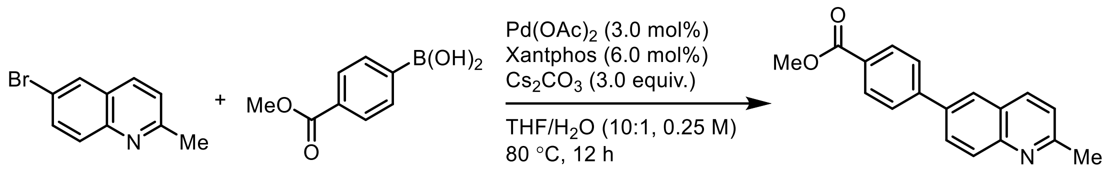

S22, 73%

Under a nitrogen atmosphere, a 20-mL borosilicate equipped with a magnetic stir bar was charged with 6-bromo-2-methylquinoline (444 mg, 2.00 mmol, 1.00 equiv.), 4-methoxycarbonylphenylboronic (364 mg, 2.02 mmol, 1.01 equiv.), palladium acetate (13.5 mg, 0.06 mmol, 3 mol%), Xantphos (69.4 mg, 0.12 mmol, 6 mol%), cesium carbonate (1.95 g, 6.00 mmol, 3.00 equiv.), followed by dry THF (7.3 mL) and water (0.7 mL). The reaction was stirred at 80 °C for 12 hours. The reaction mixture was then cooled to room temperature, the layers were separated, and the aqueous layer was extracted twice with ethyl acetate (10 mL each time). Then the combined organic layers were dried over Na_2SO_4, and the solvent was removed *in vacuo*. The residue was then purified by flash column chromatography on silica gel eluting with hexanes/ethyl acetate (10:1 to 3:1 + 1% Et_3N, v:v:v) to obtain the title compound (**S22**, 404 mg, 73%) as a colorless solid.

m. p. = 110 – 112 °C

R*f* = 0.25 (pentane/EtOAc, 5:1, v/v (UV))

NMR Spectroscopy:

^{1}H NMR (500 MHz, $CDCl_3$, 25 °C, δ): 8.17 – 8.11 (m, 4H), 8.02 (d, *J* = 2.1 Hz, 1H), 7.97 (dd, *J* = 8.7, 2.1 Hz, 1H), 7.82 – 7.75 (m, 2H), 7.35 (d, *J* = 8.4 Hz, 1H), 3.96 (s, 3H), 2.79 (s, 3H).

^{13}C {^{1}H} NMR (126 MHz, $CDCl_3$, 25 °C, δ): 167.0, 159.6, 147.3, 144.9, 137.4, 136.9, 130.4, 129.3, 129.2, 129.1, 127.4, 126.7, 125.9, 122.8, 52.3, 25.4.

HRMS GC-EI (m/z) calc'd for $C_{18}H_{15}NO_2^+$ $[M]^+$, 277.1097; found, 277.1103. Deviation: –2.0 ppm.

8-Sulfonyloxyquinoline S23

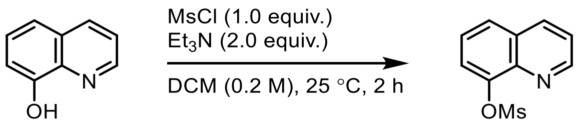

S23, 95%

Under an ambient atmosphere, a 25-mL round bottom flask equipped with a magnetic stir bar was charged with 8-hydroxylquinoline (288 mg, 2.00 mmol, 1.00 equiv.), followed by dry DCM (4.0 mL, *c* = 0.5 M). followed by dry DCM (10 mL, *c* = 0.2 M). After that, mesyl chloride (228 mg, 2.00 mmol, 1.00 equiv.), and Et_3N (557 µL, 404 mg, 4.00 mmol, 2.00 equiv.) were added. The reaction was stirred at 25 °C for 2 hours. The reaction was quenched with 10 mL water, the layers were separated, and the aqueous layer was extracted

twice with CH_2Cl_2 (10 mL each time). Then the combined organic layers were dried over Na_2SO_4, and the solvent was removed *in vacuo*. The residue was then purified by flash column chromatography on silica gel eluting with hexanes/ethyl acetate (4:1 + 1% Et_3N, v:v:v) to obtain the title compound (**S23**, 424 mg, 95%) as a colorless oil.

R_f = 0.40 (pentane/EtOAc, 1:1, v/v (UV))

NMR Spectroscopy:

^{1}H NMR (500 MHz, $CDCl_3$, 25 °C, δ): 8.98 (dd, *J* = 4.2, 1.7 Hz, 1H), 8.23 (dd, *J* = 8.3, 1.7 Hz, 1H), 7.81 (dd, *J* = 8.2, 1.3 Hz, 1H), 7.73 (dd, *J* = 7.7, 1.3 Hz, 1H), 7.57 (t, *J* = 7.9 Hz, 1H), 7.50 (dd, *J* = 8.3, 4.2 Hz, 1H), 3.46 (s, 3H).

^{13}C {^{1}H} NMR (126 MHz, $CDCl_3$, 25 °C, δ): 151.1, 145.6, 141.4, 136.4, 130.0, 127.3, 126.6, 123.9, 122.2, 39.3.

HRMS GC-EI (m/z) calc'd for $C_{10}H_9NO_3S^+$ $[M]^+$, 223.0297; found, 223.0299. Deviation: –0.7 ppm.

Trifluoroethyl quinoline-7-carboxylate (S24)

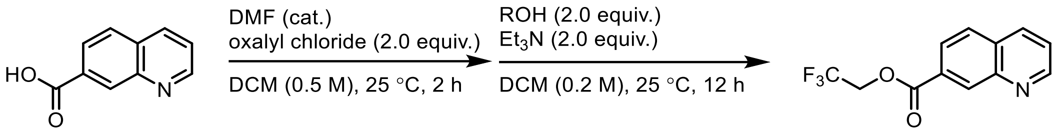

S24, 85%

Under an ambient atmosphere, a 25-mL round bottom flask equipped with a magnetic stir bar was charged with quinoline-7-carboxylic acid (346 mg, 2.00 mmol, 1.00 equiv.), followed by dry DCM (4.0 mL, *c* = 0.5 M). Subsequently, one drop of DMF followed by oxalyl chloride (343 µL, 507 mg, 4.00 mmol, 2.00 equiv.) were added to the flask. The reaction was stirred at 25 °C until gas evolution ceased (ca. 2 hours). The volatiles were removed by a rotary evaporator, then the residue was re-dissolved in dry CH_2Cl_2 (10.0 mL, *c* = 0.2 M). After that, 2-bromoethanol (496 mg, 4.00 mmol, 2.00 equiv.), and Et_3N (557 µL, 404 mg, 4.00 mmol, 2.00 equiv.) were added. The reaction was stirred at 25 °C for 2 hours. The reaction was quenched with 10 mL water, the layers were separated, and the aqueous layer was extracted twice with CH_2Cl_2 (10 mL each time). Then the combined organic layers were dried over Na_2SO_4, and the solvent was removed *in vacuo*. The residue was then purified by flash column chromatography on silica gel eluting with hexanes/ethyl acetate (from 20:1 to 10:1 + 1% Et_3N, v:v:v) to obtain the title compound (**S24**, 433 mg, 85%) as a colorless oil.

R_f = 0.25 (pentane/EtOAc, 5:1, v/v (UV))

NMR Spectroscopy:

^{1}H NMR (500 MHz, $CDCl_3$, 25 °C, δ): 9.02 (dd, *J* = 4.2, 1.8 Hz, 1H), 8.92 – 8.86 (m, 1H), 8.21 (dt, *J* = 8.4, 1.4 Hz, 1H), 8.15 (dd, *J* = 8.5, 1.7 Hz, 1H), 7.91 (d, *J* = 8.5 Hz, 1H), 7.52 (dd, *J* = 8.4, 4.2 Hz, 1H), 4.78 (q, *J* = 8.4 Hz, 2H).

^{13}C {^{1}H} NMR (126 MHz, $CDCl_3$, 25 °C, δ): 164.8, 151.8, 147.6, 136.0, 133.1, 131.3, 129.3, 128.6,

126.1, 123.2 (q, J = 277.3 Hz), 61.3 (q, J = 36.9 Hz).

^{19}F NMR (471 MHz, $CDCl_3$, 25 °C, δ): –73.5 (t, J = 8.6 Hz).

HRMS GC-EI (m/z) calc'd for $C_{12}H_8NO_2F_3^+$ [M]$^+$, 255.0502, found, 255.0506. Deviation: –1.7 ppm.

Trichloroethyl 2-methylnicotinate (S26)

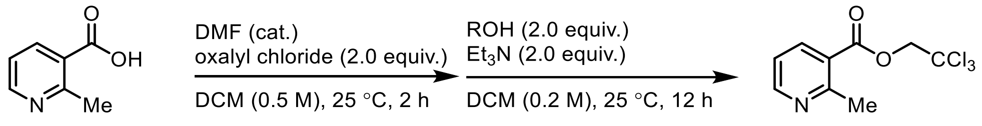

S26, 80%

Under an ambient atmosphere, a 25-mL round bottom flask equipped with a magnetic stir bar was charged with 2-methylnicotinic acid (274 mg, 2.00 mmol, 1.00 equiv.), followed by dry DCM (4.0 mL, c = 0.5 M). Subsequently, one drop of DMF followed by oxalyl chloride (343 μL, 507 mg, 4.00 mmol, 2.00 equiv.) were added to the flask. The reaction was stirred at 25 °C until gas evolution ceased (ca. 2 hours). The volatiles were removed by a rotary evaporator, then the residue was re-dissolved in dry CH_2Cl_2 (10.0 mL, c = 0.2 M). After that, 2,2,2-trichloroethanol (600 mg, 4.00 mmol, 2.00 equiv.), and Et_3N (557 μL, 404 mg, 4.00 mmol, 2.00 equiv.) were added. The reaction was stirred at 25 °C for 2 hours. The reaction was quenched with 10 mL water, the layers were separated, and the aqueous layer was extracted twice with CH_2Cl_2 (10 mL each time). Then the combined organic layers were dried over Na_2SO_4, and the solvent was removed *in vacuo*. The residue was then purified by flash column chromatography on silica gel eluting with hexanes/ethyl acetate (from 20:1 to 10:1 + 1% Et_3N, v:v:v) to obtain the title compound (**S26**, 486 mg, 80%) as a colorless oil.

R*f* = 0.30 (pentane/EtOAc, 10:1, v/v (UV))

NMR Spectroscopy:

^{1}H NMR (500 MHz, $CDCl_3$, 25 °C, δ): 8.65 (dd, J = 4.9, 1.9 Hz, 1H), 8.31 (dd, J = 7.9, 1.8 Hz, 1H), 7.24 (dd, J = 7.9, 4.9 Hz, 1H), 4.95 (s, 2H), 2.87 (s, 3H).

^{13}C {^{1}H} NMR (126 MHz, $CDCl_3$, 25 °C, δ): 164.7, 160.8, 152.7, 139.0, 124.0, 121.2, 94.9, 74.7, 25.2.

HRMS ESIpos (m/z) calc'd for $C_9H_9NO_2Cl_3^+$ [M+H]$^+$, 267.9693, found, 267.9693. Deviation: 0.0 ppm.

Ezetimibe-intermediate-A derivative S27

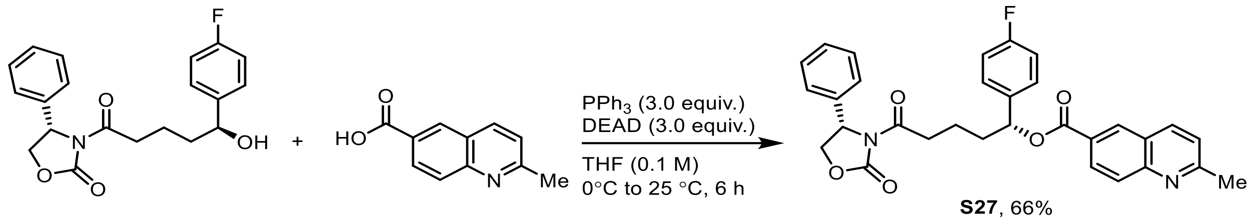

S27, 66%

Under an ambient atmosphere, a 25-mL round bottom flask equipped with a magnetic stir bar was charged with ezetimibe intermediate A (357 mg, 1.00 mmol, 1.00 equiv.), followed by dry THF (10.0 mL, *c* = 0.1 M). Subsequently, 2-methylquinoline-6-carboxylic acid (561 mg, 3.00 mmol, 3.00 equiv.), and Ph_3P (786 mg, 3.00 mmol, 3.00 equiv.) were added to the flask. The solution was cooled to 0 °C, then diethyl azodicarboxylate (522 mg, 3.00 mmol, 3.00 equiv.) was added dropwise over 10 min. Subsequently, the mixture was allowed to warm to 25 °C and the reaction mixture was stirred for 6 h. The mixture was concentrated to give a thick yellow oil. After that, 30 mL of Et_2O and 5 mL of hexane was added, and the mixture was stirred until a precipitate formed. The precipitate was removed by filtration, and the filter cake was washed with 20 mL of Et_2O/hexane (1:1). The filtrate was collected, and the solvent was removed *in vacuo*. The residue was then purified by flash column chromatography on silica gel eluting with ethyl acetate/hexanes (from 1:10 to 1:1 + 1% Et_3N, v:v:v) to obtain the title compound (**S27**, 350 mg, 66%) as a colorless oil.

$[\alpha]_D^{25}$ = –9.0 (*c* = 0.50, $CHCl_3$)

R_f = 0.35 (EtOAc/pentane, 1:1, v/v (UV))

NMR Spectroscopy:

^{1}H NMR (500 MHz, $CDCl_3$, 25 °C, δ): 8.56 (d, *J* = 1.9 Hz, 1H), 8.28 (dd, *J* = 8.8, 1.9 Hz, 1H), 8.18 (d, *J* = 8.4 Hz, 1H), 8.07 (d, *J* = 8.7 Hz, 1H), 7.50 – 7.27 (m, 8H), 7.05 (t, *J* = 8.7 Hz, 2H), 6.01 (dd, *J* = 7.8, 5.8 Hz, 1H), 5.40 (dd, *J* = 8.7, 3.7 Hz, 1H), 4.66 (t, *J* = 8.8 Hz, 1H), 4.28 (dd, *J* = 9.0, 3.7 Hz, 1H), 3.07 – 2.99 (m, 2H), 2.80 (s, 3H), 2.14 (dddd, *J* = 13.2, 10.5, 7.8, 5.2 Hz, 1H), 1.96 (ddt, *J* = 13.7, 10.9, 5.6 Hz, 1H), 1.86 – 1.74 (m, 1H), 1.75 – 1.61 (m, 1H).

^{13}C {^{1}H} NMR (126 MHz, $CDCl_3$, 25 °C, δ): 172.1, 165.4, 162.4 (d, *J* = 248.8 Hz), 161.7, 153.7, 149.7, 139.0, 137.5, 136.1 (d, *J* = 3.0 Hz), 130.8, 129.2, 129.1, 129.0, 128.8, 128.3 (d, *J* = 8.1 Hz), 127.4, 125.9, 125.7, 122.9, 115.5 (d, *J* = 21.7 Hz), 76.7, 70.0, 57.6, 35.7, 35.2, 25.6, 20.2.

^{19}F NMR (471 MHz, $CDCl_3$, 25 °C, δ): –114.2 (ddd, *J* = 13.7, 8.8, 5.2 Hz).

HRMS ESIpos (m/z) calc'd for $C_{31}H_{27}N_2FO_5^+$ $[M+H]^+$, 527.1977; found, 527.1971. Deviation: –1.1 ppm.

Florfenicol derivative S28

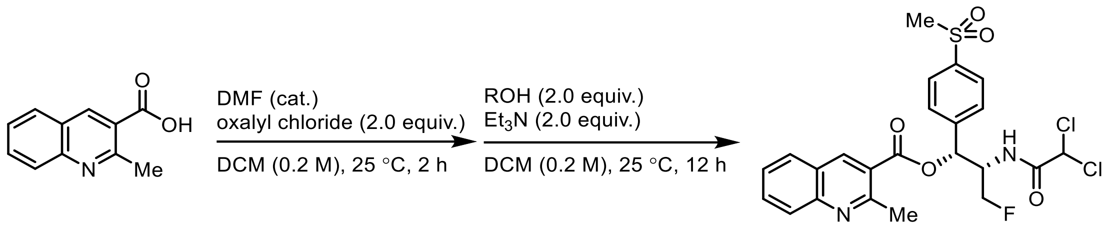

S28, 76%

Under an ambient atmosphere, a 25-mL round bottom flask equipped with a magnetic stir bar was charged with 2-methylquinoline-6-carboxylic acid (187 mg, 1.00 mmol, 1.00 equiv.), followed by dry DCM (5.0 mL, *c* = 0.2 M). Subsequently, one drop of DMF followed by oxalyl chloride (172 µL, 253 mg, 2.00 mmol, 2.00 equiv.) were added to the flask. The reaction was stirred at 25 °C until gas evolution ceased (ca. 2 hours). The volatiles were removed by a rotary evaporator, then the residue was re-dissolved in dry CH_2Cl_2 (5.0 mL, *c* = 0.2 M).After that, florfenicol (716 mg, 2.00 mmol, 2.00 equiv.), and Et_3N (278 µL, 202 mg, 2.00 mmol, 2.00 equiv.) were added. The reaction was stirred at 25 °C for 12 hours. The reaction was quenched with 5 mL water, the layers were separated, and the aqueous layer was extracted twice with CH_2Cl_2 (5 mL each time). Then the combined organic layers were dried over Na_2SO_4 and the solvent was removed *in vacuo*. The residue was then purified by flash column chromatography on silica gel eluting with hexanes/ethyl acetate (from 10:1 to 1:1 + 1% Et_3N, v:v:v) to obtain the title compound (**S28**, 401 mg, 76%) as a colorless solid.

m. p. = 197 – 199 °C

$[\alpha]_D^{25}$ = +61.6 (*c* = 0.50, $CHCl_3$)

R_f = 0.35 (pentane/EtOAc, 1:1, v/v (UV))

NMR Spectroscopy:

^{1}H NMR (500 MHz, $CDCl_3$, 25 °C, δ): 8.85 (s, 1H), 8.06 (d, *J* = 8.3 Hz, 1H), 8.06 – 8.03 (m, 2H), 7.93 (dd, *J* = 8.1, 1.5 Hz, 1H), 7.83 (ddd, *J* = 8.4, 6.9, 1.4 Hz, 1H), 7.78 (m, 2H), 7.59 (ddd, *J* = 8.1, 6.9, 1.1 Hz, 1H), 7.13 (d, *J* = 9.4 Hz, 1H), 6.36 (d, *J* = 8.8 Hz, 1H), 5.92 (s, 1H), 4.73 (dtt, *J* = 29.4, 9.4, 2.4 Hz, 1H), 4.56 (ddd, *J* = 47.3, 10.2, 3.0 Hz, 1H), 4.35 (ddd, *J* = 46.4, 10.2, 2.2 Hz, 1H), 3.07 (s, 3H), 2.95 (s, 3H).

^{13}C {^{1}H} NMR (126 MHz, $CDCl_3$, 25 °C, δ): 165.1, 164.6, 158.7, 148.9, 142.3, 141.8, 141.3, 132.7, 129.0, 128.5, 128.5, 128.5, 127.2, 125.7, 121.9, 81.9 (d, *J* = 172.6 Hz), 74.2 (d, *J* = 4.0 Hz), 66.2, 54.4 (d, *J* = 18.4 Hz), 44.6, 25.9.

^{19}F NMR (471 MHz, $CDCl_3$, 25 °C, δ): –234.0 (td, *J* = 46.7, 29.6 Hz).

HRMS ESIpos (m/z) calc'd for $C_{23}H_{22}N_2FCl_2O_5S^+$ $[M+H]^+$, 527.0605; found, 527.0606. Deviation: –0.1 ppm.

MECHANISTIC STUDIES

Kinetic isotope effect

KIE determined from intermolecular competition kinetics

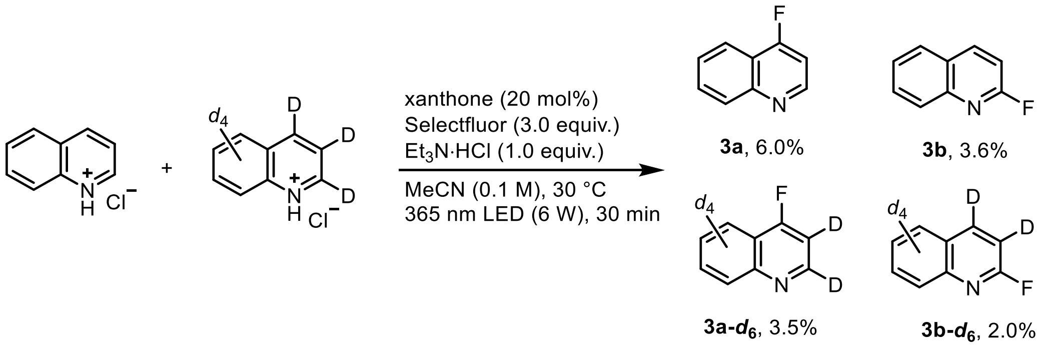

Under a nitrogen atmosphere, to a 4-mL vial equipped with a magnetic stir bar were added quinoline hydrochloride (8.3 mg, 50 μmol, 0.50 equiv.), quinoline-d_7 hydrochloride (8.6 mg, 50 μmol, 0.50 equiv.), xanthone (3.9 mg, 20 μmol, 20 mol%), Selectfluor (106.2 mg, 0.300 mmol, 3.00 equiv.), $Et_3N·HCl$ (13.7 mg, 0.100 mmol, 1.00 equiv.), and dry acetonitrile (1.0 mL, c = 0.1 M). The vial was sealed with a septum cap. Then the vial was placed onto an aluminum plate fitted with a 6 W high-power single LED plate (EpiLED Chip, size: 10 × 10 mm, λ_{max} = 365 nm, 600 mA, 9–11 V). Another aluminum block was utilized to hold the vial. The temperature was kept at approximately 30 °C through the use of a cooling fan. The reaction mixture was stirred and irradiated for 30 min. After that, 2-fluorotoluene (11 μL, 0.10 mmol, 1.0 equiv.) was added as an internal standard, followed by 1 mL saturated aqueous Na_2CO_3. An aliquot (ca. 0.1 mL) of the organic phase was taken and diluted with $CDCl_3$ (0.5 mL), and the yield was determined by ^{19}F NMR integration relative to the internal standard (**3a**, 6.0%; **3b**, 3.6%; **3a-*d*$_6$**, 3.5%; **3b-*d*$_6$**, 2.0%; Figure S14).

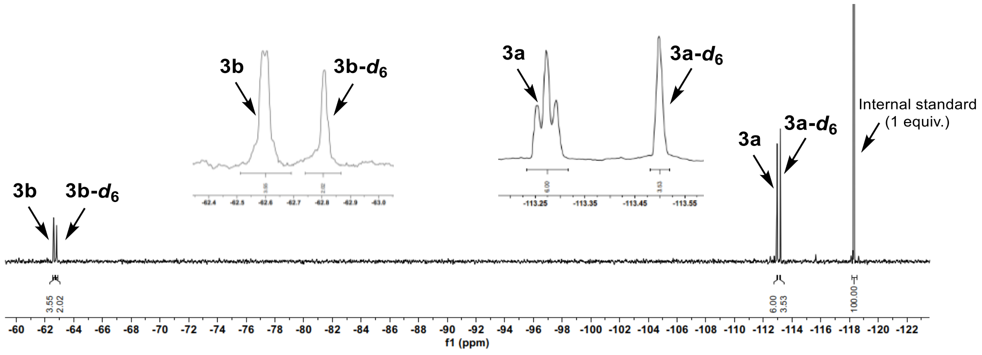

Figure S14. ^{19}F NMR of the crude mixture of intermolecular competition reactions

KIE determined from parallel reactions

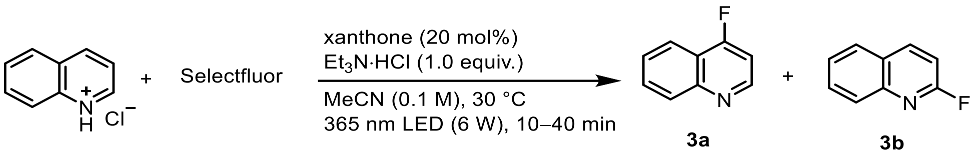

Under a nitrogen atmosphere, to a 4-mL vial equipped with a magnetic stir bar were added quinoline hydrochloride (16.6 mg, 0.100 mmol, 1.00 equiv.), xanthone (3.9 mg, 20 μmol, 20 mol%), Selectfluor (106.2 mg, 0.300 mmol, 3.00 equiv.), $Et_3N{\cdot}HCl$ (13.7 mg, 0.100 mmol, 1.00 equiv.), and dry acetonitrile (1.0 mL, c = 0.1 M). The vial was sealed with a septum cap. Then the vial was placed onto an aluminum plate fitted with a 6 W high-power single LED plate (size: 10 × 10 mm, λ_{max} = 365 nm, 600 mA, 9–11 V). Another aluminum block was utilized to hold the vial. The temperature was kept at approximately 30 °C through the use of a cooling fan. The reaction mixture was stirred and irradiated for indicated time. After that, 2-fluorotoluene (11 μL, 0.10 mmol, 1.0 equiv.) was added as an internal standard, followed by 1 mL of saturated aqueous Na_2CO_3. An aliquot (0.1 mL) of the organic phase was taken and diluted with $CDCl_3$ (0.5 mL), and the yield was determined by ^{19}F NMR integration relative to the internal standard. The results are summarized in Figure S11.

Reaction time	yield of **3a**	yield of **3b**
10 min	1.5%	1.0%
20 min	7.1%	4.1%
30 min	10.0%	6.1%
40 min	12.3%	7.5%

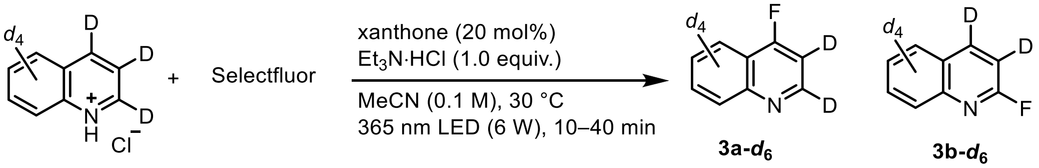

Under a nitrogen atmosphere, to a 4-mL vial equipped with a magnetic stir bar were added quinoline-d_7 hydrochloride (17.3 mg, 0.100 mmol, 1.00 equiv.), xanthone (3.9 mg, 20 μmol, 20 mol%), Selectfluor (106.2 mg, 0.300 mmol, 3.00 equiv.), $Et_3N{\cdot}HCl$ (13.7 mg, 0.100 mmol, 1.00 equiv.), and dry acetonitrile (1.0 mL, c = 0.1 M). The vial was sealed with a septum cap. Then the vial was placed onto an aluminum plate fitted with a 6 W high-power single LED plate (size: 10 × 10 mm, λ_{max} = 365 nm, 600 mA, 9–11 V). Another aluminum block was utilized to hold the vial. The temperature was kept at approximately 30 °C through the use of a cooling fan. The reaction mixture was stirred and irradiated for indicated time. After that, 2-fluorotoluene (11 μL, 0.10 mmol, 1.0 equiv.) was added as an internal standard, followed by 1 mL of saturated aqueous Na_2CO_3. An aliquot (0.1 mL) of the organic phase was taken and diluted with $CDCl_3$ (0.5

mL), and the yield was determined by ^{19}F NMR integration relative to the internal standard. The results are summarized in Figure S11.

Reaction time	yield of **3a-*d*$_6$**	yield of **3b-*d*$_6$**
10 min	1.5%	0.9%
20 min	6.4%	3.1%
30 min	9.1%	5.0%
40 min	10.8%	6.1%

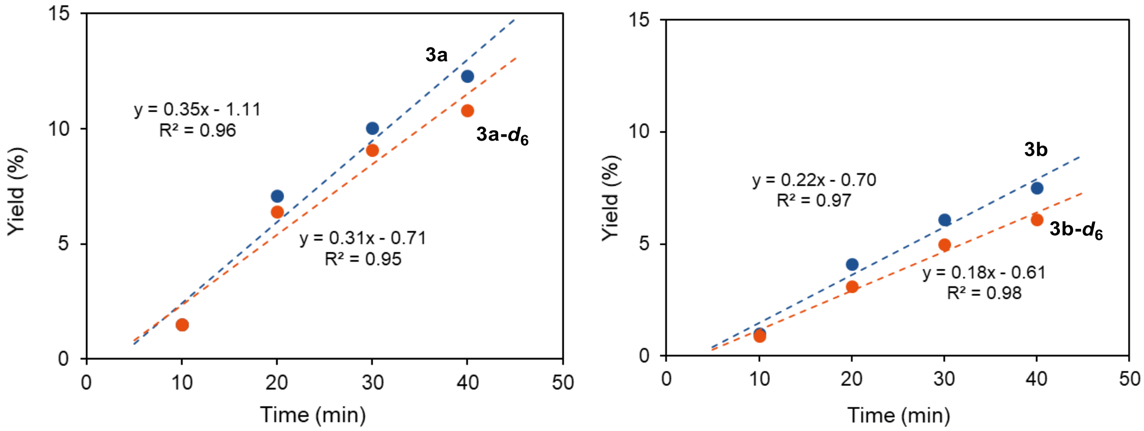

Figure S15. Kinetic profile of the fluorination reactions of quinoline hydrochloride and quinoline-*d*$_7$ hydrochloride (left, C4 fluorination; right, C2 fluorination)

X-Ray Crystallographic Data

3-Acetylquinoline hydrochloride (CCDC 2212696)

The single crystal of 3-acetylquinoline hydrochloride was obtained by mixing ca. 0.5 mL of 3-acetylquinoline solution in DCM (c = 0.1 M) and ca. 0.5 mL of HCl solution in 9:1 DCM/1,4-dioxane (c = 0.4 M). The atoms are depicted with 50% probability ellipsoids. The detailed X-ray structure is shown in Figure S16 and crystallographic data are summarized in Table S3.

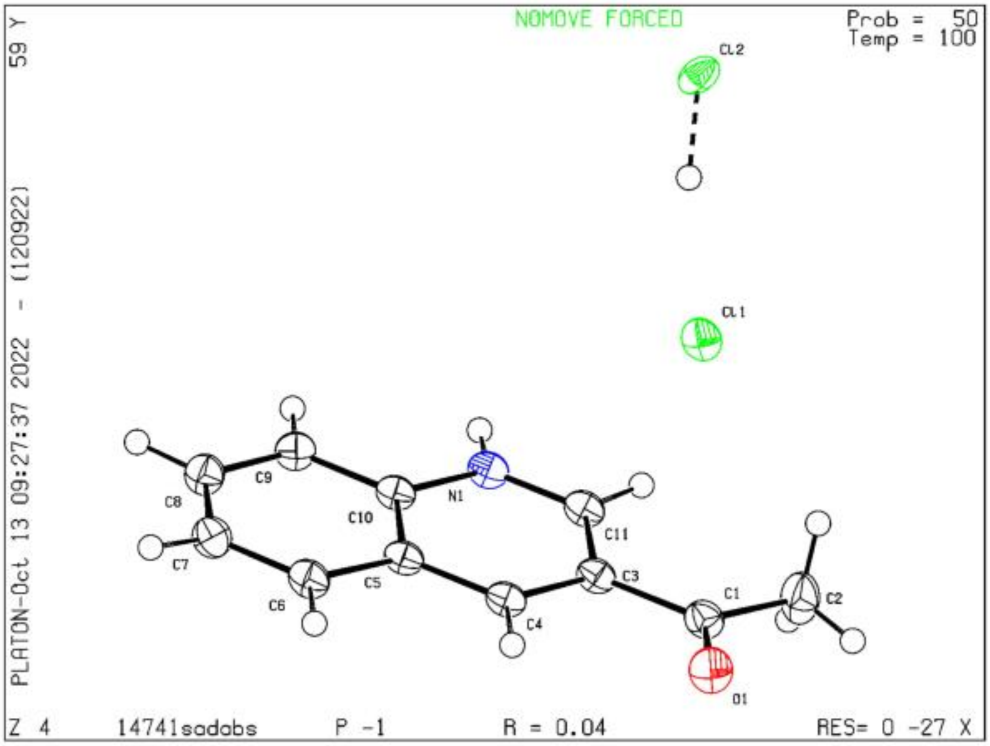

Figure S16. X-ray structure of 3-acetylquinoline hydrochloride

Table S3. Crystallographic data

Empirical formula	C_{11} $H_{10.35}$ $Cl_{1.35}$ N O
Color	colourless
Formula weight	220.41 g·mol^{-1}
Temperature	100(2) K
Wavelength	0.71073 Å
Crystal system	Triclinic
Space group	*P*-1, (no. 2)
Unit cell dimensions	a = 5.7542(3) Å α =112.224(2)°<br>b = 9.4278(4) Å β = 97.532(2)°<br>c = 10.7145(5) Å γ = 100.358(2)°
Volume	516.50(4) Å^3
Z	2
Density (calculated)	1.417 Mg·m^{-3}
Absorption coefficient	0.426 mm^{-1}
F(000)	229 e
Crystal size	0.188 x 0.133 x 0.038 mm^3
θ range for data collection	2.410 to 31.668°
Index ranges	-8 ≤ h ≤ 8, -13 ≤ k ≤ 13, -15 ≤ l ≤ 15
Reflections collected	61681
Independent reflections	3477 [R_{int} = 0.0448]
Reflections with I>2σ(I)	3062
Completeness to θ = 25.242°	99.9 %
Absorption correction	Gaussian
Max. and min. transmission	1.0000 and 0.9367
Refinement method	Full-matrix least-squares on F^2
Data / restraints / parameters	3477 / 1 / 180
Goodness-of-fit on F^2	1.139
Final R indices [I>2σ(I)]	R_1 = 0.0396 wR^2 = 0.1126
R indices (all data)	R_1 = 0.0455 wR^2 = 0.1164
Largest diff. peak and hole	0.815 and -0.249 e·Å^{-3}

DFT Calculation

Methods

Density Functional Theory (DFT) calculations were performed on the Max-Planck-Institut für Kohlenforschung computer cluster using the ORCA 5.0 program package (a development version)[6]. Structural optimizations and frequency calculations were performed with the UB3LYP functional[7,8] with D3 dispersion correction[9] and Becke-Johnson damping (BJ)[10] along with RIJCOSX approximation[11], utilizing the def2/J auxiliary basis set[12] and the def2-SVP basis set[13] on all atoms. SHARK was used for the computation of integrals[14]. Tight SCF convergence and geometry optimization criterions were chosen. Frequency calculations at the same level had been performed to confirm each stationary point to be either a minimum or a transition structure. Solvent effects of acetonitrile were taken into account using the conductor-like polarized continuum model (CPCM)[15]. To obtain more accurate energies, single-point energy calculations were performed on all optimized structures applying the def2/J auxiliary basis set[12] and the def2-TZVPP basis set[13] for all atoms. The reported energies are Gibbs free energies in solution. Molecular orbitals were generated through the LargePrint command. Natural bond orbital (NBO) analysis was done by applying the NBO keyword (NBO version 7.0[16]) with the same basis set of single-point energy calculations. Input files were created using Avogadro 1.2[17] and images were generated using Chemcraft 1.8[18].

Discussion

Potential surface

The potential surface of the reaction between protonated quinoline and $TEDA^{2+\cdot}F^{-}$ is shown in Figure S17 ($TEDA^{+}$ as the proton acceptor) and Figure S18 (Cl^{-} as the proton acceptor).

On the potential surface, we take the pre-associated complexes into consideration, including **Complex-1** of $Q\text{-}H^{+}$ and $TEDA^{2+\cdot}F^{-}$, **Complex-3** of **Int-3** and $TEDA\text{-}H^{2+}$. The **Complex-4** of Meisenheimer intermediate **Int-1** and $TEDA^{2+\cdot}$ has no energy minima in calculation and directly leads to the product (**Complex-2**) after electron transfer, which indicates the low barrier associated with the electron transfer. The related conformations of **Complex-2** of radical cation **Int-2** and $TEDA^{+}$ are listed in Table 4 and one of the most stable conformations was applied as reactant for transition state analysis.

In addition to frequency calculations, the transition state of fluoride-coupled electron transfer (**TS**) was confirmed by IRC (intrinsic reaction coordinate, Figure S19, S21). The reaction pathway of C–H cleavage from **Complex-2** to **Complex-3** was analyzed by NEB-TS (Nudged Elastic Band with TS optimization, Figure S20, S22).

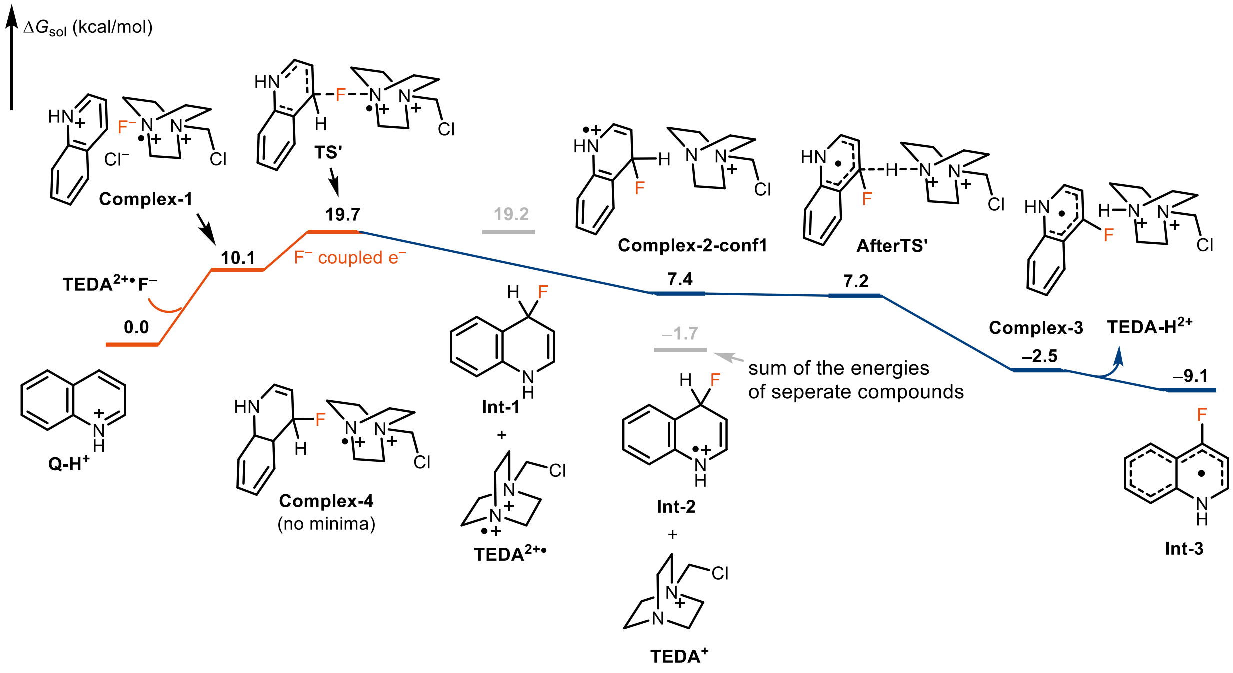

Figure S17. Potential surface of the reaction between protonated quinoline and $TEDA^{2+\cdot}F^{-}$

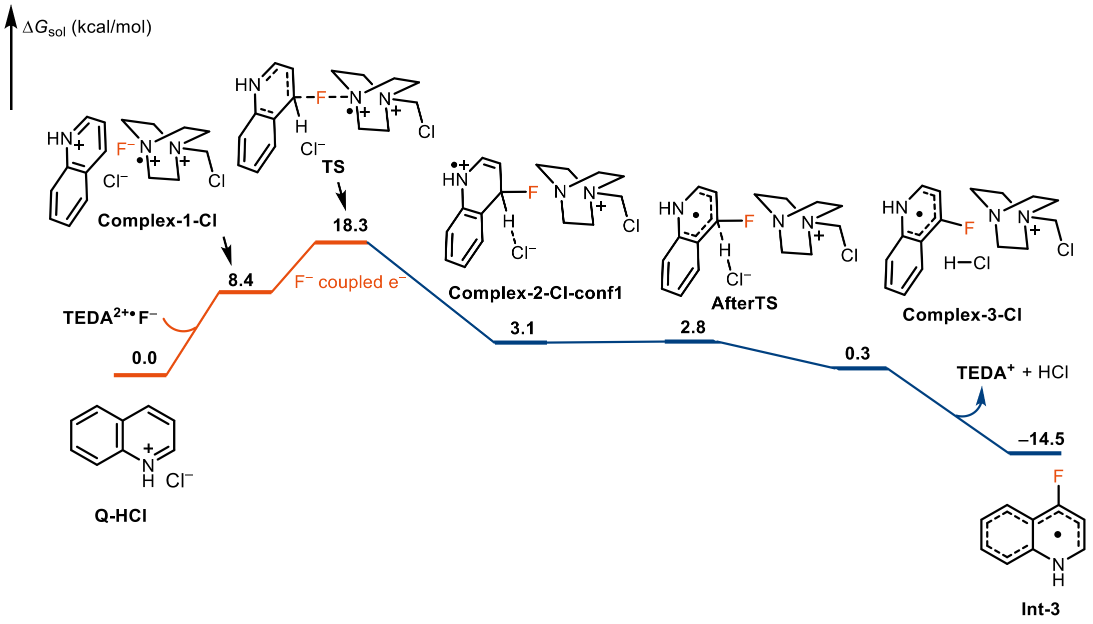

Figure S18. Potential surface of the reaction between quinoline hydrochloride and $TEDA^{2+\cdot}F^{-}$

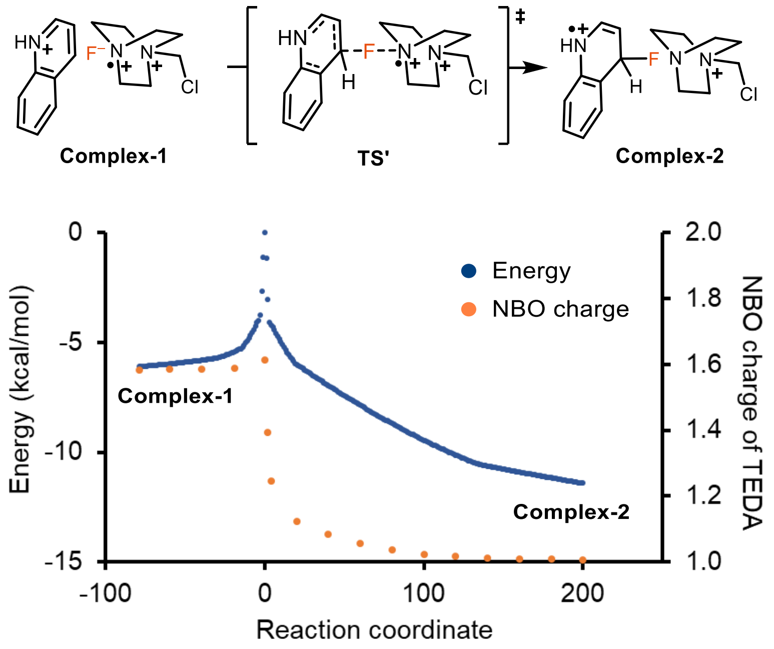

Figure S19. Potential surface (IRC, UB3LYP-D3(BJ)/SVP level) of the fluoride-coupled electron transfer

The NBO analysis of the structures along the internal reaction coordinate (IRC) shows a significant charge transfer process after the transition state, consistent with a fluoride-coupled electron transfer process. The possibility of a fluorine atom transfer process was also taken into consideration. However, the radical addition to protonated quinolines are usually limited to nucleophilic radicals such as alkyl-, boryl-, or silyl-centered radicals[19], but not the electrophilic fluorine radical due to the lack of polarity match[20].

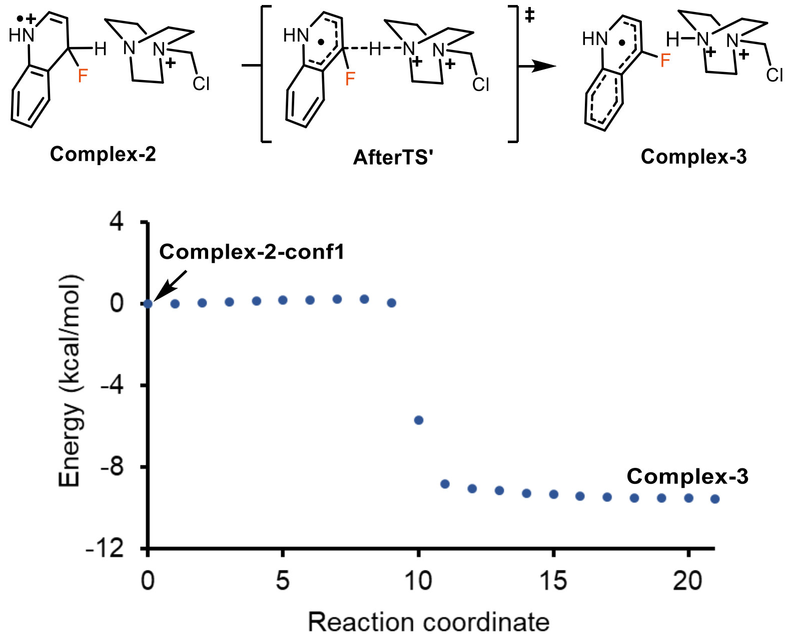

Figure S20. Potential surface (NEB, UB3LYP-D3(BJ)/SVP level) from **Complex-2** to **Complex-3**

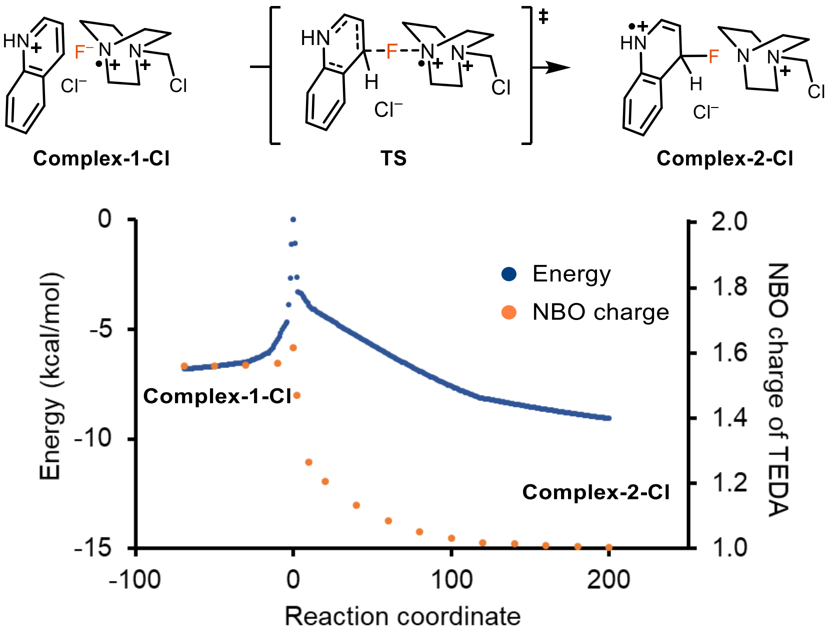

Figure S21. Potential surface (IRC, UB3LYP-D3(BJ)/SVP level) of the fluoride-coupled electron transfer

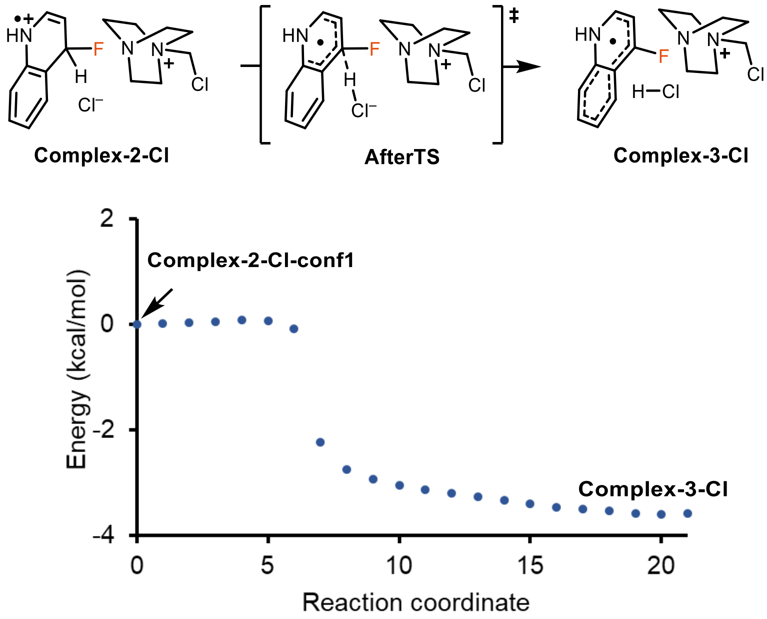

Figure S22. Potential surface (NEB, UB3LYP-D3(BJ)/SVP level) from **Complex-2-Cl** to **Complex-3-Cl**

Table S4. Conformation analysis of Complex-2

**Complex-2-conf1**<br>**Structure for NEB-TS calculation**	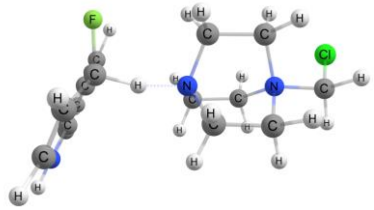<br>ΔG = 0.0 kcal/mol
**Complex-2-conf2**	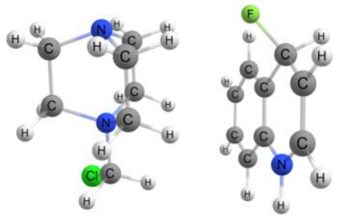<br>ΔG = 0.5 kcal/mol
**Complex-2-conf3**	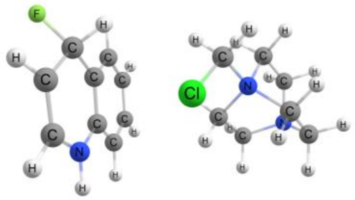<br>ΔG = 0.6 kcal/mol
**Complex-2-conf4**	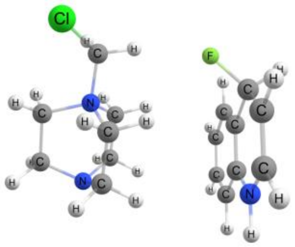<br>ΔG = 0.9 kcal/mol
**Complex-2-conf5**	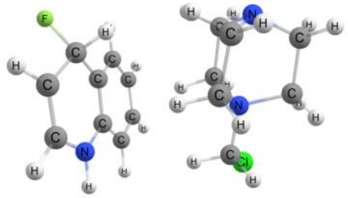<br>ΔG = 1.1 kcal/mol
**Complex-2-conf6**	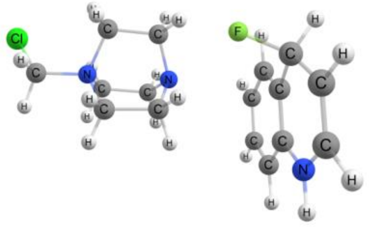<br>ΔG = 1.5 kcal/mol

Barriers to the outersphere single electron transfer

The Gibbs free energies of activation of the outersphere single electron transfer (SET) between **Int-3** and Selectfluor as well as Meisenheimer intermediate **Int-1** and $TEDA^{2+\cdot}$ were calculated using Marcus theory.

Based on Marcus theory[21-24], the activation energy of outersphere single electron transfer can be calculated from:

$$\Delta G^{\ddagger} = \frac{(\lambda + \Delta G^{\circ})^2}{4\lambda}$$

Here, the ΔG° = –46.2 kcal/mol is the reaction energy of the outersphere single electron transfer obtained from DFT calculation. The λ is calculated from:

$$\lambda = \lambda_i + \lambda_0$$

The inner reorganization energy is expected to be small, and thus neglected in the calculations. The solvent reorganization energy λ_0 is calculated as follows:

$$\lambda_0 = 95[(2r_{\mathbf{Int\text{-}3}})^{-1} + (2r_{\text{Selectfluor}})^{-1} - (r_{\mathbf{Int\text{-}3}} + r_{\text{Selectfluor}})^{-1}] = 95(\frac{1}{2\times3.6} + \frac{1}{2\times3.9} - \frac{1}{3.6\times3.9}) = 18.6\ \text{kcal/mol}$$

Here, the radii of **Int-3** and Selectfluor (3.6 and 3.9 Å, respectively) were derived from the molecular volume (inside a contour of 0.001 electrons/$Bohr^3$ density) by assuming the shapes of the molecules are perfect spheres.

Thus, the activation energy can be obtained:

$$\Delta G^{\ddagger} = \frac{(\lambda + \Delta G^{\circ})^2}{4\lambda} = \frac{(18.6 - 46.2)^2}{4\times18.6} = 10.4\ \text{kcal/mol}$$

The results are summarized below:

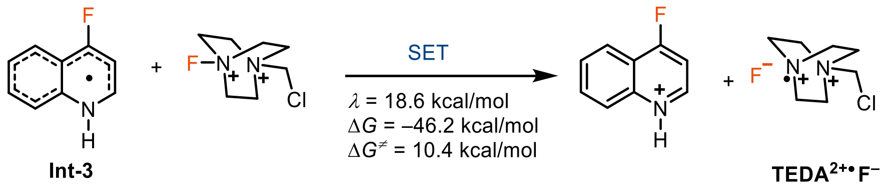

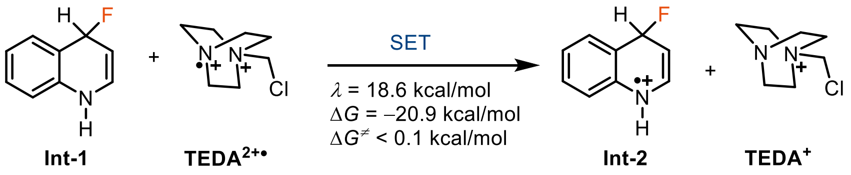

Discussion of the hydrogen atom transfer mechanism

A typical HAT transition state is concerted transfer of H^+ and e^- from a single donor group[25-27]. However, in our reaction, the DFT-calculated HOMO of **Int-1** shows that the fluoride and electron-rich enamine sub-structures have the major contribution to the HOMO, and the C–H bond contributes considerably less, which indicates that a C–H cleavage via a traditional HAT pathway is difficult (Figure S23). Therefore, it is more likely that our reaction proceeds via an asynchronous F^--e^--H^+ transfer rather than a fluoride attack followed by HAT mechanism. For comparison, the DFT-calculated SOMO of the Meisenheimer intermediate in the Chichibabin-type fluorination[25] shows a large contribution from the C–H bond because the Meisenheimer intermediate behaves more like a π-radical after AgF_2 addition and charge redistribution, and a HAT mechanism was proposed for the Chichibabin-type reaction[28].

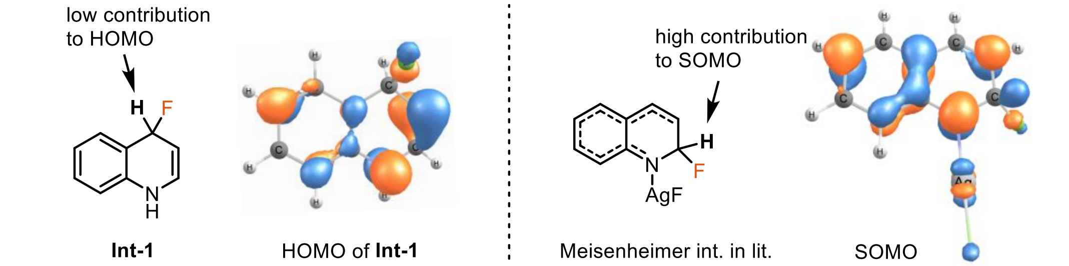

Figure S23. Comparison of the orbitals of Meisenheimer intermediates (*iso*-surface value = 0.06)

Discussion of the site-selectivity of the fluorination

The calculated LUMOs of protonated quinolines show that the C4 position of the quinoline rings have the largest contribution, indicating the site-selectivity of fluorination is consistent with the electrophilicity of the quinoline rings (Figure S24). In the case of 3-acetylquinoline, the enhanced C4 reactivity can also be explained by the electronic effects (Figure S24, right).

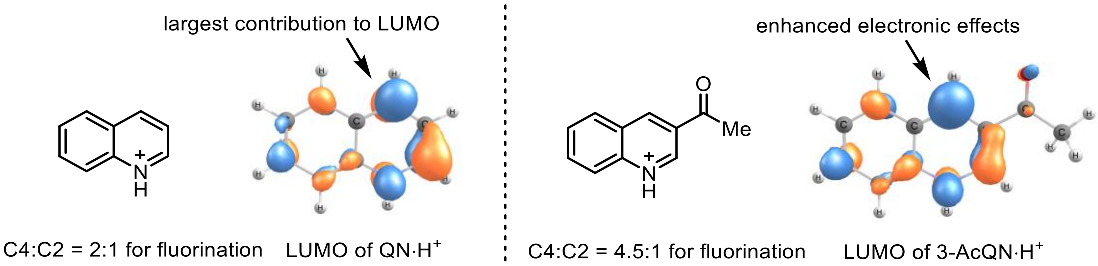

Figure S24. Orbital analysis of protonated quinolines (*iso*-surface value = 0.06)

Calculated coordinates

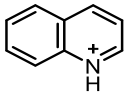

Q-H+

G_{sol} = -402.2080371 Hartree

C	-5.212826	2.100026	2.673007
C	-3.831234	2.103030	2.672829
C	-3.154390	3.340490	2.672921
C	-3.874768	4.573729	2.673201
C	-5.295517	4.527444	2.673374
C	-5.948586	3.313081	2.673277
H	-1.264548	2.518553	2.672512
H	-5.746583	1.147016	2.672937
H	-3.259397	1.172428	2.672620
C	-3.138574	5.784255	2.673288
H	-5.850545	5.467931	2.673582
H	-7.040083	3.279366	2.673409
C	-1.753828	5.770107	2.673108
C	-1.089706	4.538144	2.672828
H	-3.680962	6.733091	2.673498
H	-1.170439	6.690966	2.673173
H	-0.002120	4.447733	2.672664
N	-1.782814	3.399952	2.672743

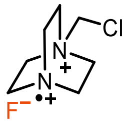

TEDA2+·F−

G_{sol} = -944.2108825 Hartree

C	-1.499143	-0.943628	1.214113
C	-2.177529	1.015107	-0.014116
C	-0.025391	-0.466612	-1.241559
C	-0.023565	-0.460595	1.243019
N	0.235700	0.373068	-0.001472
C	-0.722838	1.557998	-0.003437
C	-1.493522	-0.970352	-1.195625

C	1.630158	0.934105	-0.004210
Cl	2.879529	-0.328744	0.004821
H	-2.054285	-0.563691	2.080186
H	-1.554164	-2.038828	1.205713
H	-2.729874	1.357034	0.869327
H	-2.710531	1.337494	-0.916595
H	0.698896	-1.287661	-1.231511
H	0.170864	0.182372	-2.103686
H	0.191298	0.186046	2.102396
H	0.688942	-1.291783	1.225634
H	-0.502423	2.145387	0.895652
H	-0.492408	2.152257	-0.895434
H	-2.054932	-0.624333	-2.071778
H	-1.533089	-2.065490	-1.154048
H	1.745553	1.548662	0.895800
H	1.746676	1.536910	-0.912026
F	-3.999465	-1.077819	-0.000047
N	-2.111313	-0.433211	0.002020

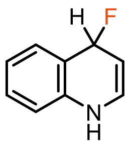

Int-1

G_{sol} = -502.2063022 Hartree

H	-0.913265	0.407384	4.150569
F	2.304092	-0.795035	1.080868
C	-2.498490	-0.079857	0.519938
C	-2.025016	0.192720	1.798111
C	-0.794773	-0.347947	2.225893
C	-0.036321	-1.159737	1.349955
C	-0.545444	-1.427225	0.068473
C	-1.764077	-0.901053	-0.352919
H	-3.454264	0.342716	0.198901
H	-2.600337	0.818505	2.485622
C	1.329225	-1.636175	1.745488
H	0.042405	-2.055947	-0.606693
H	-2.143634	-1.119288	-1.353822

C	1.549567	-1.565248	3.211166
C	0.767664	-0.784229	3.993871
H	1.536573	-2.635810	1.331727
H	2.402840	-2.085798	3.649249
H	0.956593	-0.650827	5.061549
N	-0.338162	-0.131353	3.512725

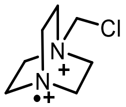

TEDA$^{2+\cdot}$

G_{sol} = -844.1819313 Hartree

C	-1.512366	-1.018291	1.189903
C	-2.216571	1.016635	0.057211
C	-0.027020	-0.523672	-1.207354
C	-0.061752	-0.417994	1.268817
N	0.208386	0.366685	-0.001125
C	-0.745024	1.549665	-0.072404
C	-1.545157	-0.929140	-1.240708
C	1.616033	0.930741	0.000611
Cl	2.848000	-0.339546	0.013380
H	-2.096105	-0.723287	2.071174
H	-1.485611	-2.108183	1.075436
H	-2.661341	1.308670	1.015700
H	-2.824861	1.365956	-0.786760
H	0.617348	-1.402493	-1.104006
H	0.244595	0.048571	-2.102240
H	0.041468	0.276969	2.110557
H	0.683817	-1.216696	1.339936
H	-0.506968	2.229448	0.753879
H	-0.580710	2.045212	-1.036021
H	-2.063042	-0.458169	-2.084468
H	-1.649549	-2.021035	-1.271377
H	1.720593	1.544081	0.903176
H	1.727367	1.533738	-0.908316
N	-2.072123	-0.418409	0.002356

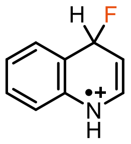

Int-2

G_{sol} = -502.0067339 Hartree

H	-1.028351	0.214215	4.180929
F	2.290447	-1.496649	0.882572
C	-2.507507	-0.062972	0.529769
C	-2.098285	0.080053	1.846325
C	-0.854344	-0.460743	2.243039
C	-0.029392	-1.159367	1.322681
C	-0.459988	-1.283692	0.006636
C	-1.688624	-0.738574	-0.392114
H	-3.465415	0.351579	0.209881
H	-2.713165	0.604636	2.581077
C	1.269638	-1.767331	1.787185
H	0.167241	-1.809190	-0.716014
H	-2.012670	-0.844223	-1.429759
C	1.667300	-1.350461	3.162197
C	0.802200	-0.680249	3.984307
H	1.151619	-2.873046	1.801028
H	2.657833	-1.635033	3.522448
H	1.042182	-0.416333	5.015140
N	-0.415543	-0.286822	3.533343

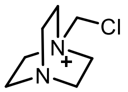

TEDA⁺

G_{sol} = -844.4148141 Hartree

C	-1.530491	-0.951546	1.200155
C	-2.203272	0.993668	-0.009558
C	-0.055082	-0.477606	-1.236041
C	-0.055921	-0.465874	1.240864
N	0.209082	0.362970	-0.001431
C	-0.750236	1.543410	-0.007058
C	-1.527373	-0.969305	-1.187962

C	1.599375	0.923995	-0.003777
Cl	2.856143	-0.335917	0.004823
H	-2.066198	-0.587632	2.087860
H	-1.560313	-2.049894	1.206966
H	-2.739897	1.362548	0.875451
H	-2.731739	1.348224	-0.905263
H	0.668807	-1.299501	-1.220556
H	0.153155	0.168264	-2.098494
H	0.157038	0.186069	2.097534
H	0.664345	-1.291017	1.230669
H	-0.521833	2.137033	0.886653
H	-0.517443	2.132915	-0.902351
H	-2.063673	-0.623151	-2.082385
H	-1.552565	-2.067722	-1.175521
H	1.716898	1.537789	0.896339
H	1.718166	1.526880	-0.911114
N	-2.207565	-0.465144	0.001553

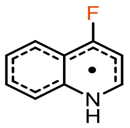

Int-3

G_{sol} = -501.5902549 Hartree

C	-5.249243	2.094878	2.673009
C	-3.842925	2.115323	2.672828
C	-3.155465	3.332611	2.672918
C	-3.885873	4.567310	2.673198
C	-5.296782	4.519555	2.673373
C	-5.969699	3.291525	2.673278
H	-1.257523	2.509641	2.672529
H	-5.772264	1.135493	2.672935
H	-3.272685	1.182585	2.672616
C	-3.125089	5.772424	2.673283
H	-5.852621	5.458728	2.673584
H	-7.062590	3.273719	2.673416
C	-1.722695	5.774048	2.673097
C	-1.054521	4.569841	2.672826

H	-1.172171	6.715957	2.673168
H	0.031089	4.481073	2.672667
N	-1.768506	3.386109	2.672742
F	-3.794938	6.949712	2.673554

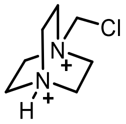

TEDA-H^{2+}

G_{sol} = -844.8431559 Hartree

C	-1.469992	-1.063919	1.170022
C	-2.198206	1.034720	0.115027
C	-0.036951	-0.574675	-1.169586
C	-0.102890	-0.375662	1.288572
N	0.194866	0.361296	-0.000520
C	-0.765729	1.530926	-0.127434
C	-1.538728	-0.876489	-1.279721
C	1.594340	0.924469	-0.000977
Cl	2.833338	-0.341121	0.025781
H	-2.075202	-0.901967	2.069757
H	-1.383011	-2.140106	0.978852
H	-2.564198	1.290178	1.116596
H	-2.887649	1.432478	-0.638940
H	0.554182	-1.477002	-0.981862
H	0.337562	-0.077706	-2.072488
H	-0.076546	0.370210	2.092834
H	0.696845	-1.106713	1.448715
H	-0.479824	2.282991	0.617468
H	-0.629109	1.944389	-1.134129
H	-2.021740	-0.319488	-2.091434
H	-1.715925	-1.948913	-1.421318
H	1.698678	1.547851	0.894603
H	1.708234	1.518608	-0.915370
N	-2.198146	-0.461608	0.003600
H	-3.171056	-0.799050	0.005093

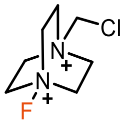

Selectfluor^{2+}

G_{sol} = -944.0124058 Hartree

C	-1.459317	-1.055941	1.177128
C	-2.187733	1.043160	0.109740
C	-0.020333	-0.562663	-1.173796
C	-0.081953	-0.377486	1.284169
N	0.211062	0.367569	-0.000664
C	-0.748710	1.537661	-0.120649
C	-1.523177	-0.879507	-1.280794
C	1.615263	0.932237	0.000030
Cl	2.848571	-0.334941	0.021111
H	-2.080763	-0.858658	2.058943
H	-1.395491	-2.133317	0.984819
H	-2.569537	1.286040	1.108407
H	-2.871449	1.409360	-0.665435
H	0.574320	-1.464991	-0.998694
H	0.336445	-0.057217	-2.078972
H	-0.045378	0.356521	2.098547
H	0.706314	-1.123900	1.429560
H	-0.474979	2.281285	0.637063
H	-0.613386	1.963134	-1.122174
H	-2.024572	-0.330927	-2.086943
H	-1.703936	-1.956199	-1.381682
H	1.716351	1.551868	0.898807
H	1.724824	1.529888	-0.912796
F	-3.467449	-0.900547	0.004724
N	-2.161843	-0.448735	0.002692

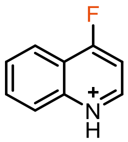

FQ-H+

G_{sol} = -501.4653564 Hartree

C	-5.215321	2.104111	2.673008
C	-3.833078	2.106972	2.672831
C	-3.153181	3.341671	2.672921
C	-3.882864	4.566867	2.673200
C	-5.300664	4.531631	2.673374
C	-5.951420	3.315160	2.673276
H	-1.260303	2.520310	2.672512
H	-5.749191	1.151292	2.672939
H	-3.261027	1.176708	2.672624
C	-3.130117	5.768910	2.673286
H	-5.855184	5.470636	2.673580
H	-7.042737	3.280770	2.673407
C	-1.746640	5.773000	2.673106
C	-1.091442	4.541801	2.672827
H	-1.183955	6.706427	2.673175
H	-0.003287	4.459373	2.672664
N	-1.780347	3.400304	2.672743
F	-3.783743	6.914585	2.673546

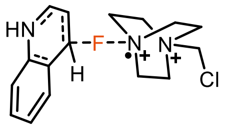

TS’

G_{sol} = -1346.387468 Hartree

C	-2.964203	-1.458115	0.130720
C	-2.611534	-0.320731	0.832755
C	-1.539115	-0.387944	1.747453
C	-0.809589	-1.598880	1.938540
C	-1.203684	-2.742351	1.207421
C	-2.265157	-2.675293	0.323485
H	-3.801277	-1.422696	-0.569973
H	-3.164660	0.612788	0.702208
C	0.302302	-1.596093	2.843647
H	-0.667220	-3.680943	1.362130
H	-2.578053	-3.565547	-0.226036
C	0.509815	-0.475234	3.675597
C	-0.246199	0.666870	3.471182

H	0.759560	-2.548861	3.111062
H	1.247133	-0.491636	4.478211
H	-0.130408	1.569778	4.074077
H	-1.766531	1.538936	2.429610
N	-1.199745	0.696538	2.521995
C	4.427068	-1.933727	0.767964
C	3.825428	0.362959	0.369798
C	4.231164	-1.337786	-1.997247
C	5.727155	-1.691831	-0.044895
N	5.402360	-0.762476	-1.208785
C	4.984033	0.589045	-0.644502
C	2.983146	-1.370053	-1.071906
C	6.613370	-0.553047	-2.091668
Cl	7.156262	-2.042363	-2.865755
H	4.590348	-1.755752	1.838911
H	4.065044	-2.963914	0.656728
H	4.147364	0.568293	1.398999
H	2.974913	1.020689	0.147940
H	4.541785	-2.331691	-2.338894
H	4.091552	-0.700379	-2.880453
H	6.509840	-1.191234	0.540998
H	6.147905	-2.609995	-0.471145
H	5.869532	1.041342	-0.178808
H	4.684858	1.209346	-1.499788
H	2.217498	-0.654723	-1.399826
H	2.522124	-2.366138	-1.062832
H	7.419392	-0.157323	-1.460764
H	6.340849	0.179870	-2.861727
F	1.729151	-1.322501	1.542973
N	3.406663	-1.023174	0.273984

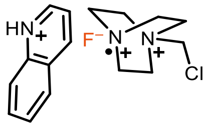

Complex-1

G_{sol} = -1346.4028403 Hartree

C	-1.061062	0.234935	-0.580510

C	-0.868138	0.729772	0.695257
C	-0.603438	-0.174428	1.744566
C	-0.542771	-1.579306	1.506939
C	-0.746889	-2.049478	0.181889
C	-0.998697	-1.157985	-0.840802
H	-1.260606	0.927083	-1.401520
H	-0.909587	1.801142	0.903146
C	-0.265868	-2.437279	2.599838
H	-0.696956	-3.124077	-0.006510
H	-1.151549	-1.521098	-1.859188
C	-0.047622	-1.918574	3.861867
C	-0.114104	-0.532523	4.045470
H	-0.219951	-3.515366	2.426772
H	0.178427	-2.558174	4.715159
H	0.049062	-0.052798	5.011801
H	-0.439777	1.275651	3.192874
N	-0.403668	0.268874	3.024878
C	4.671231	-0.454365	1.187925
C	2.764230	0.120013	-0.170698
C	3.927517	-2.356749	-0.926215
C	5.574583	-0.718906	-0.045782
N	4.684033	-1.072373	-1.226488
C	3.676546	0.053498	-1.424289
C	3.174908	-2.181081	0.418988
C	5.475330	-1.187718	-2.499296
Cl	6.749561	-2.421090	-2.406450
H	4.703713	0.599978	1.487615
H	4.974994	-1.078580	2.036502
H	2.744312	1.133857	0.246182
H	1.741775	-0.188692	-0.412463
H	4.663469	-3.166788	-0.889447
H	3.246055	-2.520233	-1.770061
H	6.156226	0.164257	-0.336946
H	6.251892	-1.565374	0.107434
H	4.257227	0.972281	-1.570326
H	3.110353	-0.173028	-2.335484
H	2.111962	-2.419764	0.299885
H	3.601497	-2.816687	1.203907

H	5.941356	-0.214380	-2.690100
H	4.780803	-1.458598	-3.302346
F	2.269951	-0.551862	2.507240
N	3.309328	-0.791903	0.817378

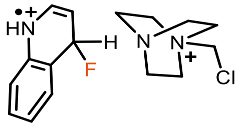

Complex-2-conf1

G_{sol} = -1346.4071139 Hartree

C	-4.115555	-1.176138	1.601266
C	-3.588646	-0.266720	2.506021
C	-2.231646	0.099717	2.402917
C	-1.404361	-0.460391	1.395283
C	-1.960428	-1.370534	0.490064
C	-3.305403	-1.723177	0.587341
H	-5.166495	-1.463927	1.673946
H	-4.205962	0.176866	3.290743
C	0.046657	-0.131769	1.404204
H	-1.327205	-1.797390	-0.289555
H	-3.733238	-2.431529	-0.125440
C	0.414451	1.060856	2.168439
C	-0.442925	1.550618	3.119870
H	0.548183	-1.033576	1.968865
H	1.412349	1.486466	2.050849
H	-0.179132	2.366949	3.793722
H	-2.302247	1.446210	3.969109
N	-1.694707	1.046989	3.253409
C	0.306975	-3.496551	2.772134
C	2.180498	-2.210394	3.561357
C	2.902419	-4.232024	1.669125
C	0.999313	-4.833335	3.137724
N	2.502062	-4.633873	3.075643
C	2.882507	-3.510452	4.027189
C	2.112579	-2.959634	1.274867
C	3.239740	-5.862294	3.526575
Cl	2.878986	-7.287876	2.529579
H	-0.278132	-3.117733	3.620358

H	-0.368212	-3.638142	1.918715
H	1.567607	-1.797666	4.373347
H	2.921449	-1.456901	3.263850
H	2.679422	-5.082324	1.016143
H	3.987070	-4.067538	1.690004
H	0.767237	-5.163187	4.157730
H	0.756565	-5.636381	2.433864
H	2.561673	-3.825339	5.027804
H	3.975737	-3.428711	4.003459
H	2.800553	-2.158997	0.974378
H	1.433814	-3.167557	0.437823
H	2.939515	-6.071632	4.559152
H	4.312650	-5.650477	3.462216
F	0.626694	-0.193193	0.145245
N	1.317067	-2.497254	2.415006

Complex-2-conf2

G_{sol} = -1346.4063842 Hartree

C	1.806423	-0.535100	-0.851602
C	1.813075	0.621999	-0.087377
C	1.759477	0.517949	1.321128
C	1.693964	-0.747032	1.962398
C	1.682350	-1.891796	1.172215
C	1.740590	-1.791548	-0.225238
H	1.853356	-0.467596	-1.939995
H	1.863585	1.609966	-0.550417
C	1.635273	-0.829677	3.465864
H	1.635057	-2.872552	1.649351
H	1.737355	-2.700784	-0.829954
C	1.819794	0.481996	4.148605
C	1.849099	1.657893	3.447007
H	0.643613	-1.229063	3.764832
H	1.897153	0.491756	5.237502
H	1.930769	2.635063	3.924653
H	1.851215	2.557710	1.609929
N	1.801894	1.656168	2.091800
C	7.677590	-1.560003	2.537882
C	5.797336	-0.566457	3.629190

C	5.351840	-1.251343	0.869042
C	7.641206	-0.491725	1.409849
N	6.203512	-0.048583	1.223184
C	5.707274	0.533792	2.537420
C	5.463030	-2.290188	2.015890
C	6.077437	1.032974	0.192680
Cl	6.599840	0.514141	-1.426583
H	8.334521	-1.218999	3.349996
H	8.080186	-2.503221	2.143689
H	6.441818	-0.222960	4.449961
H	4.795154	-0.764979	4.030251
H	5.719993	-1.629644	-0.090501
H	4.332638	-0.874487	0.732781
H	8.214561	0.410484	1.656982
H	7.981744	-0.879663	0.443697
H	6.340113	1.403654	2.752674
H	4.680261	0.869477	2.359157
H	4.468312	-2.479958	2.439244
H	5.861998	-3.236275	1.625288
H	6.703800	1.874384	0.509292
H	5.021598	1.322621	0.141174
F	2.573888	-1.752125	3.942741
N	6.341431	-1.801892	3.075070

Complex-2-conf3

G_{sol} = -1346.4060353 Hartree

C	1.364412	0.489818	0.029594
C	1.915336	1.140020	1.123499
C	2.222830	0.395117	2.284485
C	1.965196	-0.998700	2.353353
C	1.408572	-1.626022	1.243917
C	1.111412	-0.891766	0.086733
H	1.123443	1.053958	-0.873241
H	2.118010	2.213300	1.106431
C	2.285248	-1.769922	3.609371
H	1.207709	-2.698591	1.278598
H	0.675428	-1.399954	-0.775939
C	3.018136	-0.968398	4.630763

C	3.209433	0.380092	4.485280
H	1.334442	-2.109115	4.071057
H	3.378857	-1.477361	5.526660
H	3.700476	0.996633	5.239338
H	2.980934	2.024781	3.293280
N	2.805498	1.019520	3.361188
C	-2.446566	2.014944	1.190881
C	-4.286579	0.506991	1.385515
C	-3.201840	1.341641	3.906912
C	-1.548569	1.110209	2.075085
N	-2.433814	0.356231	3.047297
C	-3.431433	-0.465869	2.243663
C	-4.018677	2.272745	2.970039
C	-1.644001	-0.606239	3.878716
Cl	-0.377170	0.190049	4.849022
H	-2.333805	1.732038	0.135086
H	-2.139031	3.063800	1.300558
H	-4.194741	0.239227	0.323930
H	-5.343998	0.424000	1.671895
H	-2.462900	1.884157	4.506756
H	-3.834982	0.741501	4.572669
H	-1.011668	0.349006	1.498849
H	-0.829367	1.680353	2.672549
H	-2.840973	-1.162536	1.635603
H	-4.029128	-1.032564	2.968221
H	-5.084246	2.218614	3.232646
H	-3.686668	3.312587	3.094786
H	-1.160656	-1.320043	3.202534
H	-2.333701	-1.111867	4.563096
F	3.001667	-2.923349	3.301816
N	-3.850102	1.886394	1.572036

Complex-2-conf4

G_{sol} = -1346.4056581 Hartree

C	1.839968	-0.476273	-0.840287
C	1.752848	0.671827	-0.068128
C	1.689925	0.552839	1.339224
C	1.706080	-0.718708	1.969905

C	1.790173	-1.854444	1.172202
C	1.858937	-1.739196	-0.224034
H	1.893939	-0.396954	-1.927599
H	1.736128	1.664500	-0.523490
C	1.650809	-0.821812	3.471868
H	1.808410	-2.839658	1.642451
H	1.929813	-2.641105	-0.835567
C	1.681929	0.495873	4.168273
C	1.642854	1.675656	3.474369
H	0.724928	-1.354488	3.768419
H	1.724208	0.503718	5.259030
H	1.631952	2.652188	3.960150
H	1.620726	2.590082	1.644540
N	1.633616	1.682966	2.118227
C	7.770231	0.181234	0.913729
C	5.747121	-0.623432	-0.069345
C	5.495073	0.197555	2.677190
C	7.657950	-0.839250	2.080856
N	6.188593	-1.106067	2.336349
C	5.577310	-1.671253	1.064077
C	5.678048	1.179761	1.490883
C	5.977252	-2.129571	3.411946
Cl	6.650117	-1.633121	4.982434
H	8.348967	-0.259465	0.090226
H	8.294795	1.083039	1.258300
H	6.315454	-1.064598	-0.899522
H	4.760029	-0.324274	-0.446423
H	5.947007	0.563058	3.605453
H	4.448382	-0.058013	2.871640
H	8.115123	-1.809336	1.847132
H	8.079452	-0.460965	3.018458
H	6.102766	-2.611799	0.858011
H	4.526961	-1.887828	1.288563
H	4.697498	1.482900	1.098660
H	6.202768	2.081885	1.834227
H	6.470449	-3.055763	3.096709
H	4.897055	-2.268627	3.526454
F	2.710025	-1.618160	3.937253

N	6.449451	0.559457	0.417938

Complex-2-conf5

G_{sol} = -1346.4053710 Hartree

C	1.385448	0.253358	-0.741143
C	1.691366	1.242031	0.181950
C	1.962642	0.873705	1.518503
C	1.904906	-0.482054	1.933772
C	1.612700	-1.456360	0.984757
C	1.356993	-1.095258	-0.345411
H	1.168614	0.525300	-1.775768
H	1.722786	2.296932	-0.099817
C	2.110074	-0.832286	3.385375
H	1.574588	-2.504497	1.287043
H	1.124002	-1.870683	-1.078129
C	2.624742	0.299048	4.207459
C	2.649463	1.580497	3.722057
H	1.122991	-1.117601	3.814005
H	2.939115	0.094077	5.232657
H	2.958015	2.439226	4.319777
H	2.336267	2.810068	2.118904
N	2.298517	1.839703	2.439369
C	-2.059420	-1.419294	2.448490
C	-2.107598	-0.255948	4.529175
C	-3.764826	0.938702	2.532914
C	-1.719078	-0.131161	1.655513
N	-2.258383	1.061512	2.418174
C	-1.662961	1.050826	3.817926
C	-4.091841	-0.421376	3.208822
C	-1.855460	2.357587	1.780806
Cl	-2.344740	2.476502	0.075349
H	-1.132622	-1.912529	2.774210
H	-2.610276	-2.115525	1.801486
H	-1.220251	-0.802840	4.877043
H	-2.729296	-0.015088	5.402421
H	-4.168865	1.020726	1.517704
H	-4.100419	1.799856	3.124396
H	-0.643058	0.032209	1.551658

H	-2.179338	-0.108697	0.662704
H	-0.575690	1.114903	3.693370
H	-2.023611	1.956214	4.322480
H	-4.726198	-0.250985	4.089667
H	-4.641645	-1.065965	2.509337
H	-0.764130	2.432747	1.832594
H	-2.332686	3.170317	2.339546
F	2.927726	-1.946700	3.515388
N	-2.869773	-1.107211	3.621980

Complex-2-conf6

G_{sol} = -1346.4046566 Hartree

C	-0.977903	-1.320430	-0.353459
C	-1.146728	-0.216890	0.465810
C	-0.571028	-0.215124	1.764393
C	0.164376	-1.338291	2.238773
C	0.289409	-2.439761	1.411285
C	-0.257268	-2.433791	0.110746
H	-1.404899	-1.324059	-1.357935
H	-1.712886	0.656561	0.134348
C	0.900534	-1.265508	3.547360
H	0.850744	-3.310867	1.755812
H	-0.129783	-3.307971	-0.529767
C	0.538805	-0.068505	4.360593
C	-0.218522	0.935977	3.847842
H	0.779211	-2.195983	4.124031
H	0.936267	0.014925	5.373165
H	-0.465145	1.841966	4.402706
H	-1.277675	1.653540	2.234503
N	-0.732032	0.862622	2.579425
C	3.502215	-2.413601	0.294688
C	2.512659	-0.266757	-0.051881
C	3.974186	-1.345861	-2.323209
C	4.895425	-1.835635	-0.074450
N	4.705167	-0.760474	-1.129016
C	3.851375	0.351697	-0.537393
C	2.584199	-1.849980	-1.849307
C	6.013970	-0.146548	-1.533236

Cl	7.127102	-1.317723	-2.274960
H	3.334394	-2.324758	1.375745
H	3.441316	-3.473087	0.012017
H	2.432362	-0.176218	1.038653
H	1.667515	0.257854	-0.517496
H	4.603631	-2.151826	-2.715127
H	3.901854	-0.546987	-3.071523
H	5.393122	-1.350468	0.775133
H	5.564280	-2.589637	-0.502957
H	4.427804	0.796586	0.282681
H	3.711498	1.095720	-1.330834
H	1.782782	-1.286574	-2.346180
H	2.466174	-2.913652	-2.094977
H	6.486062	0.262015	-0.633036
H	5.800497	0.643699	-2.261875
F	2.297979	-1.184974	3.266064
N	2.460549	-1.675908	-0.408576

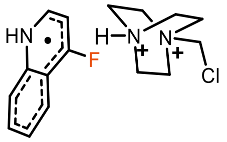

Complex-3

G_{sol} = -1346.4229452 Hartree

C	1.446220	0.515563	-0.507857
C	1.858035	1.340071	0.553303
C	1.965937	0.823336	1.847434
C	1.649015	-0.555096	2.096340
C	1.240515	-1.363314	1.011094
C	1.142379	-0.828503	-0.278530
H	1.365591	0.935798	-1.512801
H	2.098709	2.391725	0.379703
C	1.747912	-1.014040	3.445045
H	1.000035	-2.410623	1.199692
H	0.822997	-1.467483	-1.105137
C	2.178362	-0.177690	4.490311
C	2.481437	1.134187	4.209719
H	-0.446179	-0.287893	3.030038

H	2.265624	-0.566166	5.505746
H	2.813670	1.849076	4.961307
H	2.591472	2.584153	2.736771
N	2.360952	1.612314	2.919230
C	-1.701399	0.459541	1.551342
C	-1.494394	1.311464	3.846869
C	-3.749335	-0.275078	3.602982
C	-3.195578	0.795473	1.444441
N	-3.747479	1.033336	2.836949
C	-2.828752	2.009700	3.551394
C	-2.377260	-0.945054	3.450212
C	-5.123318	1.648980	2.798516
Cl	-6.277851	0.663682	1.884053
H	-1.056504	1.306931	1.289160
H	-1.439893	-0.396553	0.920062
H	-0.645743	1.963961	3.610061
H	-1.415513	0.975294	4.887497
H	-4.546187	-0.904063	3.191799
H	-3.984258	-0.026967	4.645473
H	-3.357315	1.710092	0.861605
H	-3.774709	-0.022951	1.003477
H	-2.709915	2.874709	2.887631
H	-3.323602	2.324590	4.477640
H	-2.016278	-1.334889	4.409282
H	-2.382753	-1.751743	2.707267
H	-5.032313	2.630144	2.319421
H	-5.470810	1.739922	3.834388
F	1.464431	-2.312200	3.699428
N	-1.405832	0.094597	2.975326

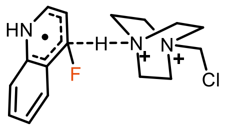

AfterTS’

G_{sol} = -1346.407456 Hartree

C	-4.400774	0.915547	-0.750279
C	-3.867794	1.804354	0.173593

C	-2.540386	2.246850	0.022615
C	-1.746561	1.778863	-1.057337
C	-2.312848	0.894227	-1.988103
C	-3.628874	0.467857	-1.839274
H	-5.428181	0.565355	-0.629149
H	-4.461296	2.166598	1.016443
C	-0.322510	2.158220	-1.083944
H	-1.701430	0.542076	-2.820715
H	-4.061436	-0.224965	-2.564180
C	0.057277	3.306914	-0.289379
C	-0.768271	3.724322	0.722115
H	0.222110	1.148143	-0.490026
H	1.030263	3.775901	-0.441648
H	-0.497327	4.521198	1.415965
H	-2.579431	3.512951	1.659723
N	-1.995405	3.169236	0.899335
C	-0.207399	-1.059683	0.335479
C	1.719418	0.185657	1.093841
C	2.408094	-1.861430	-0.744696
C	0.448068	-2.422188	0.659860
N	1.955421	-2.256057	0.646984
C	2.325814	-1.140207	1.609826
C	1.616971	-0.612868	-1.189393
C	2.654869	-3.502402	1.117478
Cl	2.270183	-4.921359	0.122402
H	-0.744379	-0.658603	1.204391
H	-0.915149	-1.166144	-0.495215
H	1.130473	0.670367	1.882782
H	2.505281	0.877306	0.765371
H	2.228787	-2.718990	-1.401625
H	3.487179	-1.674382	-0.678600
H	0.174646	-2.788261	1.656955
H	0.206534	-3.187147	-0.085670
H	1.929685	-1.430399	2.590900
H	3.420684	-1.102930	1.656690
H	2.298430	0.174143	-1.534830
H	0.917034	-0.852612	-1.999373
H	2.334328	-3.696689	2.147063

H	3.733702	-3.318054	1.067220
F	0.266777	2.082962	-2.332022
N	0.837421	-0.093679	-0.051574

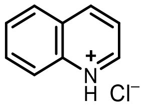

Q-HCl

G_{sol} = -862.5545228 Hartree

C	-5.223718	2.113417	2.685724
C	-3.842223	2.096343	2.688460
C	-3.146365	3.323834	2.675296
C	-3.848953	4.567506	2.659331
C	-5.270442	4.541214	2.657009
C	-5.941913	3.336926	2.669984
H	-1.270443	2.472402	2.689081
H	-5.772184	1.168760	2.695757
H	-3.284186	1.157397	2.700449
C	-3.094949	5.765939	2.646084
H	-5.809572	5.490813	2.644853
H	-7.033835	3.317553	2.668196
C	-1.710110	5.729527	2.649298
C	-1.062677	4.491839	2.664678
H	-3.570926	6.753352	2.629967
H	-1.160447	6.672511	2.635341
H	0.023247	4.382463	2.667907
N	-1.773980	3.361706	2.677071
Cl	-2.477365	9.079066	2.543538

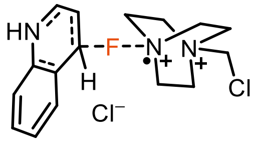

TS

G_{sol} = -1806.7364849 Hartree

C	-2.476406	-1.249375	-0.184751
C	-2.506368	-0.383317	0.896598
C	-1.505832	-0.481249	1.886637

C	-0.471080	-1.444395	1.774462
C	-0.476537	-2.324584	0.671774
C	-1.466576	-2.230439	-0.295483
H	-3.249750	-1.176719	-0.953318
H	-3.293296	0.367831	1.000956
C	0.602079	-1.445224	2.752519
H	0.316920	-3.075472	0.608673
H	-1.467033	-2.913953	-1.147696
C	0.382481	-0.707409	3.955423
C	-0.651474	0.198981	4.015799
H	1.243474	-2.330015	2.763944
H	1.072118	-0.794137	4.795117
H	-0.822906	0.843123	4.880166
H	-2.286891	1.005995	3.090548
N	-1.534172	0.326450	3.003111
C	4.397920	-1.286566	1.180281
C	3.879809	0.761524	0.015619
C	3.767374	-1.461670	-1.713080
C	5.406916	-1.781525	0.113089
N	5.130171	-1.041281	-1.187972
C	5.100792	0.456084	-0.903294
C	2.733063	-1.284888	-0.569969
C	6.208299	-1.282556	-2.205810
Cl	6.433138	-3.007960	-2.561788
H	4.877172	-0.642822	1.927892
H	3.918710	-2.163190	1.643535
H	4.192527	1.370678	0.873105
H	3.084532	1.278362	-0.535431
H	3.847450	-2.507760	-2.028709
H	3.557592	-0.826610	-2.582968
H	6.448680	-1.580272	0.391266
H	5.268099	-2.848926	-0.090551
H	6.054277	0.703024	-0.421209
H	5.028282	0.974668	-1.866744
H	1.850401	-0.738528	-0.924061
H	2.450524	-2.261605	-0.148699
H	7.145160	-0.880139	-1.803997
H	5.918674	-0.766139	-3.127931

F	1.828075	-0.423092	1.971881
N	3.391193	-0.522060	0.472784
Cl	2.653540	-4.200524	1.250475

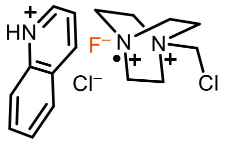

Complex-1-Cl

G_{sol} = -1806.7520883 Hartree

C	-1.718038	0.958971	-0.098281
C	-1.501206	1.105509	1.257782
C	-0.920104	0.037416	1.975656
C	-0.560755	-1.173730	1.320479
C	-0.799625	-1.289328	-0.078100
C	-1.370422	-0.241897	-0.769357
H	-2.167058	1.780687	-0.661282
H	-1.767072	2.026596	1.781770
C	0.059909	-2.196230	2.073837
H	-0.479669	-2.216768	-0.565272
H	-1.550261	-0.327974	-1.843365
C	0.257387	-2.032902	3.433052
C	-0.125842	-0.834714	4.040328
H	0.438640	-3.078749	1.534129
H	0.754267	-2.798624	4.029601
H	0.018328	-0.622707	5.100464
H	-0.925377	1.009931	3.785306
N	-0.679588	0.135724	3.319555
C	4.600953	-0.054205	0.937638
C	2.633795	-0.404049	-0.398673
C	4.296415	-2.708106	-0.284319
C	5.545198	-0.566882	-0.183831
N	4.762328	-1.507011	-1.086266
C	3.535573	-0.771651	-1.606855
C	3.510137	-2.202005	0.948582
C	5.569811	-1.924308	-2.280354
Cl	7.070534	-2.764945	-1.833230
H	4.412923	1.021572	0.836749

H	5.026425	-0.254040	1.928287
H	2.414797	0.670232	-0.381894
H	1.693615	-0.959751	-0.435134
H	5.186443	-3.286984	-0.017935
H	3.628474	-3.301084	-0.926055
H	5.925888	0.245165	-0.815335
H	6.389106	-1.141581	0.212699
H	3.900900	0.112309	-2.143461
H	3.026128	-1.448684	-2.302881
H	2.530837	-2.689468	0.927966
H	4.035273	-2.409656	1.887881
H	5.829629	-1.020747	-2.843300
H	4.952411	-2.602381	-2.879481
F	2.251421	-0.172534	2.402772
N	3.343434	-0.766056	0.811755
Cl	1.122593	-4.144782	-0.616188

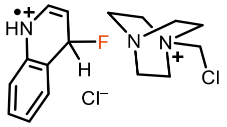

Complex-2-Cl-conf1

G_{sol} = -1806.760766 Hartree

C	1.053080	-0.245559	-0.822937
C	1.426106	0.742436	0.078197
C	1.540103	0.427036	1.445416
C	1.284012	-0.888922	1.902392
C	0.911634	-1.870637	0.974390
C	0.789381	-1.554189	-0.376290
H	0.961507	-0.002065	-1.883847
H	1.622455	1.765008	-0.252764
C	1.493209	-1.204315	3.330725
H	0.722192	-2.884998	1.329197
H	0.494415	-2.324560	-1.092219
C	1.617370	-0.063555	4.219875
C	1.859923	1.180948	3.707717
H	2.574879	-1.792392	3.343931
H	1.597407	-0.222376	5.298892

H	2.040441	2.053038	4.337603
H	2.025075	2.349106	2.030006
N	1.860360	1.403962	2.368493
C	-1.753734	1.086684	1.564058
C	-1.583805	1.564554	3.896797
C	-3.531542	-0.452445	3.221601
C	-3.277751	1.381766	1.579043
N	-3.821337	1.011187	2.946563
C	-3.107137	1.850247	3.995842
C	-1.996618	-0.668619	3.161864
C	-5.284853	1.314245	3.063098
Cl	-6.276714	0.411212	1.893615
H	-1.196863	2.003580	1.327799
H	-1.521002	0.339585	0.794104
H	-1.043875	2.492239	3.662434
H	-1.209834	1.183424	4.856464
H	-4.072440	-1.028277	2.462949
H	-3.955423	-0.673179	4.209421
H	-3.510011	2.444039	1.430660
H	-3.833659	0.787074	0.846186
H	-3.358119	2.897469	3.786418
H	-3.531137	1.562858	4.966175
H	-1.626440	-1.053210	4.120333
H	-1.748587	-1.406329	2.387621
H	-5.423343	2.385545	2.880720
H	-5.604166	1.039655	4.074532
F	0.638642	-2.193753	3.804609
N	-1.311597	0.581774	2.857509
Cl	3.841001	-3.011842	3.216908

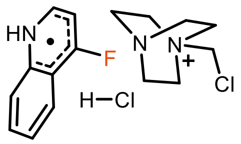

Complex-3-Cl

G_{sol} = -1806.7651881 Hartree

C	1.542914	-0.086598	-0.493071
C	1.933858	0.737282	0.574270

C	1.869280	0.265190	1.888099
C	1.402080	-1.062373	2.147025
C	1.010577	-1.869610	1.060687
C	1.081557	-1.383289	-0.249970
H	1.599024	0.295908	-1.514985
H	2.282968	1.756960	0.392227
C	1.370934	-1.485857	3.510931
H	0.651928	-2.880533	1.260738
H	0.774262	-2.021548	-1.081979
C	1.666747	-0.609550	4.570401
C	2.116276	0.655806	4.276053
H	3.499687	-1.798099	3.333884
H	1.566402	-0.941066	5.604513
H	2.401377	1.383810	5.034435
H	2.572726	1.996501	2.771946
N	2.239350	1.057516	2.963874
C	-1.487606	0.863976	1.613275
C	-1.507090	1.892063	3.767567
C	-3.303775	-0.345342	3.493166
C	-3.021148	1.053023	1.469206
N	-3.642086	0.987742	2.852288
C	-3.046057	2.098792	3.704546
C	-1.758593	-0.471547	3.572001
C	-5.121353	1.218204	2.803619
Cl	-5.984820	-0.003810	1.839717
H	-0.960388	1.732212	1.195991
H	-1.161751	-0.026227	1.060270
H	-0.995063	2.769670	3.349568
H	-1.188481	1.772488	4.812214
H	-3.762433	-1.120084	2.869369
H	-3.785454	-0.346736	4.479266
H	-3.297208	2.030316	1.053101
H	-3.493031	0.263667	0.874065
H	-3.330663	3.045386	3.228900
H	-3.526743	2.029089	4.688073
H	-1.441962	-0.590001	4.617073
H	-1.424138	-1.357402	3.016993
H	-5.294323	2.202065	2.353734

H	-5.501685	1.182482	3.830335
F	0.814221	-2.692997	3.784780
N	-1.115750	0.710294	3.013800
Cl	4.775611	-1.699459	3.050617

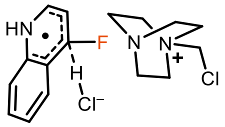

AfterTS

G_{sol} = -1806.761332 Hartree

C	2.038301	-0.499902	-3.244059
C	2.425549	0.463683	-2.320266
C	2.526963	0.122418	-0.959855
C	2.239894	-1.197819	-0.530732
C	1.848226	-2.153268	-1.480147
C	1.742025	-1.809737	-2.825264
H	1.960627	-0.233696	-4.300590
H	2.642242	1.488377	-2.631668
C	2.442821	-1.534538	0.883962
H	1.630517	-3.168900	-1.145146
H	1.435354	-2.560072	-3.557361
C	2.583593	-0.432356	1.803812
C	2.854341	0.818514	1.322246
H	3.623594	-2.119357	0.855908
H	2.558438	-0.619541	2.877962
H	3.054191	1.670768	1.972836
H	3.051745	2.020999	-0.330398
N	2.867287	1.072568	-0.014013
C	-0.780355	0.772086	-0.879656
C	-0.610722	1.334495	1.433616
C	-2.538173	-0.725689	0.838123
C	-2.307419	1.050218	-0.871370
N	-2.844133	0.723991	0.509951
C	-2.137060	1.608128	1.526813
C	-1.000753	-0.927499	0.780573
C	-4.310152	1.016843	0.617990
Cl	-5.295461	0.062080	-0.515499

H	-0.233573	1.685667	-1.149542
H	-0.540960	0.000658	-1.623160
H	-0.079671	2.258084	1.165188
H	-0.231172	0.991461	2.405530
H	-3.075735	-1.335066	0.103742
H	-2.956755	-0.912764	1.835215
H	-2.552112	2.103533	-1.058529
H	-2.857820	0.422588	-1.580529
H	-2.400247	2.644236	1.279697
H	-2.556618	1.352208	2.507876
H	-0.624566	-1.274214	1.751705
H	-0.747314	-1.689532	0.032167
H	-4.459008	2.079323	0.396299
H	-4.625616	0.777294	1.639536
F	1.655817	-2.578202	1.337612
N	-0.330898	0.318336	0.429838
Cl	4.914772	-3.086403	0.709583

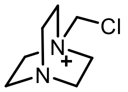

TEDA⁺

G_{sol} = -844.4148141 Hartree

C	-1.530491	-0.951546	1.200155
C	-2.203272	0.993668	-0.009558
C	-0.055082	-0.477606	-1.236041
C	-0.055921	-0.465874	1.240864
N	0.209082	0.362970	-0.001431
C	-0.750236	1.543410	-0.007058
C	-1.527373	-0.969305	-1.187962
C	1.599375	0.923995	-0.003777
Cl	2.856143	-0.335917	0.004823
H	-2.066198	-0.587632	2.087860
H	-1.560313	-2.049894	1.206966
H	-2.739897	1.362548	0.875451
H	-2.731739	1.348224	-0.905263
H	0.668807	-1.299501	-1.220556
H	0.153155	0.168264	-2.098494

H	0.157038	0.186069	2.097534
H	0.664345	-1.291017	1.230669
H	-0.521833	2.137033	0.886653
H	-0.517443	2.132915	-0.902351
H	-2.063673	-0.623151	-2.082385
H	-1.552565	-2.067722	-1.175521
H	1.716898	1.537789	0.896339
H	1.718166	1.526880	-0.911114
N	-2.207565	-0.465144	0.001553

HCl

G_{sol} = -460.7836159 Hartree

Cl	1.332203	3.880577	-0.847005
H	1.939158	2.769722	-0.574844

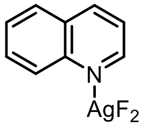

Quinoline·AgF$_2$

G_{sol} = -748.4712471 Hartree

C	-5.232897	2.144959	2.841620
C	-3.852356	2.149803	2.889438
C	-3.152503	3.378756	2.786020
C	-3.888175	4.603913	2.642320
C	-5.308120	4.554460	2.596368
C	-5.968256	3.348209	2.691732
H	-5.769159	1.196075	2.921602
H	-3.276179	1.228657	3.013893
C	-3.164598	5.820258	2.553021
H	-5.859035	5.491647	2.484041
H	-7.059657	3.314713	2.654278
C	-1.787624	5.805581	2.599825
C	-1.122929	4.567172	2.728627
H	-3.714268	6.759477	2.447705
H	-1.198379	6.721612	2.535063
H	-0.033046	4.490554	2.755825
N	-1.781898	3.421691	2.819259

Ag	-0.448713	1.634293	3.005266
F	-1.688416	0.105299	3.286040
F	1.154896	2.784459	2.774933

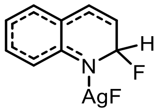

C2-fluoride Meisenheimer int. in lit.[28]

G_{sol} = -748.4659892 Hartree

C	-5.430652	2.255947	2.887433
C	-4.052792	2.221788	2.896019
C	-3.291371	3.432007	2.791086
C	-4.002602	4.686575	2.643658
C	-5.401667	4.681312	2.635850
C	-6.115304	3.488325	2.762576
H	-5.997686	1.326846	2.977342
H	-3.513169	1.276398	2.991888
C	-3.230668	5.894122	2.457612
H	-5.931224	5.630098	2.520940
H	-7.207462	3.503861	2.757328
C	-1.884561	5.851722	2.479325
C	-1.163014	4.580791	2.779534
H	-3.764868	6.832033	2.286269
H	-1.266428	6.742476	2.345288
H	-0.659772	4.668746	3.765421
N	-1.954094	3.376986	2.811883
Ag	-0.825439	1.606077	2.934488
F	0.245420	-0.070248	3.098664
F	-0.137835	4.388098	1.850619

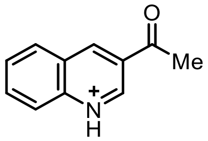

3-AcQN-H^{+}

C	-2.346361	-2.300268	0.189104
C	-0.969857	-2.366856	0.305520
C	-0.218126	-1.192264	0.112212

C	-0.855979	0.048451	-0.198595
C	-2.273421	0.075217	-0.309136
C	-3.001281	-1.080012	-0.118170
H	1.609380	-2.084731	0.437110
H	-2.940497	-3.204938	0.336610
H	-0.461613	-3.303942	0.542463
C	-0.047062	1.189303	-0.381651
H	-2.762386	1.022350	-0.546152
H	-4.089753	-1.062430	-0.202555
C	1.337747	1.119616	-0.266342
C	1.915843	-0.123196	0.041107
H	-0.506059	2.151785	-0.620266
H	2.989663	-0.277499	0.1517100
N	1.151524	-1.197621	0.214535
C	2.158358	2.368539	-0.476554
O	1.588278	3.411906	-0.732180
C	3.655231	2.267491	-0.362942
H	3.943295	1.907486	0.638627
H	4.047736	1.543017	-1.095848
H	4.100709	3.253685	-0.543185

Stern–Volmer luminescence quenching experiments

General Procedure

In the luminescence quenching experiment, [Ir(dFppy)$_3$] was chosen because it is an efficient photosensitizer for the fluorination reaction and it has a strong luminescence at room temperature. The visible light luminescence intensities were recorded using an Edinburgh Instruments FS5 spectrofluorometer. All luminescence measurements were recorded using a screw-top quartz cuvette (Hellma fluorescence quartz cuvette, 10 × 10 mm, 3.5 mL). All solutions of [Ir(dFppy)$_3$] and quenchers were prepared in MeCN, in a N_2-filled glovebox. The solutions were transferred to a screw-top cuvette, sealed, and then brought out of the glovebox for visible light luminescence measurements.

In a typical procedure, quinoline hydrochloride (33.0 mg, 0.200 mmol) was dissolved in 20.0 mL of stock solution (c = 18 μM [Ir(dFppy)$_3$] in MeCN) to the concentration (c = 10.0 mM quinoline hydrochloride). Next, 2.0 mL the quinoline hydrochloride solution (c = 10.0 mM quinoline hydrochloride) was further diluted with 18.0 mL of stock solution to the concentration (c = 1.00 mM quinoline hydrochloride). The solutions of concentrations (c = 0.75 mM quinoline hydrochloride), (c = 0.50 mM quinoline hydrochloride), (c = 0.25 mM quinoline hydrochloride), (c = 0.13 mM quinoline hydrochloride), and (c = 0.06 mM quinoline hydrochloride) were prepared by diluting respectively 7.5 mL, 5.0 mL, 2.5 mL, 1.3 mL, and 0.6 mL of the 1.0 mM quinoline hydrochloride solution with the stock solution (c = 18 μM [Ir(dFppy)$_3$] in MeCN) to a final volume of 10.0 mL. All solutions were excited at 400 nm and the emission was measured from 455 to 600 nm.

Quenching was analyzed by plotting I_0/I according to the Stern–Volmer relationship:

$$I_0/I = k_q\tau_0[Q]+1$$

where I_0 represents the integral of the luminescence over the range of 455 to 600 nm in the absence of a quencher, I is the integral of luminescence over the range of 455 to 600 nm in the presence of a quencher, k_q represents the quenching rate constant, [Q] is the concentration of a given quencher, and τ_0 is the excited state lifetime of the emissive photocatalyst in the absence of quencher. The excited state lifetime of [Ir(dFppy)$_3$] is 1640 ns[29].

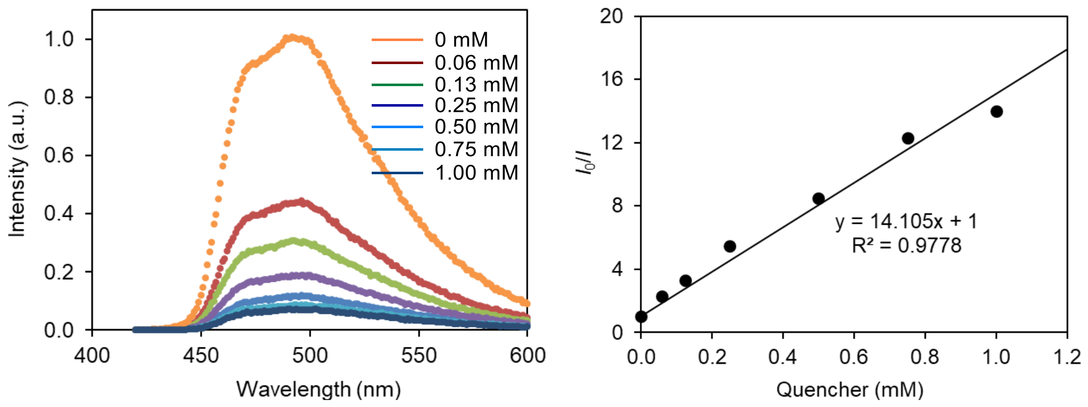

Figure S25. Luminescence spectra of [Ir(dFppy)$_3$] quenching by quinoline·HCl and Stern–Volmer plot.

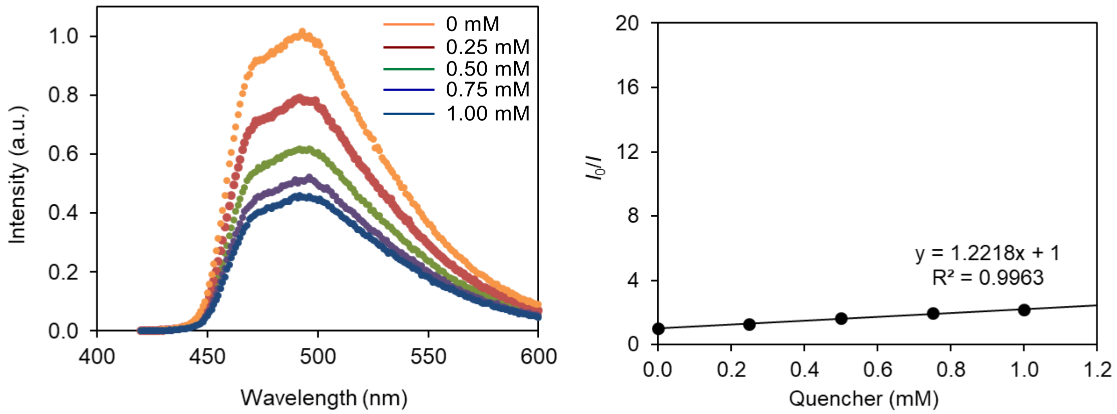

Figure S26. Luminescence spectra of [Ir(dFppy)$_3$] quenching by quinoline and Stern–Volmer plot.

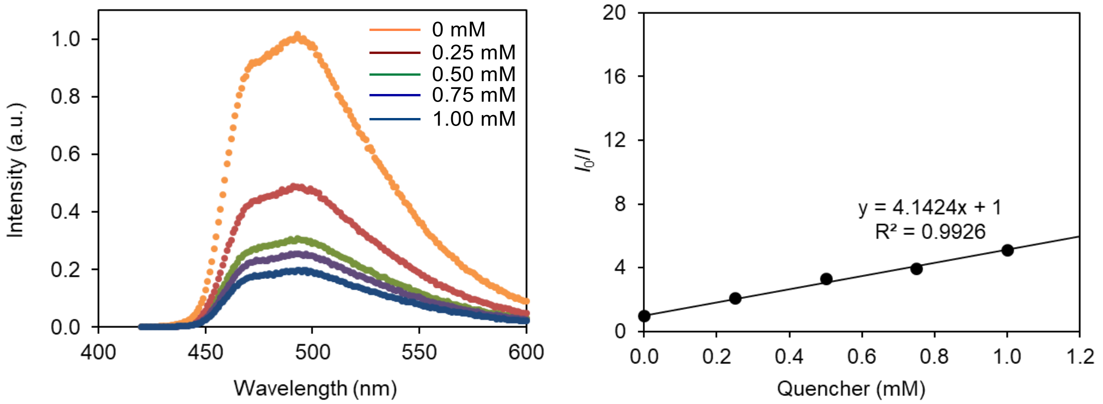

Figure S27. Luminescence spectra of [Ir(dFppy)$_3$] quenching by Selectfluor and Stern–Volmer plot.

Table S5. Quenching rate coefficients

**Quencher**	**k_q/(M^{-1}.s^{-1})**
Quinoline·HCl	8.6 x 10^9
Quinoline	7.4 x 10^8
Selectfluor	2.5 x 10^9

Intermolecular radical-trapping experiment

1-Chlorododecane

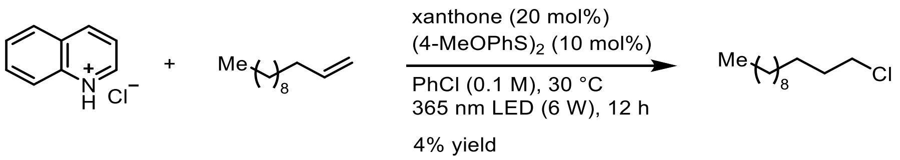

Under a nitrogen atmosphere, to a 4-mL vial equipped with a magnetic stir bar were added quinoline hydrochloride (16.7 mg, 0.100 mmol, 1.00 equiv.), xanthone (3.9 mg, 20 μmol, 20 mol%), bis(4-methoxylphenyl) disulfide (2.8 mg, 10 μmol, 10 mol%), and dry PhCl (1.0 mL, *c* = 0.1 M). The vial was sealed with a septum cap. Then the vial was placed onto an aluminum plate fitted with a 6 W high-power single LED plate (EpiLED Chip, size: 10 × 10 mm, λ_{max} = 365 nm, 600 mA, 9–11 V). Another aluminum block was utilized to hold the vial. The temperature was kept at approximately 30 °C through the use of a cooling fan. The reaction mixture was stirred and irradiated for 12 h. After that, *n*-tetradecane (26 μL, 20 mg, 0.10 mmol, 1.0 equiv.) was added as an internal standard, followed by 1 mL of saturated Na_2CO_3. An aliquot of the organic phase was taken and diluted with MeCN before analysis by GC-FID/MS. The production of 1-chlorododecane was confirmed by the comparison with an authentic sample, and the yield (4%) was determined by the integration relative to the internal standard.

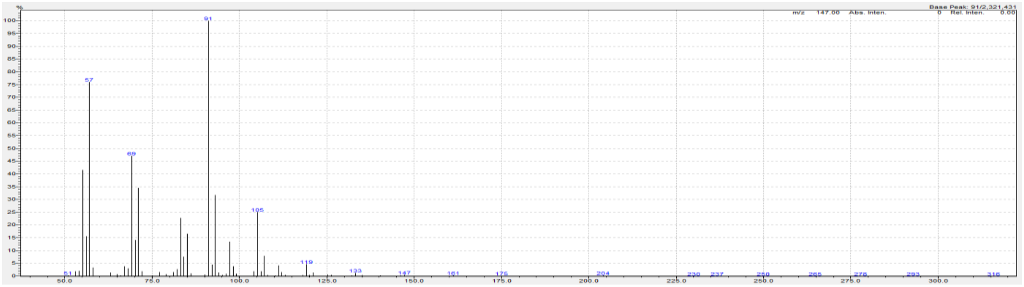

Figure S28. The MS spectrum of the hydrochlorination product.

Discussion: The chlorine radical trapping experiment condition is different from the fluorination reaction by adding HAT catalyst and changing to another solvent. When alkene was employed as radical acceptor for chlorine radical trapping experiment, a HAT catalyst is required to ensure efficient termination of the secondary alkyl radical generated from chlorine radical addition[30]. In the fluorination reaction, the generated chlorine radical was trapped by acetonitrile solvent. However, in the radical-trapping experiment, chlorobenzene was chosen as the reaction media. Despite the HAT catalyst and the reaction solvent, the intermolecular radical-trapping experiment shows excitation of an ion pair quinoline-H^+Cl^- followed by reductive quenching of its counteranion Cl^- can produce chlorine radical unambiguously.

Discussion of C–H cleavage by chlorine radical and TEDA$^{2+•}$

In the proposed initiation mechanism (Figure 2a), the reductive quench of the excited-state protonated quinoline by counter anion Cl^- produces a chlorine radical[30]. It was reported that the chlorine radical could cleave an aliphatic C–H bond via HAT mechanism[31], and we proposed if the C–H bond could be cleaved by chlorine radical in our reaction, the fluorination should work in the presence of F^- and oxidant via a non-chain mechanism (Figure S30, up). However, a variety of oxidants and fluoride sources were tested in the absence of Selectfluor but the fluorination product was not observed (Figure S30, bottom), which indicates the important role of TEDA$^{2+•}$.

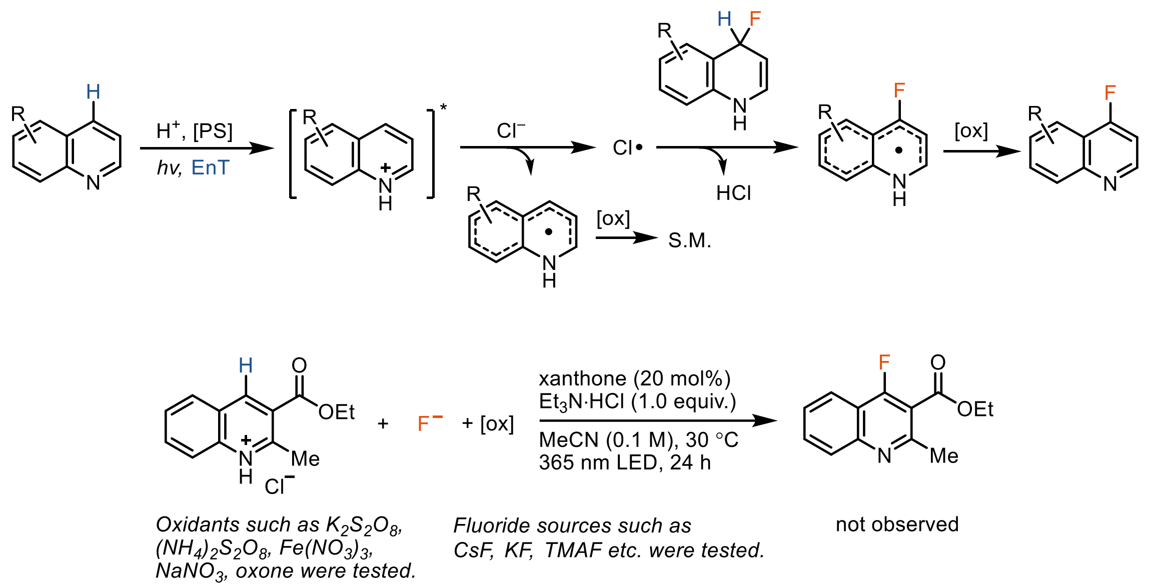

Figure S29. C–H cleavage by chlorine radical and fluorination with different oxidants and fluoride sources

Quantum yield measurement

Photon flux measurement

The photon flux of a 365 nm LED was determined by standard ferrioxalate actinometry[32-34]. A 0.15 M solution of ferrioxalate was prepared by dissolving potassium ferrioxalate hydrate (328 mg, 0.750 mmol) in 5.0 mL of 0.20 M aqueous sulfuric acid. A 0.15 M buffered solution of 1,10-phenanthroline was prepared by dissolving 1,10-phenanthroline (541 mg, 3.00 mmol) and sodium acetate (1.23 g, 15.0 mmol) in 20 mL of 0.20 M aqueous sulfuric acid.

To a 4-mL borosilicate vial equipped with a stir bar was added 1.0 mL of the ferrioxalate solution. The vial was sealed and placed on the aluminum plate fitted with a 6 W high-power single LED plate. After irradiation for 10 seconds, 1.5 mL of 0.20 M aqueous sulfuric acid and 2.0 mL of the buffered solution was added to the vial. The solution was then allowed to rest for 1 hour to allow the resultant ferrous ions to react completely with 1,10-phenanthroline. 25 μL of the resulting solution was taken as an aliquot and diluted with 3.0 mL of 0.20 M aqueous sulfuric acid. The absorbance of the resulting solution in a cuvette (l = 1.0 cm) at 510 nm was measured by UV-Vis spectrometer. A non-irradiated sample and other samples with different irradiation time were also prepared and the absorbance at 510 nm was measured.

The amount of ferrous ion formed was calculated as follows:

$$\text{mol Fe}^{2+} = (V \times \Delta A) / (l \times \varepsilon)$$

where V is the total volume (0.96 L) of the solution that was analyzed, ΔA is the difference in absorbance at 510 nm between the irradiated and non-irradiated samples, l is the path length (1.00 cm), and ε is the molar absorptivity at 510 nm (11,100 $L \cdot mol^{-1} \cdot cm^{-1}$)[33].

The photon flux was calculated as follows:

$$\text{photon flux} = (\text{mol Fe}^{2+}) / (\Phi \times t \times f)$$

where Φ is the quantum yield for the ferrioxalate actinometer (approximated as 1.26, which was reported for a 0.15 M solution at λ = 365 nm)[32], t is the time, and f is the fraction of light absorbed at 365 nm (>0.999).

$$f = 1.000 - 10^{-A} = 0.999$$

where A is the measured absorbance (>3) of the 0.15 M solution of potassium ferrioxalate at 365 nm

Table S6. Calculation of photon flux

Irradiation time [s]	Absorbance	ΔA	mol Fe^{2+} [mol]	Photon flux [$Einstein \cdot s^{-1}$]
0	0.034	–	–	–
10	0.109	0.075	6.5×10^{-6}	5.1×10^{-7}
20	0.168	0.134	1.15×10^{-5}	4.5×10^{-7}
30	0.239	0.205	1.77×10^{-5}	4.7×10^{-7}

The average photon flux is 4.8×10^{-7} $Einstein \cdot s^{-1}$.

Quantum yield of azaarene fluorination

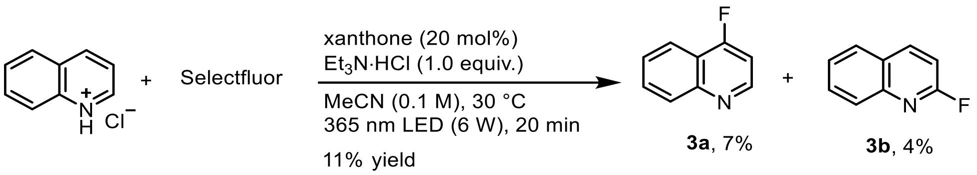

Under a nitrogen atmosphere, to a 4-mL vial equipped with a magnetic stir bar were added quinoline hydrochloride (16.6 mg, 0.100 mmol, 1.00 equiv.), xanthone (3.9 mg, 20 μmol, 20 mol%), Selectfluor (106.2 mg, 0.300 mmol, 3.00 equiv.), $Et_3N·HCl$ (13.7 mg, 0.100 mmol, 1.00 equiv.), and dry acetonitrile (1.0 mL, *c* = 0.1 M). The vial was sealed with a septum cap. Then the vial was placed onto an aluminum plate fitted with a 6 W high-power single LED plate (size: 10 × 10 mm, λ_{max} = 365 nm, 600 mA, 9–11 V). Another aluminum block was utilized to hold the vial. The temperature was kept at approximately 30 °C through the use of a cooling fan. The reaction mixture was stirred and irradiated for indicated time. After that, 2-fluorotoluene (11 μL, 0.10 mmol, 1.0 equiv.) was added as an internal standard, followed by 1 mL of saturated aqueous Na_2CO_3. An aliquot (0.1 mL) of the organic phase was taken and diluted with $CDCl_3$ (0.5 mL), and the yield (11%, 1.1×10^{-5} mol, **3a**:**3b** = 1.8:1) was determined by ^{19}F NMR integration relative to the internal standard.

The quantum yield was calculated as follows:

$$\Phi = (\text{mol product}) / (\text{flux} \times t \times f)$$

where flux is the photon flux determined by ferrioxalate actinometry (4.8×10^{-7} Einstein·s^{-1}), t is the time (1200 s), and f is the fraction of light absorbed by xanthone at 365 nm.

A solution of xanthone (11.7 mg, 60 μmol,) in acetonitrile (3.0 mL) was prepared, and the absorbance of the solution at 365 nm was 0.267. The fraction of light absorbed at 365 nm was calculated as 0.540.

$$\Phi = (1.1 \times 10^{-5}\ \text{mol}) / (4.8 \times 10^{-7}\ \text{Einstein·s}^{-1} \times 1200\ \text{s} \times 0.540) = 3.5\%$$

Quantum yield of hydrochlorination

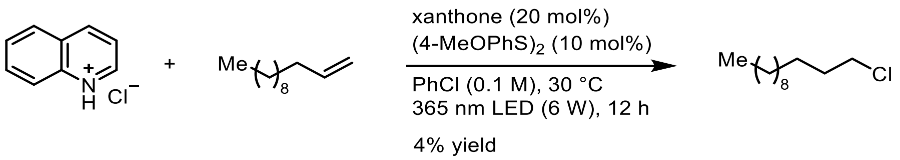

Under a nitrogen atmosphere, to a 4-mL vial equipped with a magnetic stir bar were added quinoline hydrochloride (16.7 mg, 0.100 mmol, 1.00 equiv.), xanthone (3.9 mg, 20 μmol, 20 mol%), bis(4-methoxylphenyl) disulfide (2.8 mg, 10 μmol, 10 mol%), and dry PhCl (1.0 mL, *c* = 0.1 M). The vial was sealed with a septum cap. Then the vial was placed onto an aluminum plate fitted with a 6 W high-power single LED plate (EpiLED Chip, size: 10 × 10 mm, λ_{max} = 365 nm, 600 mA, 9–11 V). Another aluminum block was utilized

to hold the vial. The temperature was kept at approximately 30 °C through the use of a cooling fan. The reaction mixture was stirred and irradiated for 12 h. After that, *n*-tetradecane (26 μL, 20 mg, 0.10 mmol, 1.0 equiv.) was added as an internal standard, followed by 1 mL of saturated aqueous Na_2CO_3. An aliquot of the organic phase was taken and diluted with MeCN before analysis by GC-FID/MS. The yield (4%, 4.0×10^{-6} mol) was determined by the integration relative to the internal standard.

The quantum yield was calculated as follows:

$$\Phi = (\text{mol product}) / (\text{flux} \times t \times f)$$

where flux is the photon flux determined by ferrioxalate actinometry (4.8×10^{-7} Einstein·s^{-1}), t is the time (1200 s), and f is the fraction of light absorbed by xanthone at 365 nm.

A solution of xanthone (11.7 mg, 60 μmol,) in acetonitrile (3.0 mL) was prepared, and the absorbance of the solution at 365 nm was 0.267. The fraction of light absorbed at 365 nm was calculated as 0.540.

$$\Phi = (4.0 \times 10^{-6}\ \text{mol}) / (4.8 \times 10^{-7}\ \text{Einstein}\cdot\text{s}^{-1} \times 43200\ \text{s} \times 0.540) = 0.04\%$$

Discussion

The quantum yield for fluorination of 3.5% is consistent with a back electron transfer process between the *N*-heterocyclic π-radical and chlorine radical. Although the Φ of the fluorination reaction is low, the large difference of Φ for the overall reaction and initiation process ($Cl^{\bullet}$ trapping, Φ = 0.04%) is consistent with a chain mechanism.

Electrochemistry study

General Procedure

A 20-mL vial was charged with the substrate (20.0 µmol, 1.00 equiv) and $TBAPF_6$ (581 mg, 1.50 mmol, 75.0 equiv). Dry acetonitrile (10.0 mL, c = 2.0 mM) was added via syringe, and the vial was swirled to ensure homogeneity. The resulting solution (3.0 mL) was added to a cuvette equipped with a magnetic stirring bar. A glassy-carbon working electrode (2 mm diameter), a platinum-wire counter electrode, and a Ag/AgCl quasi-reference electrode were immersed in the solution while not touching the stirring bar. The solution was purged with argon for 2 minutes with slow stirring. Then the stirring was stopped and a cyclic voltammogram was obtained at 298 K with an Autolab PGSTAT204 potentiostat.

For Selectfluor a sample with 4.0 mM substrate concentration was used to have the comparable current.

The quasi-reference electrode potential was calibrated by obtaining a cyclic voltammogram (CV) of a solution of ferrocene (2.0 mM) and $TBAPF_6$ (150 mM) in acetonitrile with 100 mV/s potential sweep rate. The standard redox potential of Fc/Fc^+ couple was taken as 310 mV vs SCE[36]. Potentials for irreversible waves were estimated at half the maximum current, as previously described by Nicewicz[37].

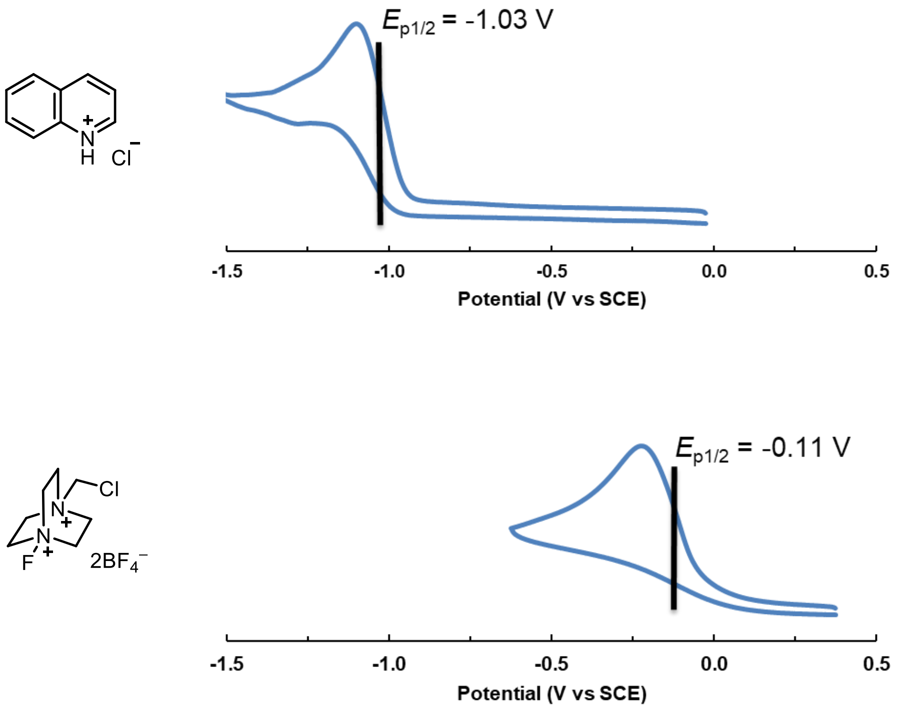

Figure S30. CVs of quinoline hydrochloride and Selectfluor. Y axis (intensity in µA) was omitted for clarity.

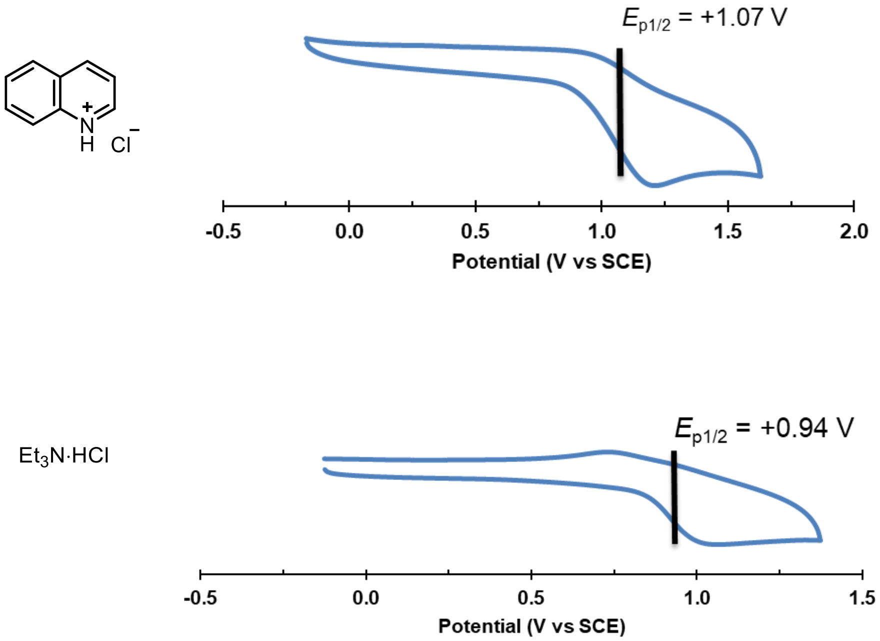

Figure S31. CVs of quinoline hydrochloride and $Et_3N{\cdot}HCl$. Y axis (intensity in μA) was omitted for clarity.

SPECTROSCOPIC DATA

^{1}H NMR of 2

CDCl$_3$, 500 MHz, 25 °C

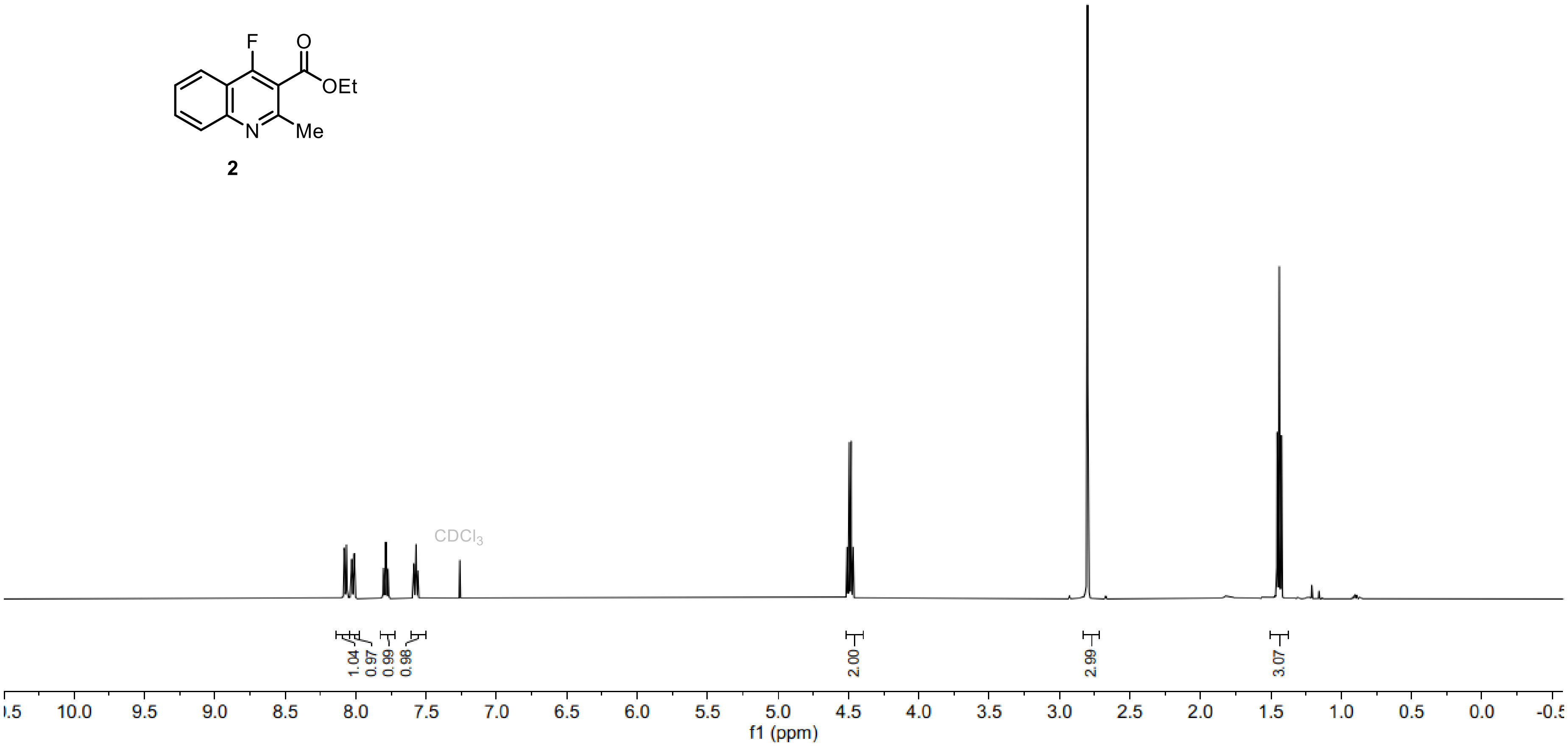

^{13}C NMR of 2

$CDCl_3$, 126 MHz, 25 °C

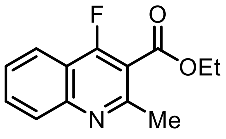

2

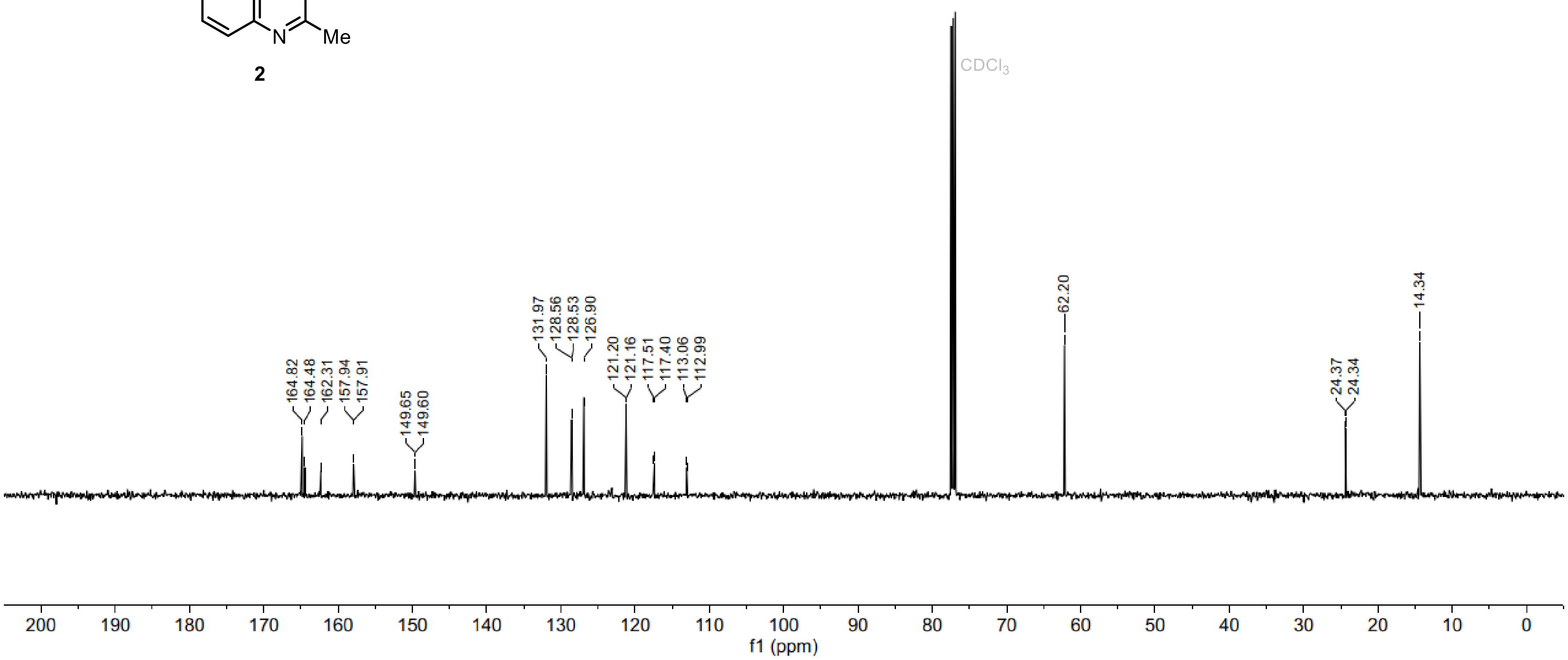

^{19}F NMR of 2

$CDCl_3$, 471 MHz, 25 °C

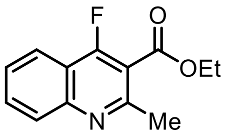

2

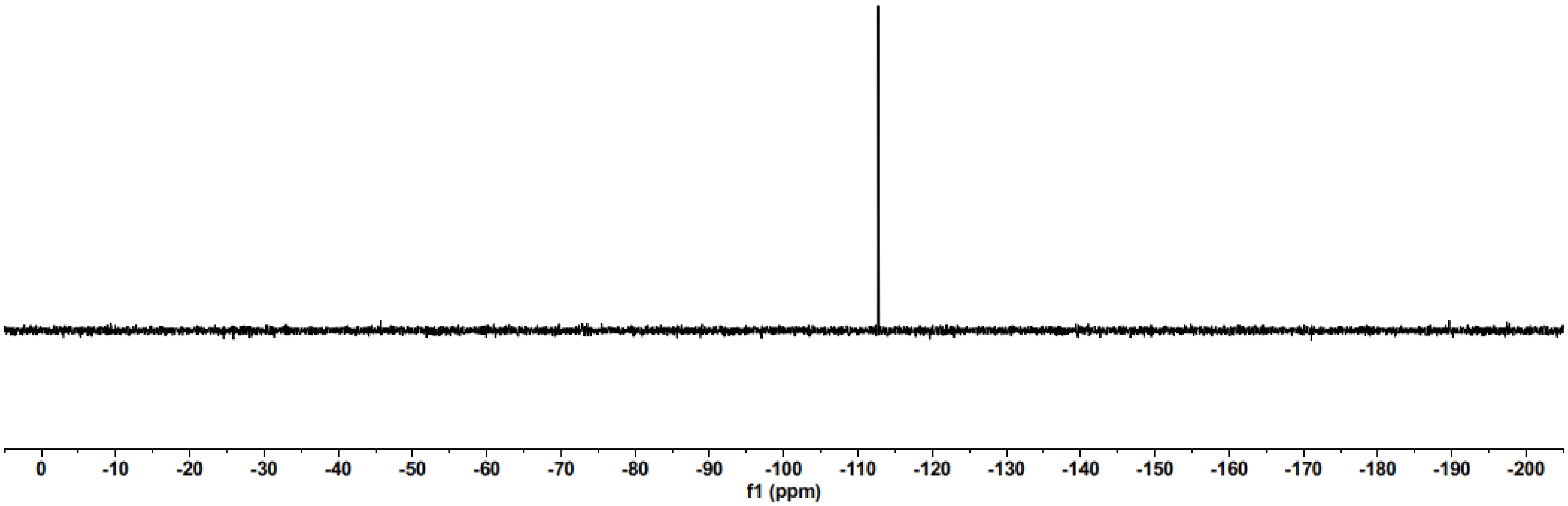

^{1}H NMR of 4a

CDCl$_3$, 500 MHz, 25 °C

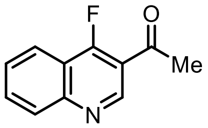

4a

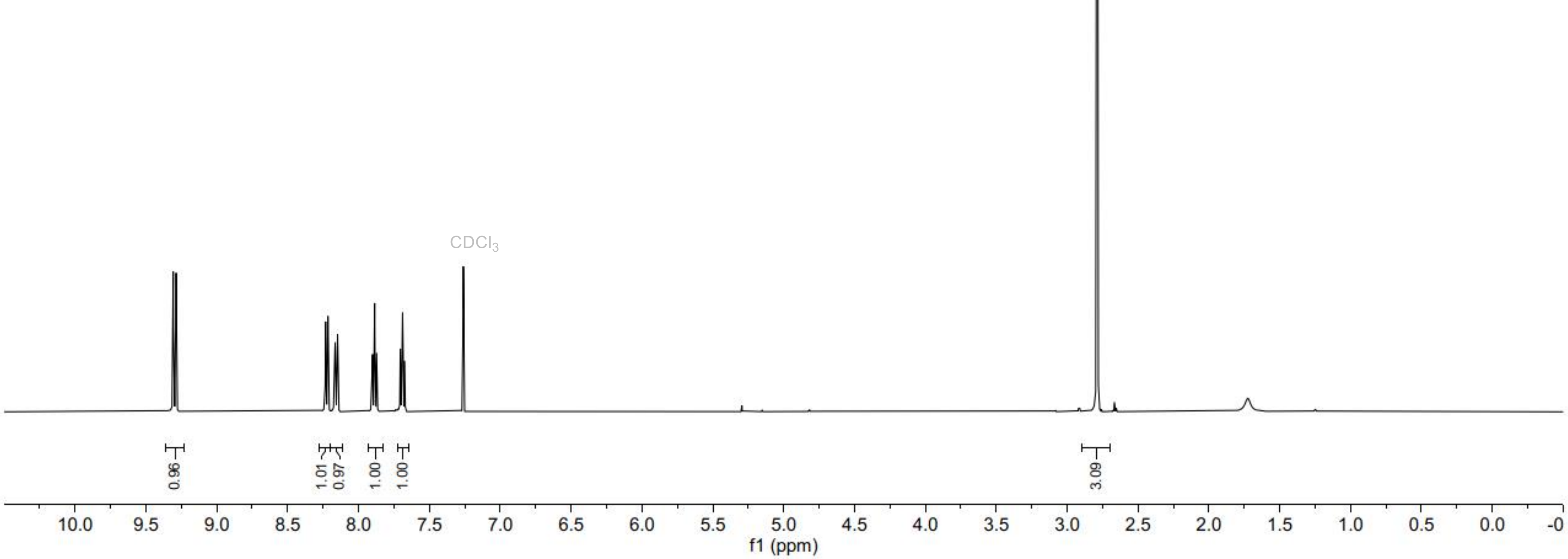

^{13}C NMR of 4a

$CDCl_3$, 126 MHz, 25 °C

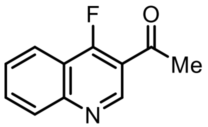

4a

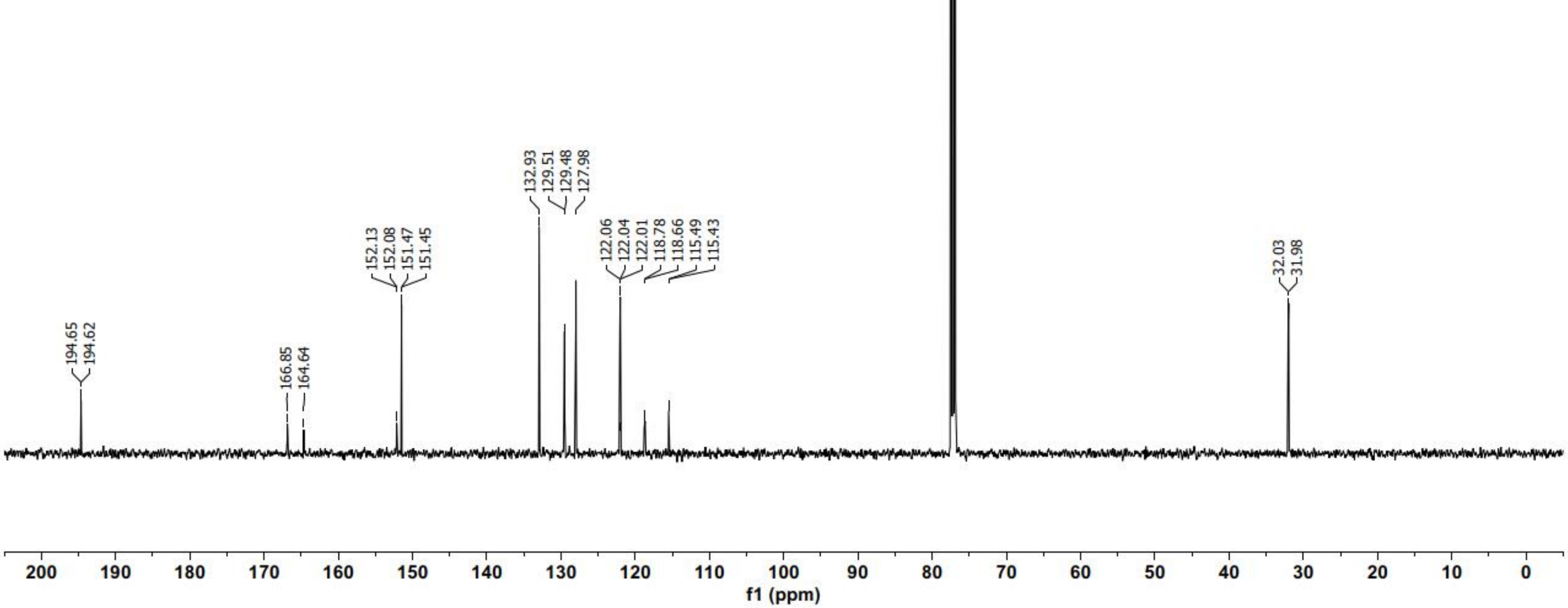

^{19}F NMR of 4a

CDCl$_3$, 471 MHz, 25 °C

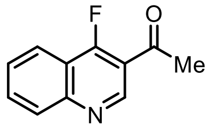

4a

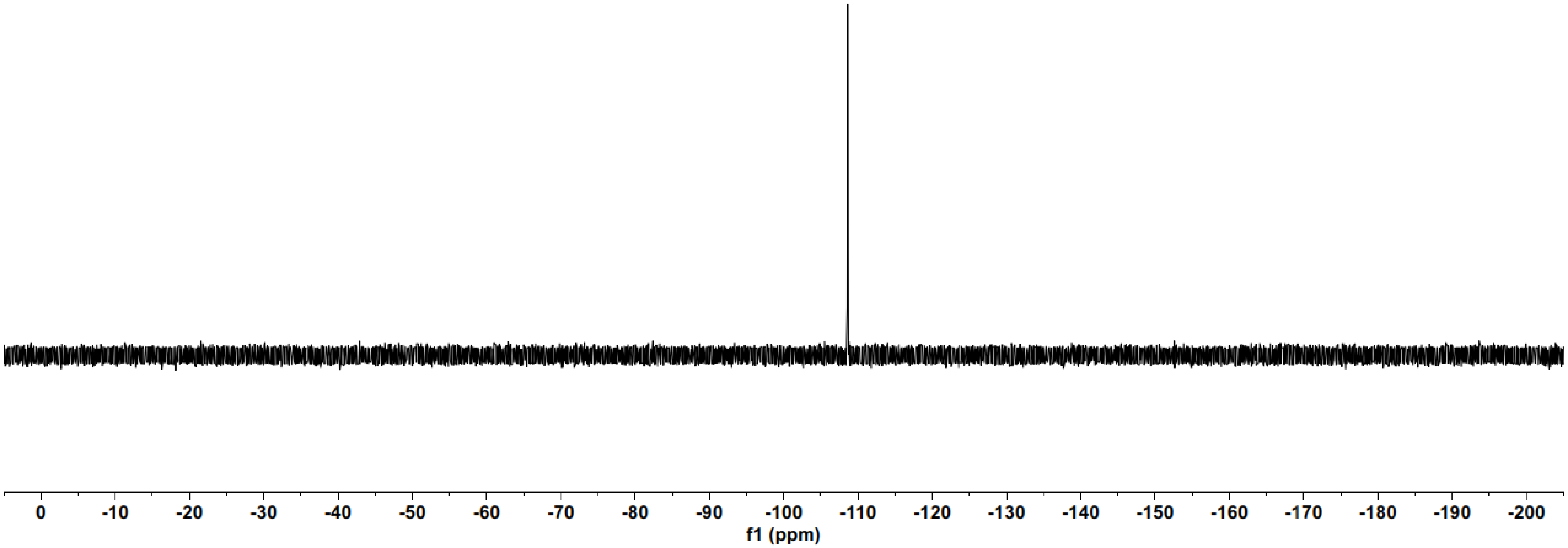

^{1}H NMR of 4b

CDCl$_3$, 500 MHz, 25 °C

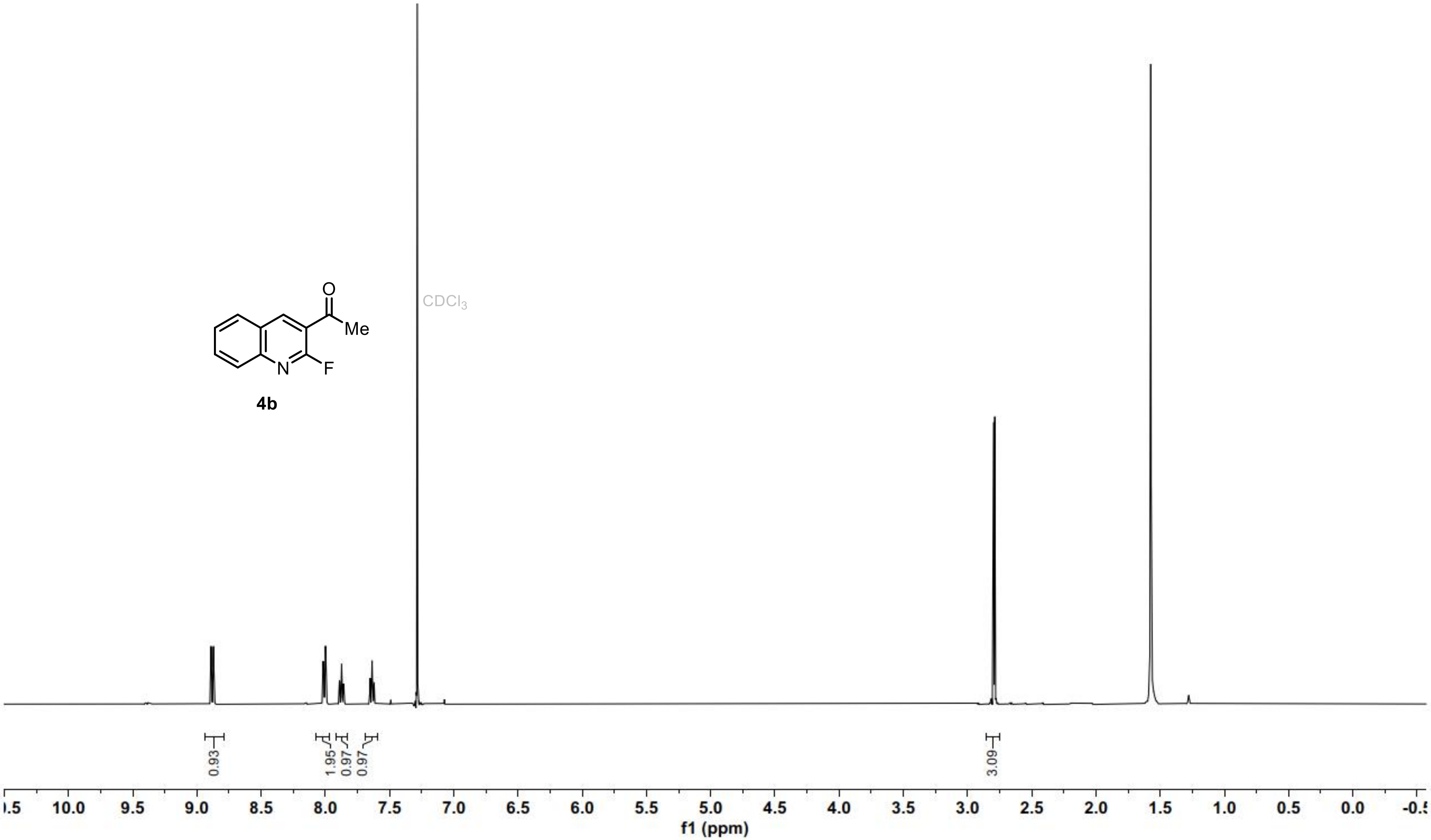

^{13}C NMR of 4b

$CDCl_3$, 151 MHz, 25 °C

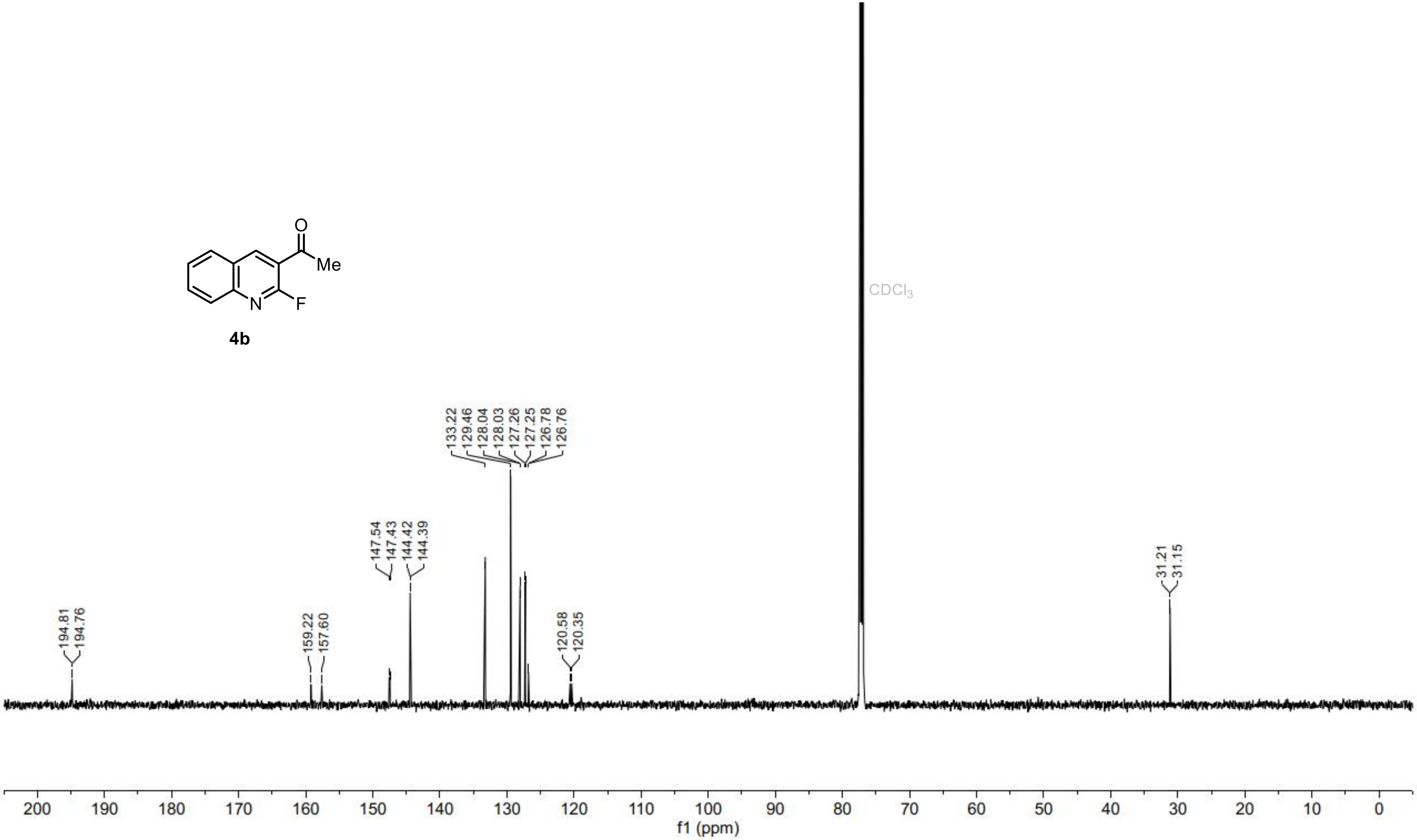

^{19}F NMR of 4b

CDCl$_3$, 471 MHz, 25 °C

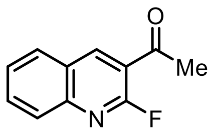

4b

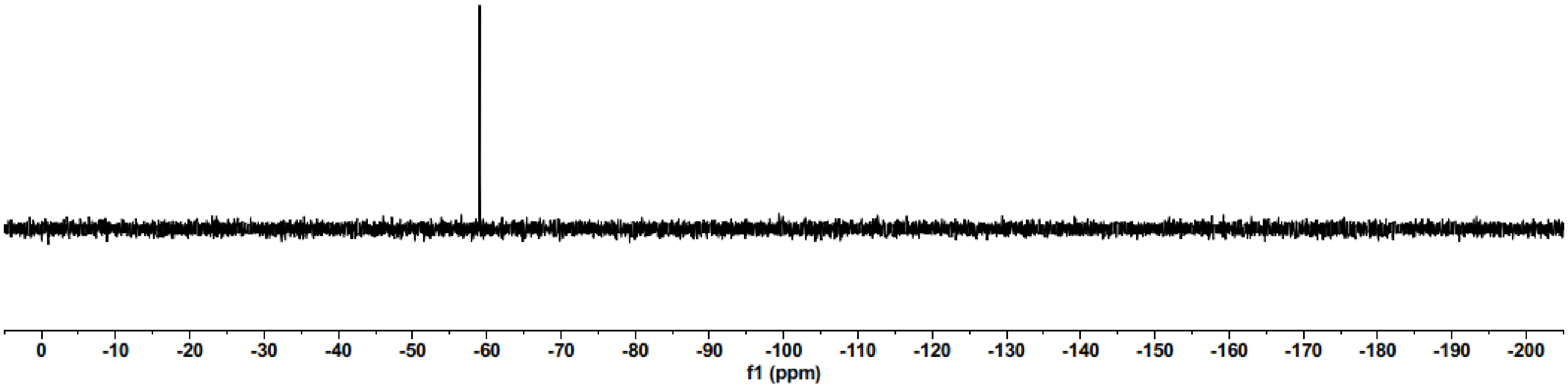

^{1}H NMR of 5·HCl

CD_3CN, 500 MHz, 25 °C

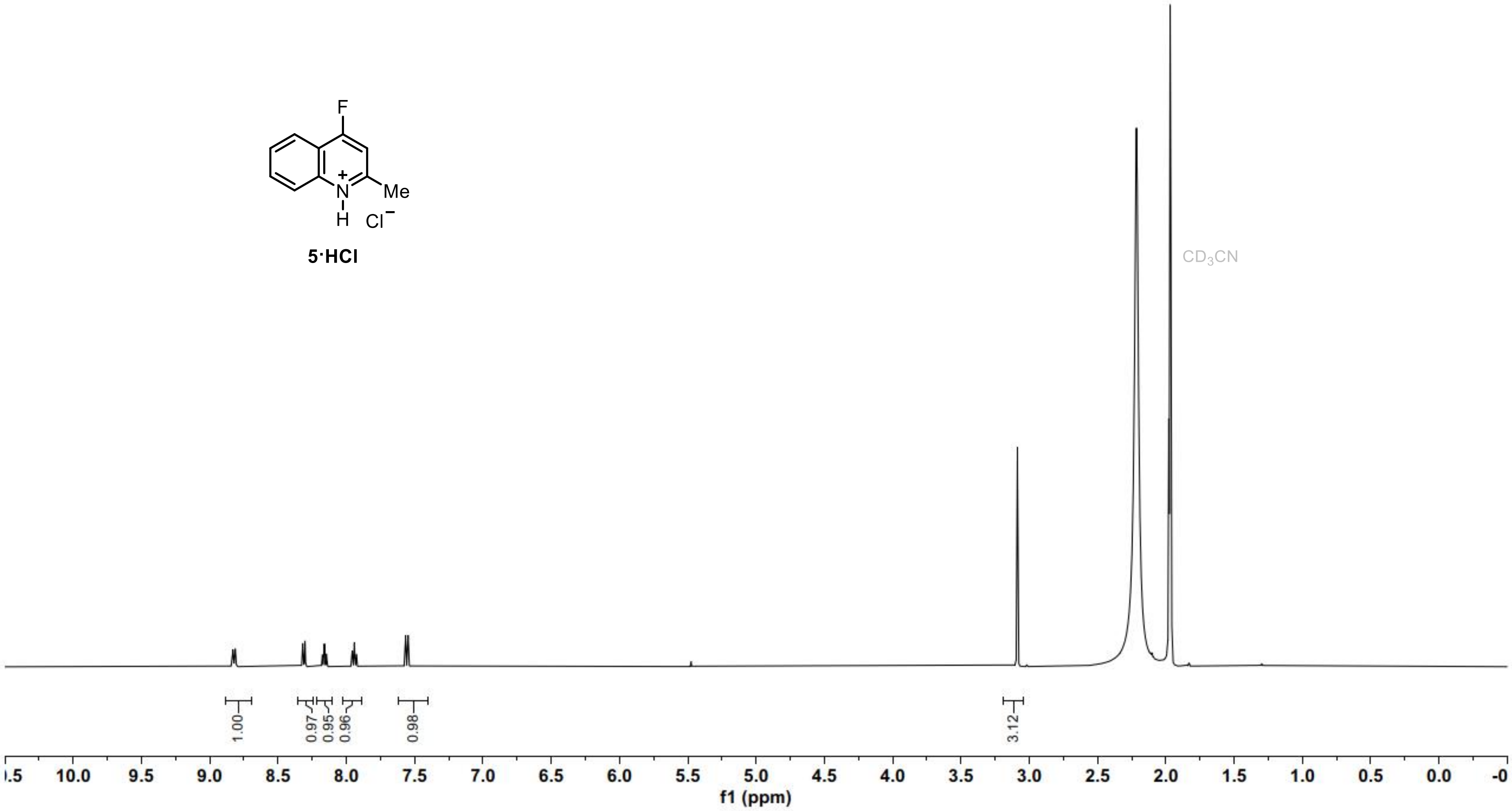

^{13}C NMR of 5·HCl

CD_3CN, 151 MHz, 25 °C

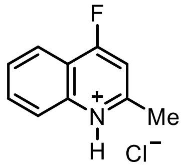

5·HCl

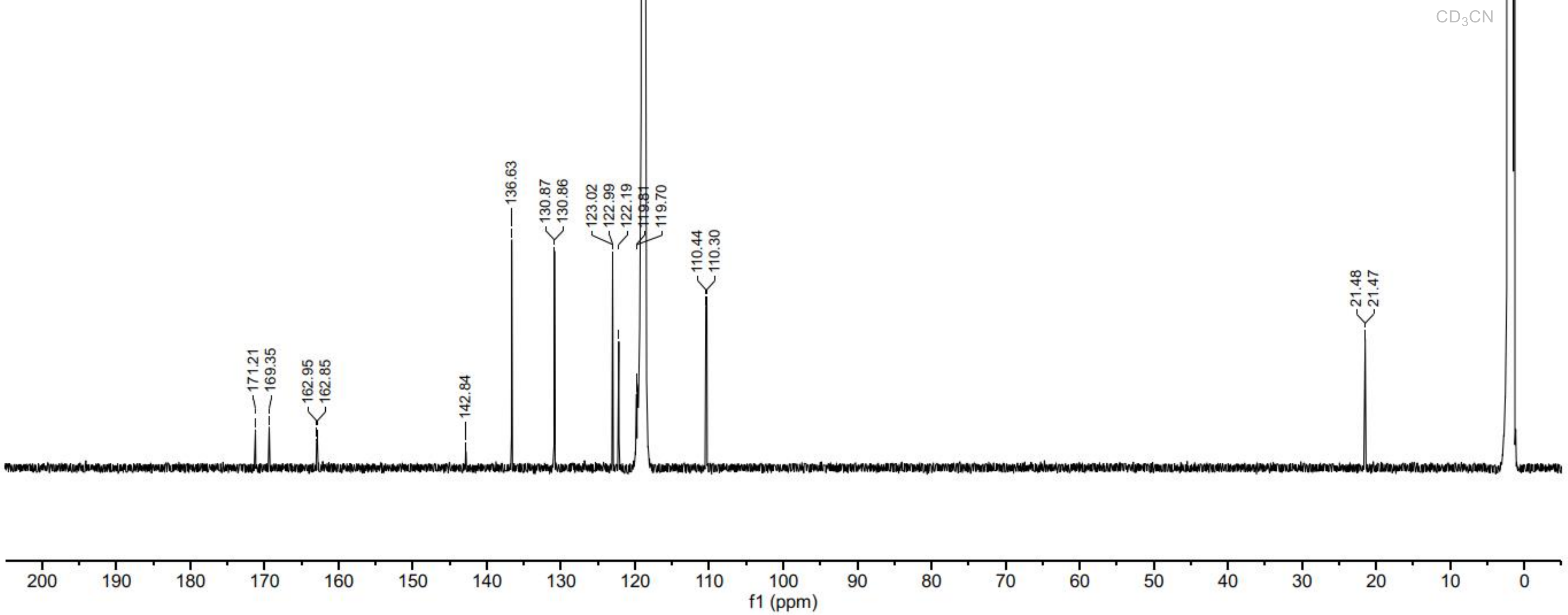

^{19}F NMR of 5·HCl

CD_3CN, 471 MHz, 25 °C

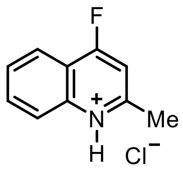

5·HCl

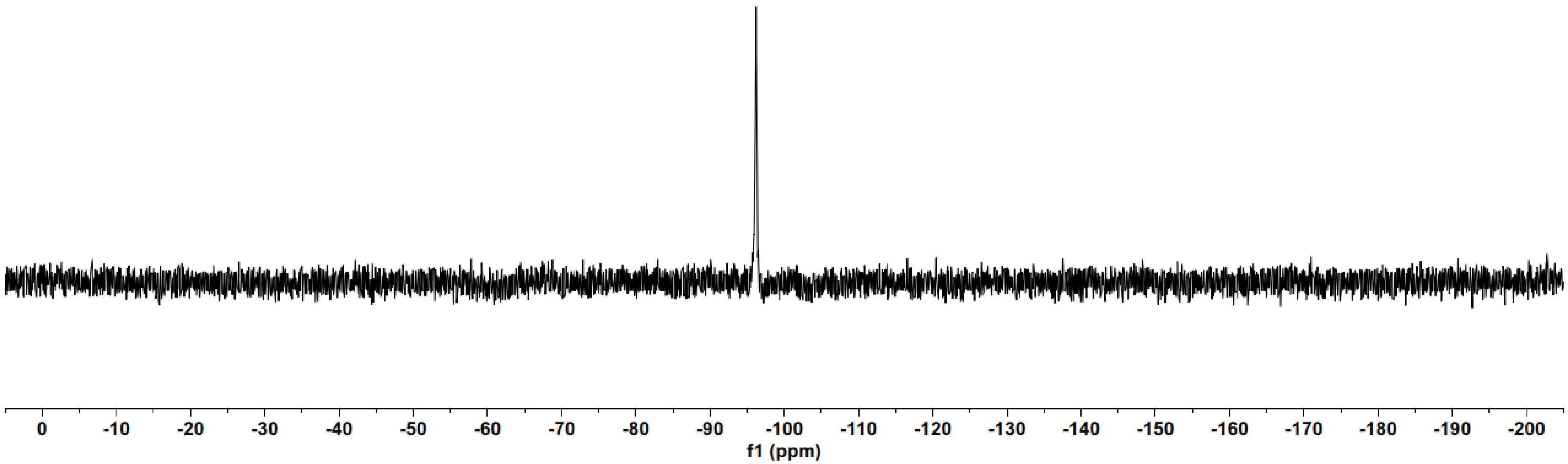

^{1}H NMR of 6

CDCl3, 500 MHz, 25 °C

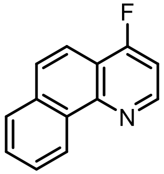

6

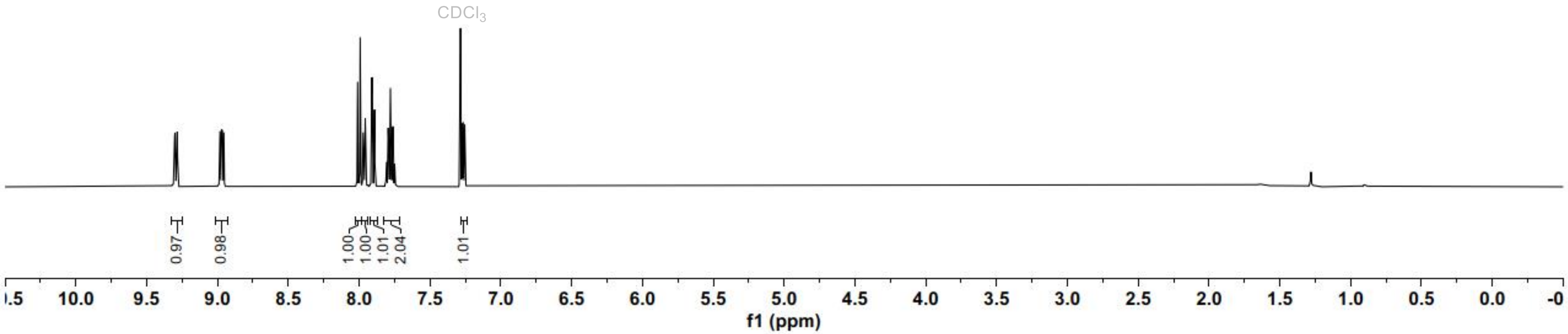

^{13}C NMR of 6

$CDCl_3$, 126 MHz, 25 °C

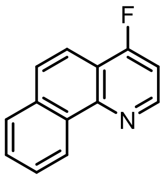

6

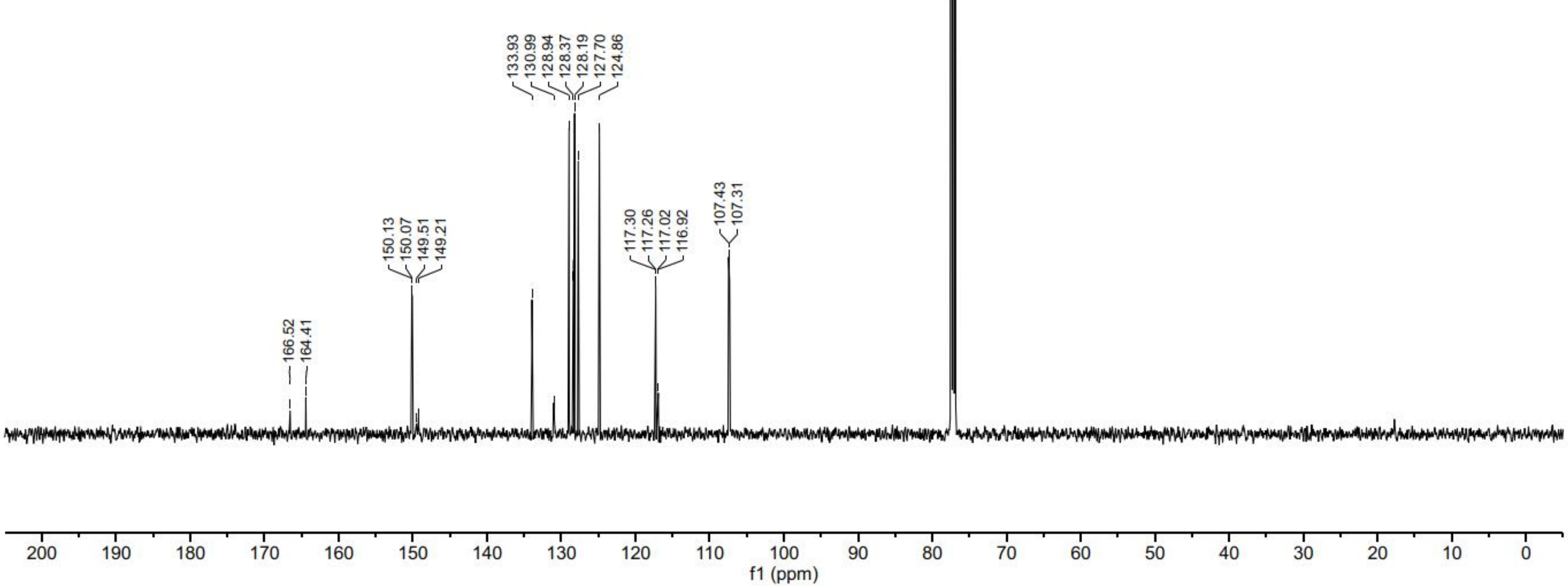

^{19}F NMR of 6

CDCl$_3$, 471 MHz, 25 °C

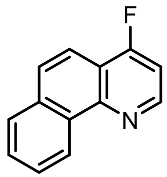

6

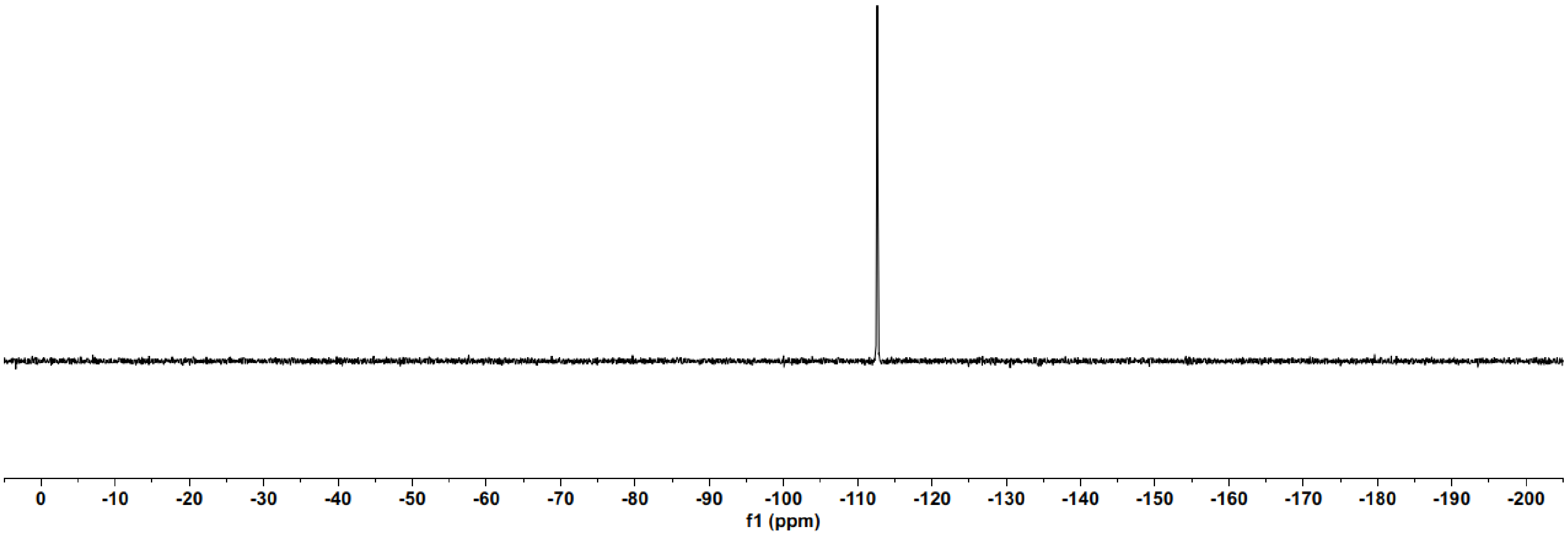

^{1}H NMR of 7

$CDCl_3$, 500 MHz, 25 °C

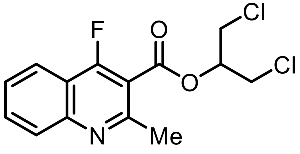

7

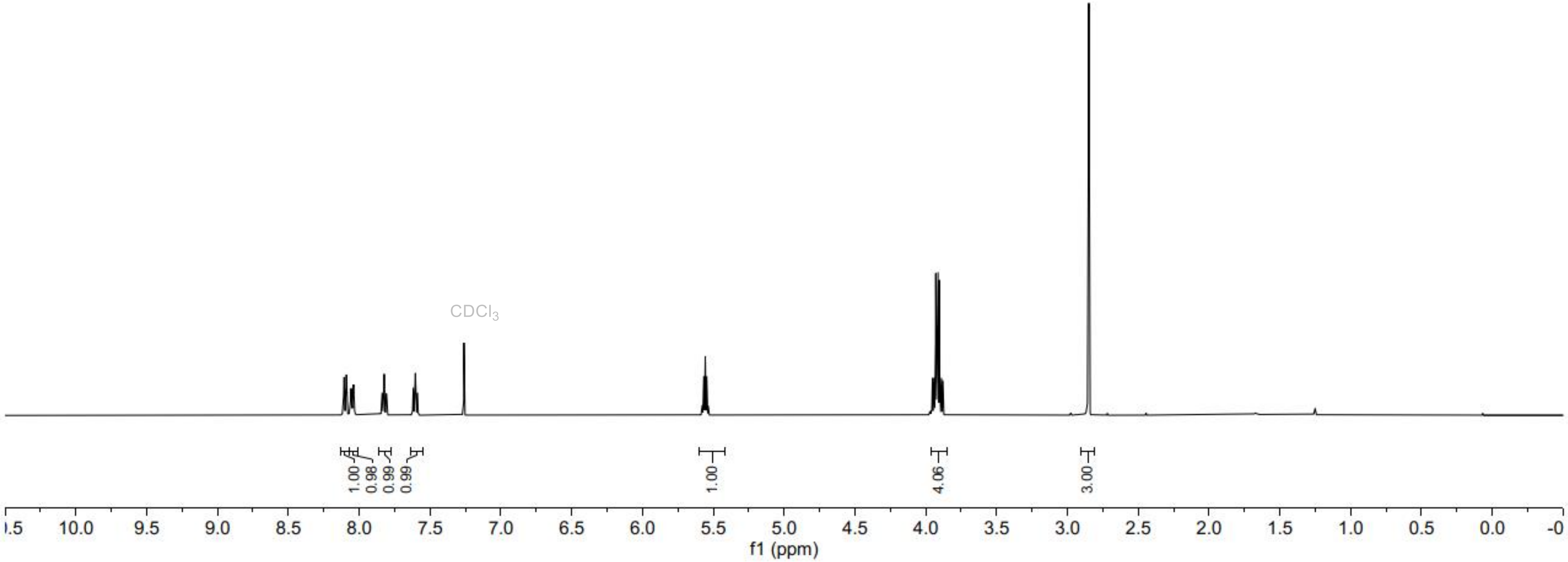

^{13}C NMR of 7

$CDCl_3$, 126 MHz, 25 °C

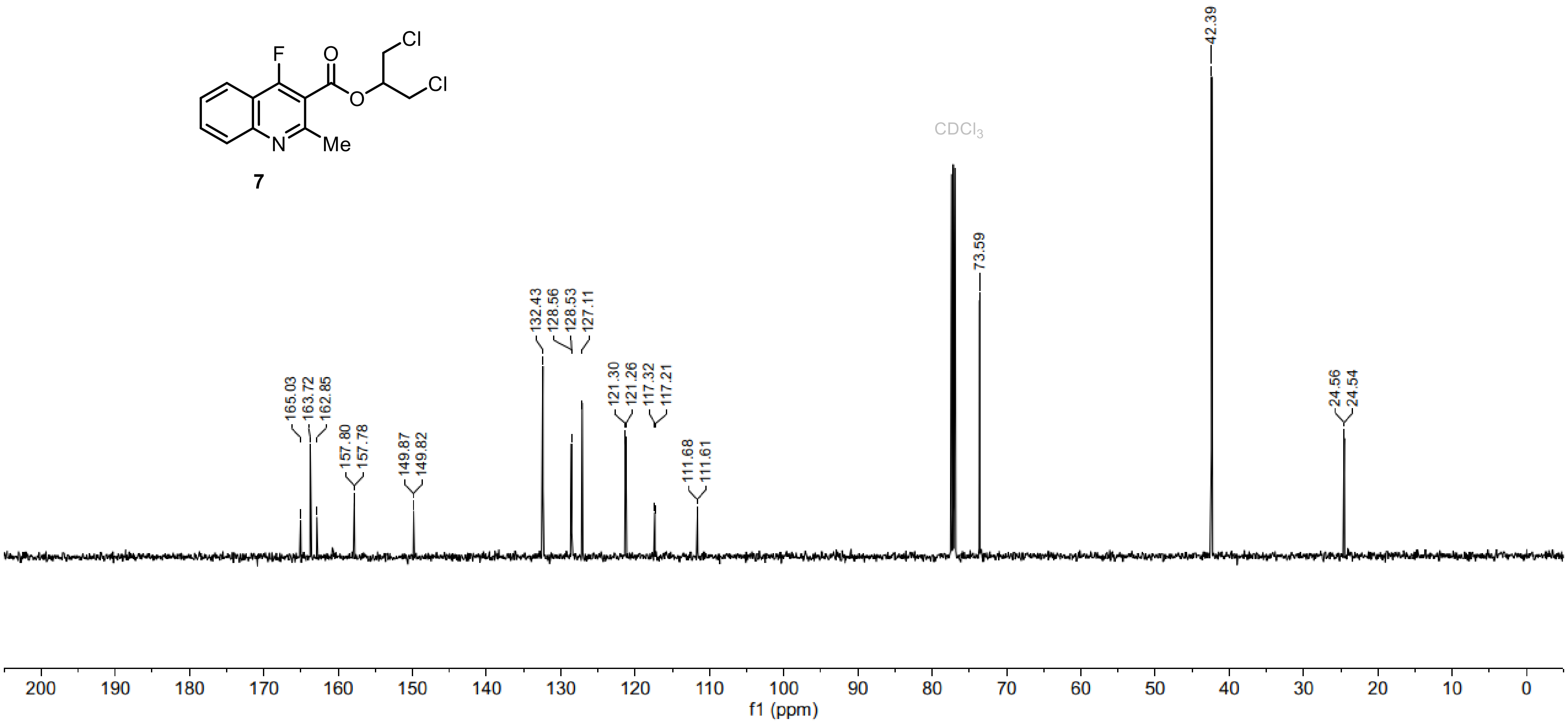

^{19}F NMR of 7

CDCl$_3$, 471 MHz, 25 °C

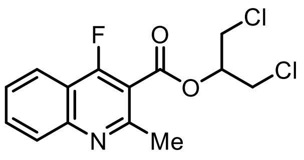

7

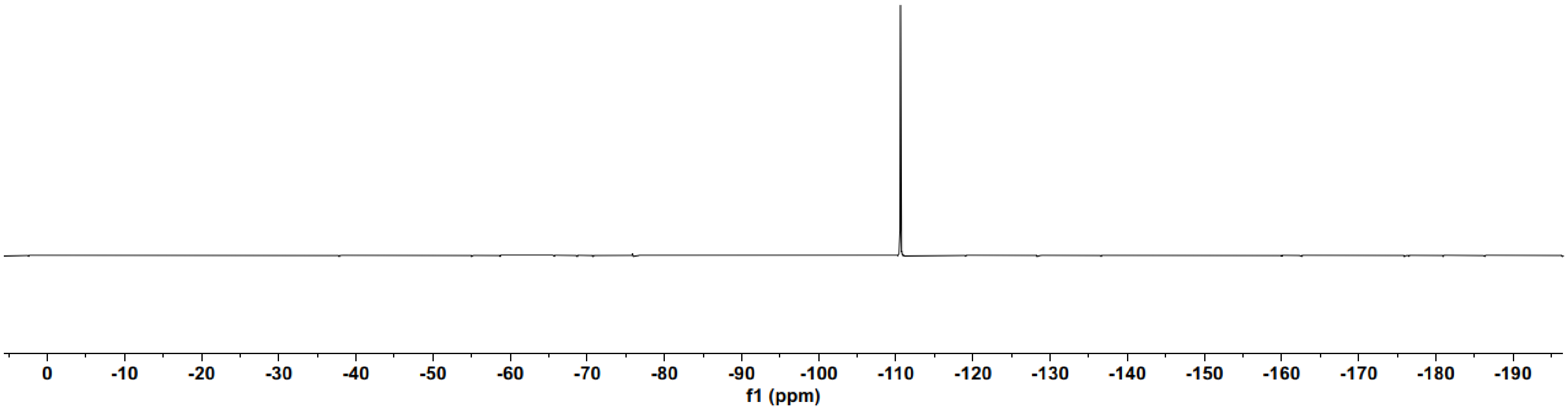

^{1}H NMR of 3a·HCl

CD_3CN, 500 MHz, 25 °C

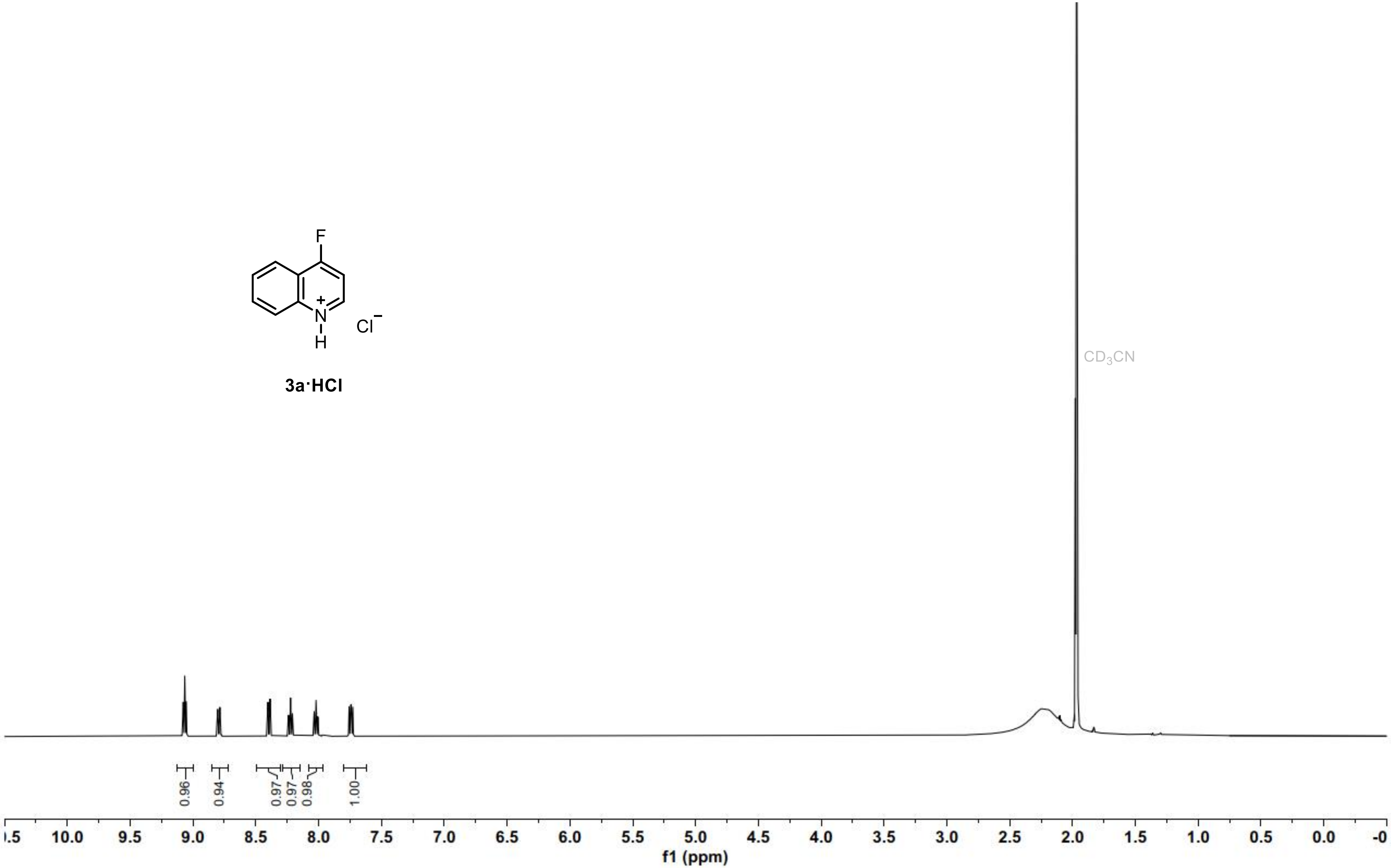

^{13}C NMR of 3a·HCl

CD_3CN, 151 MHz, 25 °C

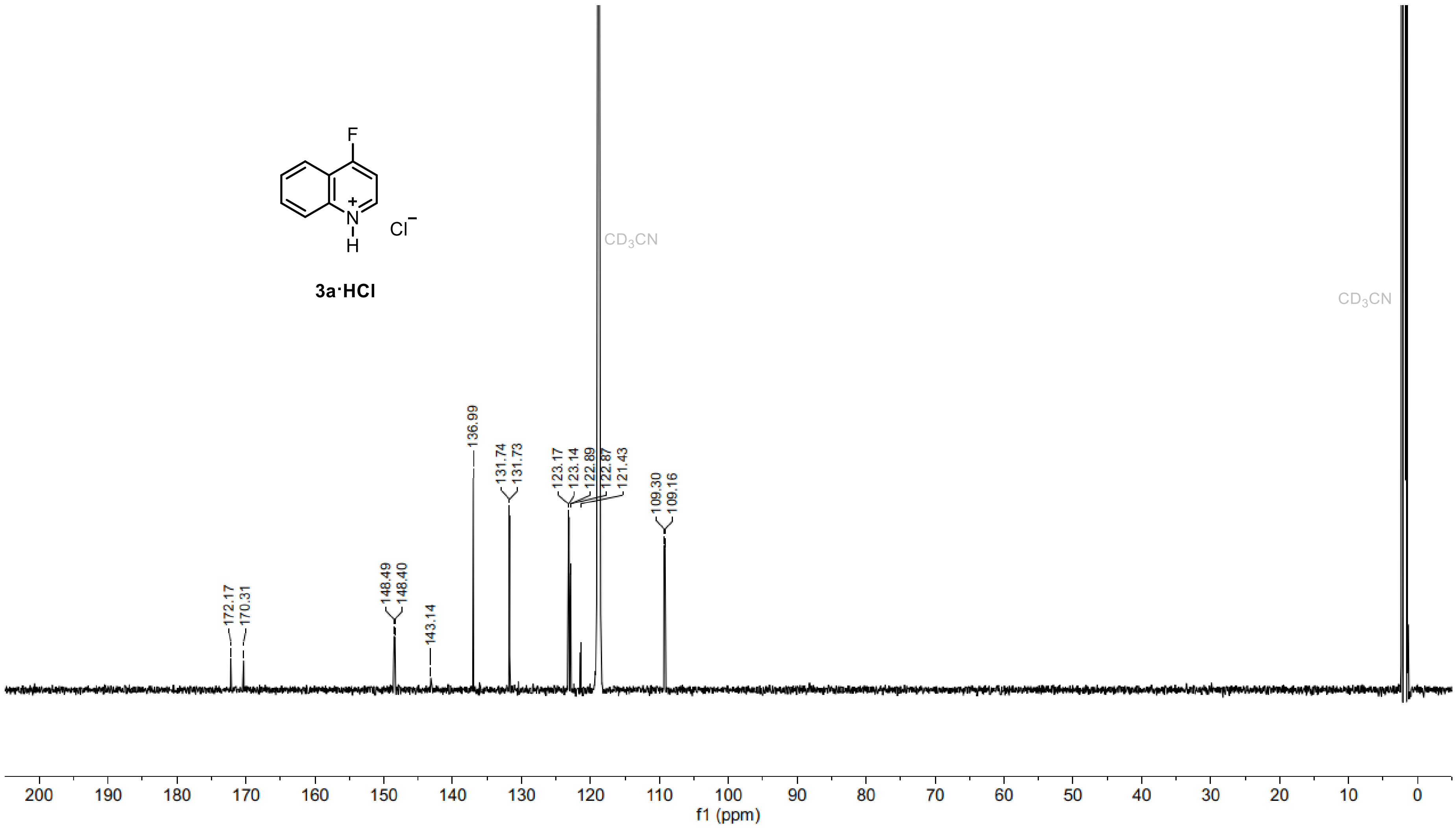

^{19}F NMR of 3a·HCl

CD_3CN, 471 MHz, 25 °C

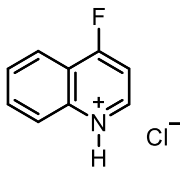

3a·HCl

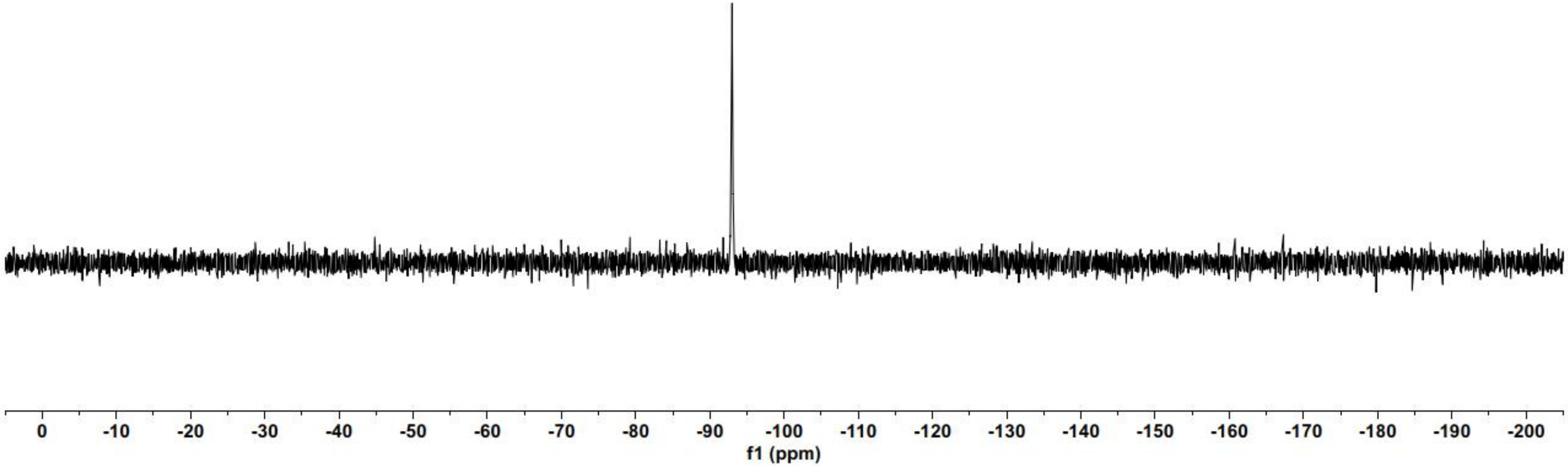

^{1}H NMR of 3b

CD$_3$CN, 500 MHz, 25 °C

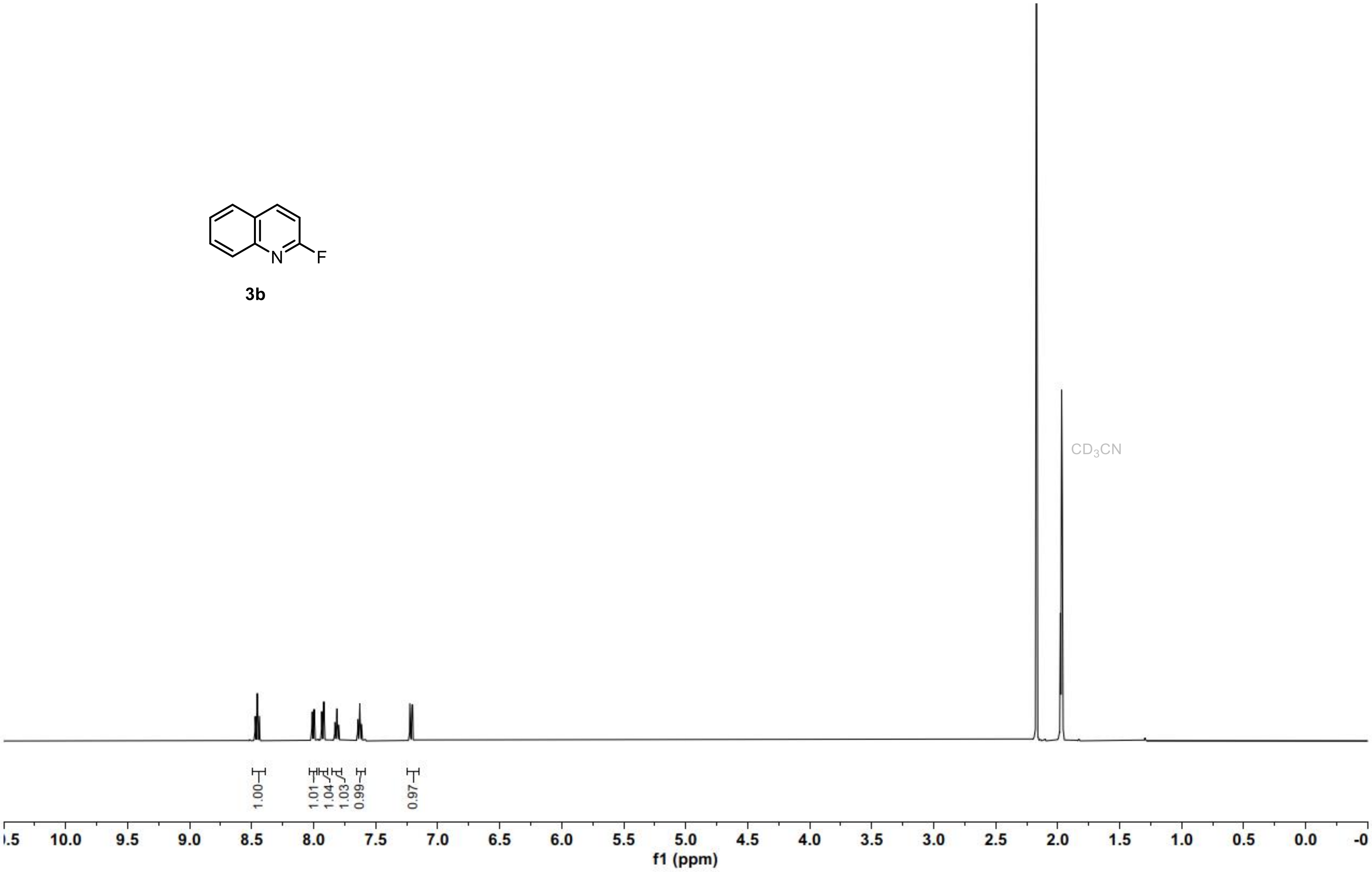

^{13}C NMR of 3b

CD_3CN, 151 MHz, 25 °C

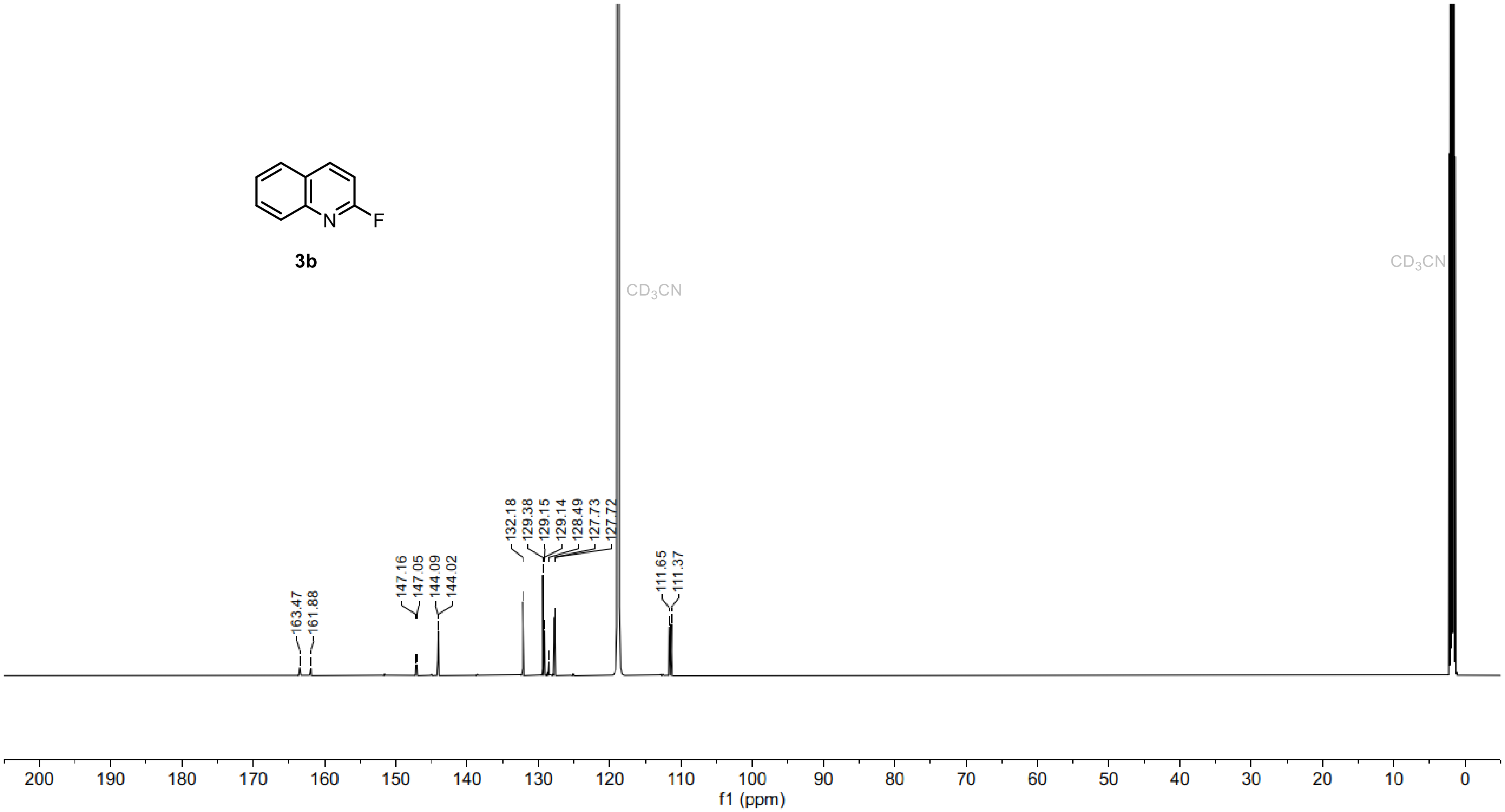

^{19}F NMR of 3b

CD_3CN, 471 MHz, 25 °C

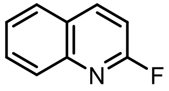

3b

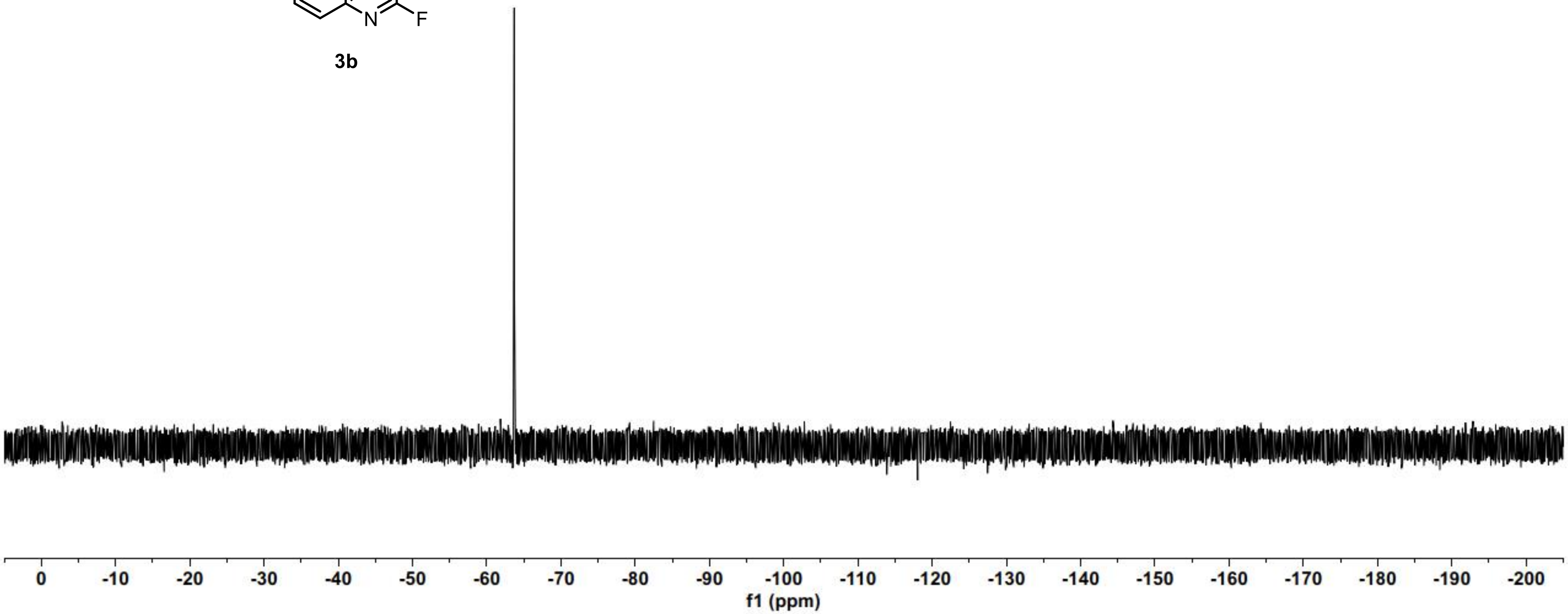

^{1}H NMR of 8a

CDCl₃, 500 MHz, 25 °C

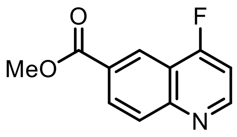

8a

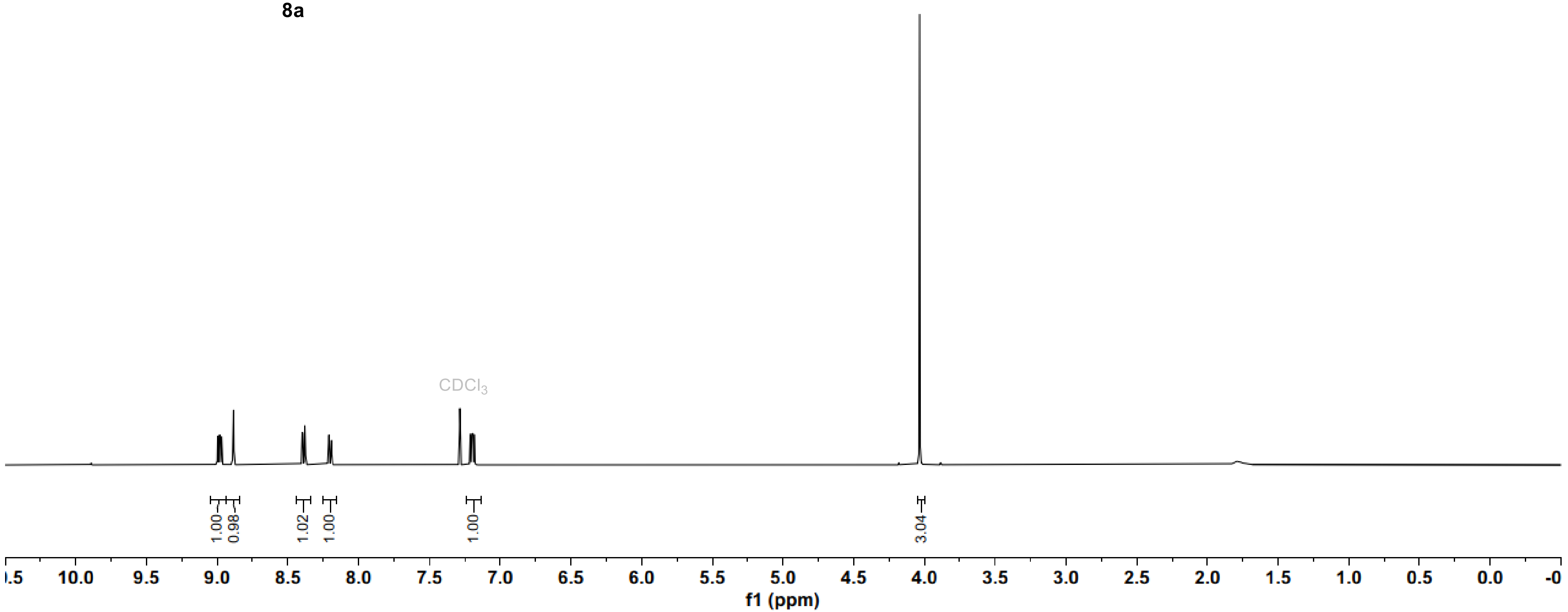

^{13}C NMR of 8a

$CDCl_3$, 126 MHz, 25 °C

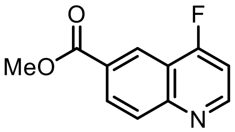

8a

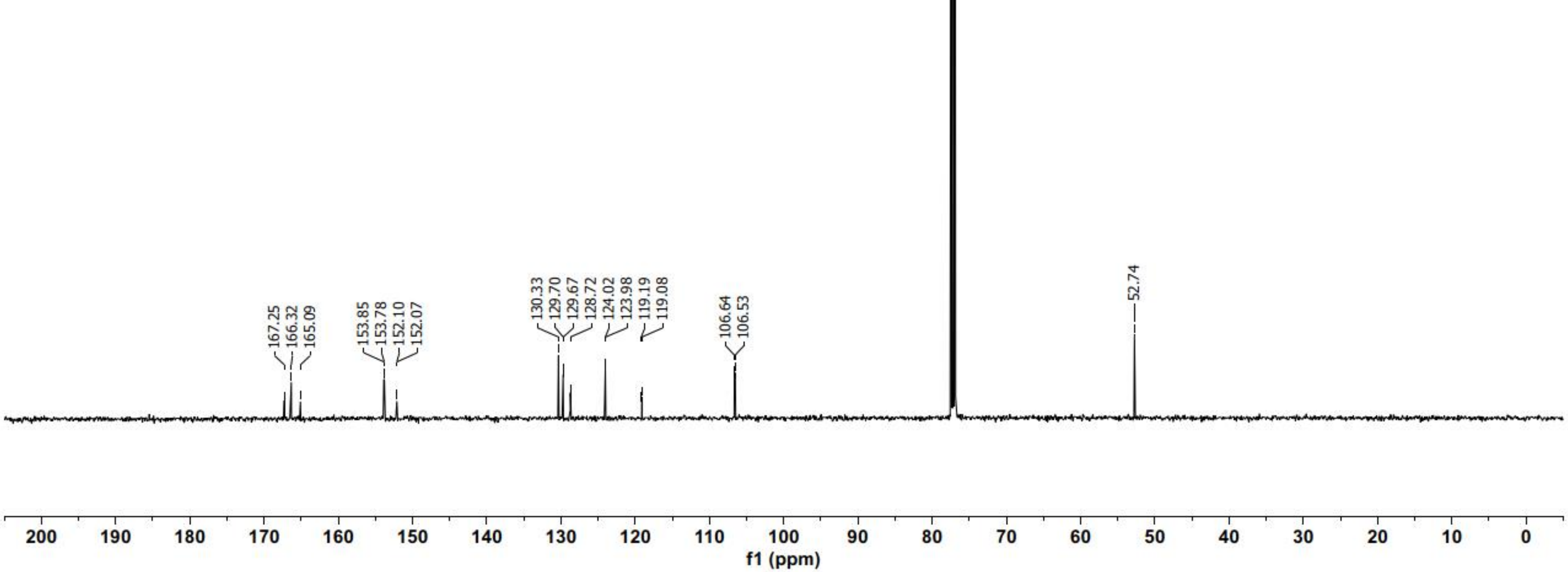

^{19}F NMR of 8a

CDCl$_3$, 471 MHz, 25 °C

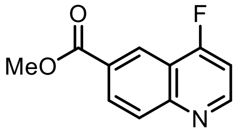

8a

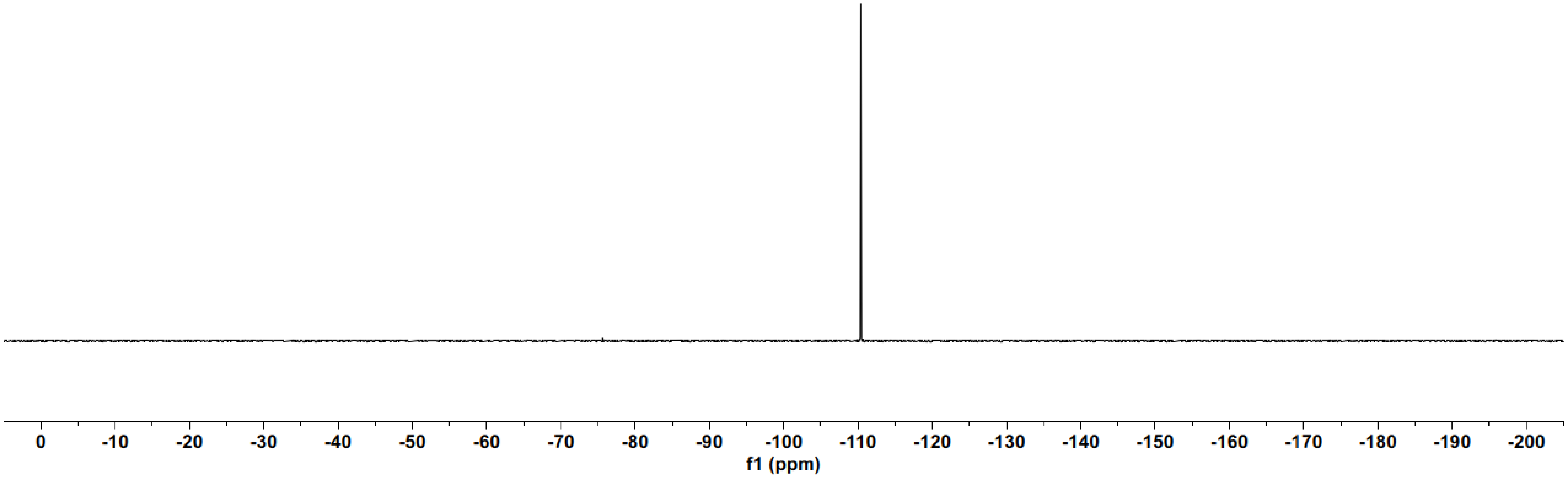

^{1}H NMR of 8b

CDCl$_3$, 500 MHz, 25 °C

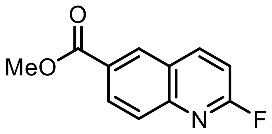

8b

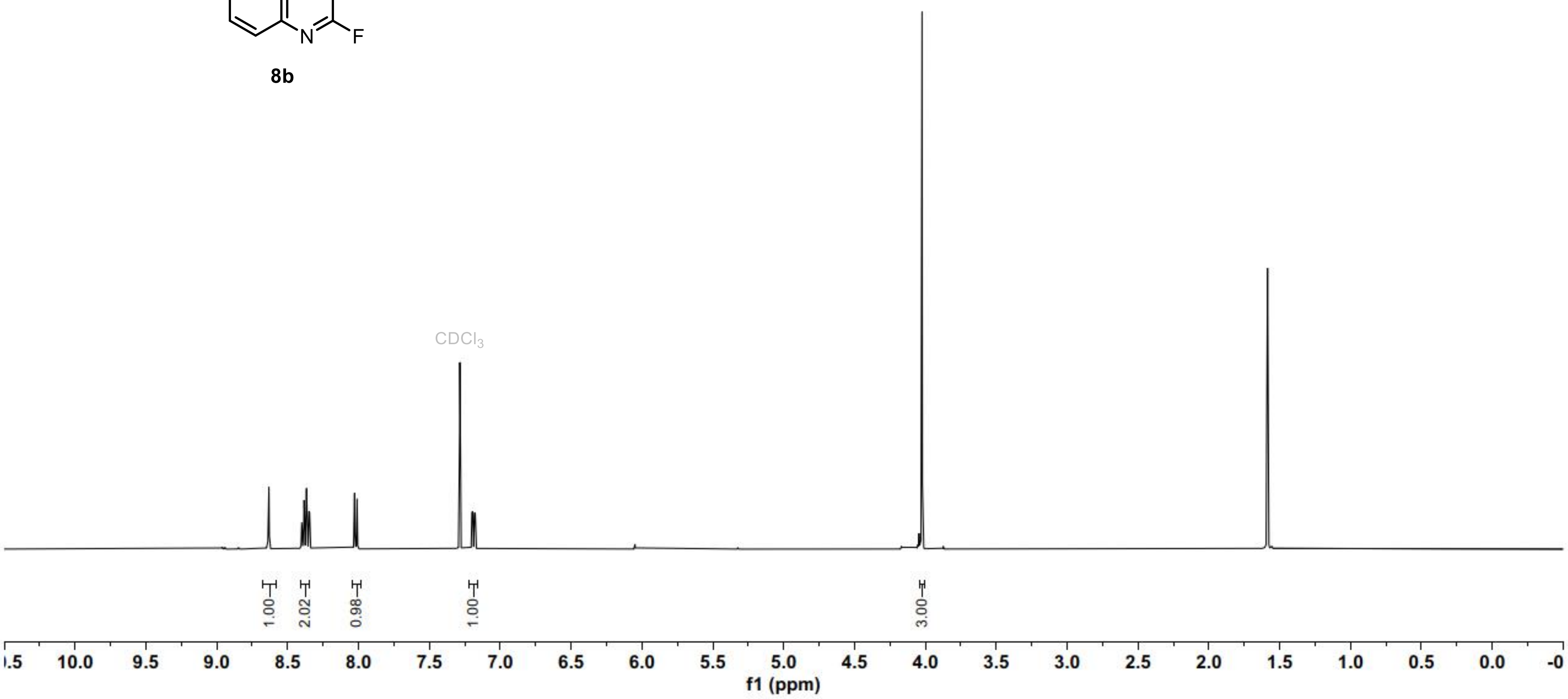

^{13}C NMR of 8b

$CDCl_3$, 151 MHz, 25 °C

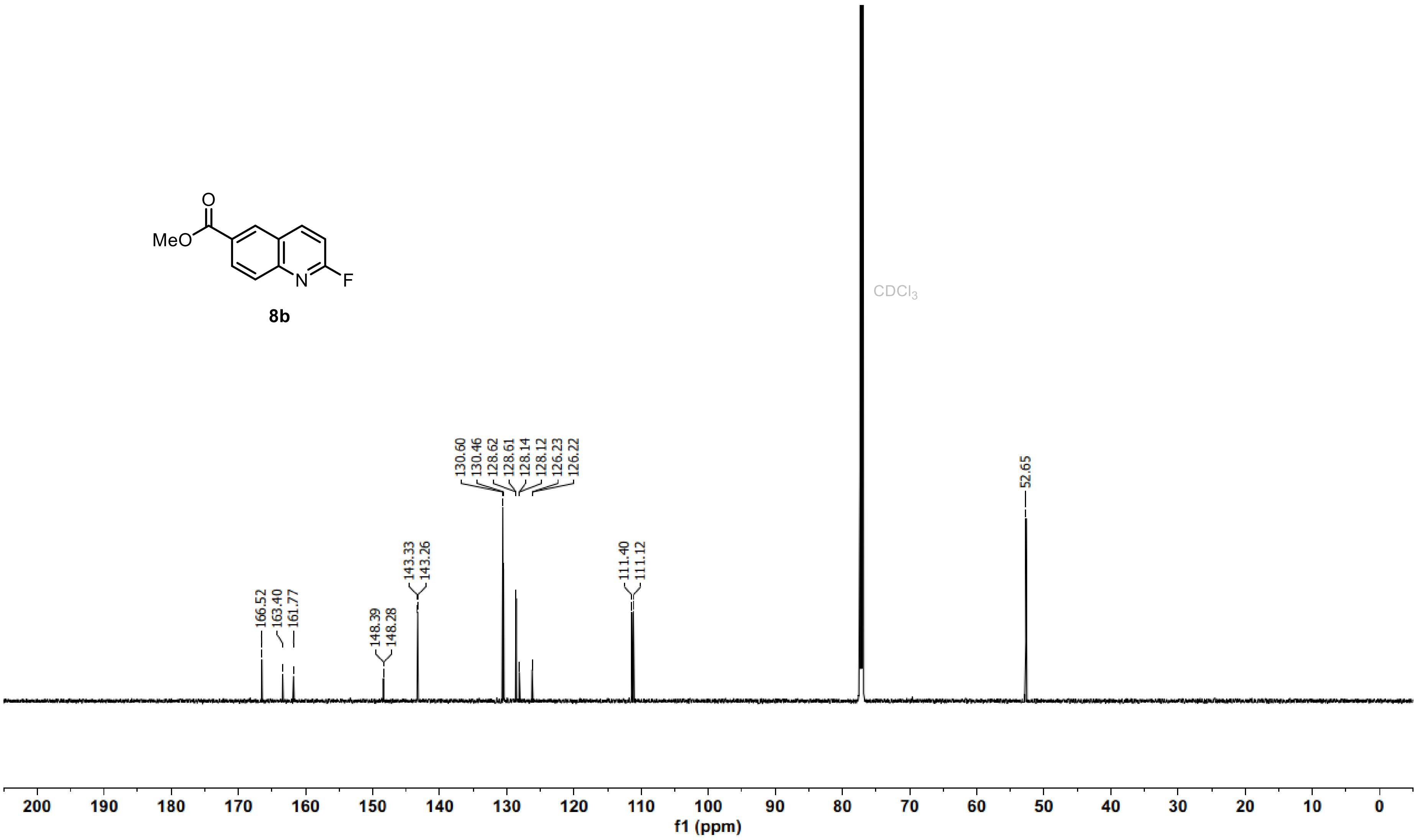

8b

^{19}F NMR of 8b

CDCl$_3$, 471 MHz, 25 °C

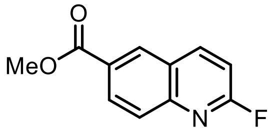

8b

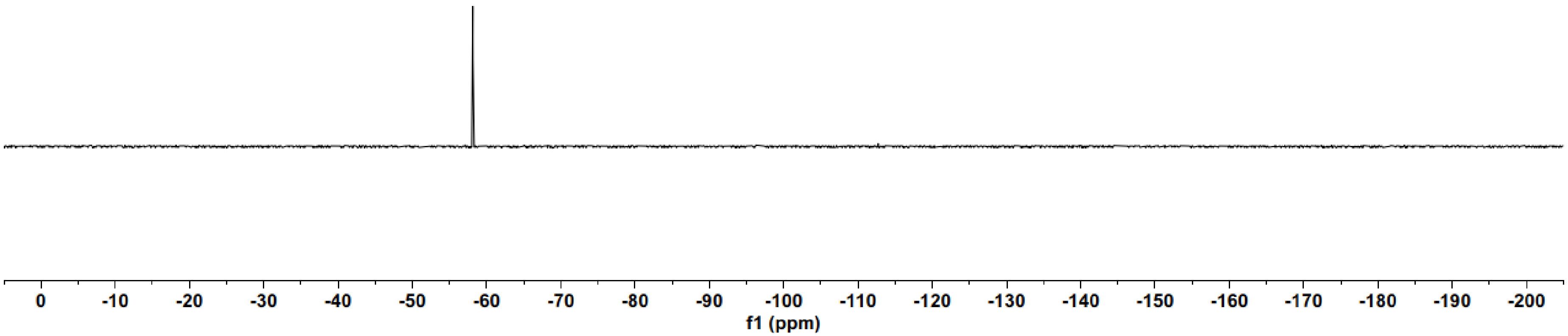

^{1}H NMR of 9·HCl

CD_3CN, 500 MHz, 25 °C

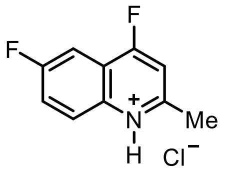

9·HCl

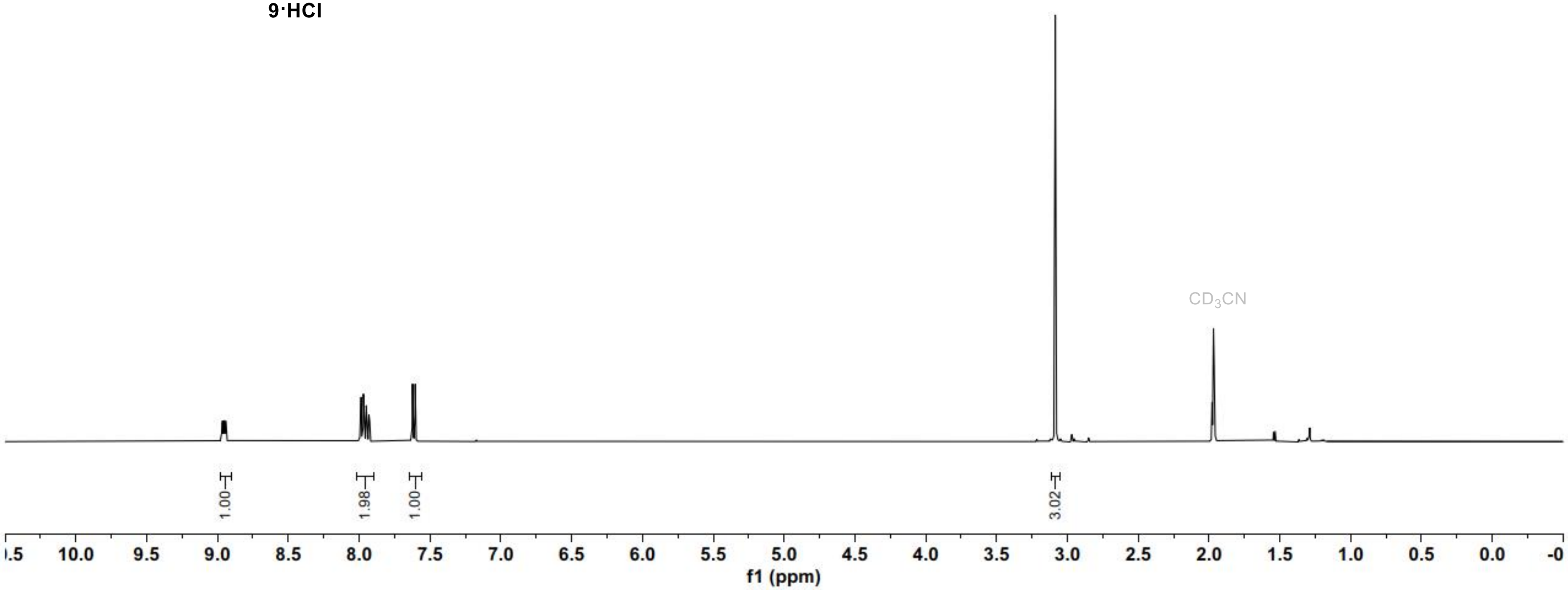

^{13}C NMR of 9·HCl

CD_3CN, 126 MHz, 25 °C

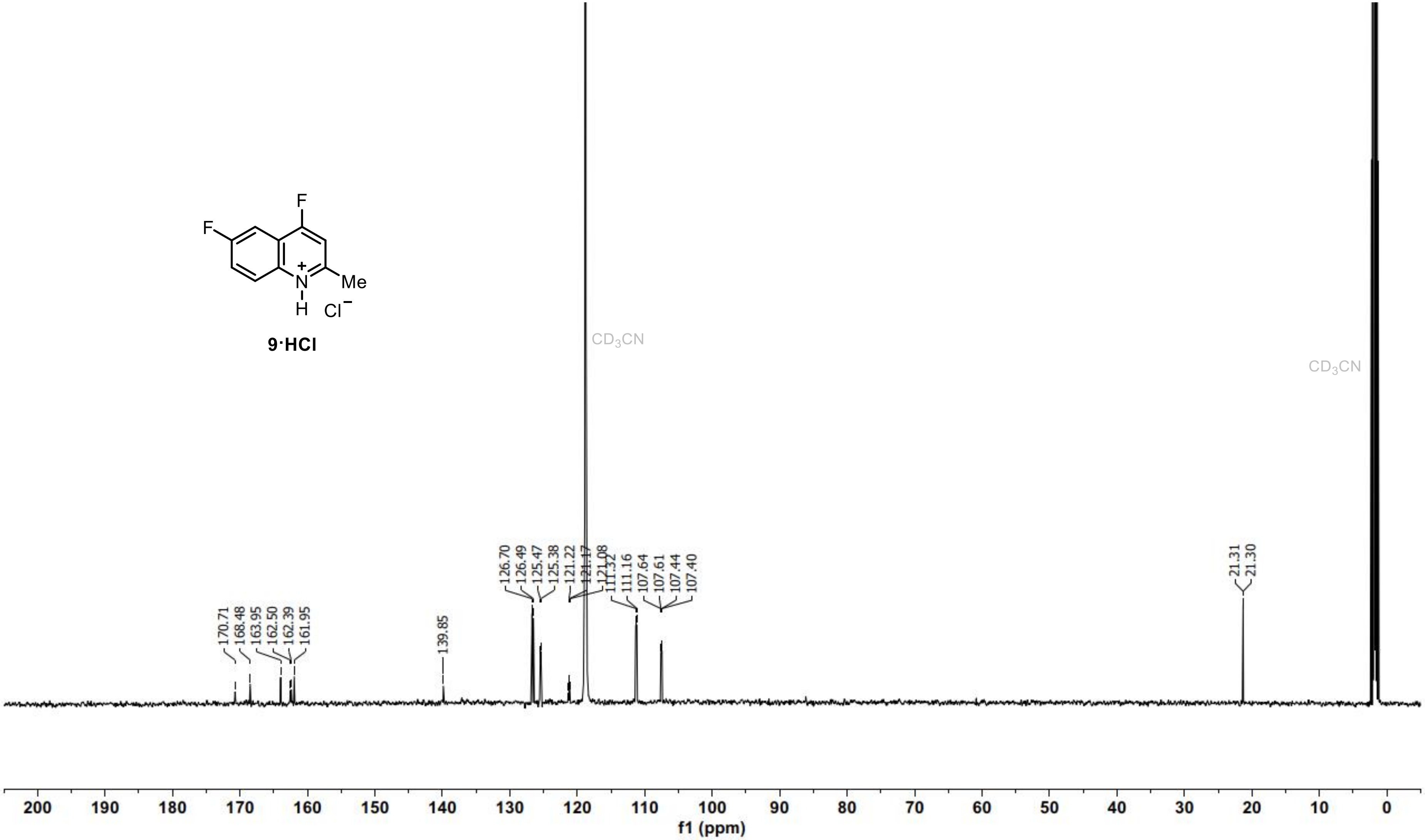

^{19}F NMR of 9·HCl

CD_3CN, 471 MHz, 25 °C

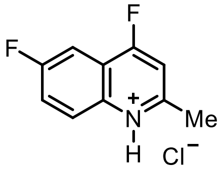

9·HCl

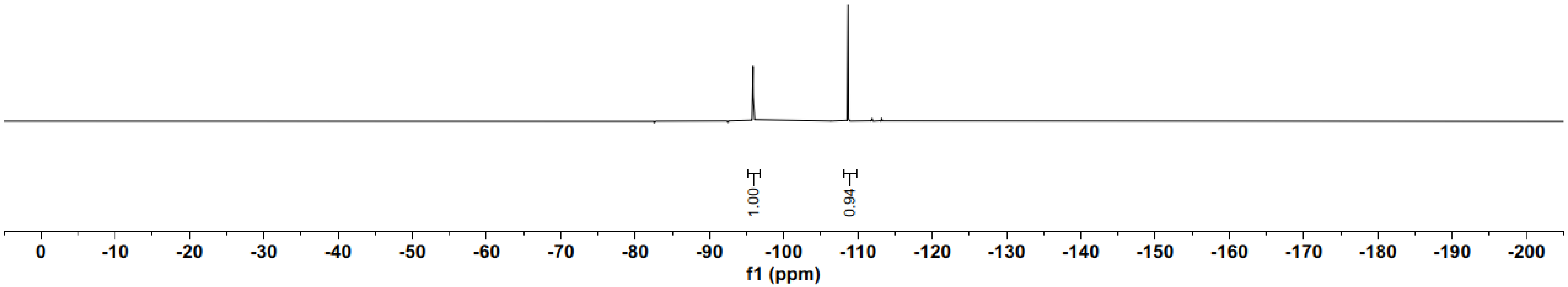

^{1}H NMR of 10

CDCl$_3$, 600 MHz, 25 °C

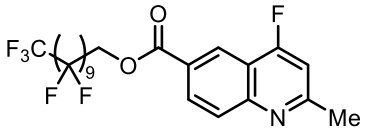

10

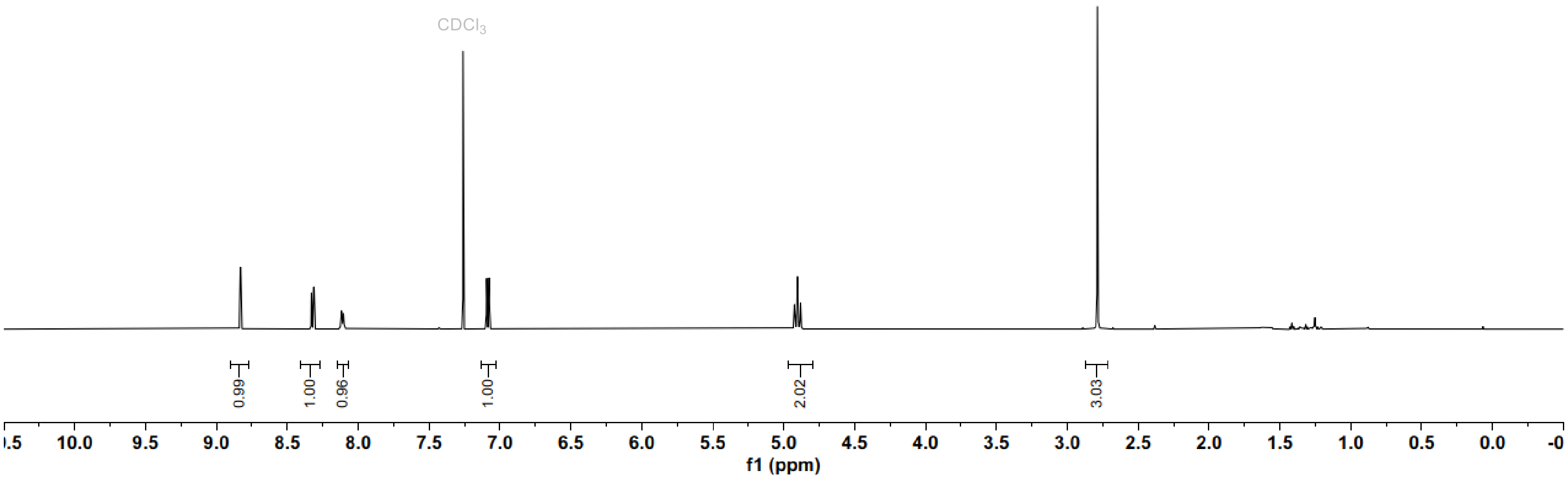

^{13}C NMR of 10

$CDCl_3$, 151 MHz, 25 °C

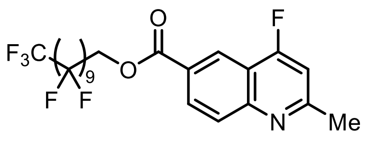

10

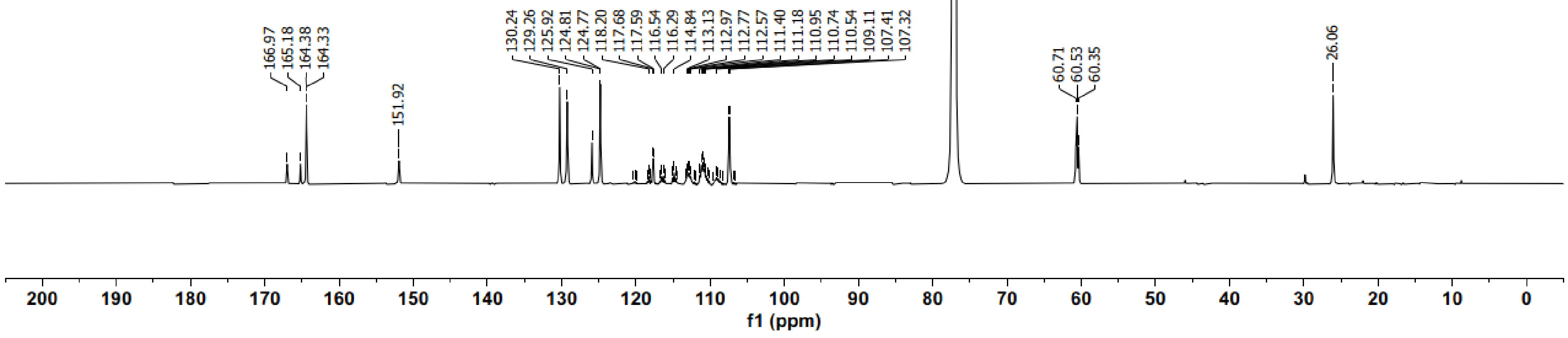

^{19}F NMR of 10

CDCl$_3$, 471 MHz, 25 °C

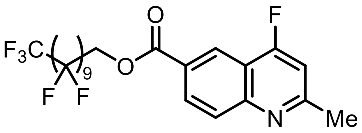

10

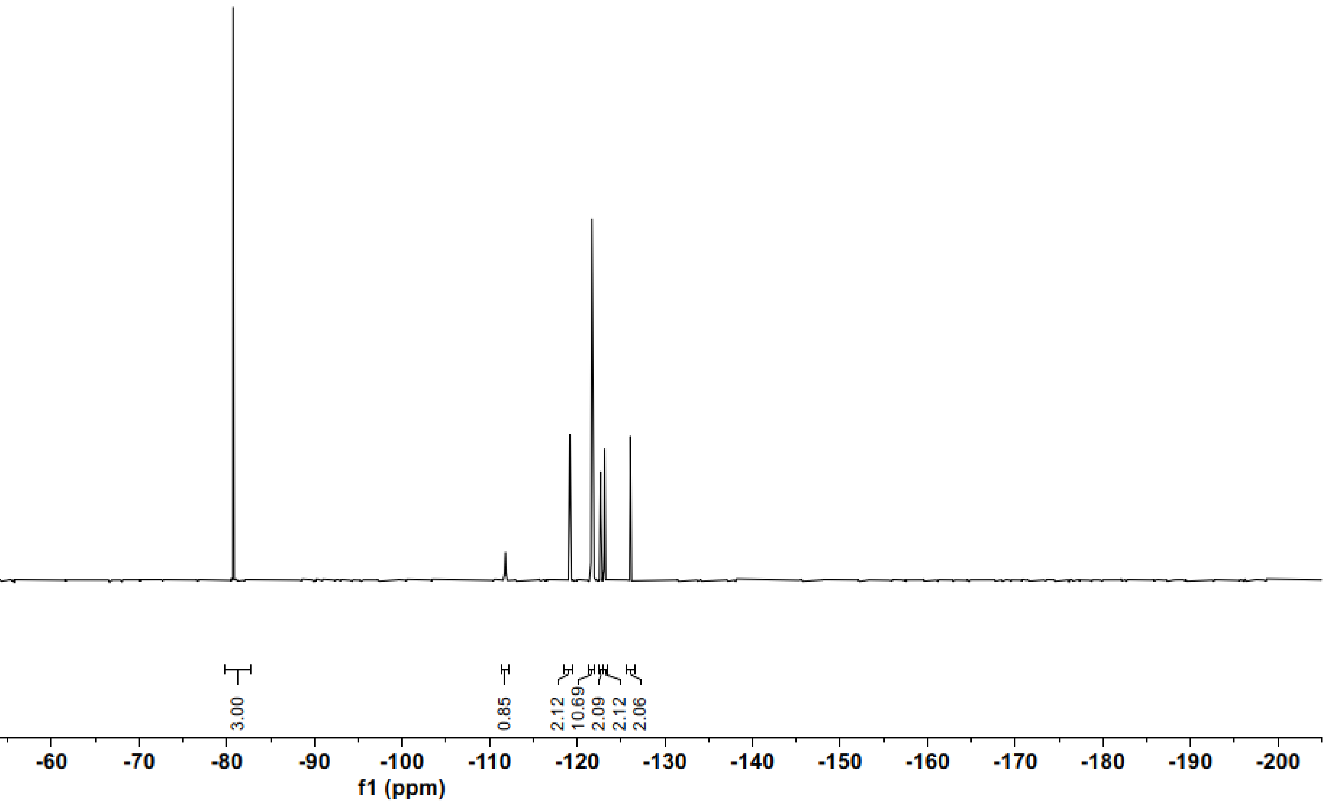

^{1}H NMR of 11

CDCl$_3$, 500 MHz, 25 °C

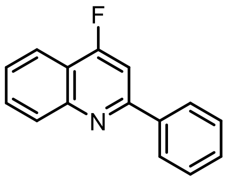

11

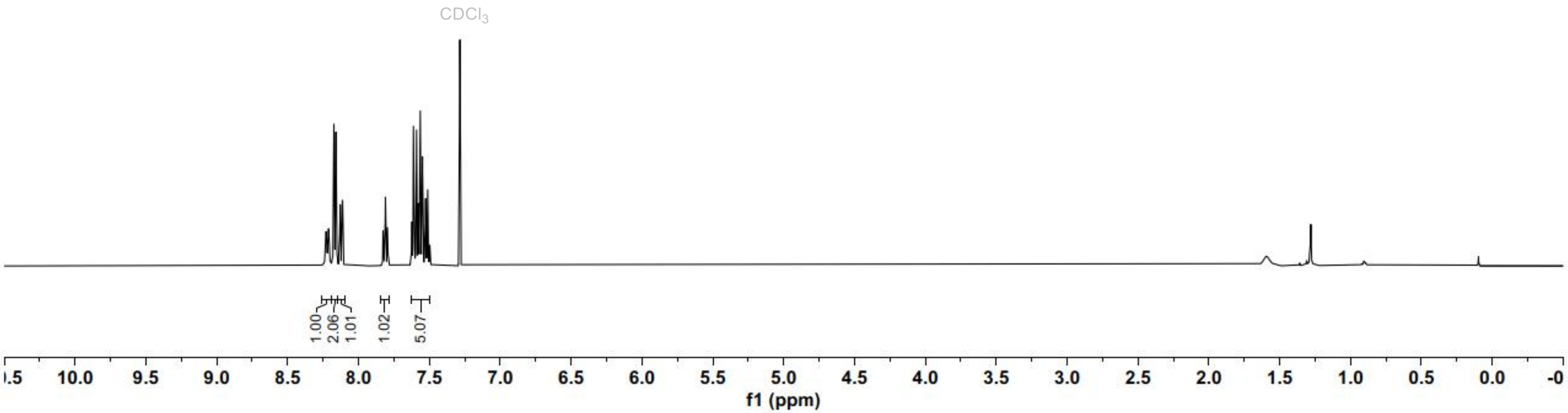

^{13}C NMR of 11

$CDCl_3$, 126 MHz, 25 °C

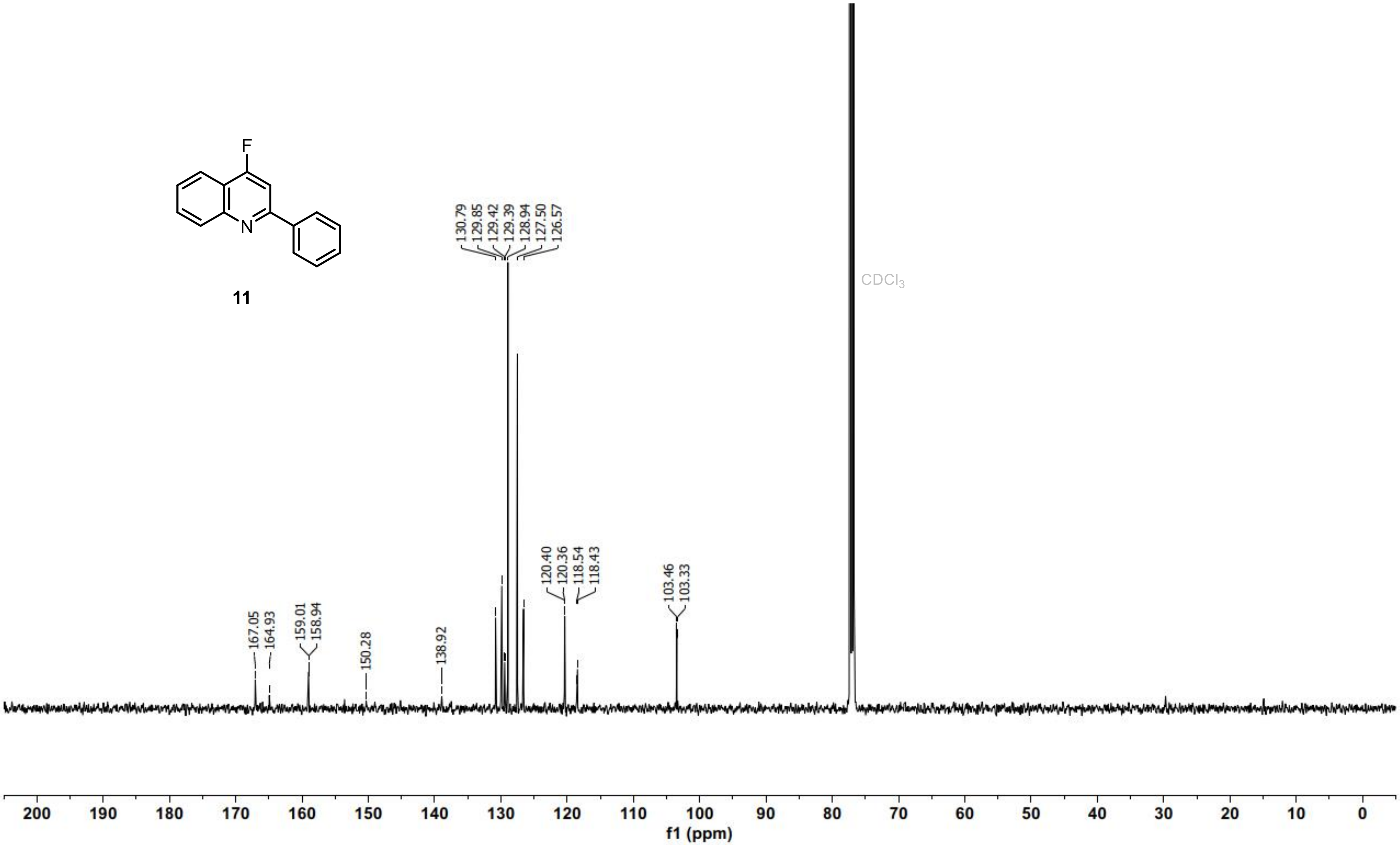

11

^{19}F NMR of 11

$CDCl_3$, 471 MHz, 25 °C

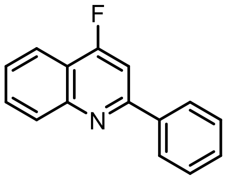

11

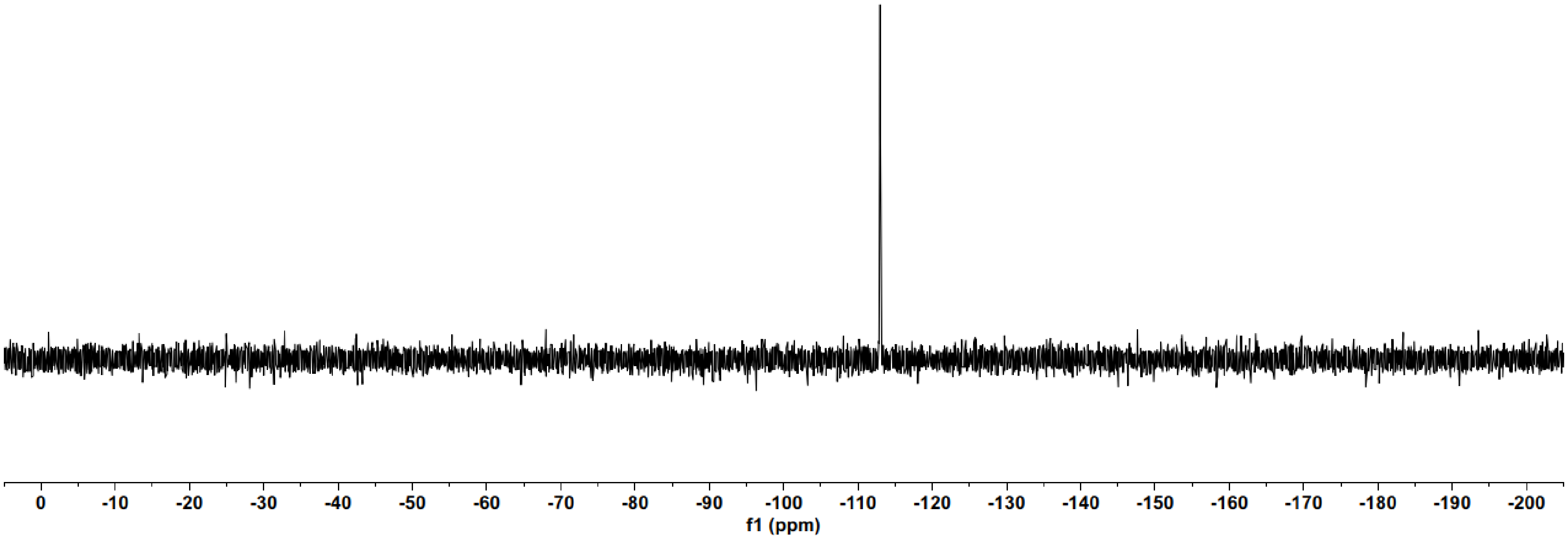

^{1}H NMR of 12a

CDCl$_3$, 500 MHz, 25 °C

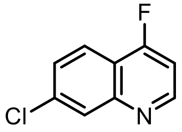

12a

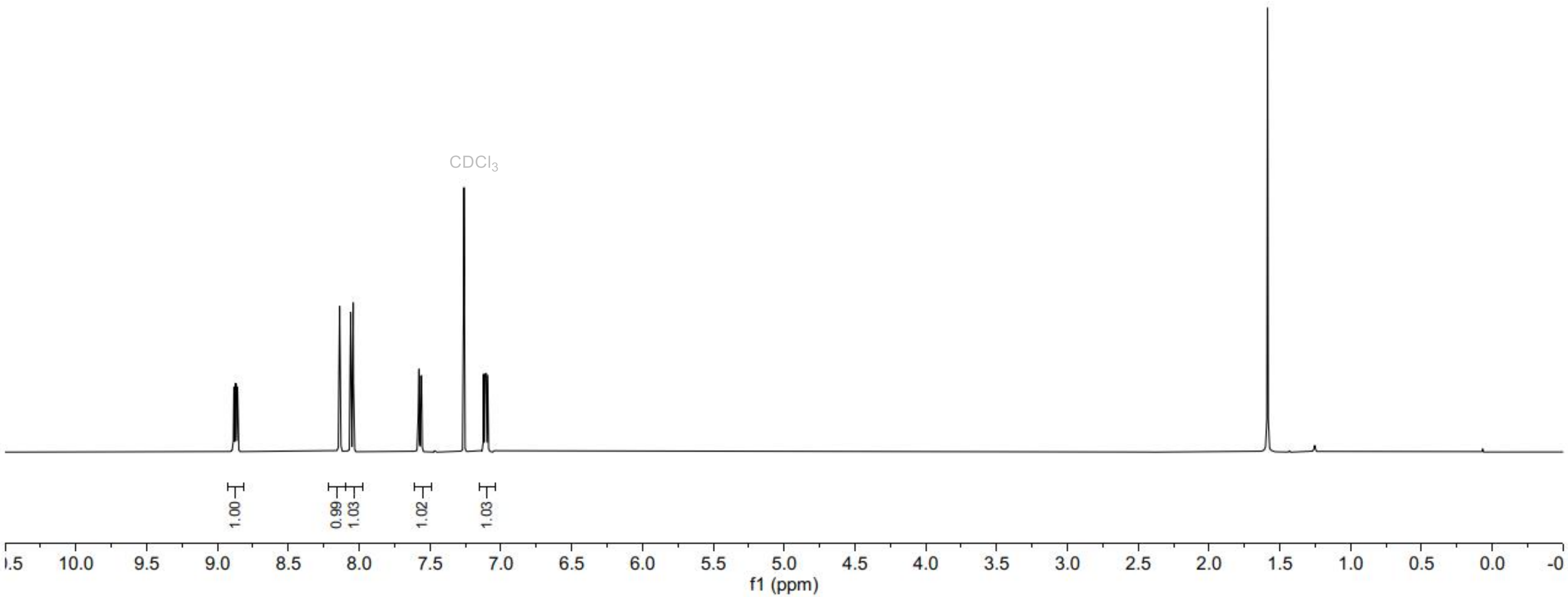

^{13}C NMR of 12a

$CDCl_3$, 151 MHz, 25 °C

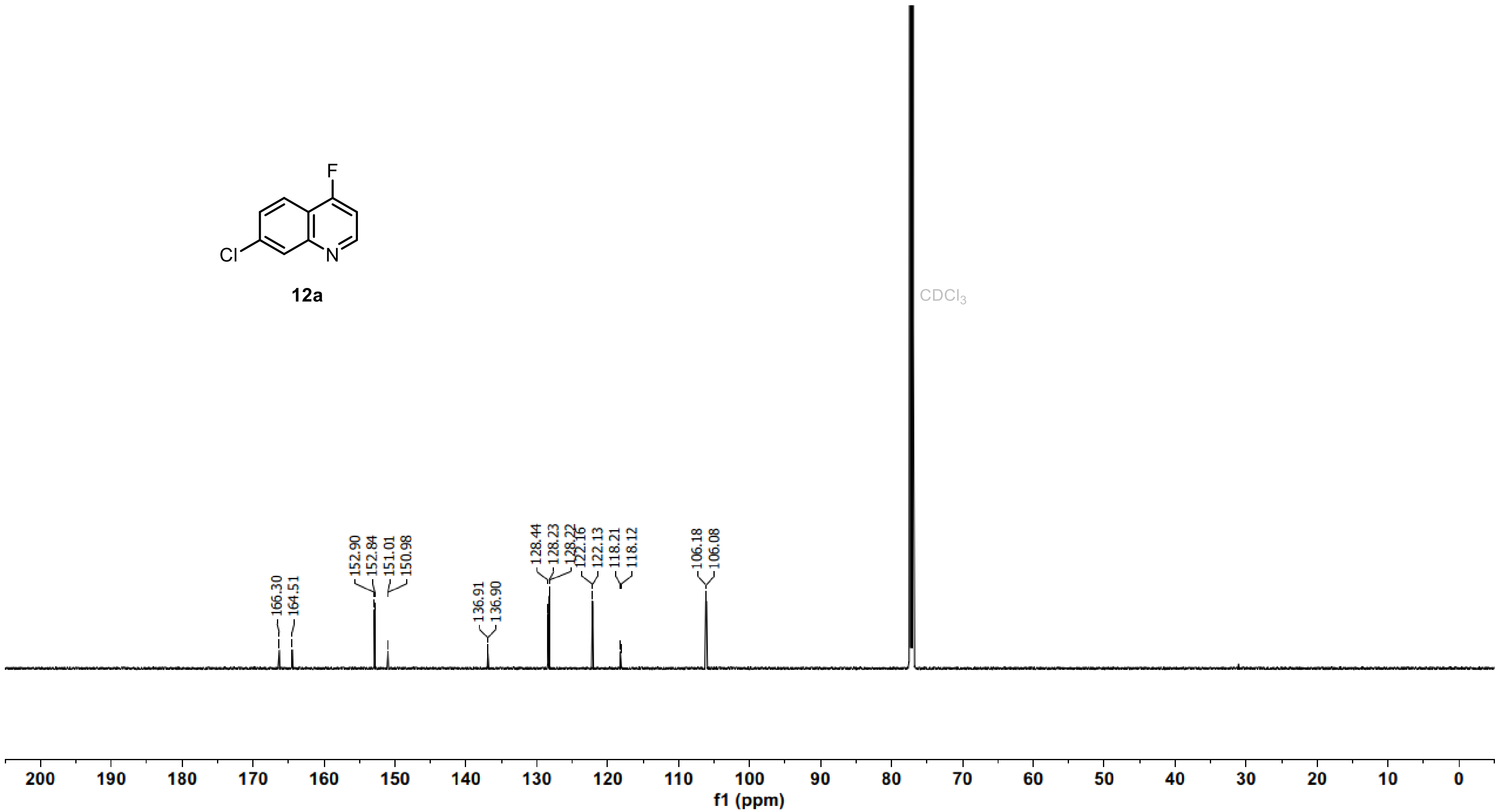

^{19}F NMR of 12a

CDCl$_3$, 471 MHz, 25 °C

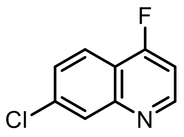

12a

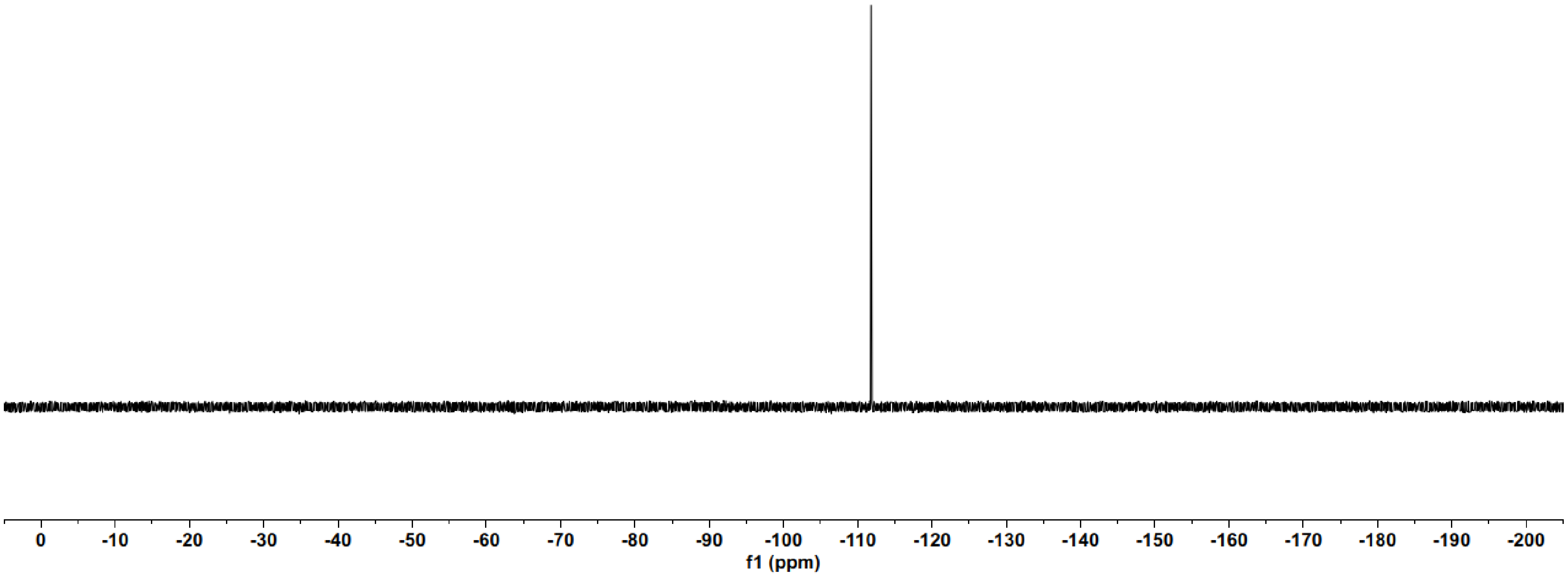

^{1}H NMR of 12b

$CDCl_3$, 500 MHz, 25 °C

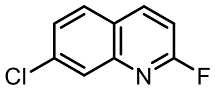

12b

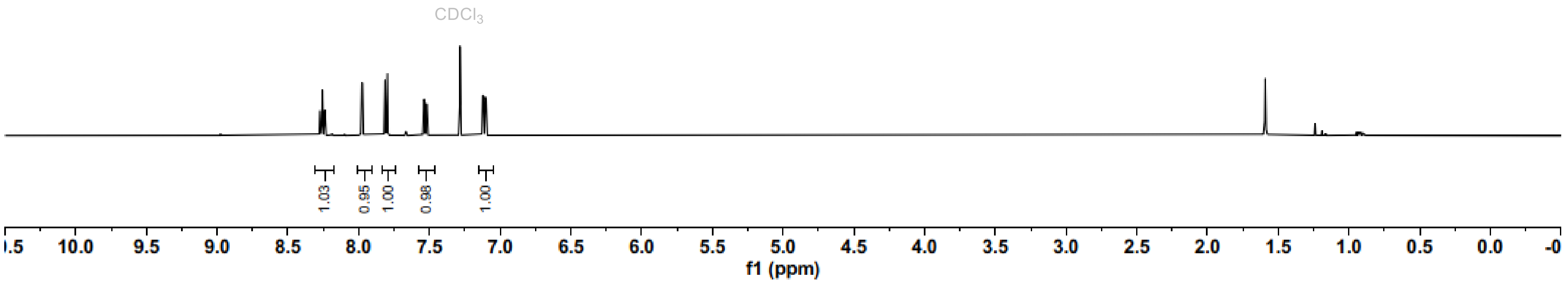

^{13}C NMR of 12b

$CDCl_3$, 126 MHz, 25 °C

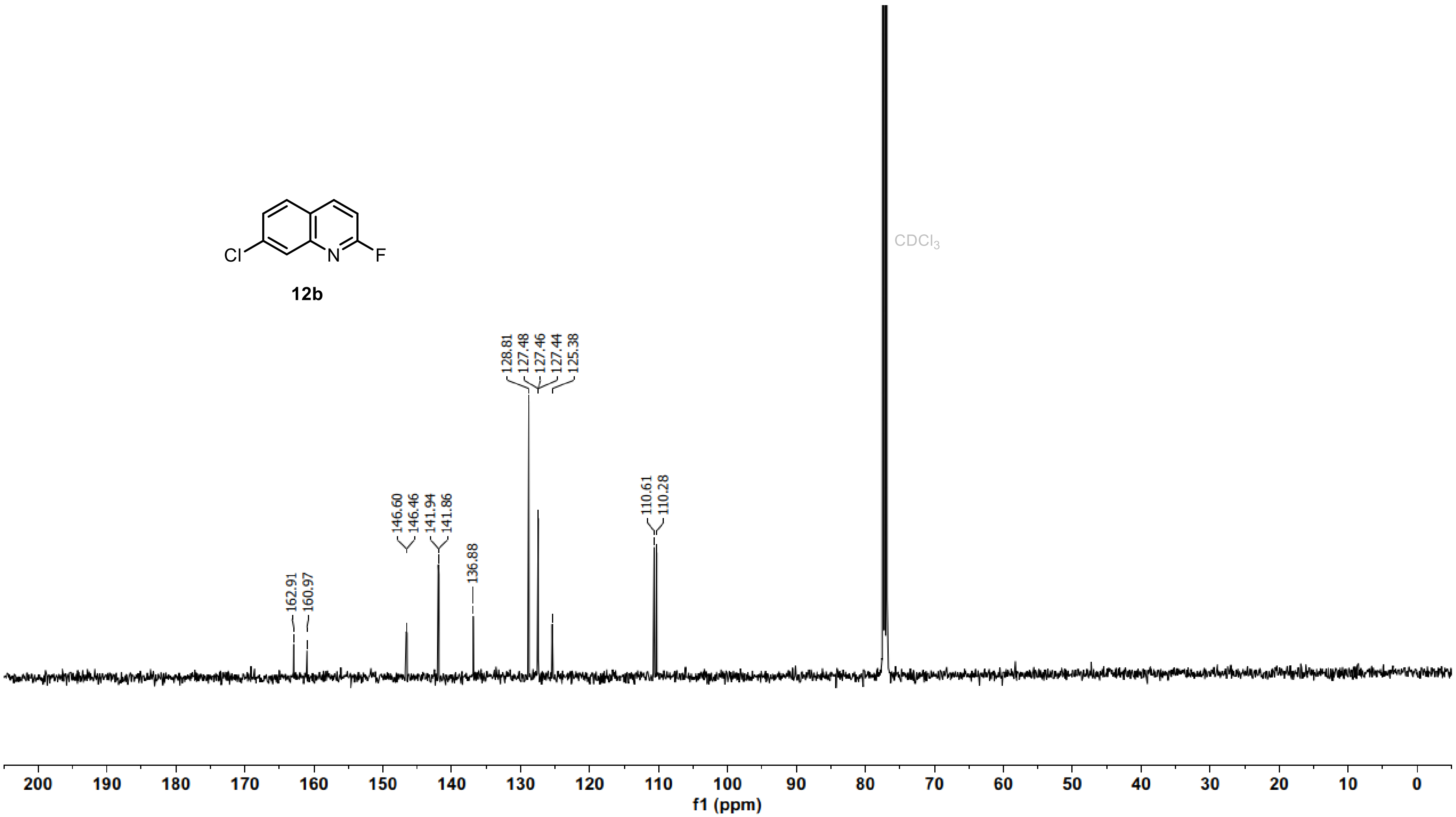

^{19}F NMR of 12b

CDCl$_3$, 471 MHz, 25 °C

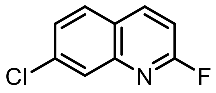

12b

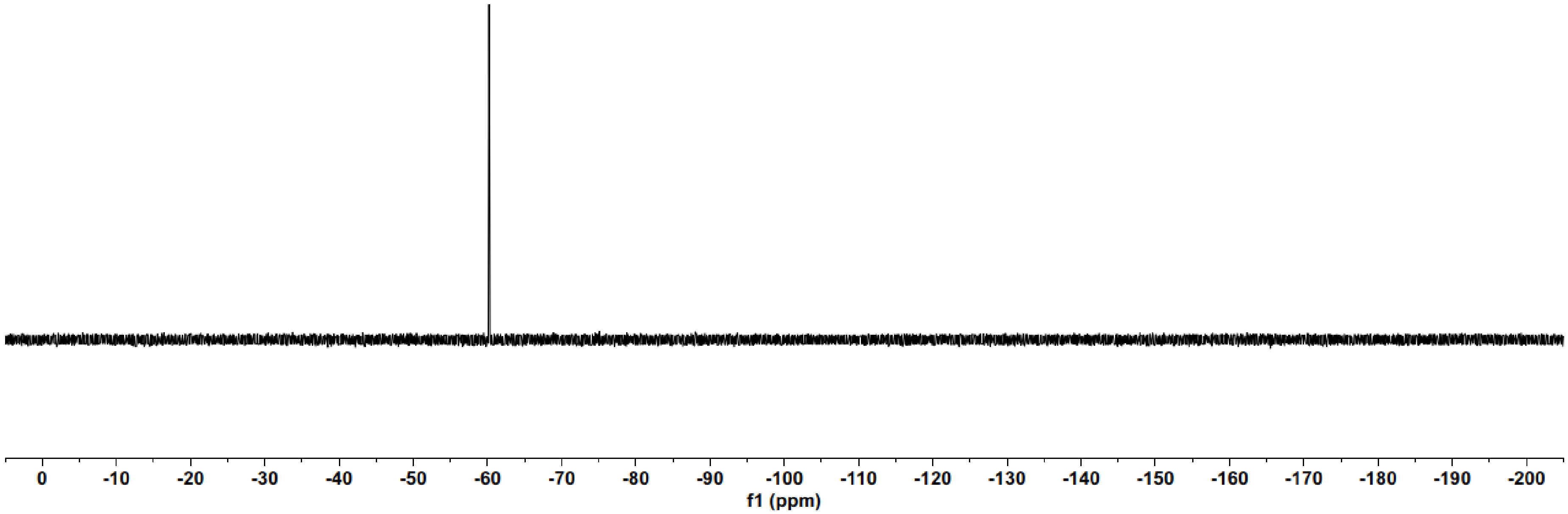

^{1}H NMR of 13

$CDCl_3$, 500 MHz, 25 °C

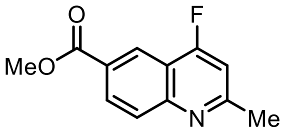

13

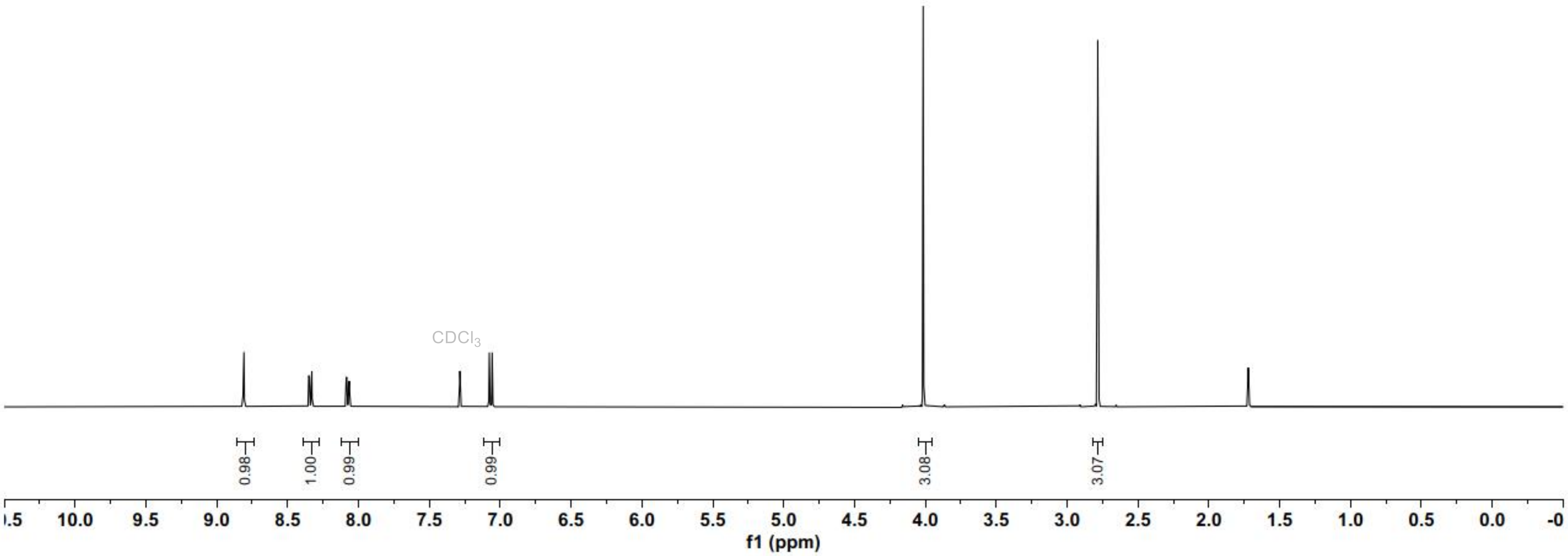

^{13}C NMR of 13

$CDCl_3$, 126 MHz, 25 °C

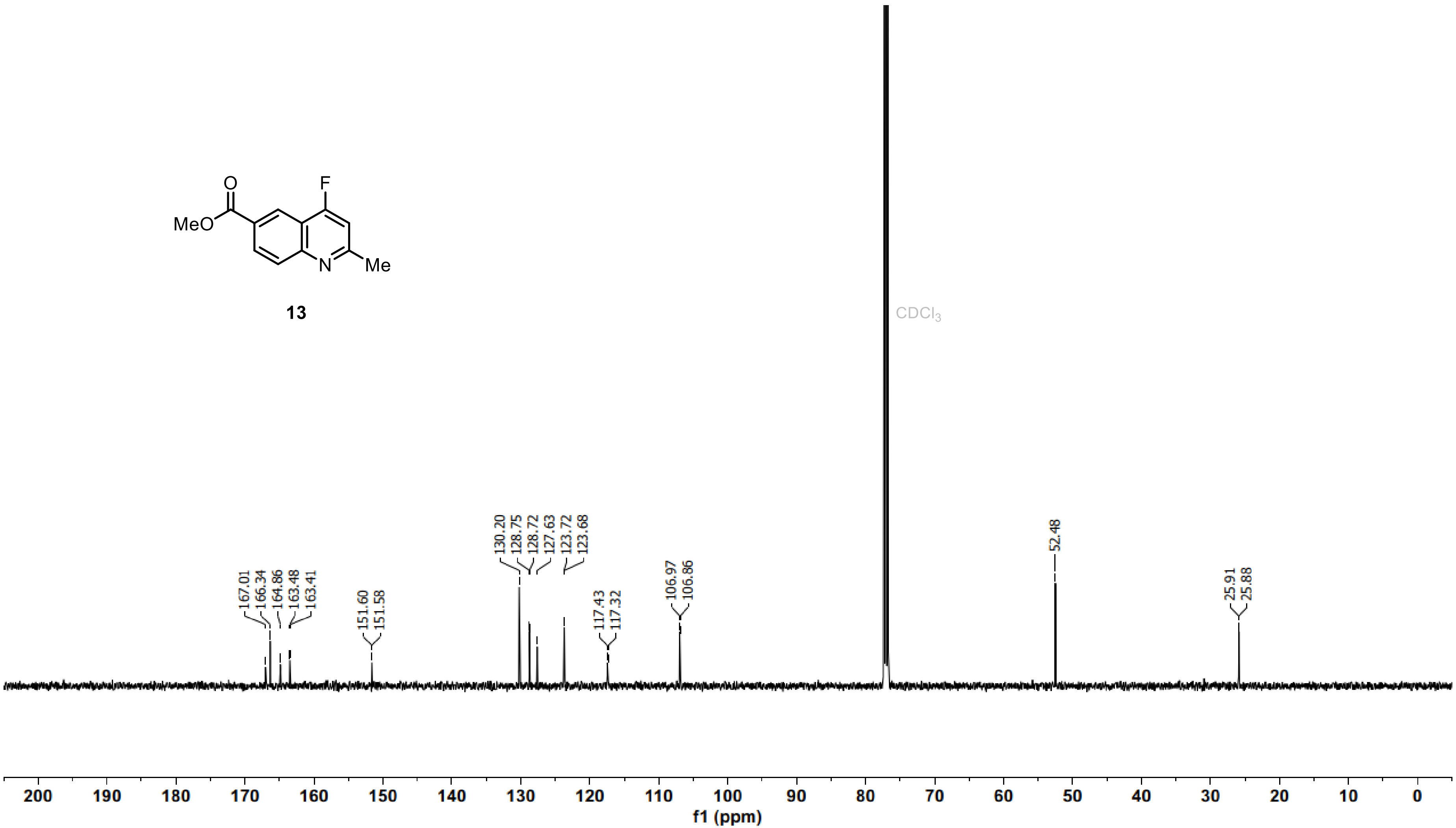

^{19}F NMR of 13

CDCl$_3$, 471 MHz, 25 °C

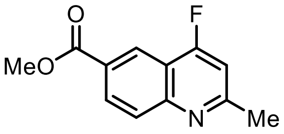

13

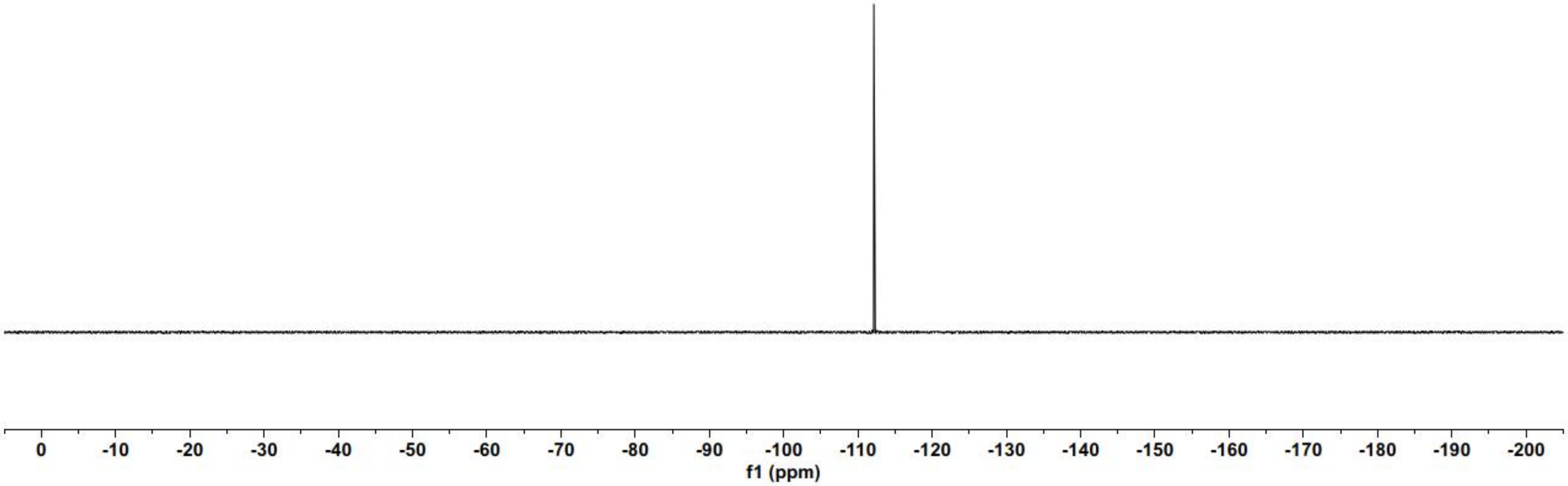

^{1}H NMR of 14

$CDCl_3$, 500 MHz, 25 °C

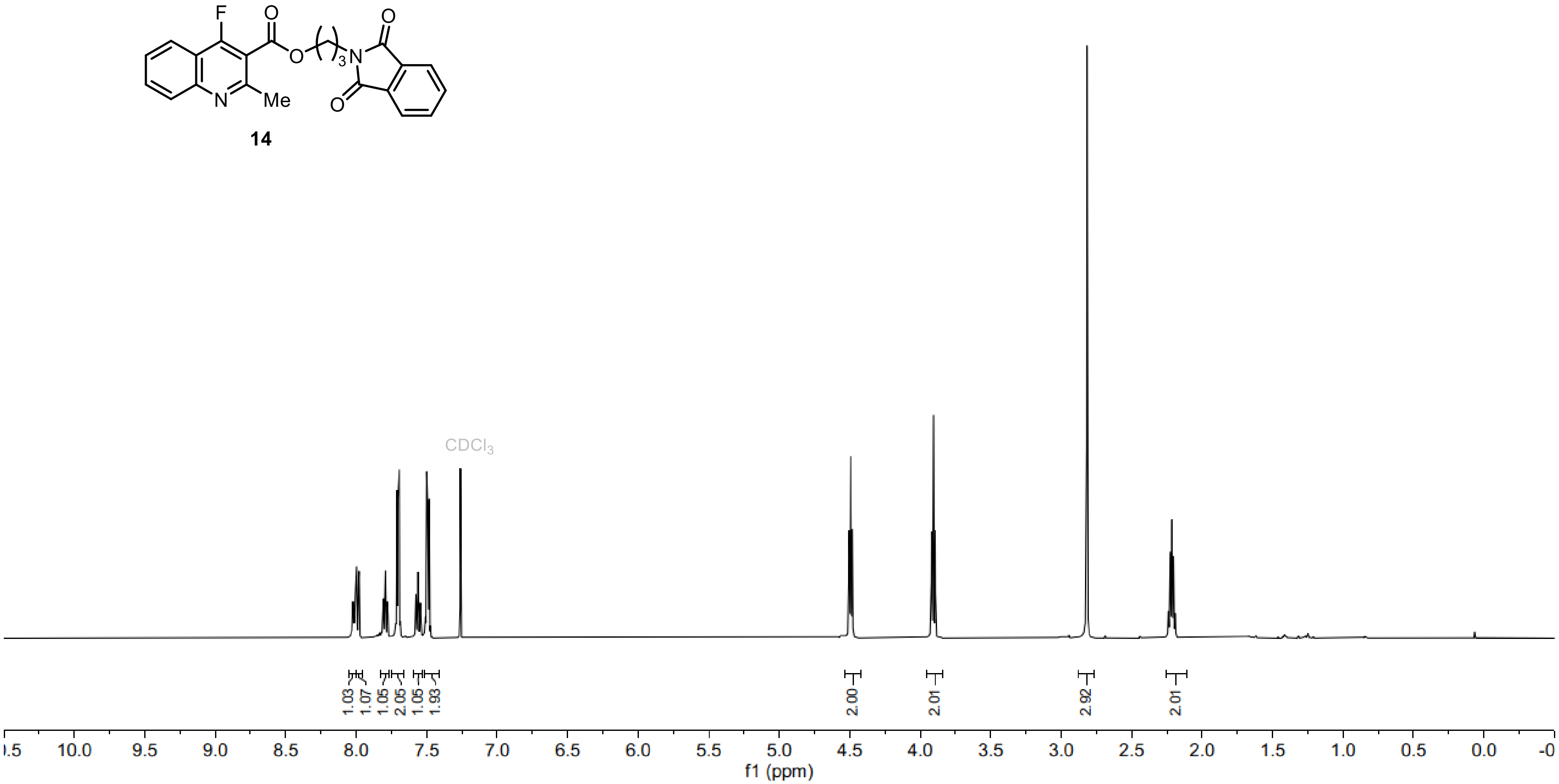

^{13}C NMR of 14

CDCl$_3$, 126 MHz, 25 °C

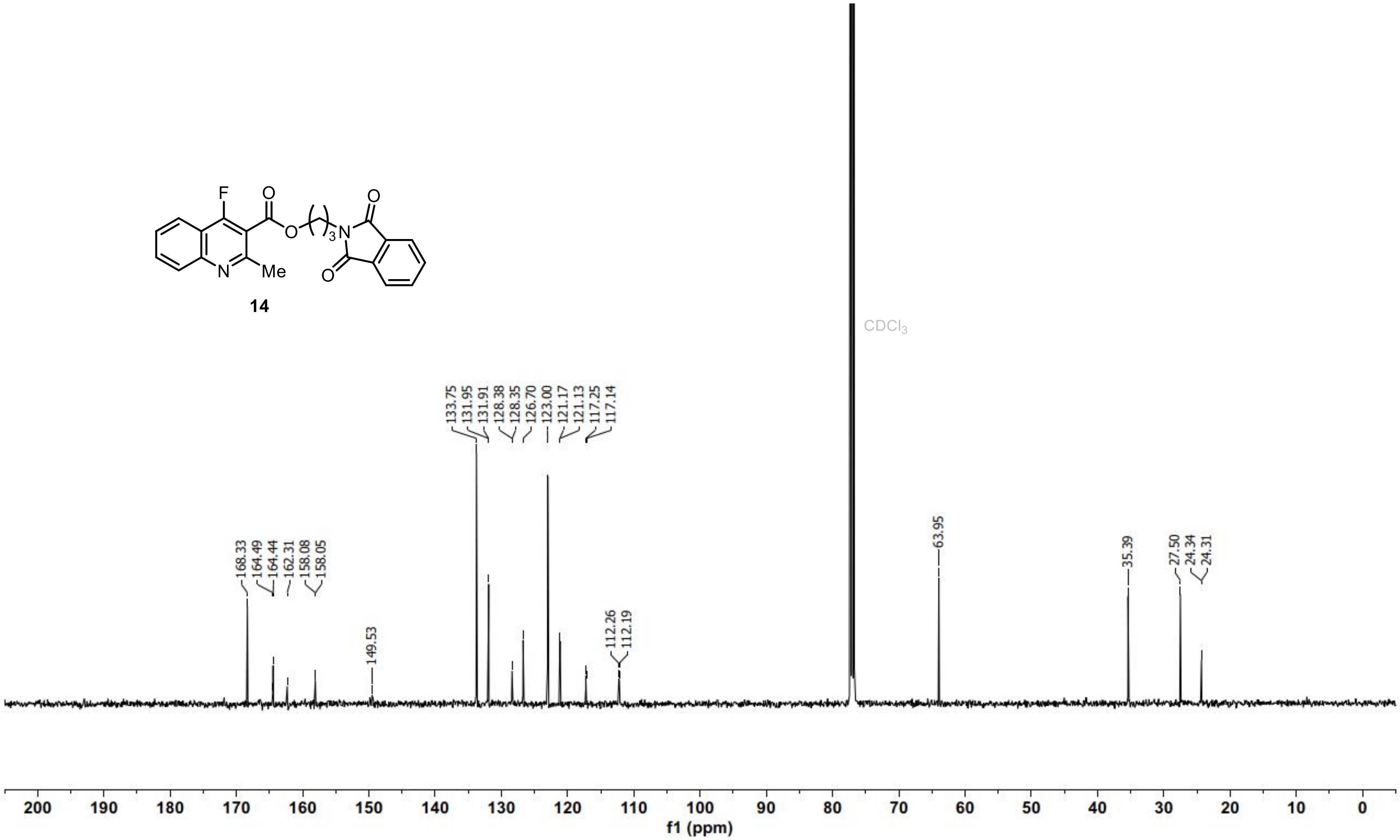

^{19}F NMR of 14

CDCl$_3$, 471 MHz, 25 °C

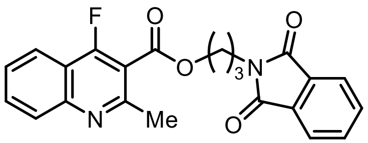

14

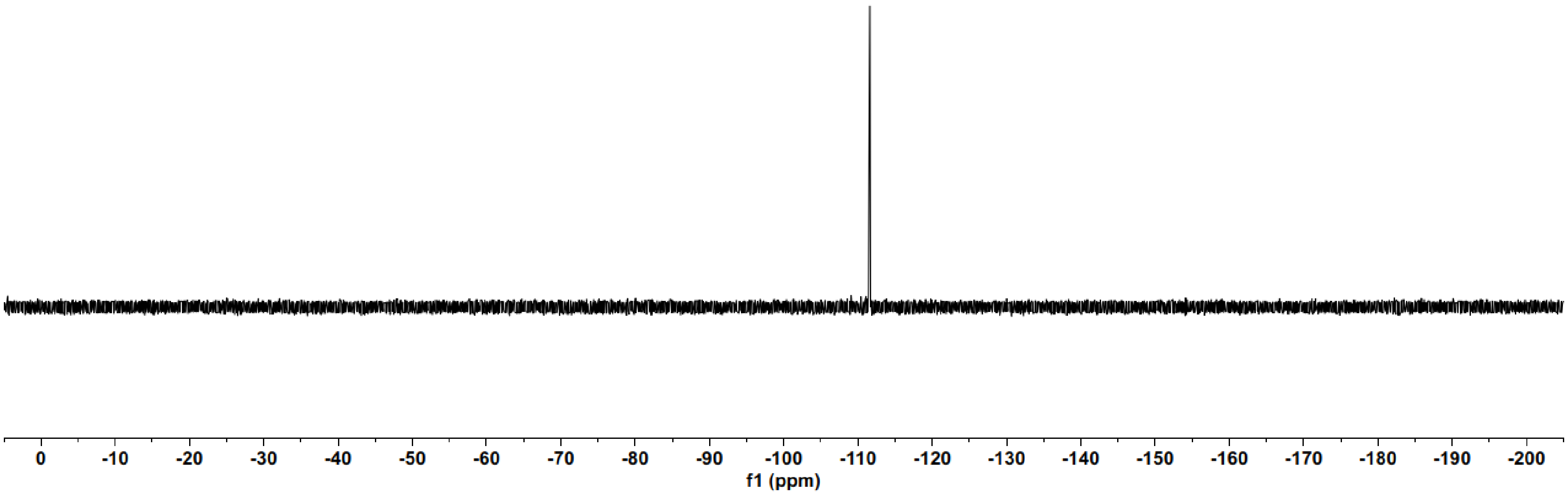

^{1}H NMR of 15

$CDCl_3$, 500 MHz, 25 °C

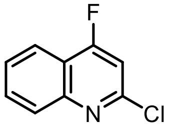

15

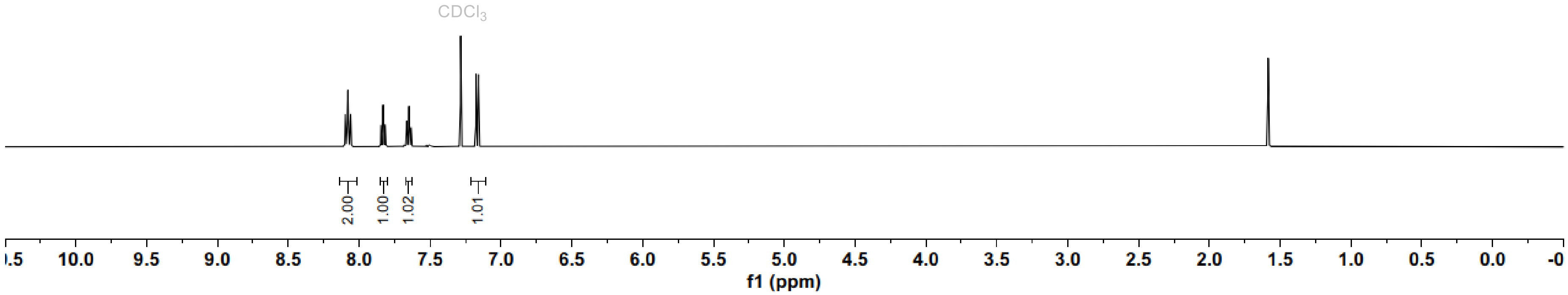

^{13}C NMR of 15

$CDCl_3$, 126 MHz, 25 °C

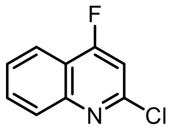

15

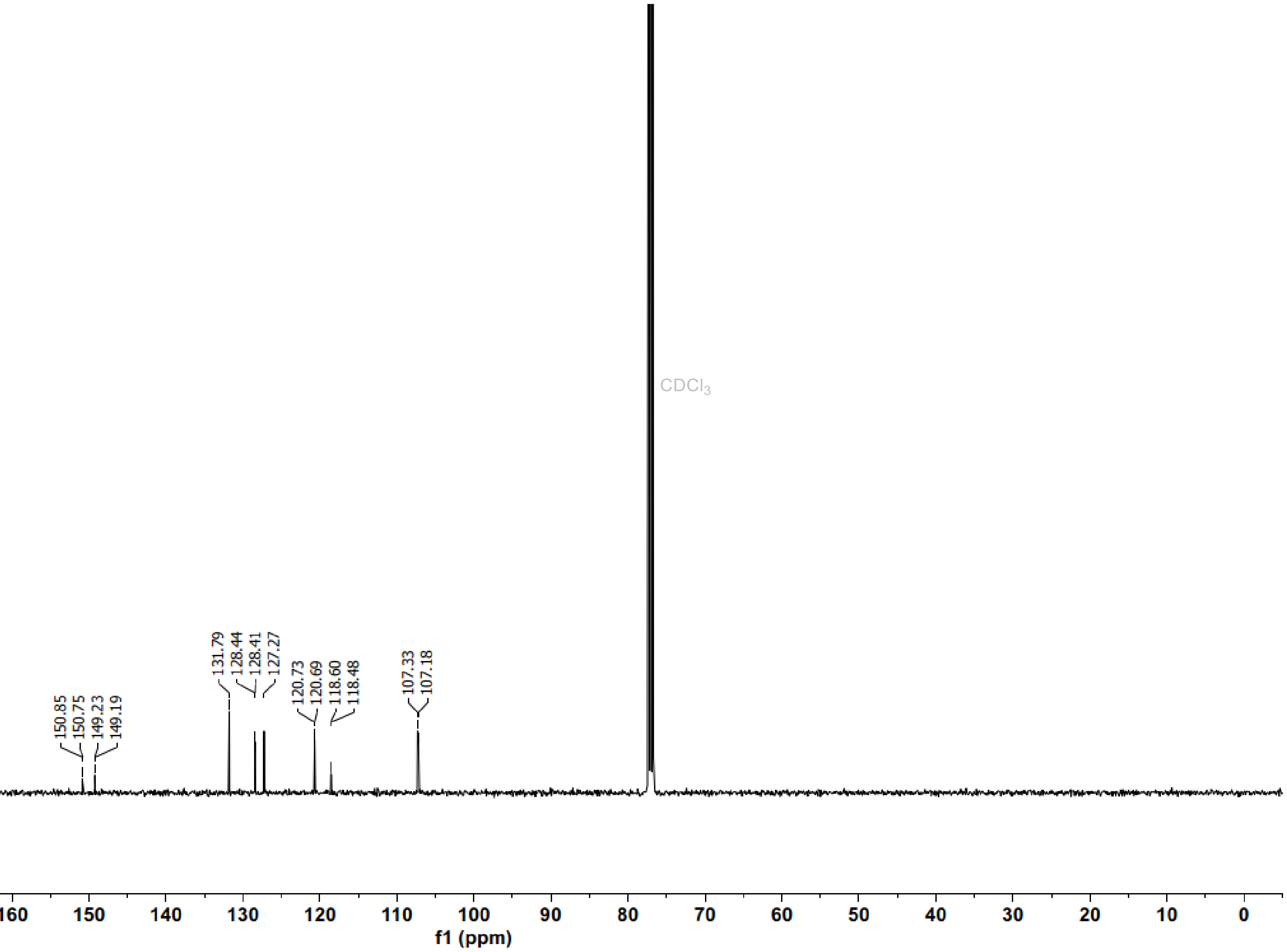

^{19}F NMR of 15

CDCl$_3$, 471 MHz, 25 °C

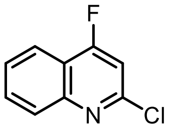

15

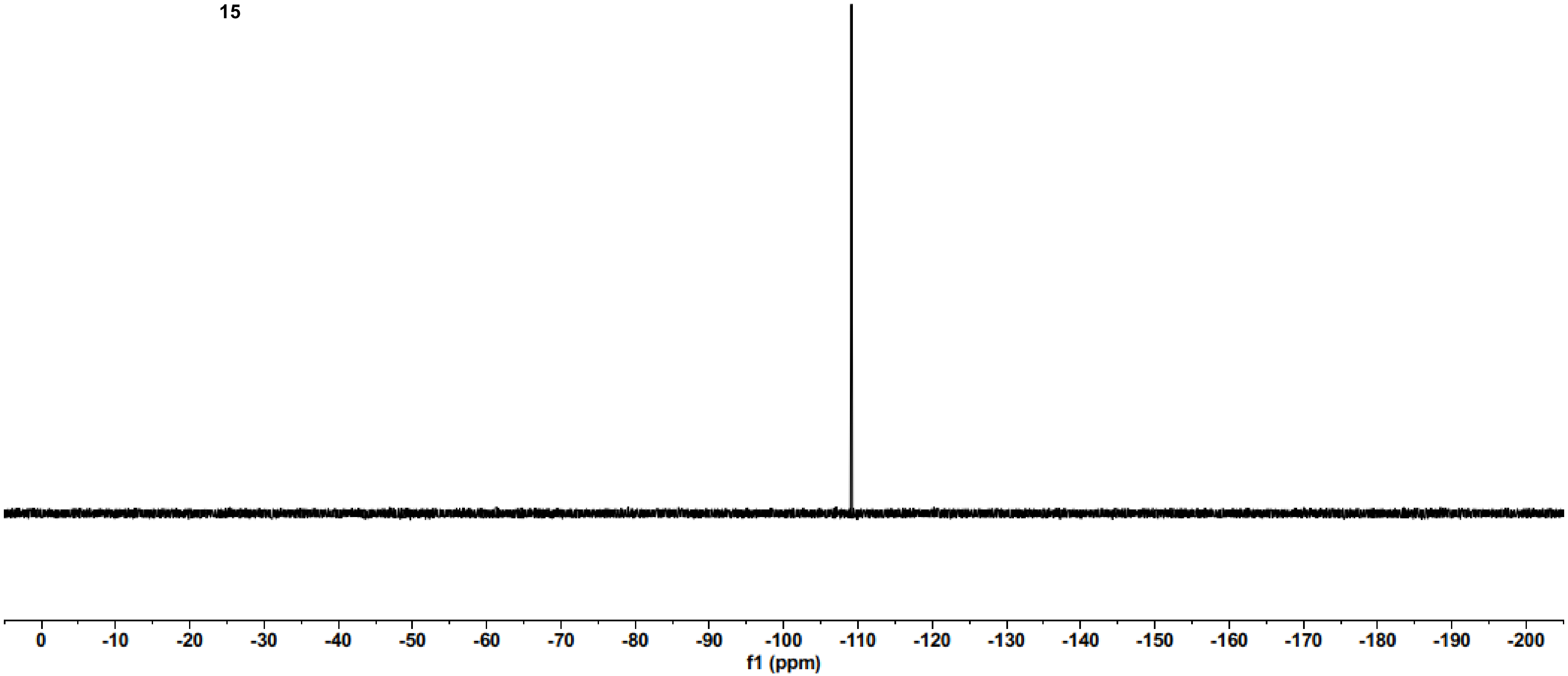

^{1}H NMR of 16

$CDCl_3$, 500 MHz, 25 °C

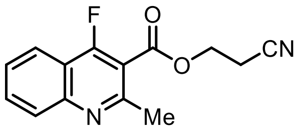

16

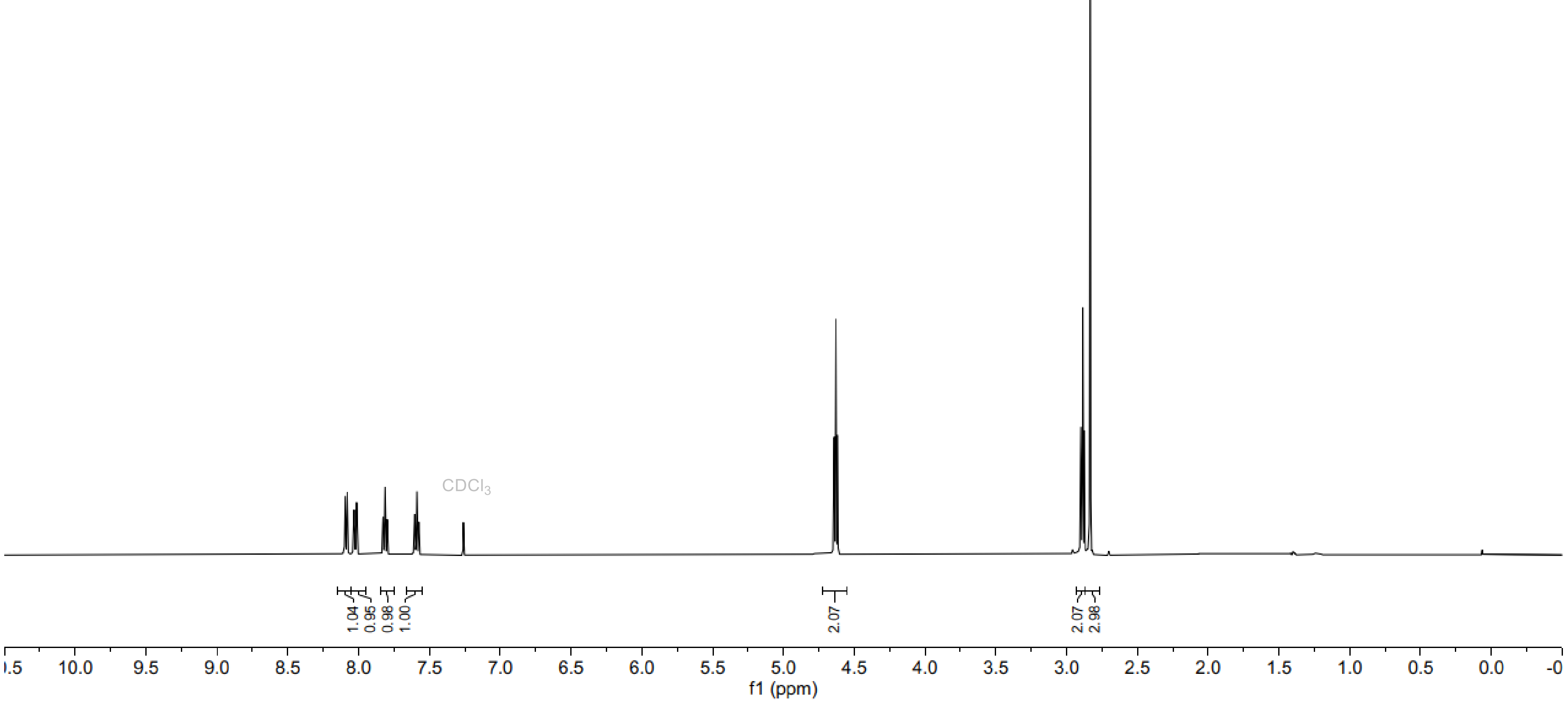

^{13}C NMR of 16

$CDCl_3$, 126 MHz, 25 °C

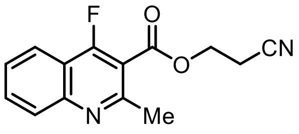

16

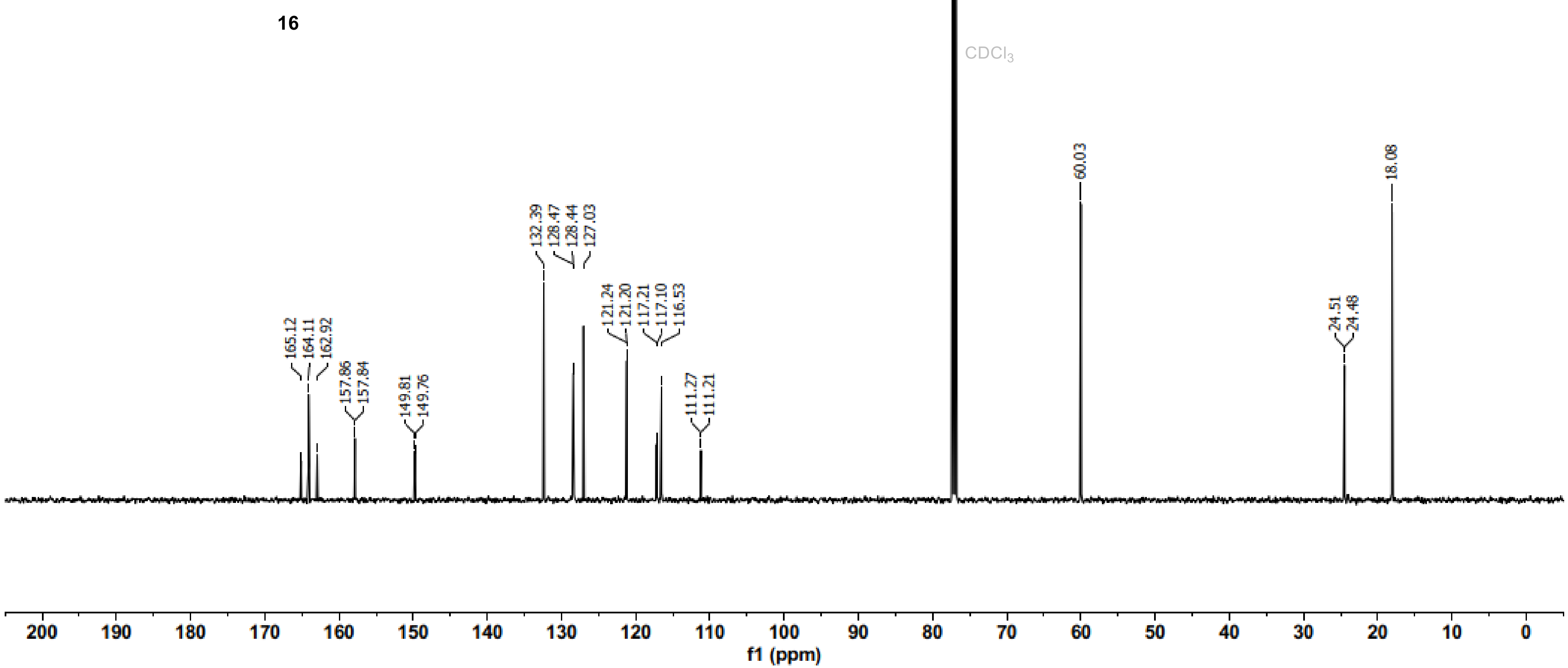

^{19}F NMR of 16

$CDCl_3$, 471 MHz, 25 °C

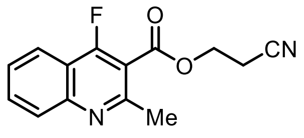

16

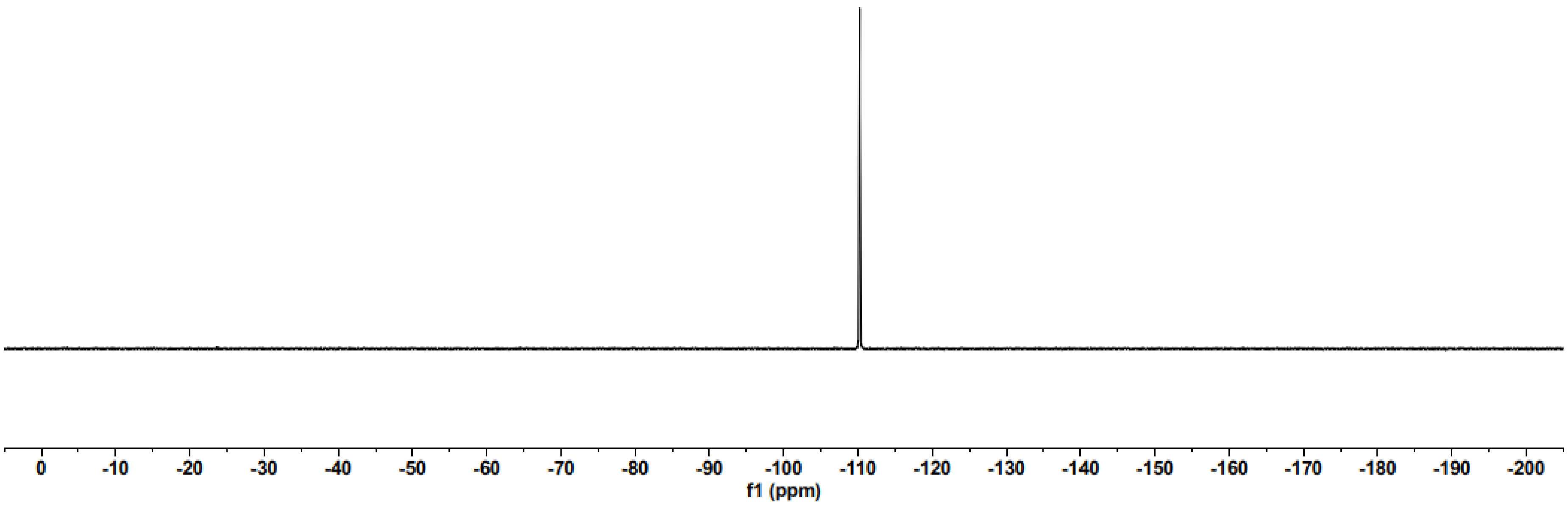

^{1}H NMR of 17

$CDCl_3$, 500 MHz, 25 °C

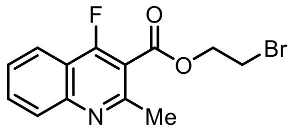

17

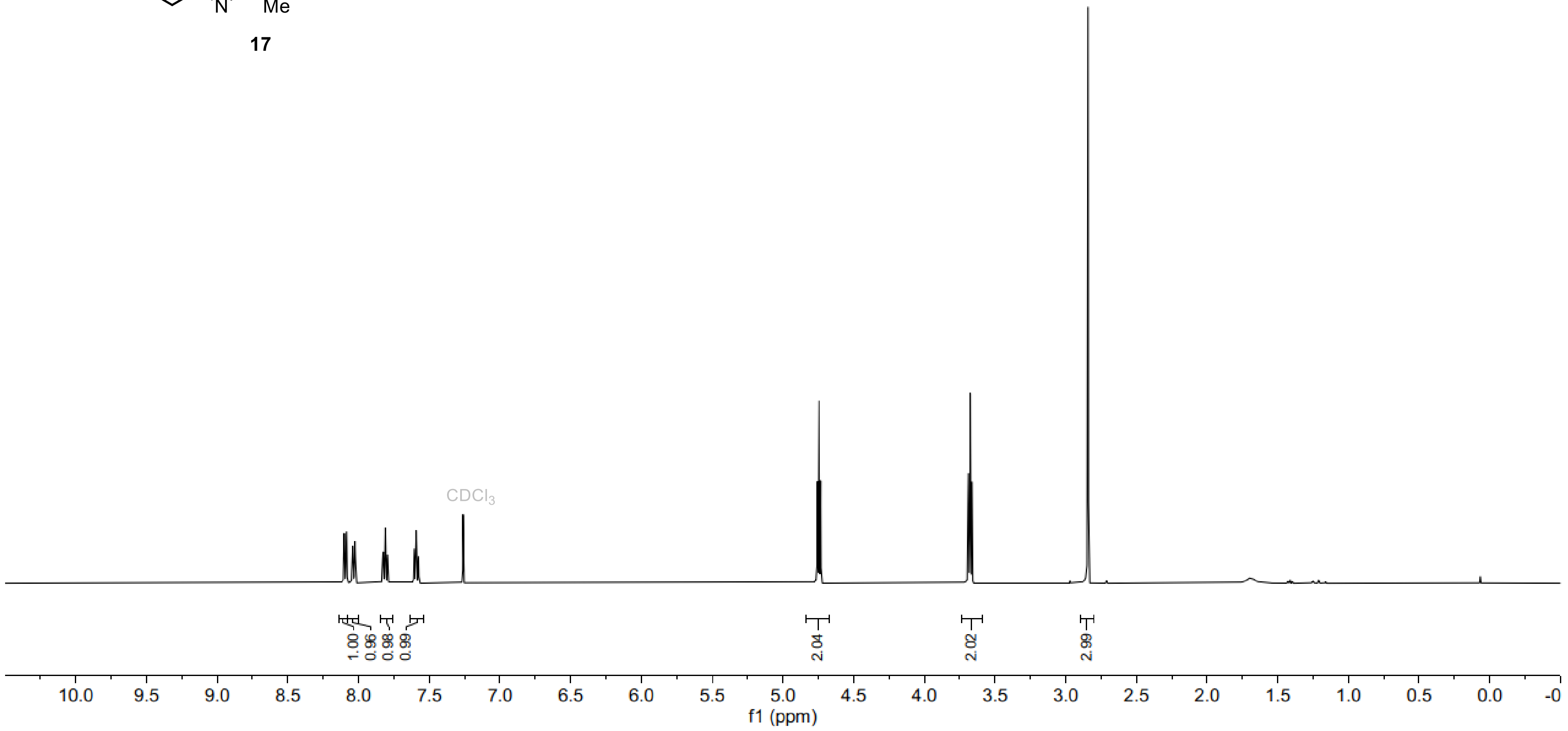

^{13}C NMR of 17

$CDCl_3$, 126 MHz, 25 °C

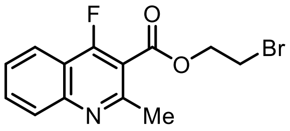

17

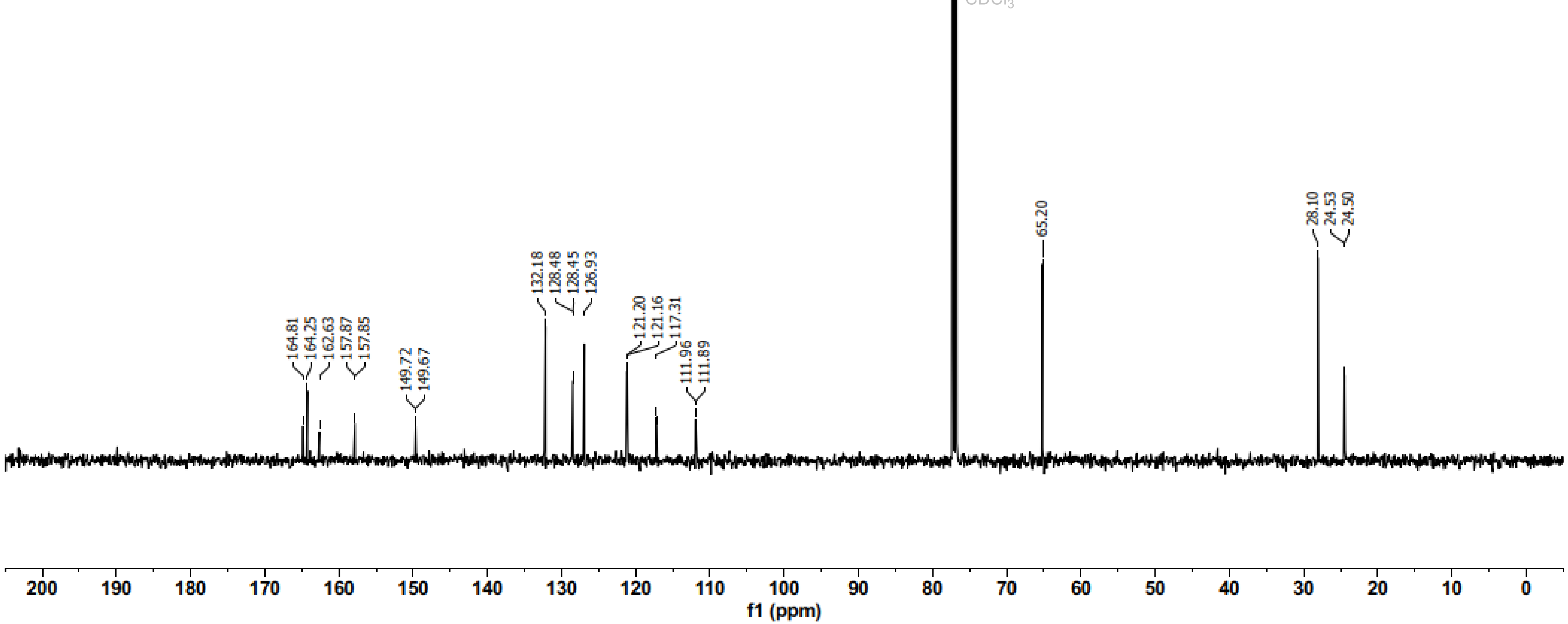

^{19}F NMR of 17

$CDCl_3$, 471 MHz, 25 °C

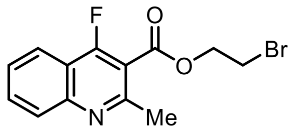

17

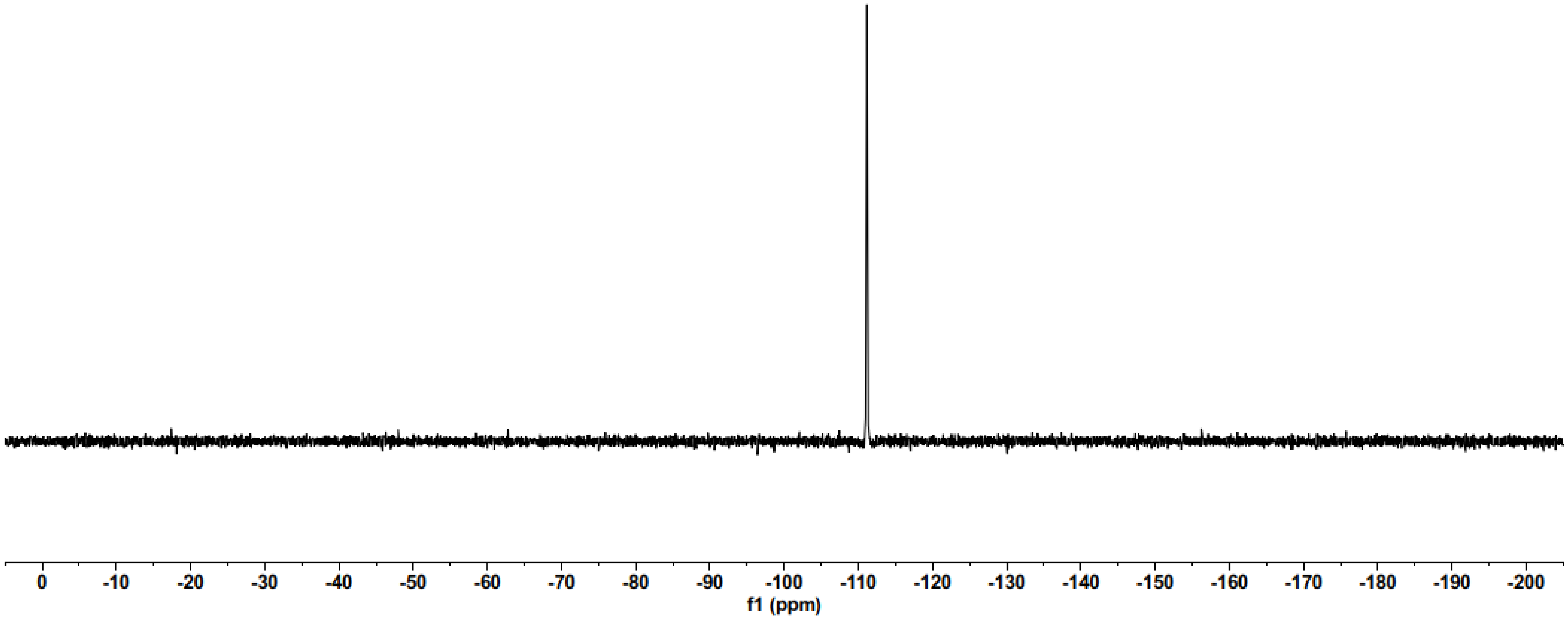

^{1}H NMR of 18

$CDCl_3$, 500 MHz, 25 °C

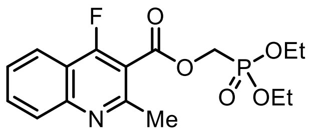

18

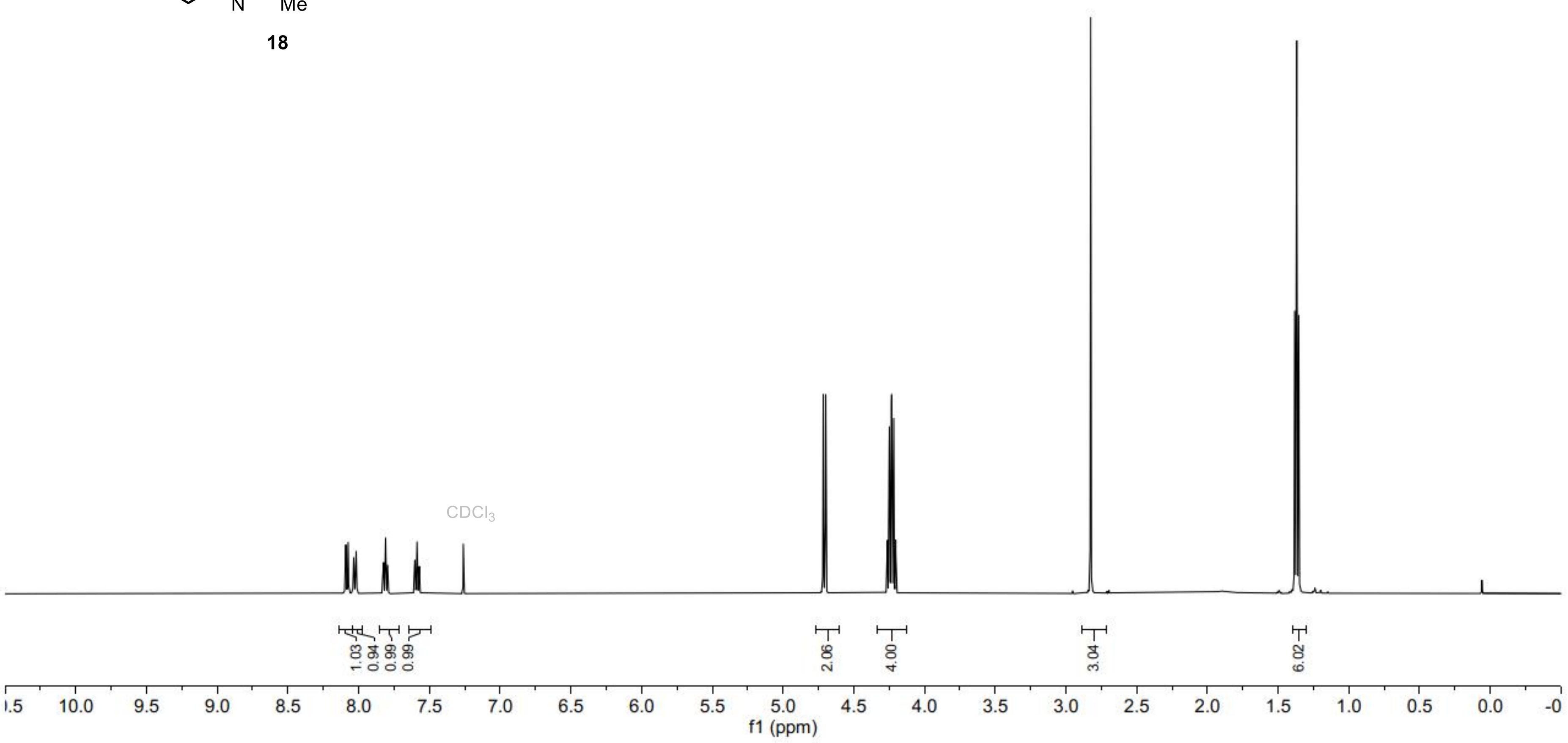

^{13}C NMR of 18

$CDCl_3$, 126 MHz, 25 °C

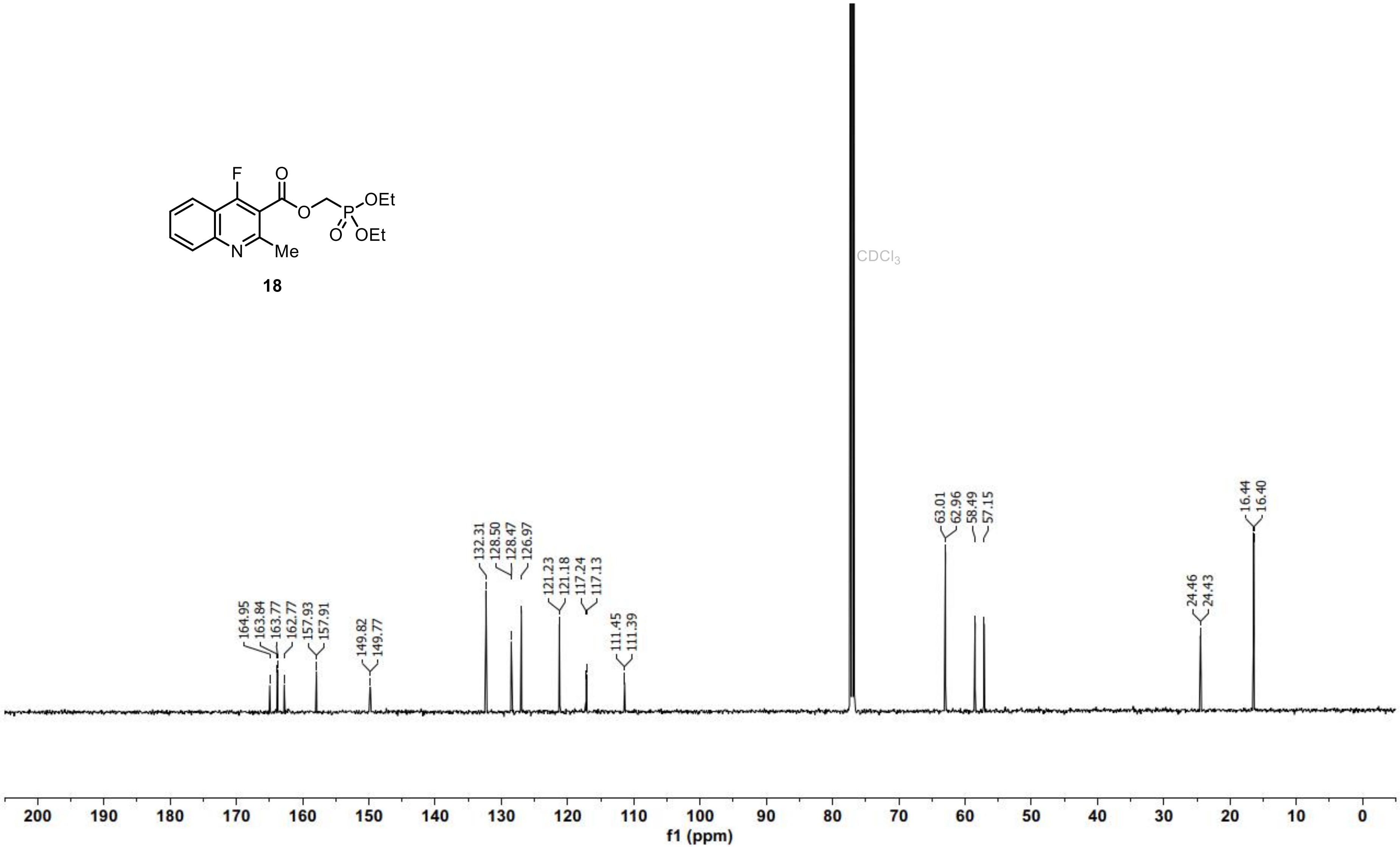

^{19}F NMR of 18

CDCl$_3$, 471 MHz, 25 °C

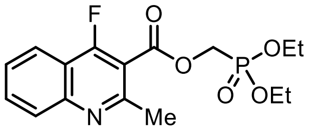

18

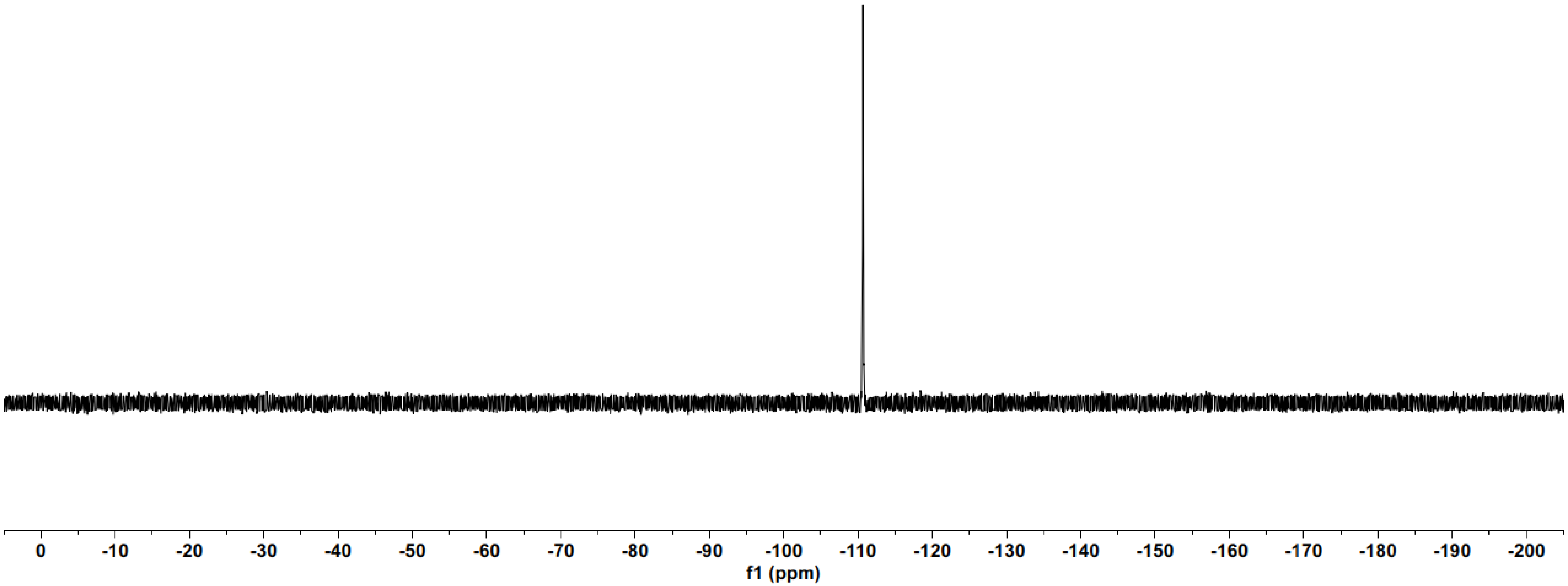

^{31}P NMR of 18

$CDCl_3$, 203 MHz, 25 °C

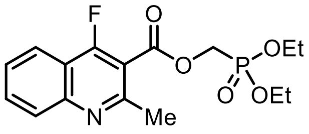

18

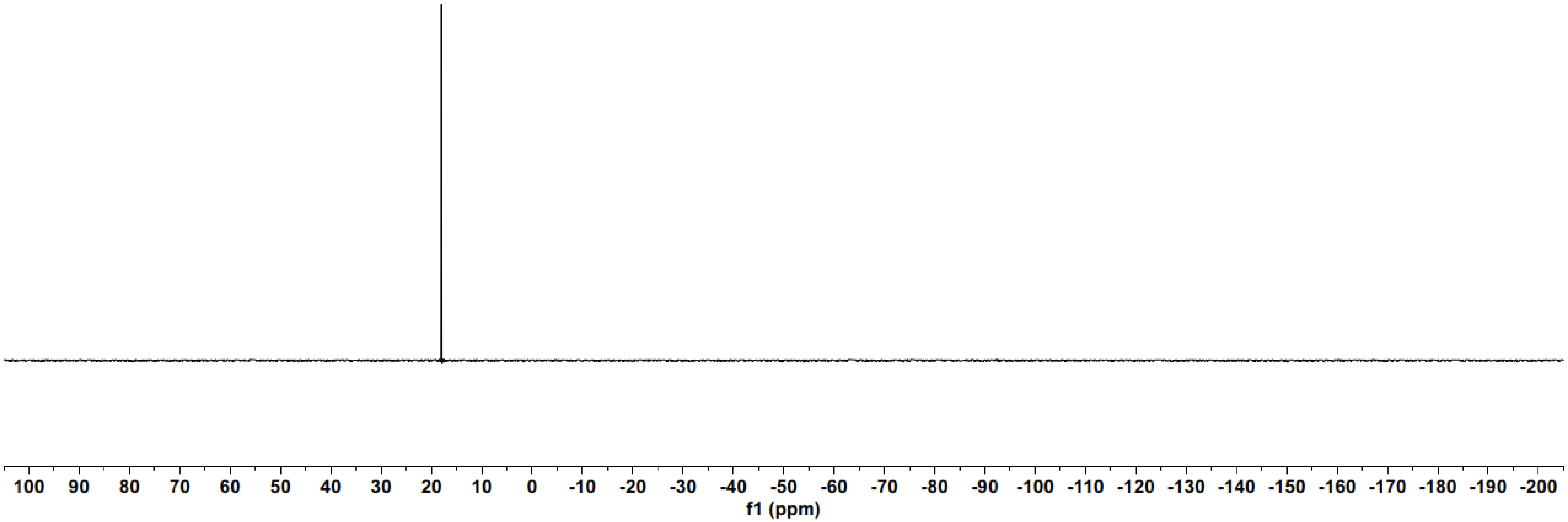

^{1}H NMR of 19a

$CDCl_3$, 500 MHz, 25 °C

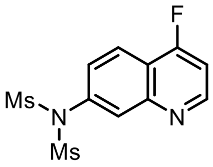

19a

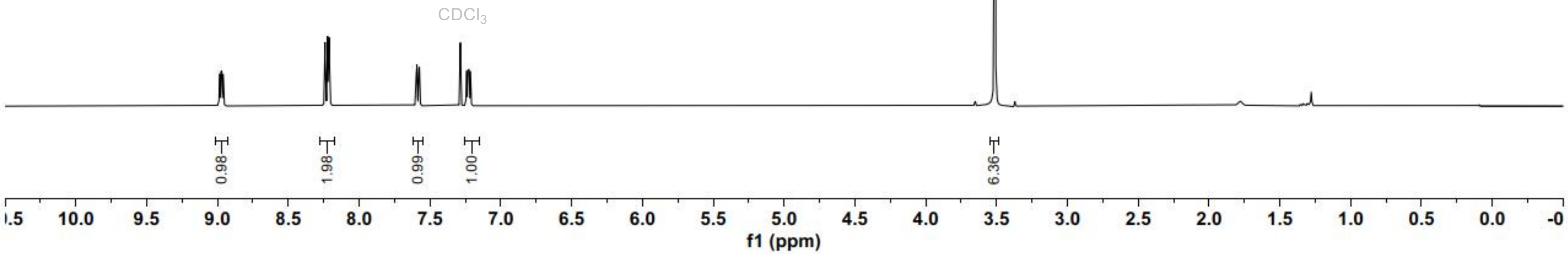

^{13}C NMR of 19a

$CDCl_3$, 126 MHz, 25 °C

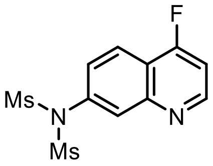

19a

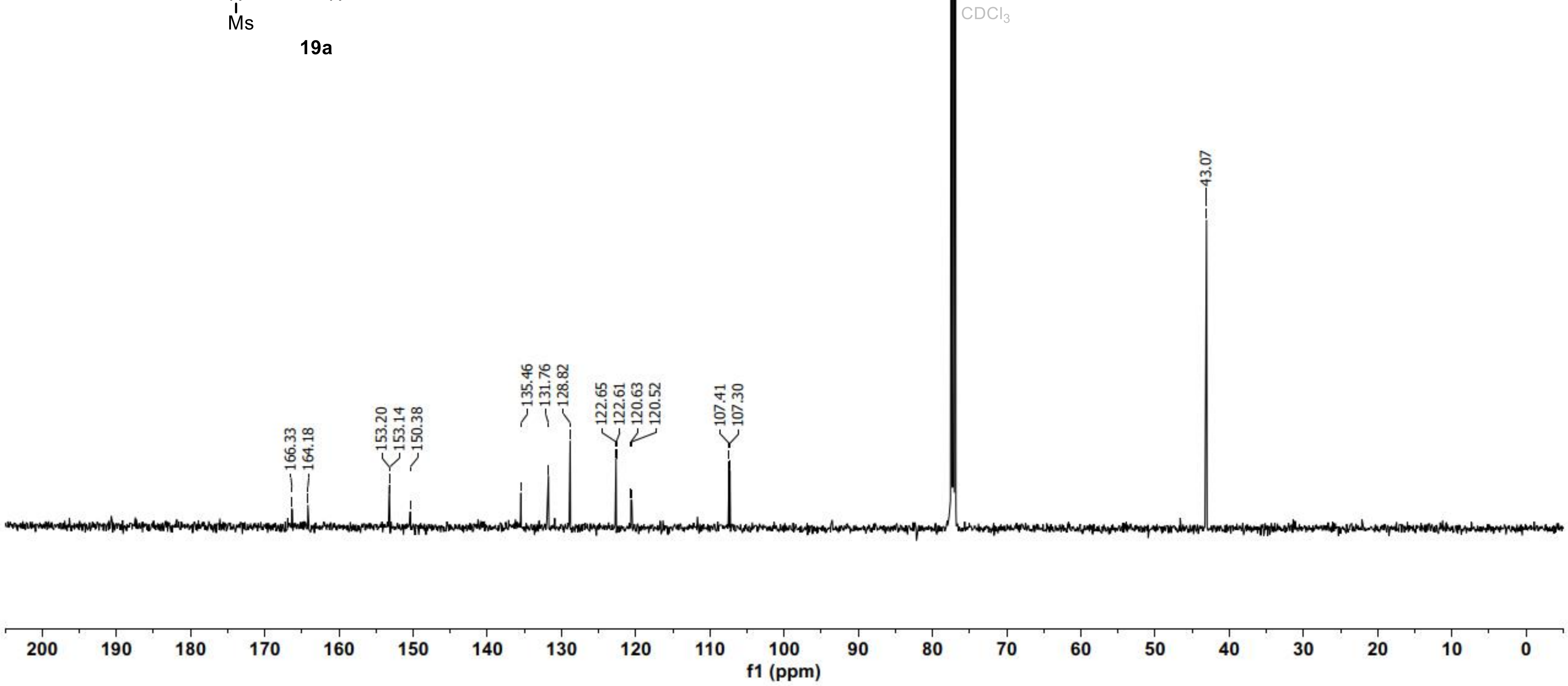

^{19}F NMR of 19a

$CDCl_3$, 471 MHz, 25 °C

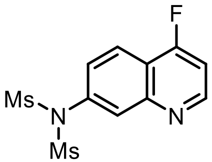

19a

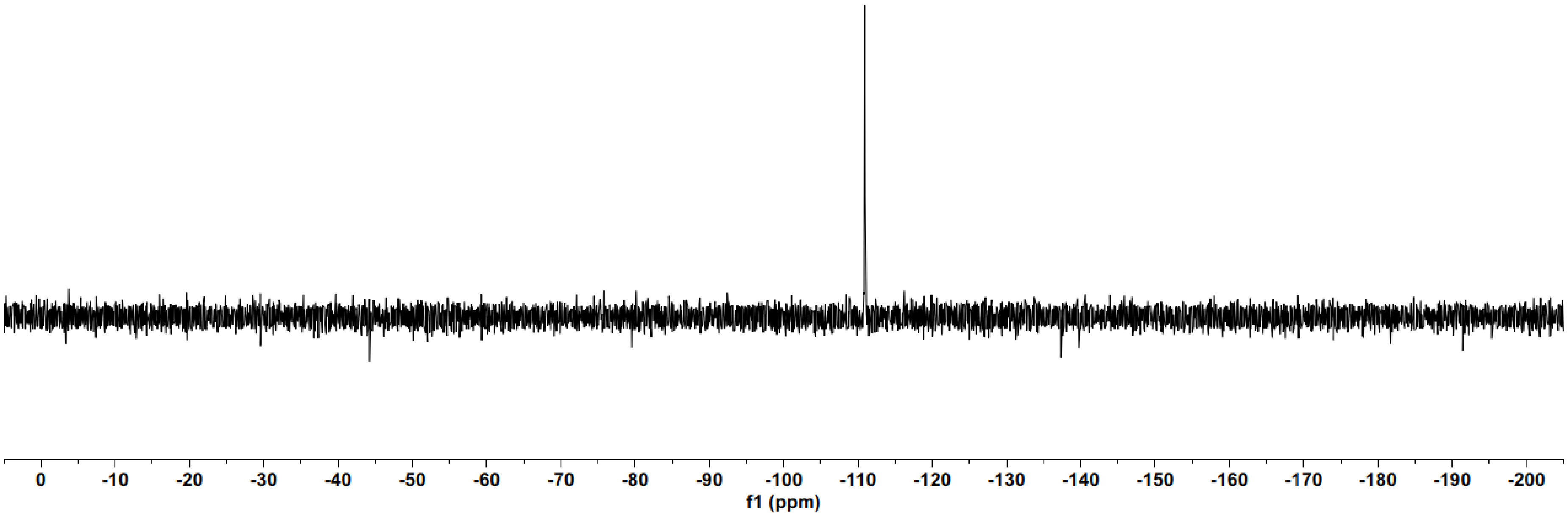

^{1}H NMR of 19b

$CDCl_3$, 500 MHz, 25 °C

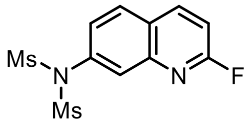

19b

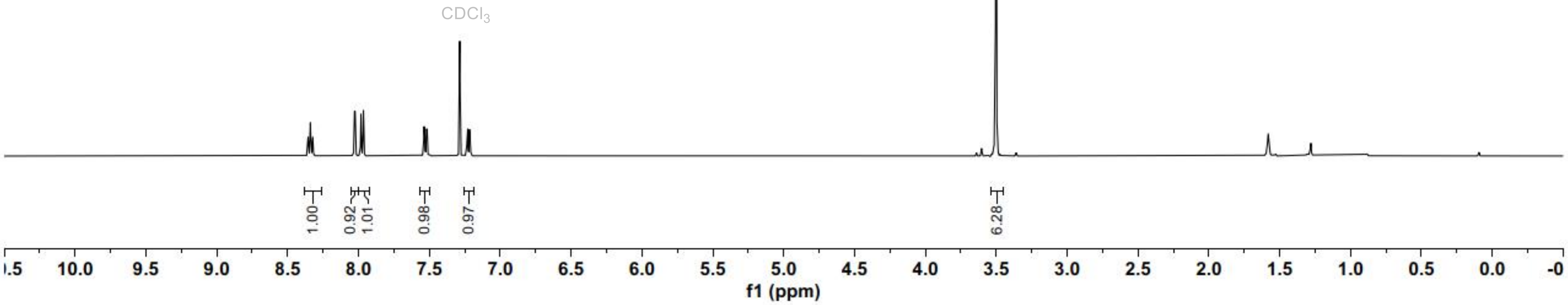

^{13}C NMR of 19b

$CDCl_3$, 126 MHz, 25 °C

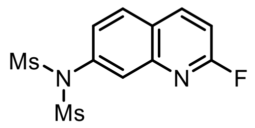

19b

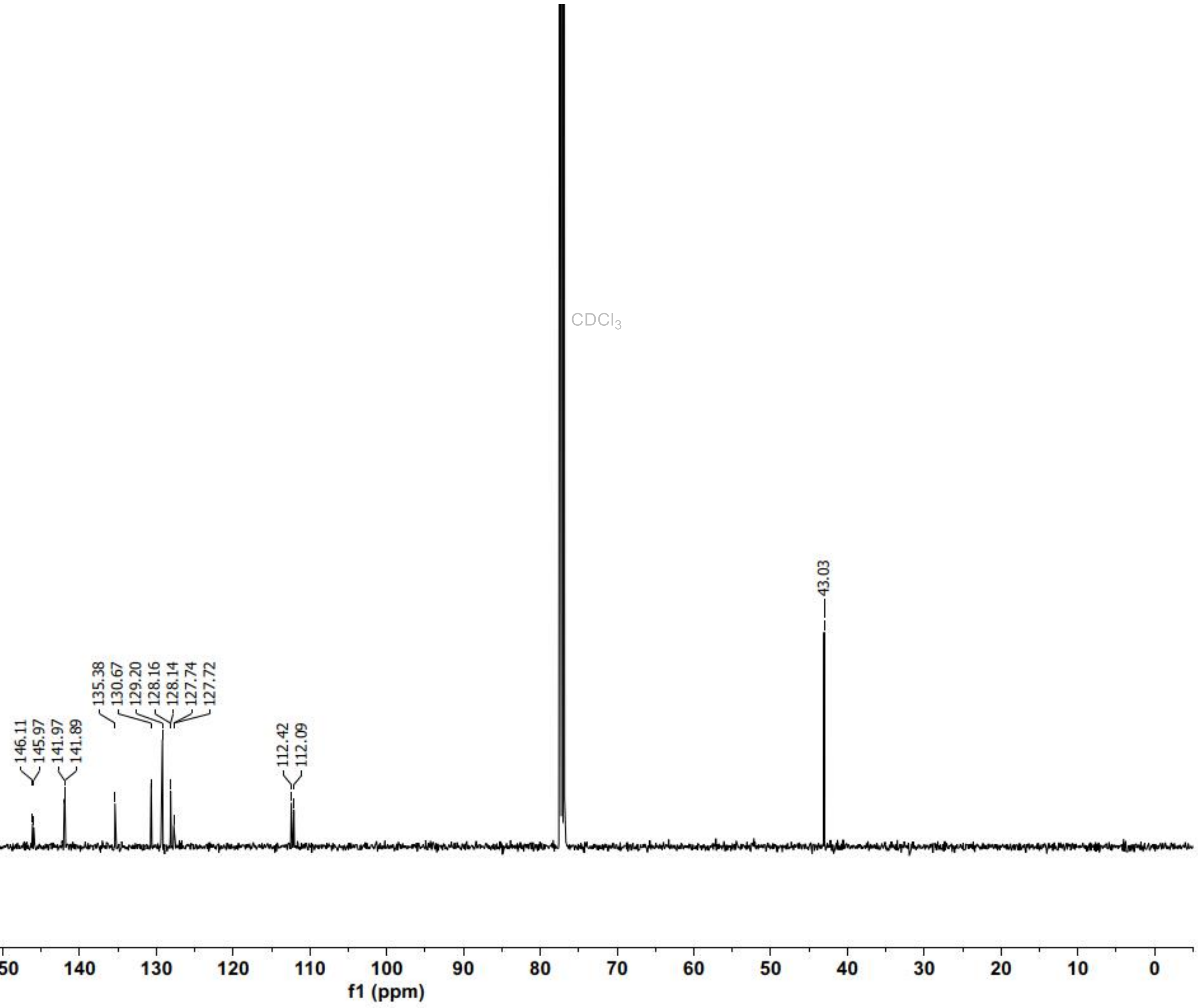

^{19}F NMR of 19b

$CDCl_3$, 471 MHz, 25 °C

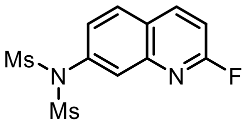

19b

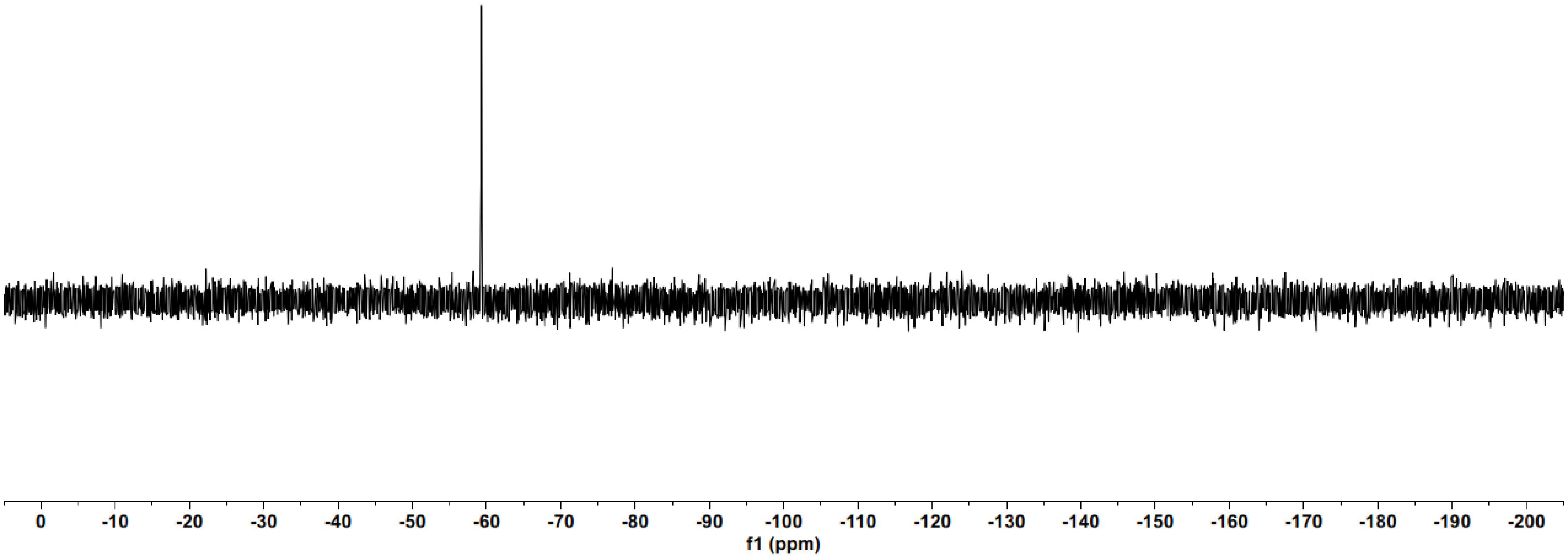

^{1}H NMR of 20a

$CDCl_3$, 500 MHz, 25 °C

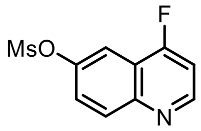

20a

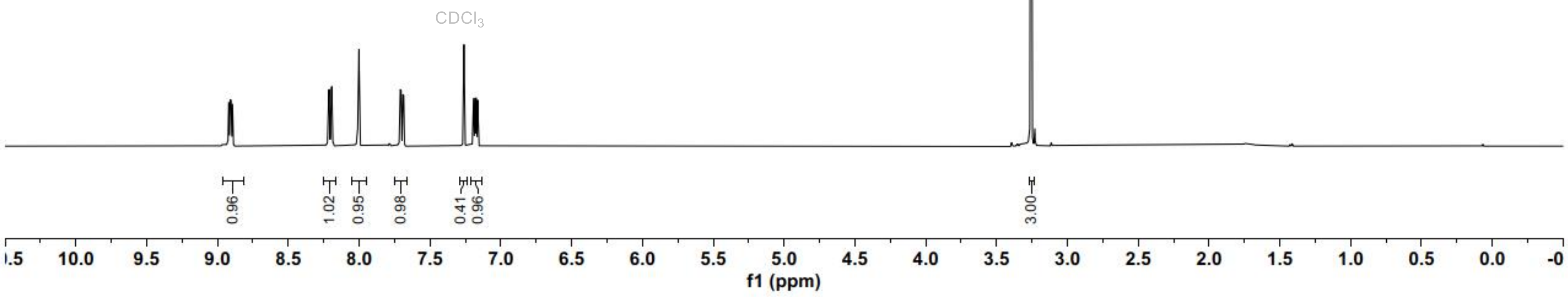

^{13}C NMR of 20a

$CDCl_3$, 126 MHz, 25 °C

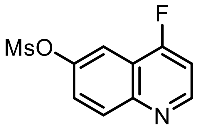

20a

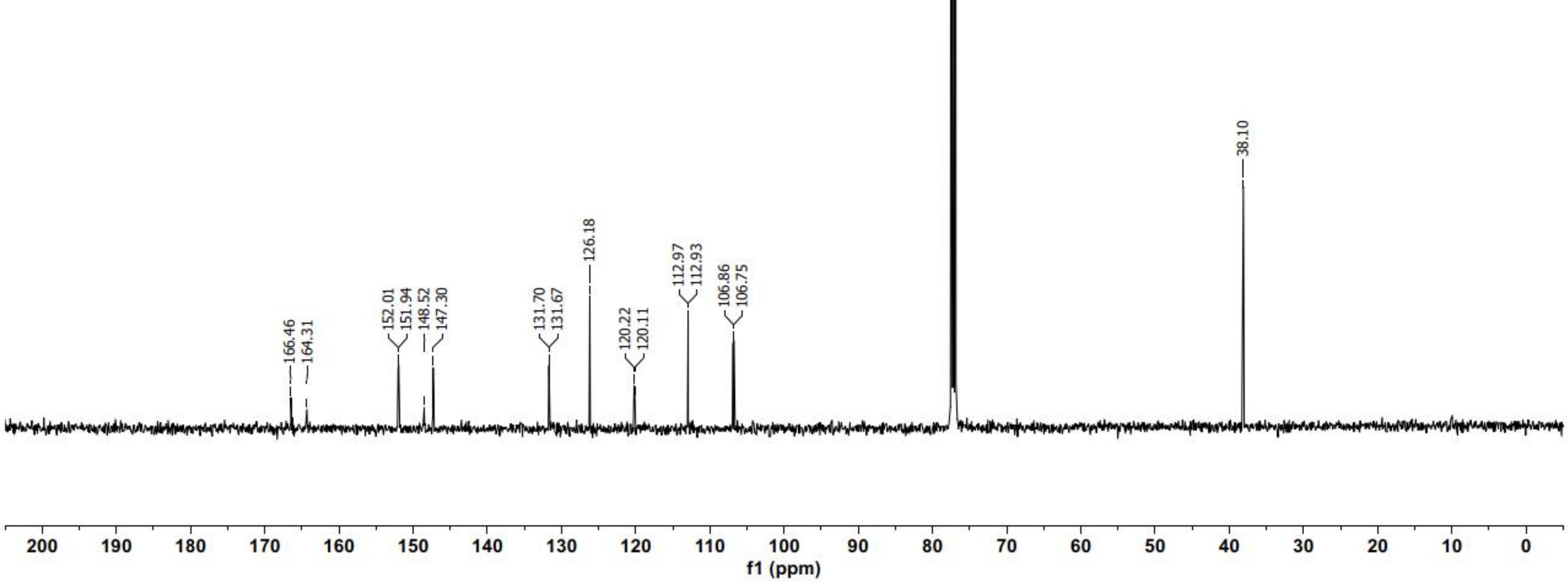

^{19}F NMR of 20a

$CDCl_3$, 471 MHz, 25 °C

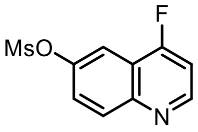

20a

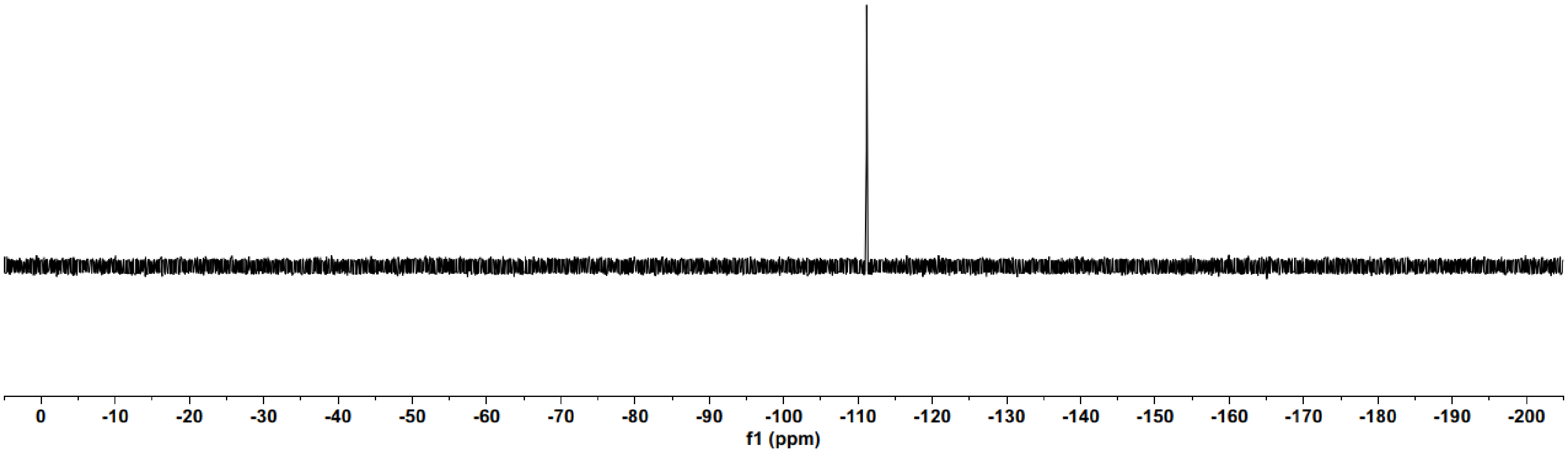

^{1}H NMR of 20b

$CDCl_3$, 500 MHz, 25 °C

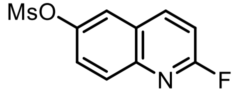

20b

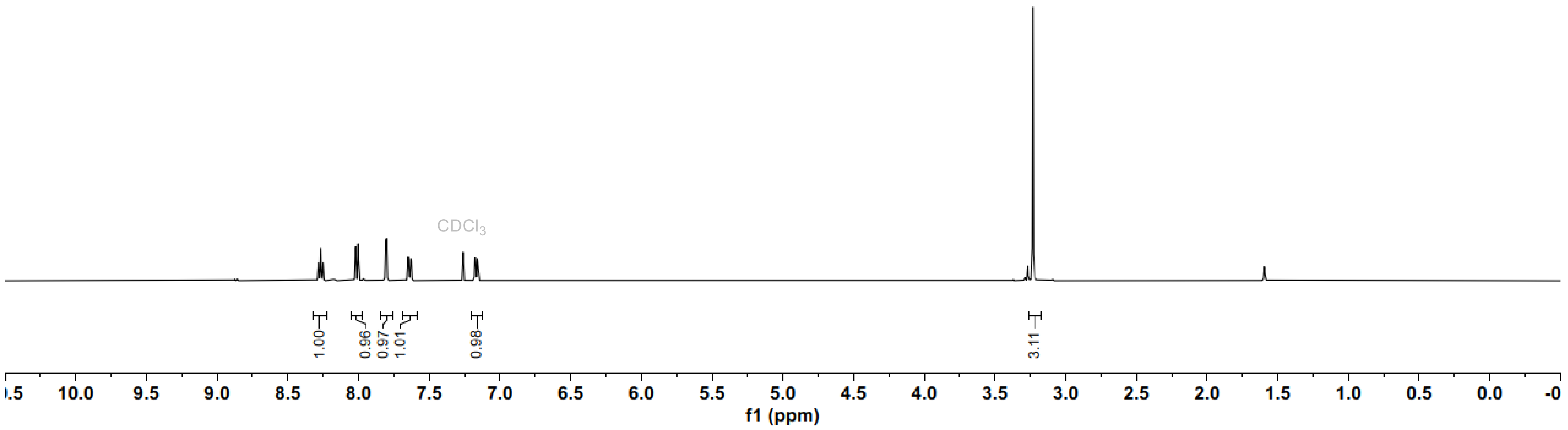

^{13}C NMR of 20b

$CDCl_3$, 126 MHz, 25 °C

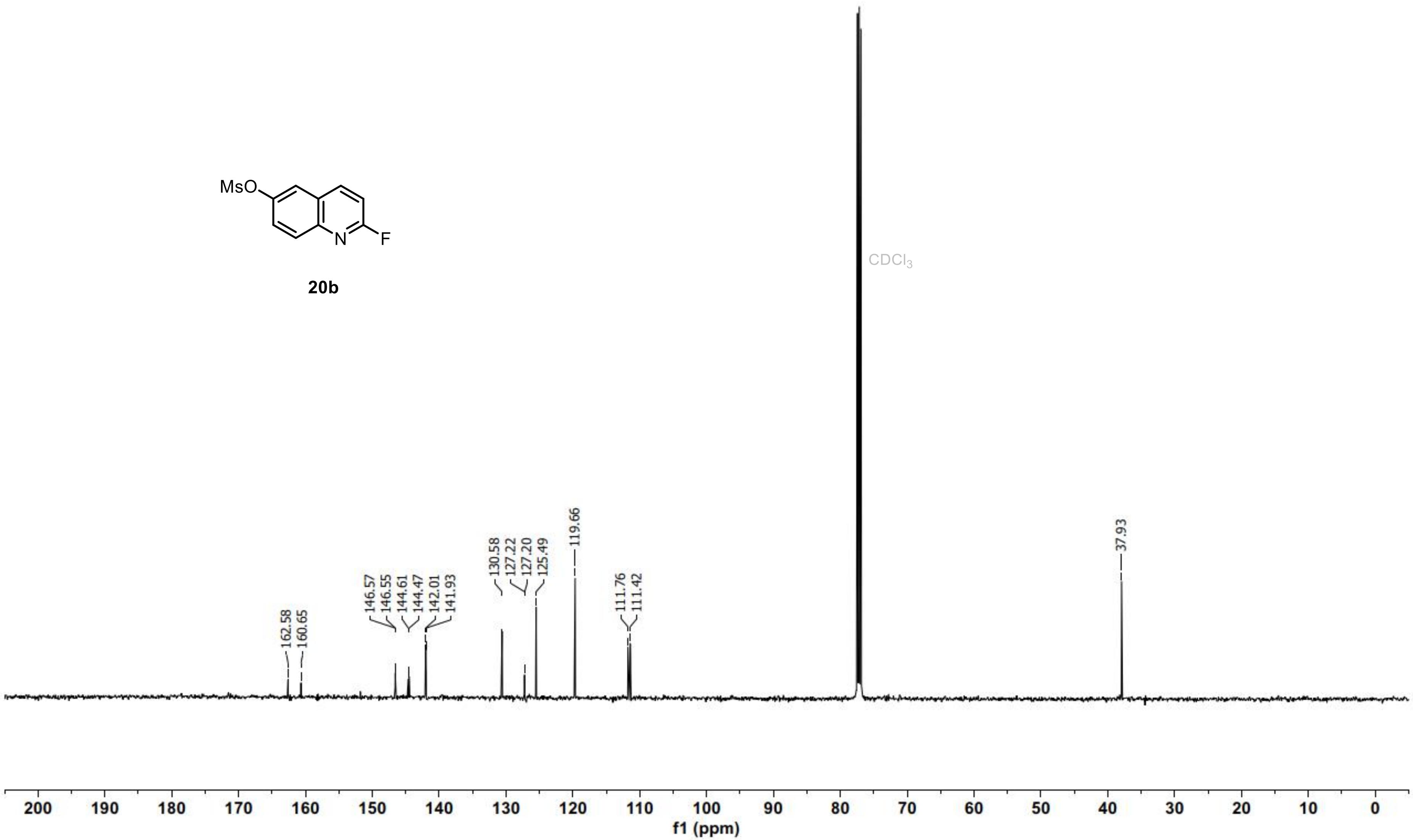

^{19}F NMR of 20b

CDCl$_3$, 471 MHz, 25 °C

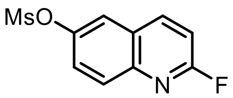

20b

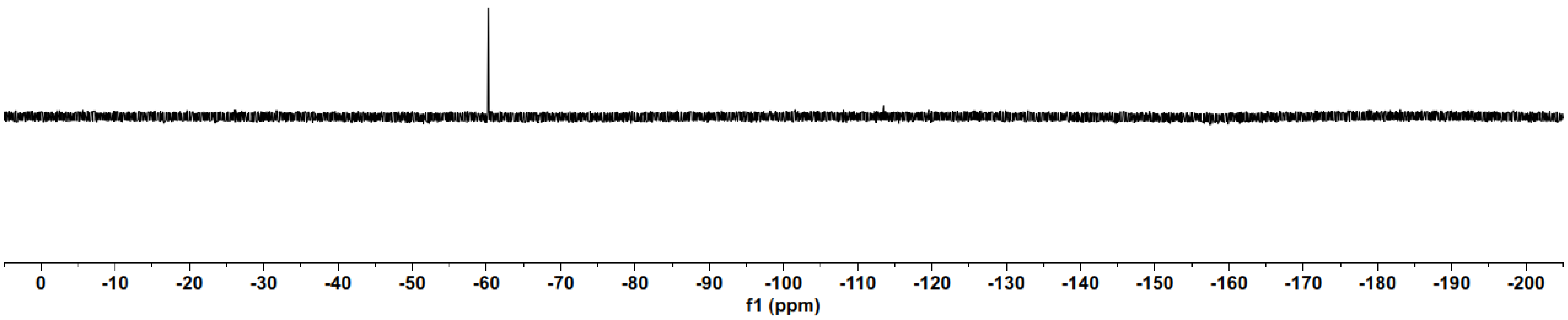

^{1}H NMR of 21

$CDCl_3$, 500 MHz, 25 °C

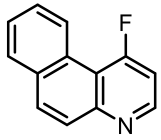

21

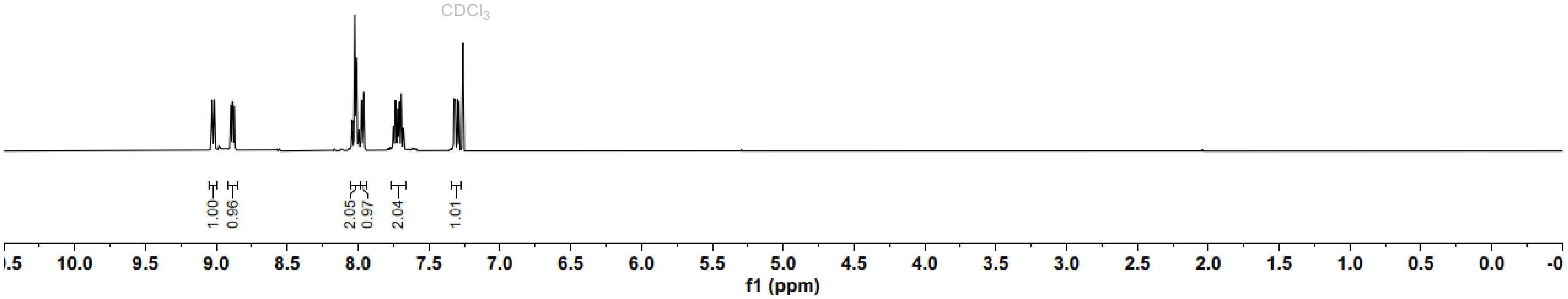

^{13}C NMR of 21

$CDCl_3$, 126 MHz, 25 °C

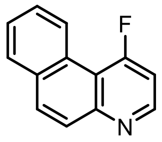

21

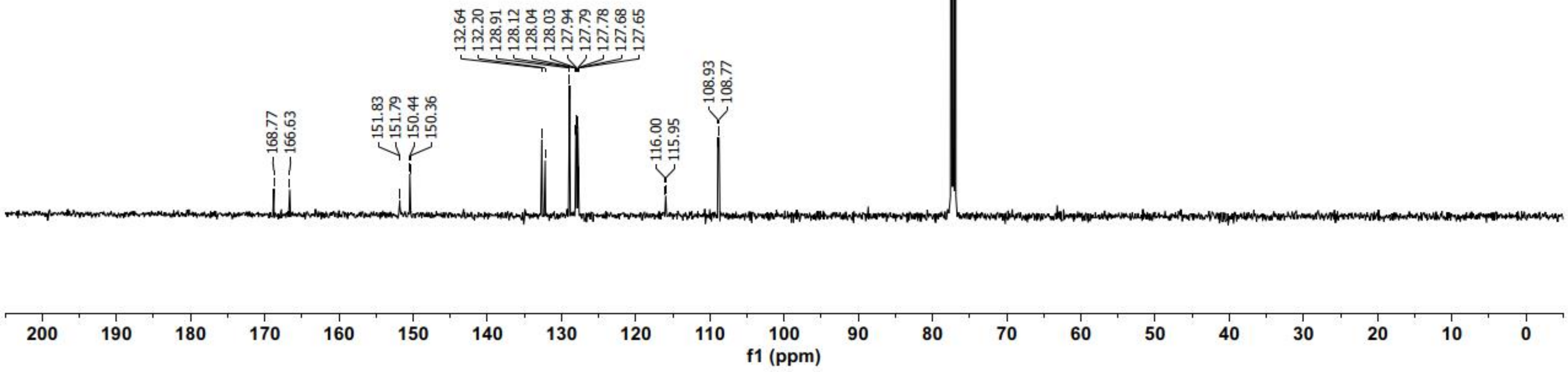

^{19}F NMR of 21

CDCl$_3$, 471 MHz, 25 °C

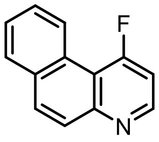

21

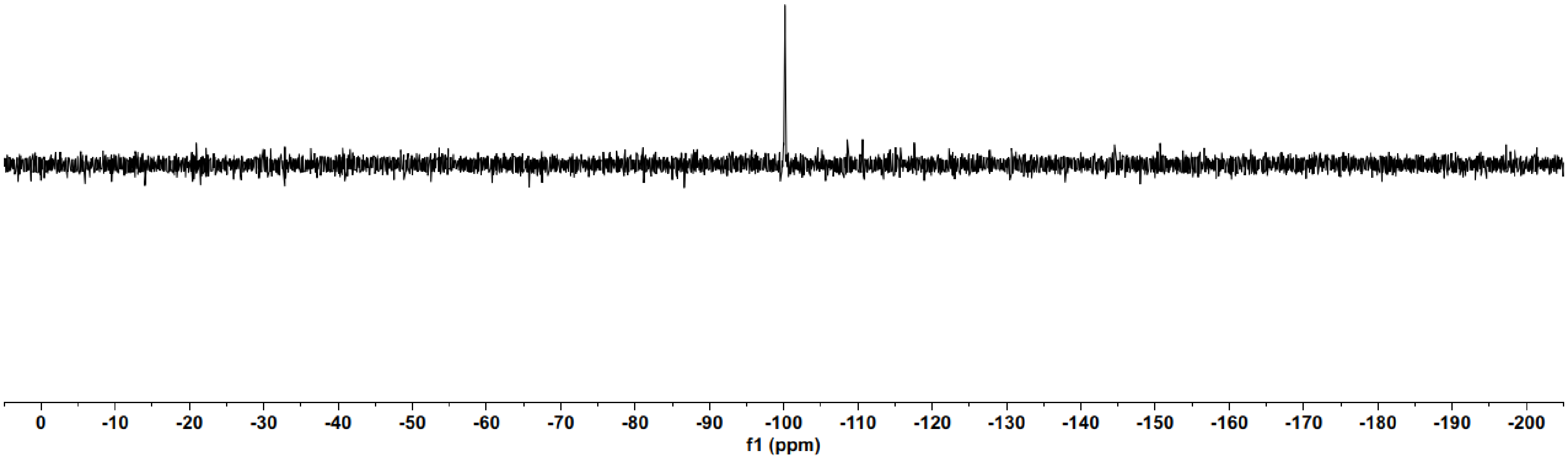

^{1}H NMR of 22

$CDCl_3$, 500 MHz, 25 °C

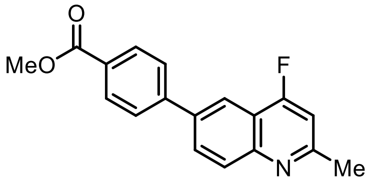

22

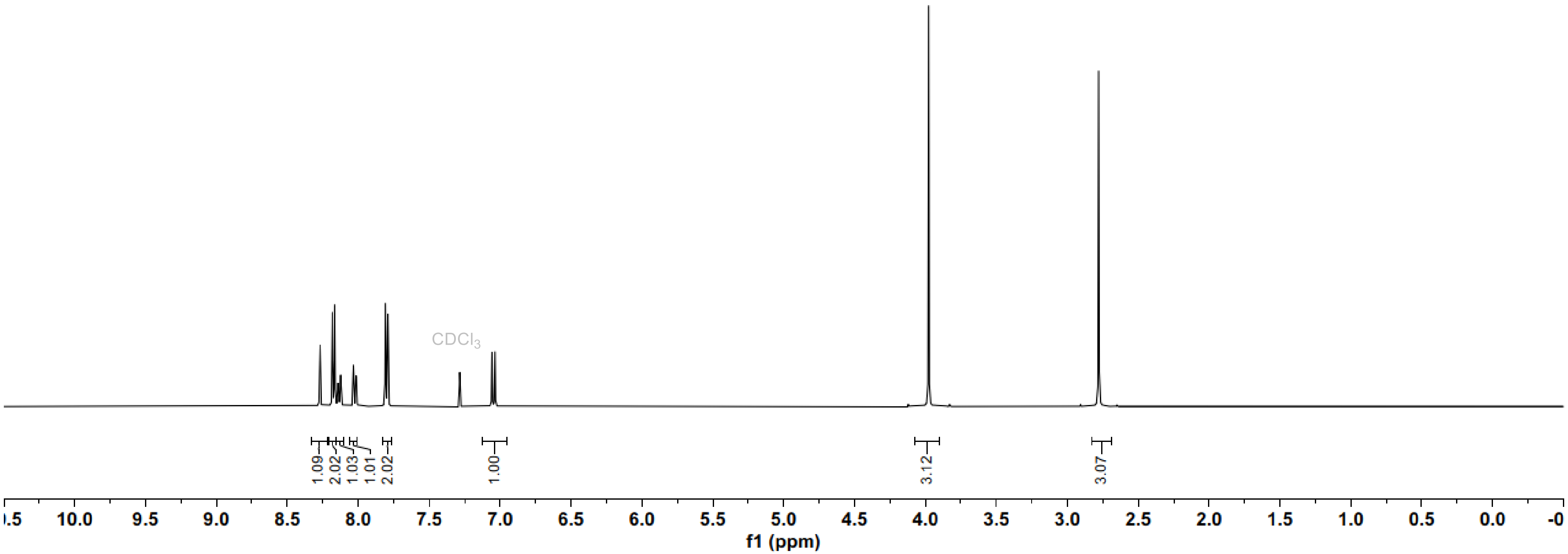

^{13}C NMR of 22

$CDCl_3$, 126 MHz, 25 °C

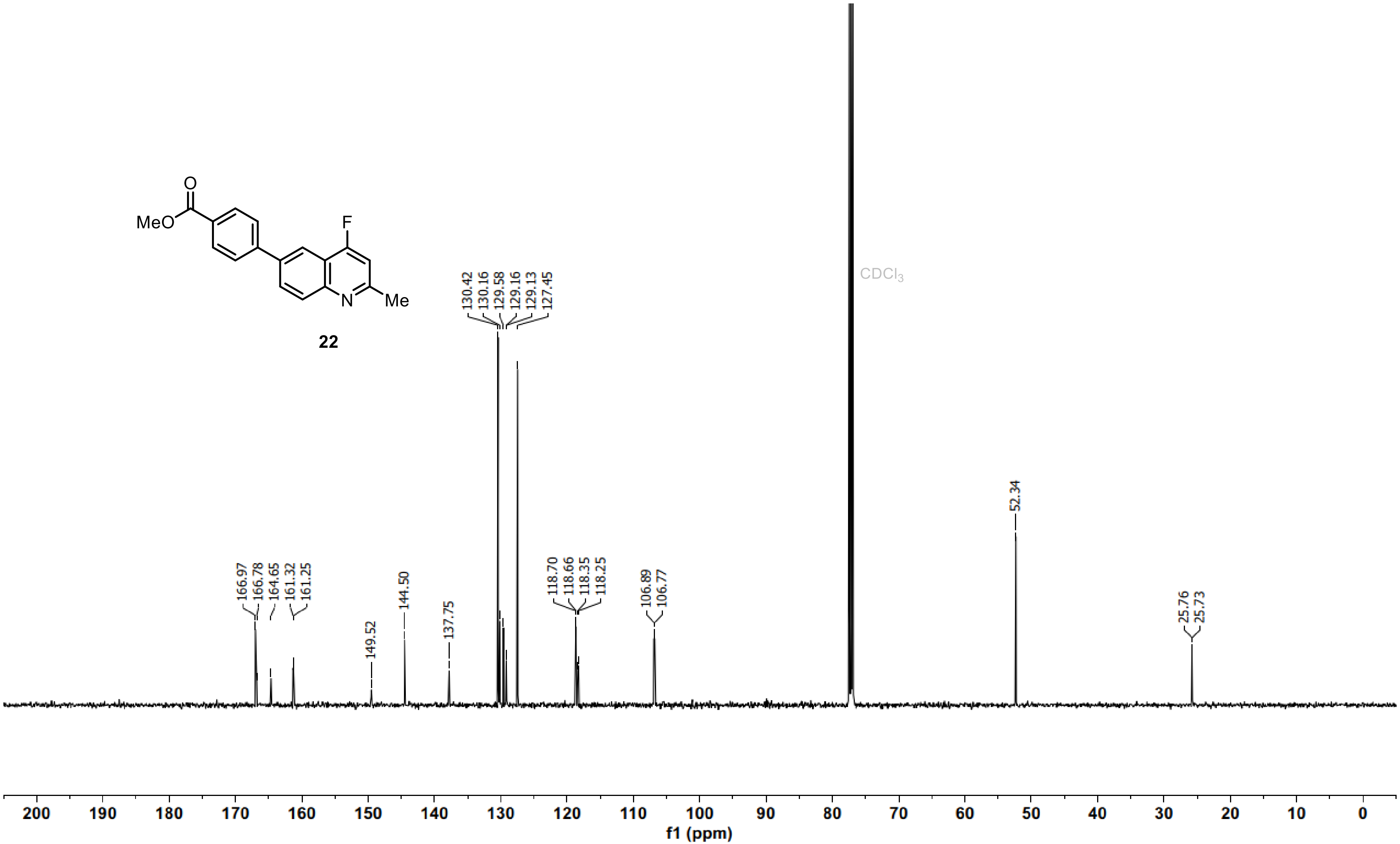

^{19}F NMR of 22

CDCl$_3$, 471 MHz, 25 °C

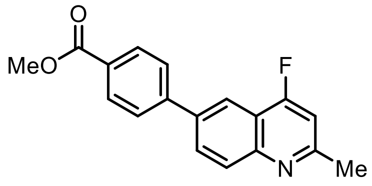

22

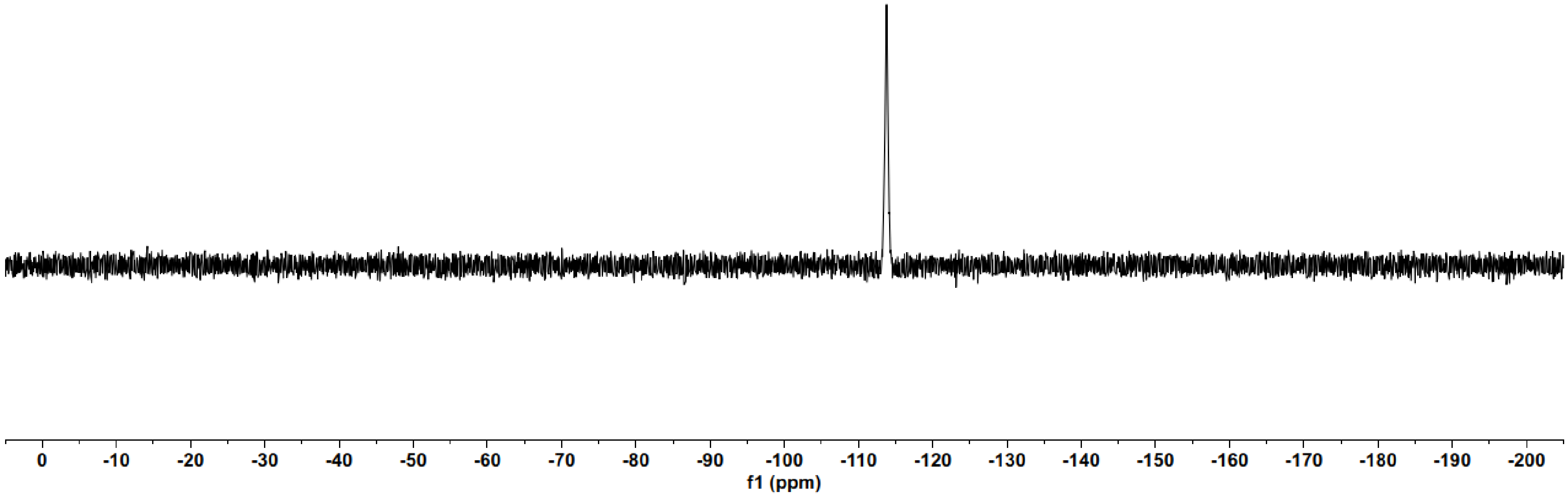

^{1}H NMR of 23a

$CDCl_3$, 500 MHz, 25 °C

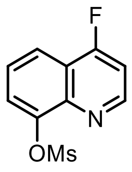

23a

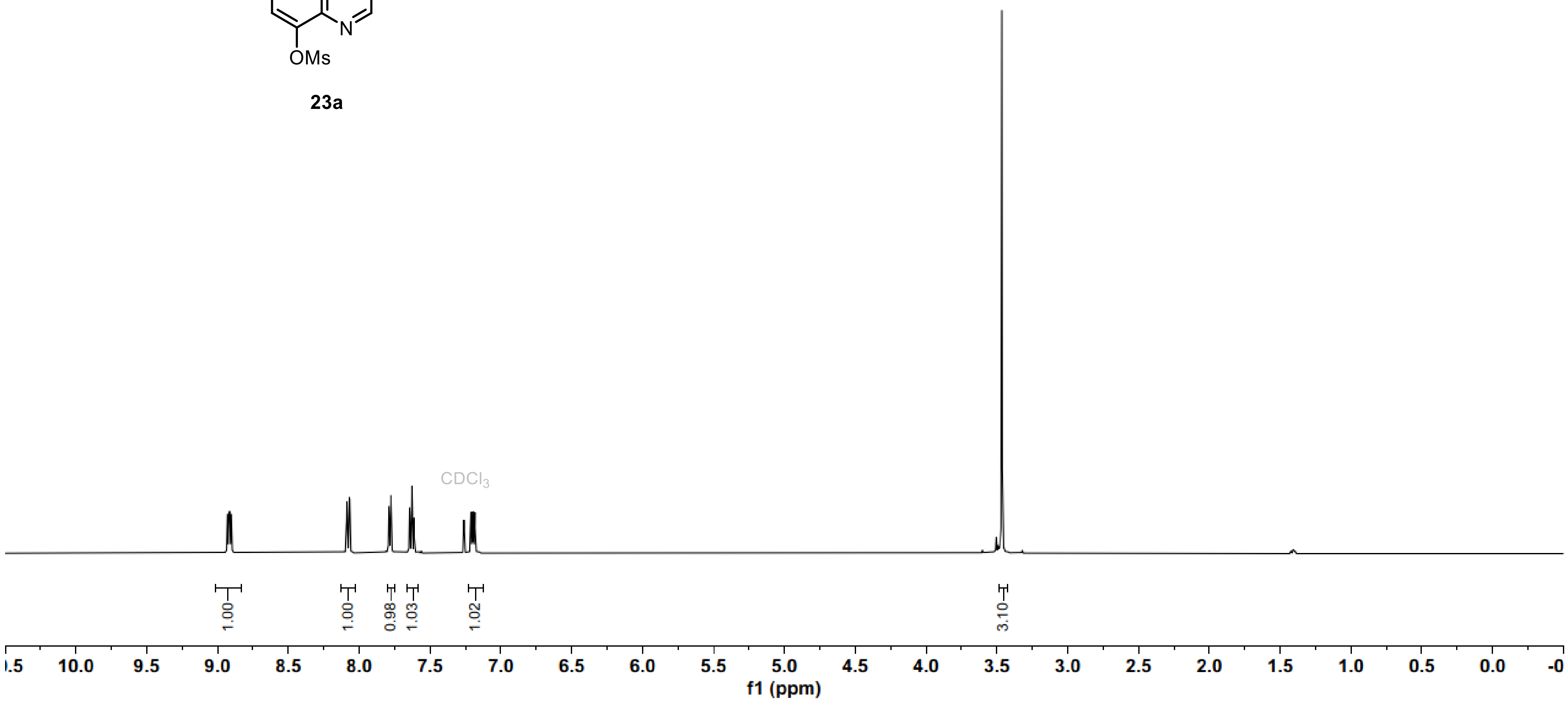

^{13}C NMR of 23a

$CDCl_3$, 126 MHz, 25 °C

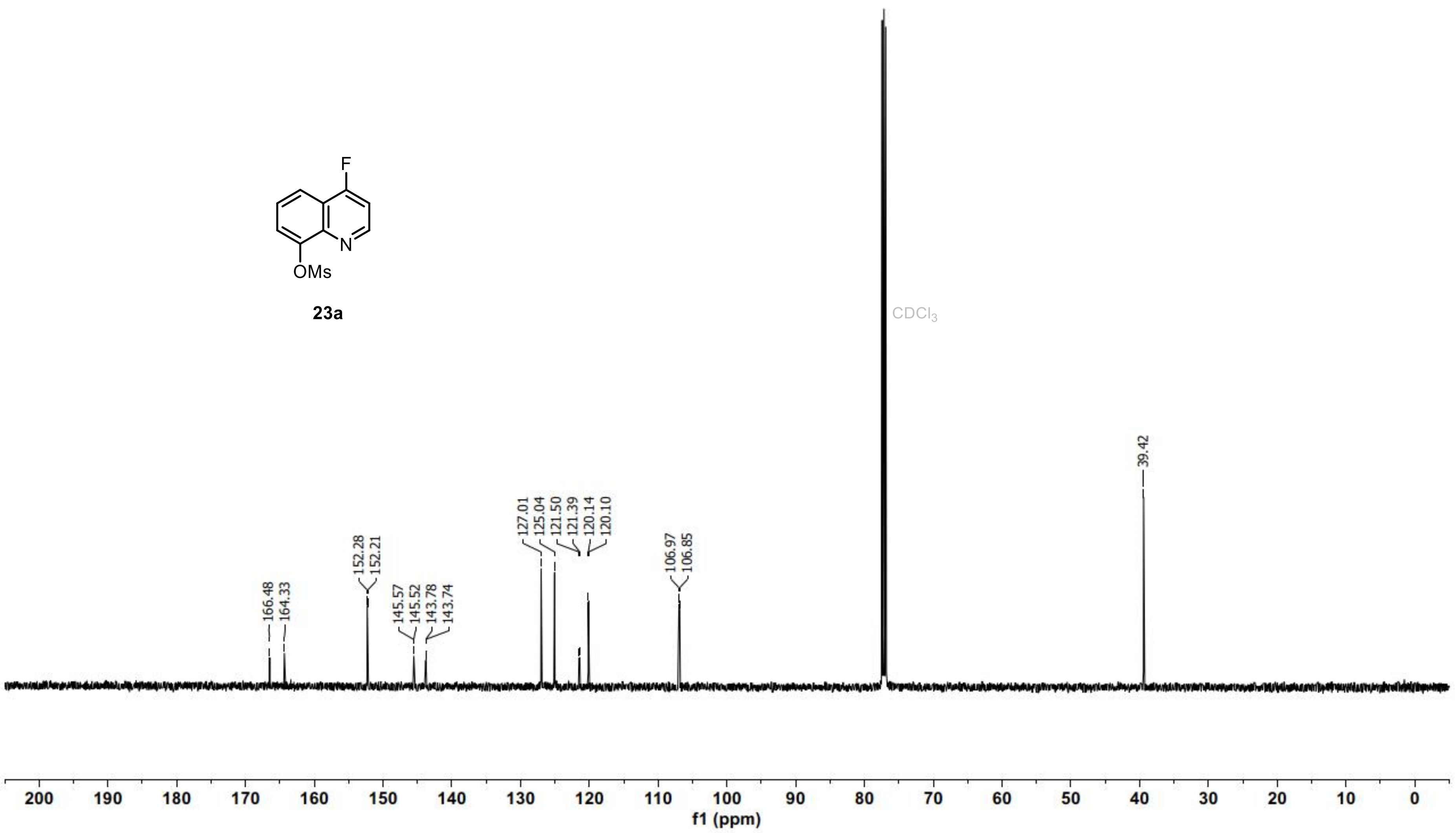

^{19}F NMR of 23a

CDCl$_3$, 471 MHz, 25 °C

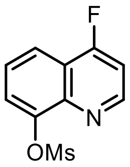

23a

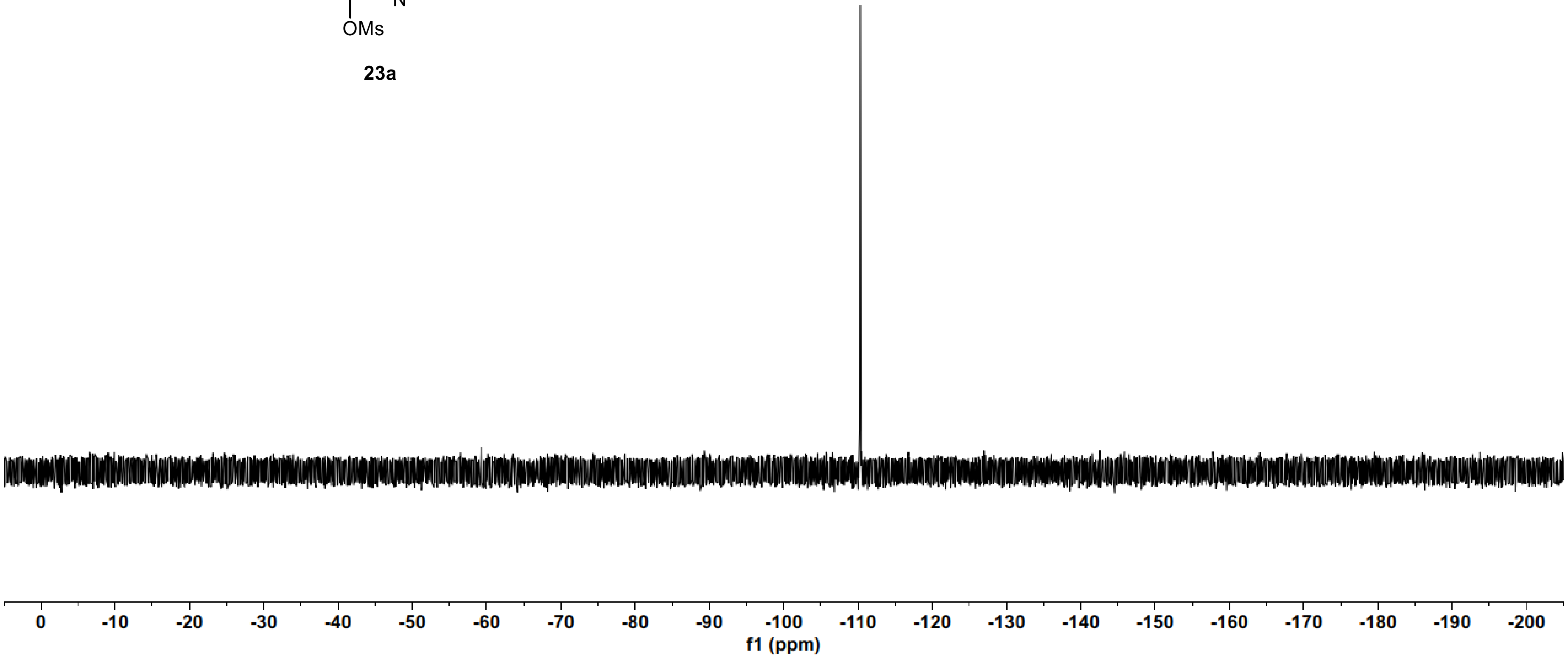

^{1}H NMR of 23b

$CDCl_3$, 500 MHz, 25 °C

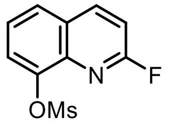

23b

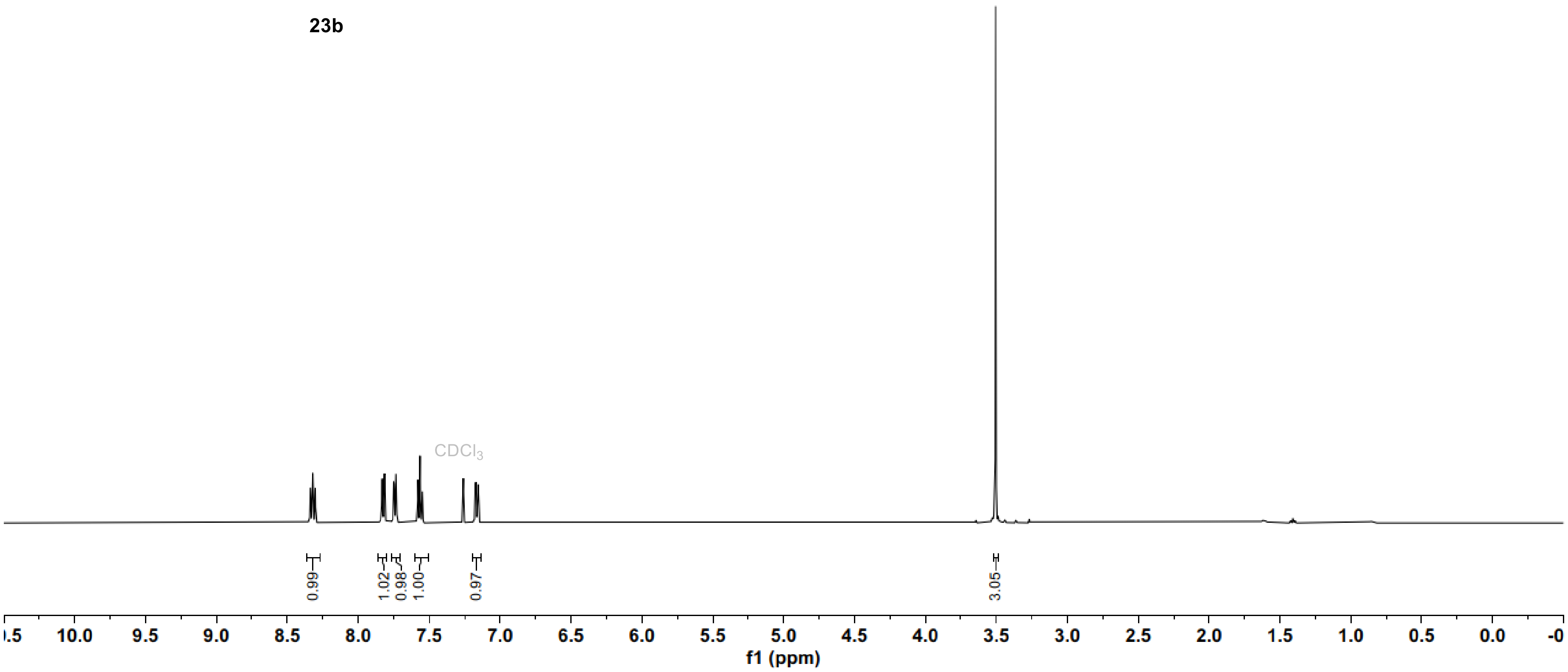

^{13}C NMR of 23b

$CDCl_3$, 126 MHz, 25 °C

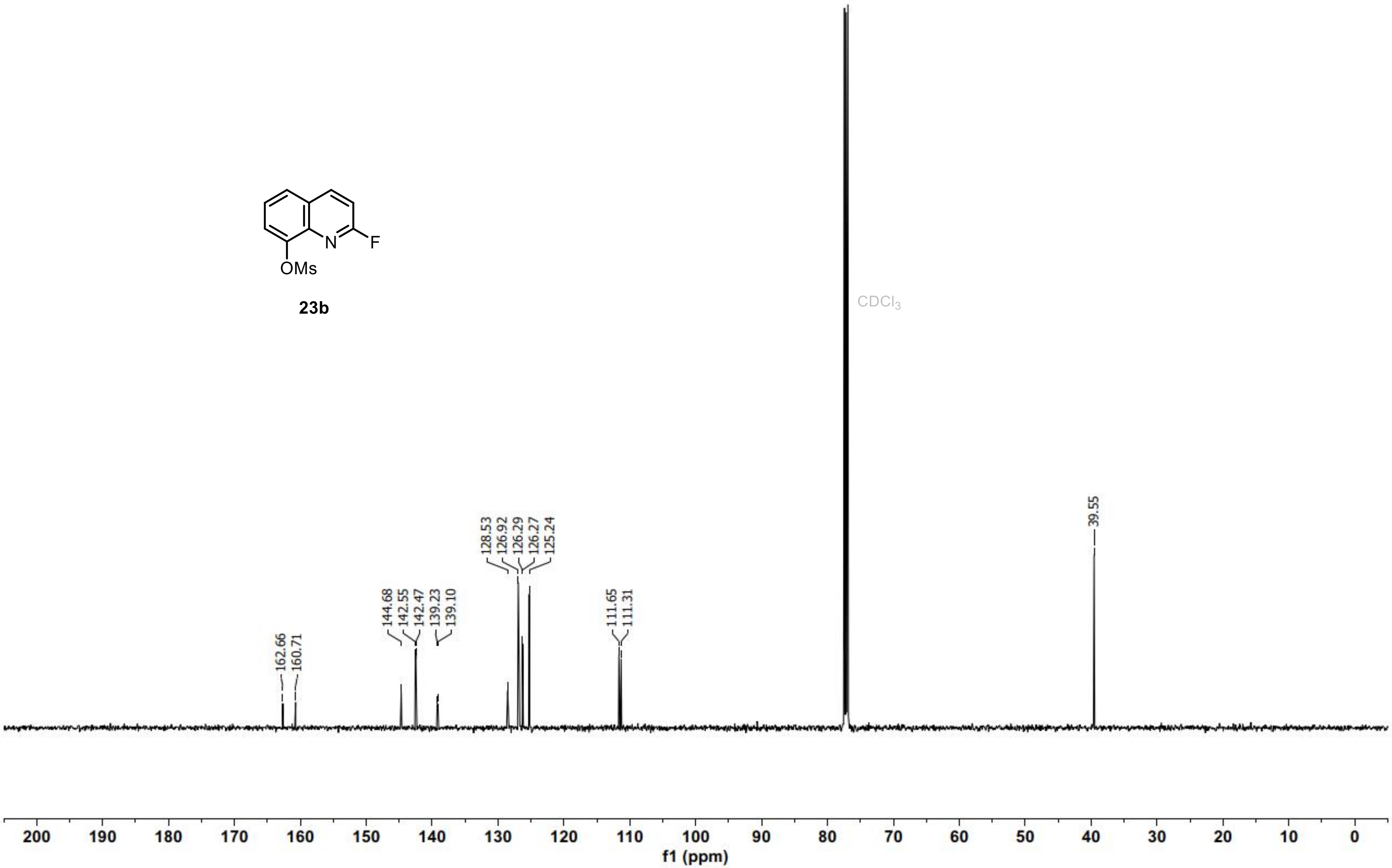

23b

^{19}F NMR of 23b

CDCl$_3$, 471 MHz, 25 °C

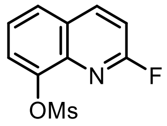

23b

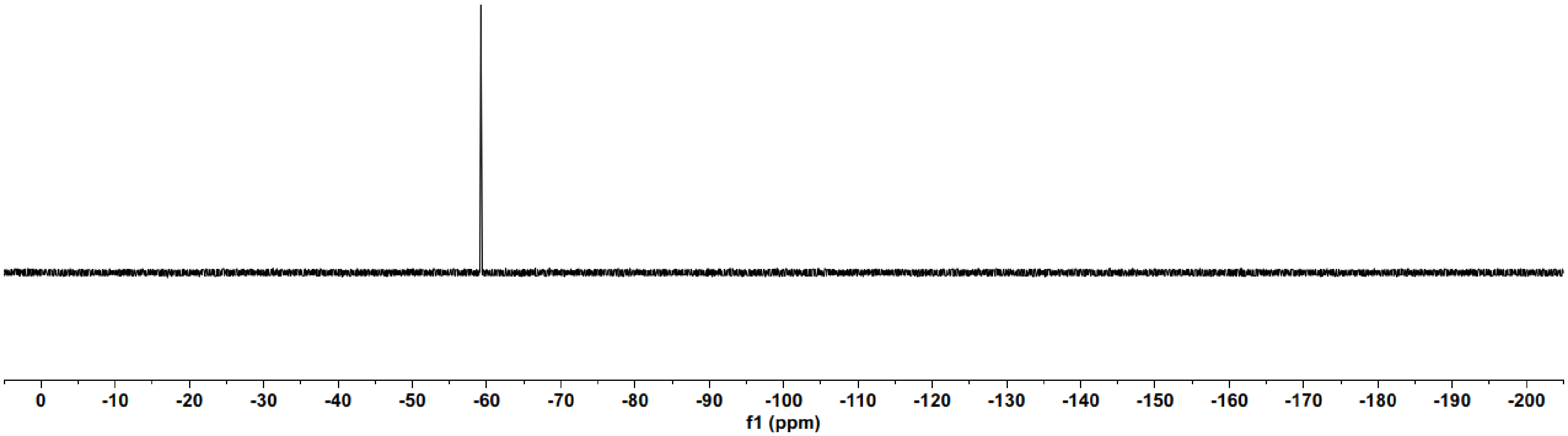

^{1}H NMR of 24a

$CDCl_3$, 500 MHz, 25 °C

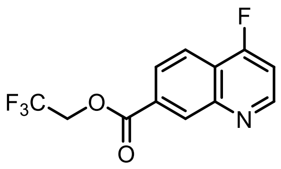

24a

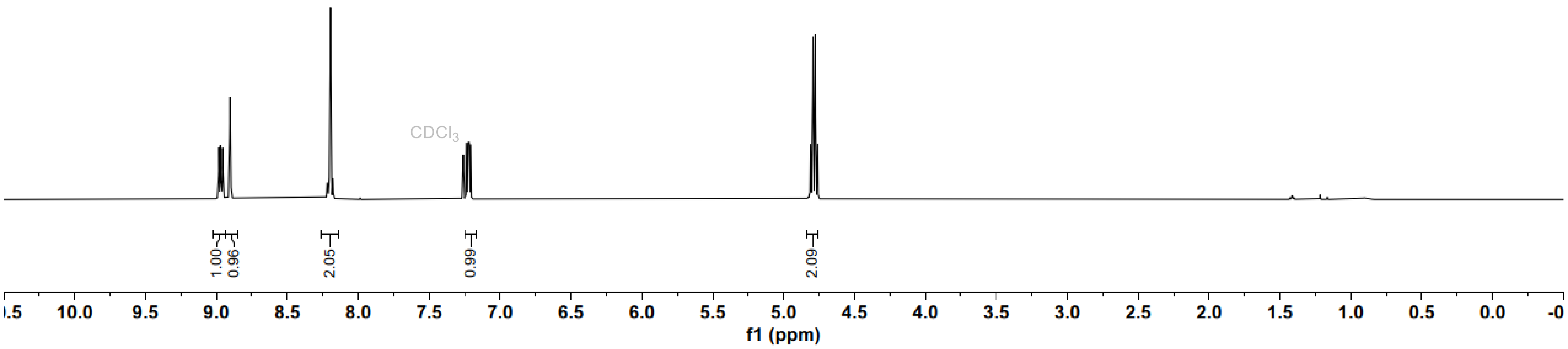

^{13}C NMR of 24a

$CDCl_3$, 126 MHz, 25 °C

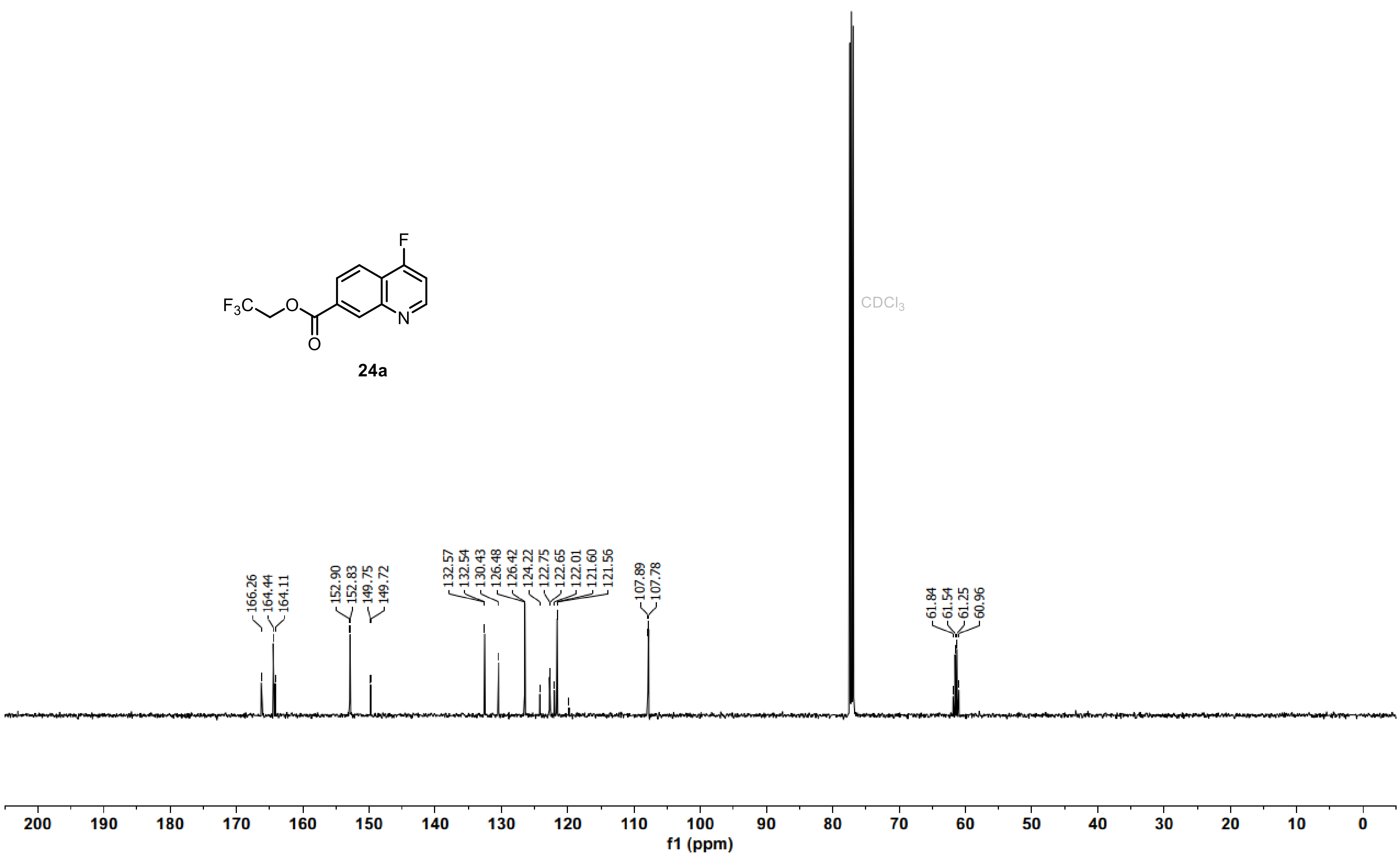

^{19}F NMR of 24a

CDCl$_3$, 471 MHz, 25 °C

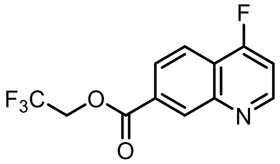

24a

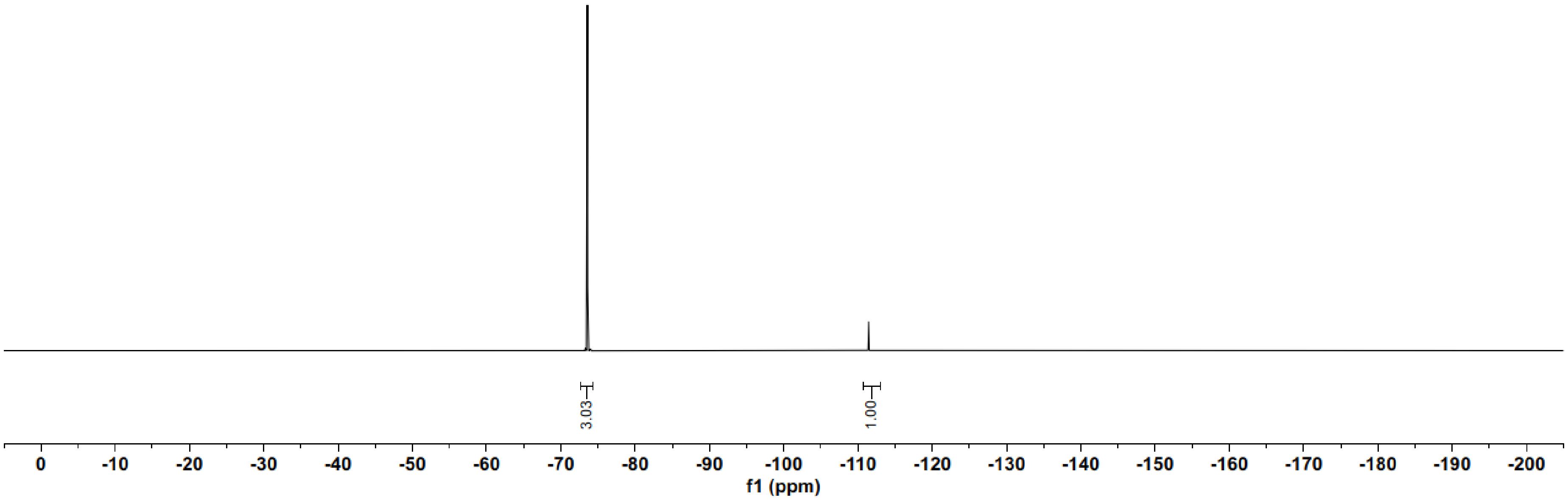

^{1}H NMR of 24b

$CDCl_3$, 500 MHz, 25 °C

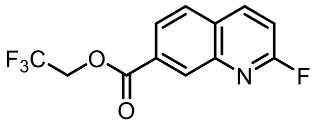

24b

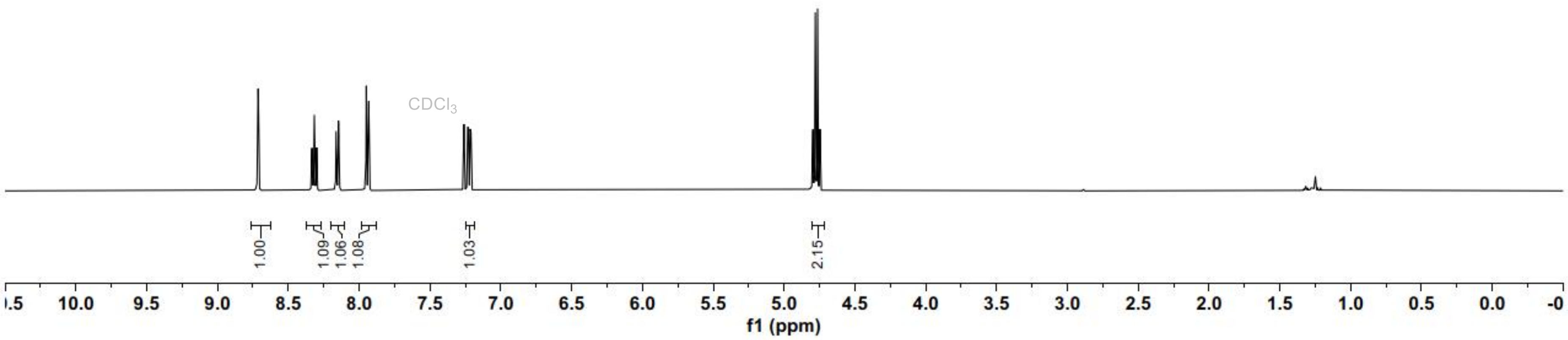

^{13}C NMR of 24b

$CDCl_3$, 126 MHz, 25 °C

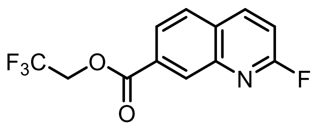

24b

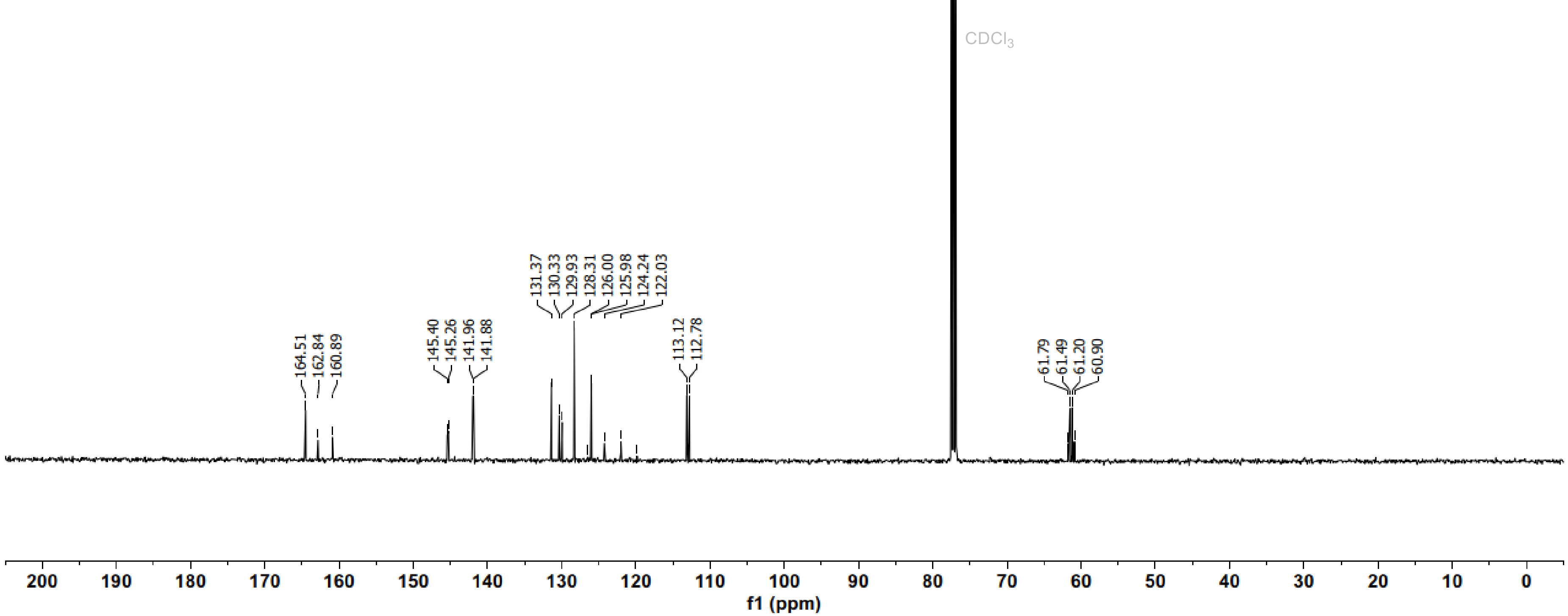

^{19}F NMR of 24b

CDCl$_3$, 471 MHz, 25 °C

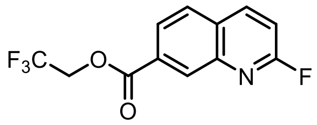

24b

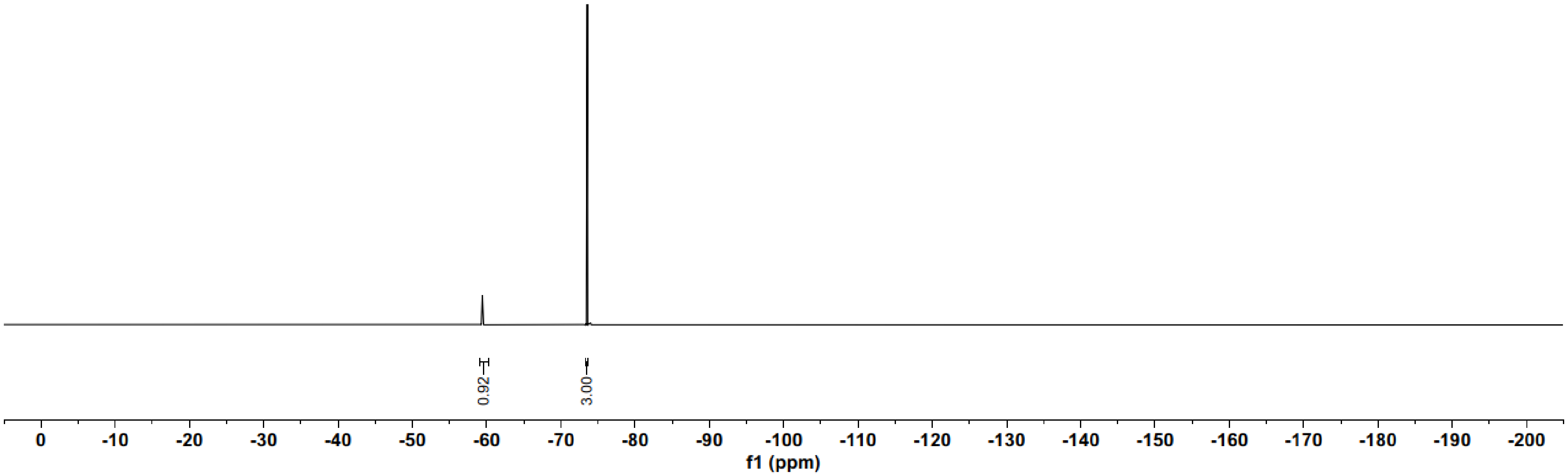

^{1}H NMR of 25

$CDCl_3$, 500 MHz, 25 °C

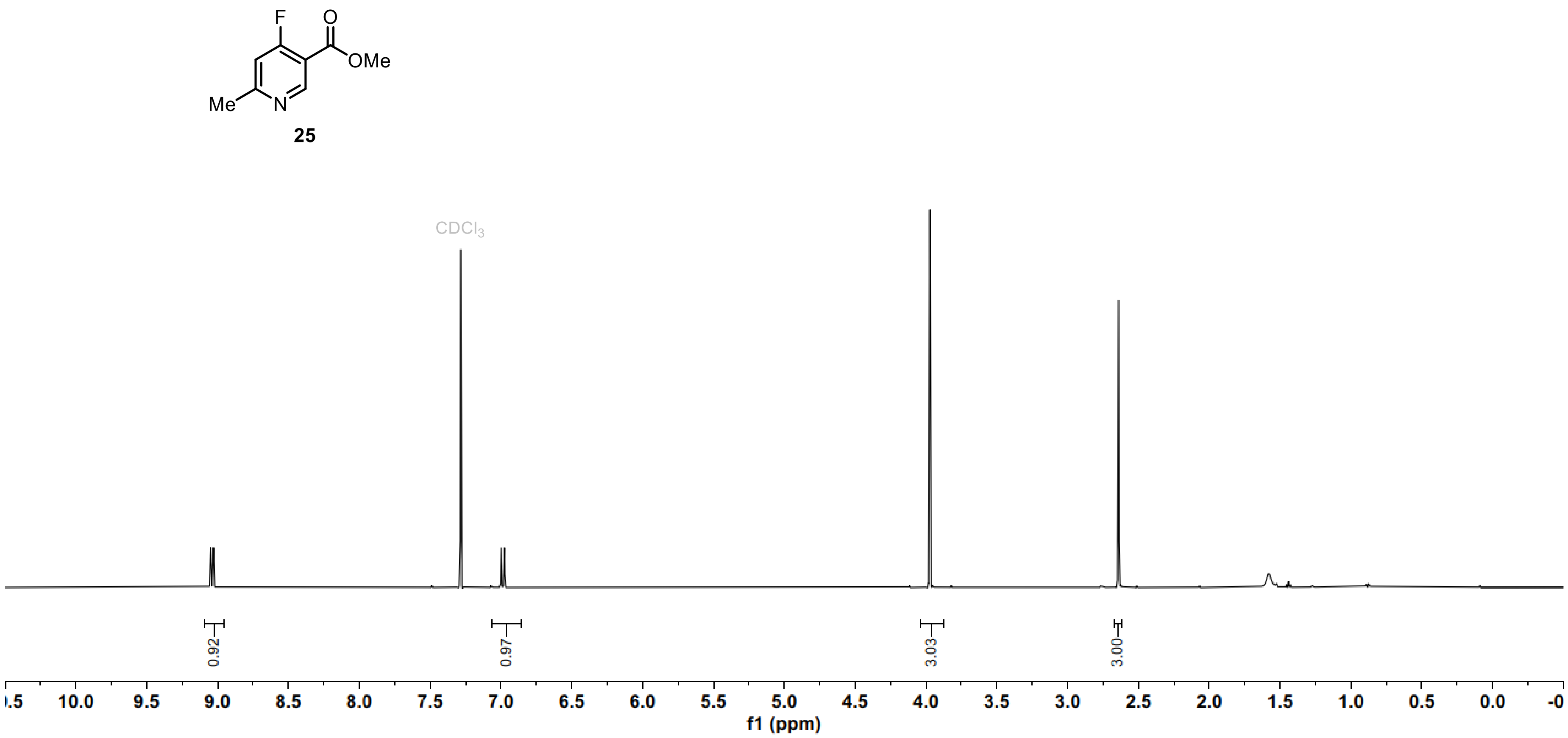

^{13}C NMR of 25

$CDCl_3$, 126 MHz, 25 °C

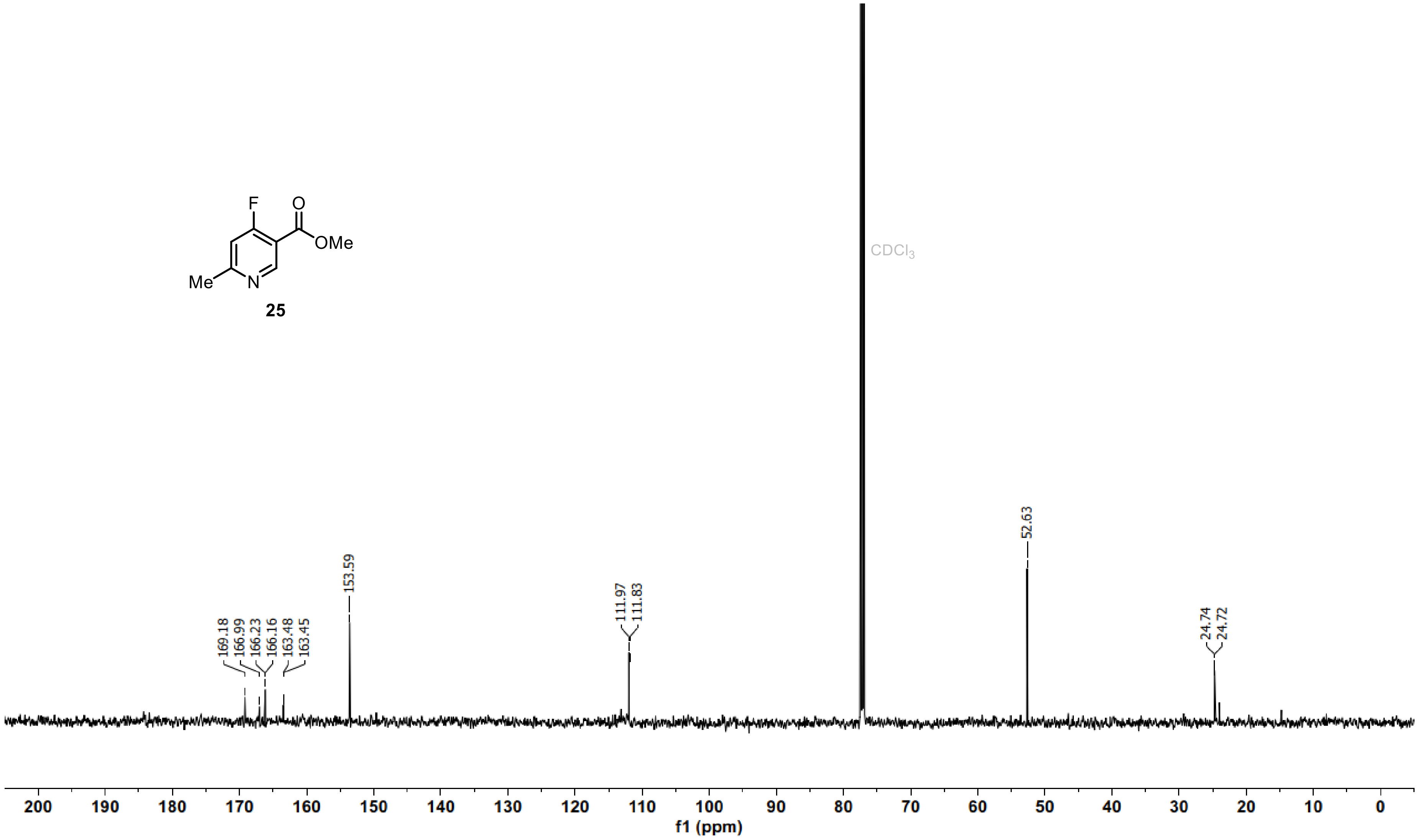

^{19}F NMR of 25

CDCl$_3$, 471 MHz, 25 °C

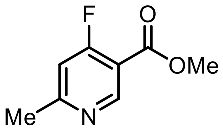

25

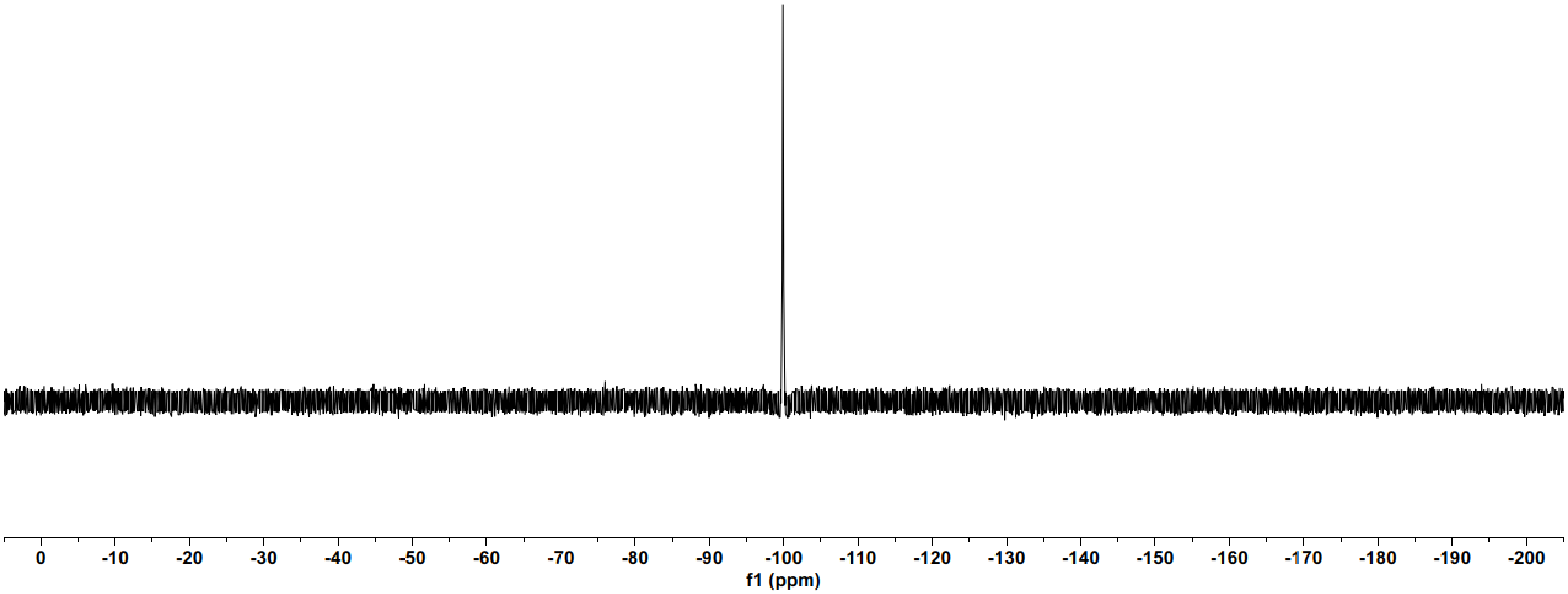

^{1}H NMR of 26a

$CDCl_3$, 500 MHz, 25 °C

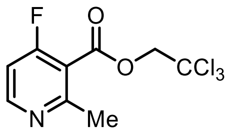

26a

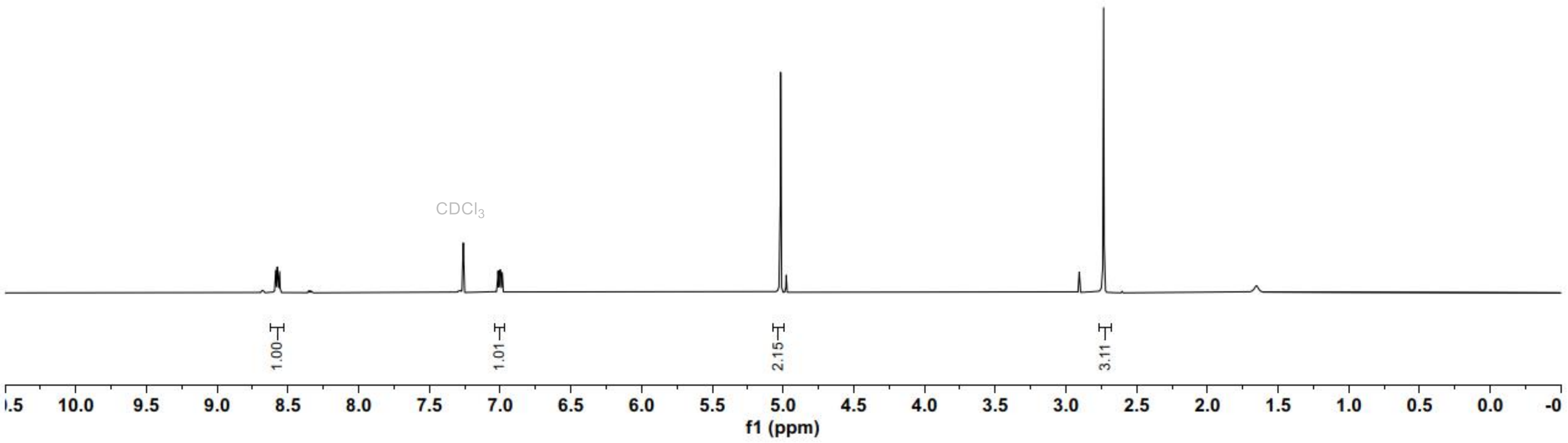

^{13}C NMR of 26a

CDCl$_3$, 126 MHz, 25 °C

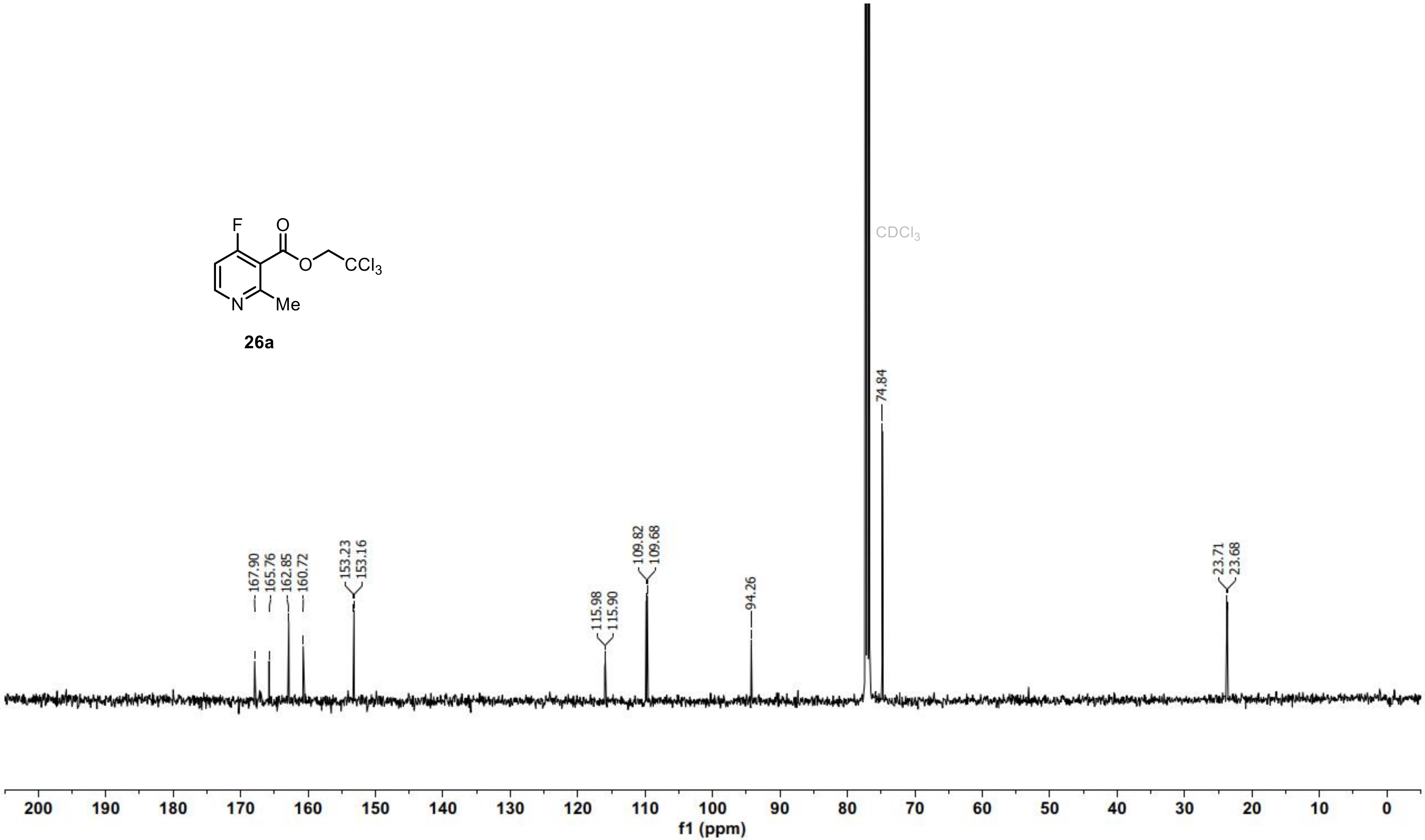

^{19}F NMR of 26a

CDCl$_3$, 471 MHz, 25 °C

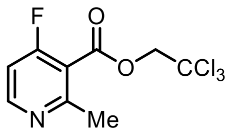

26a

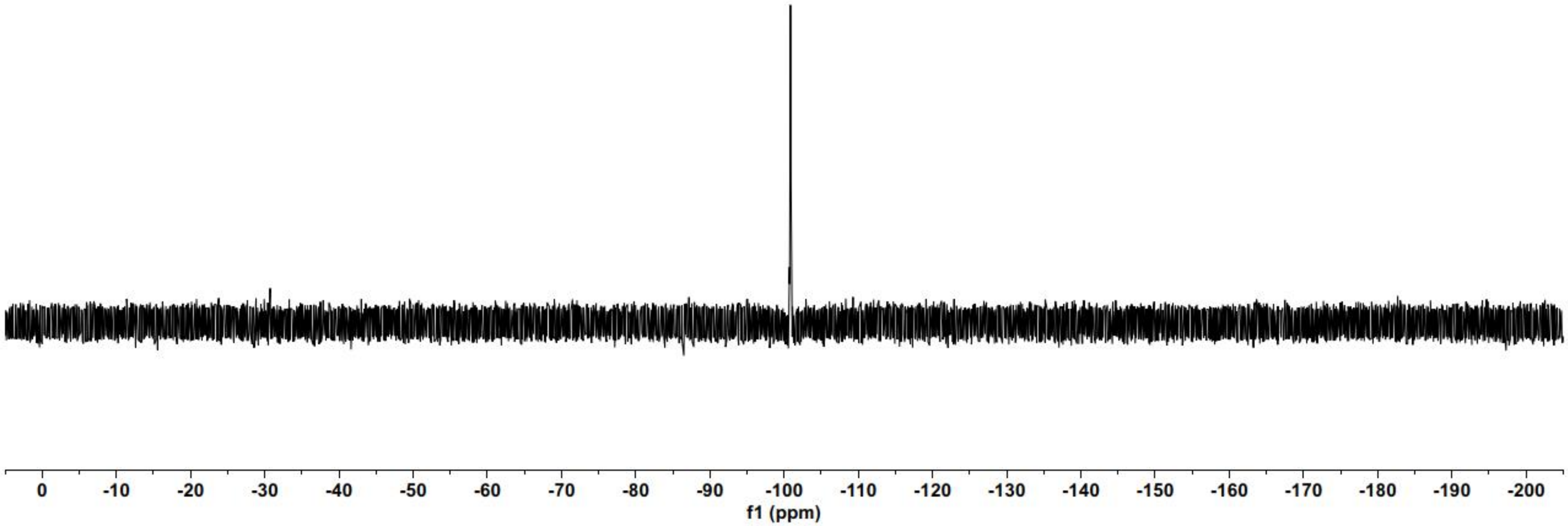

^{1}H NMR of 26b

$CDCl_3$, 500 MHz, 25 °C

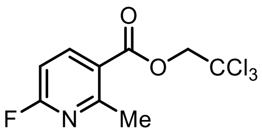

26b

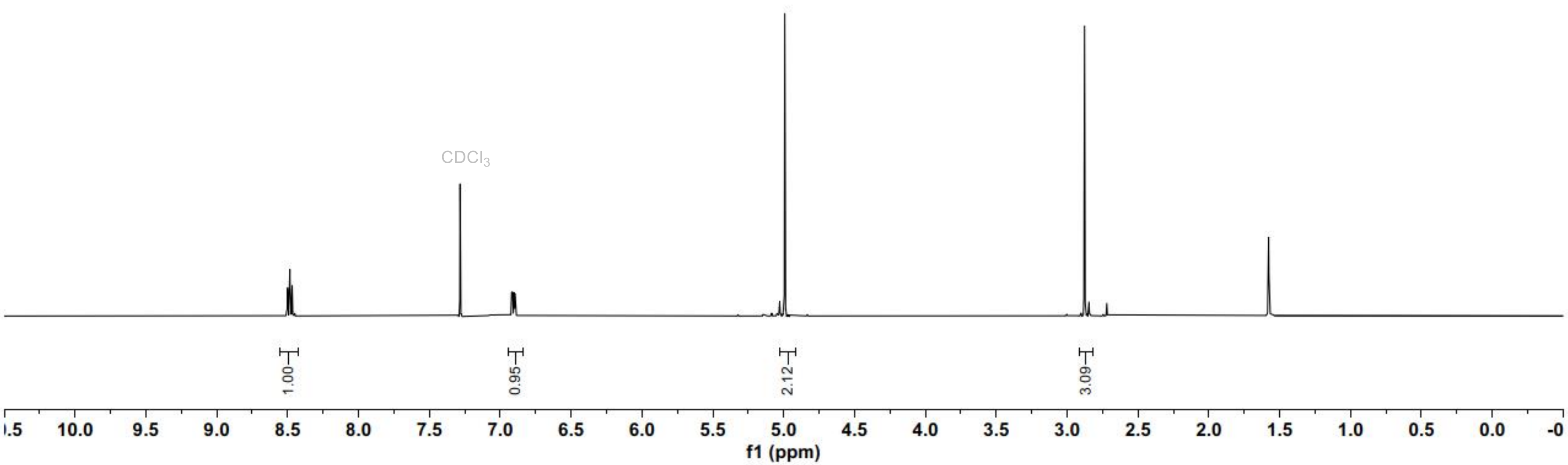

^{13}C NMR of 26b

$CDCl_3$, 126 MHz, 25 °C

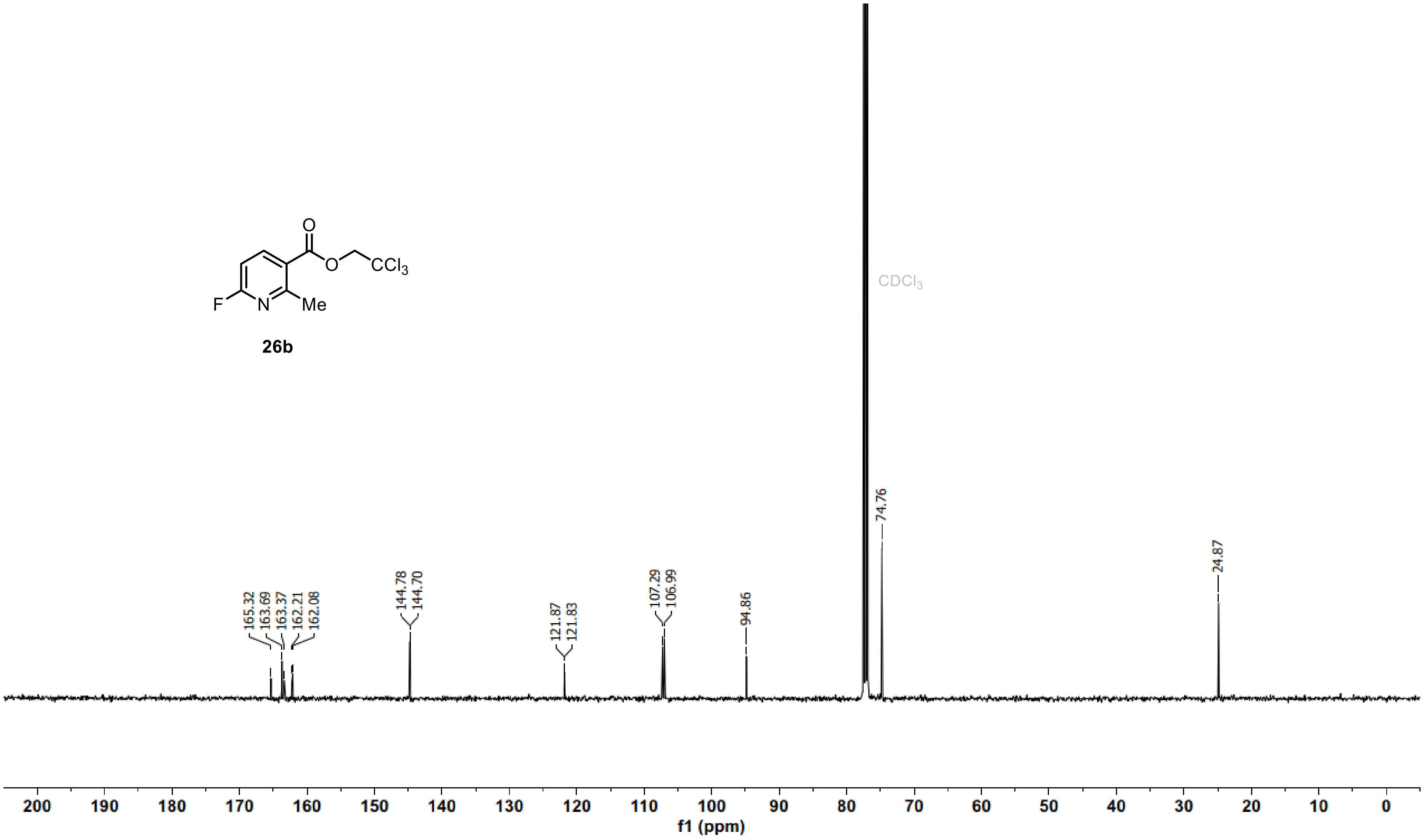

^{19}F NMR of 26b

$CDCl_3$, 471 MHz, 25 °C

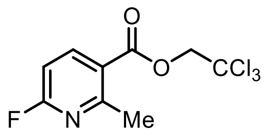

26b

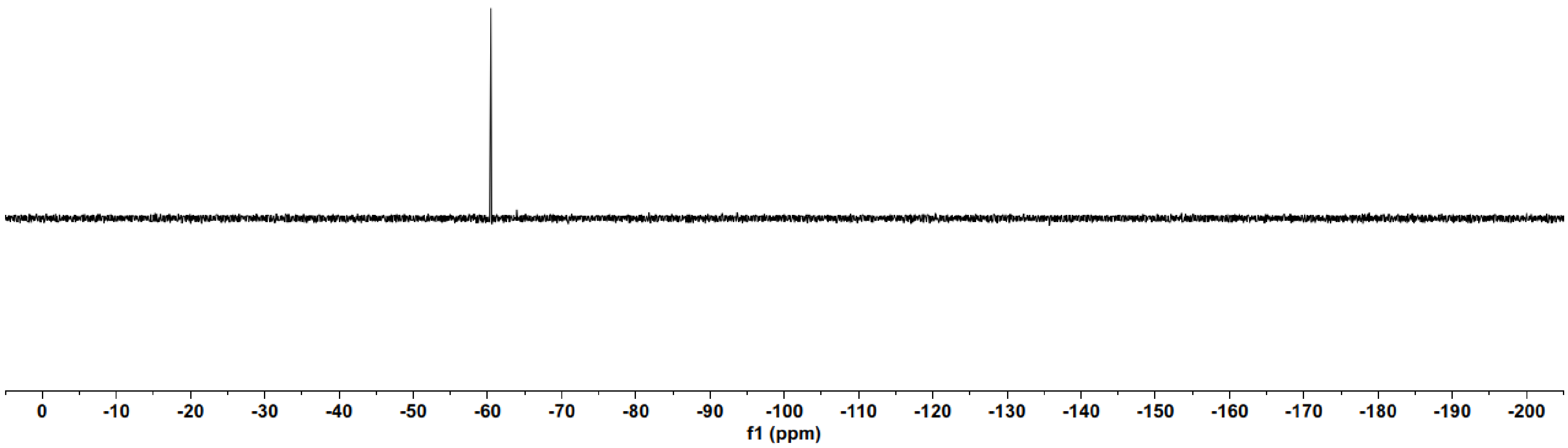

^{1}H NMR of 27

$CDCl_3$, 500 MHz, 25 °C

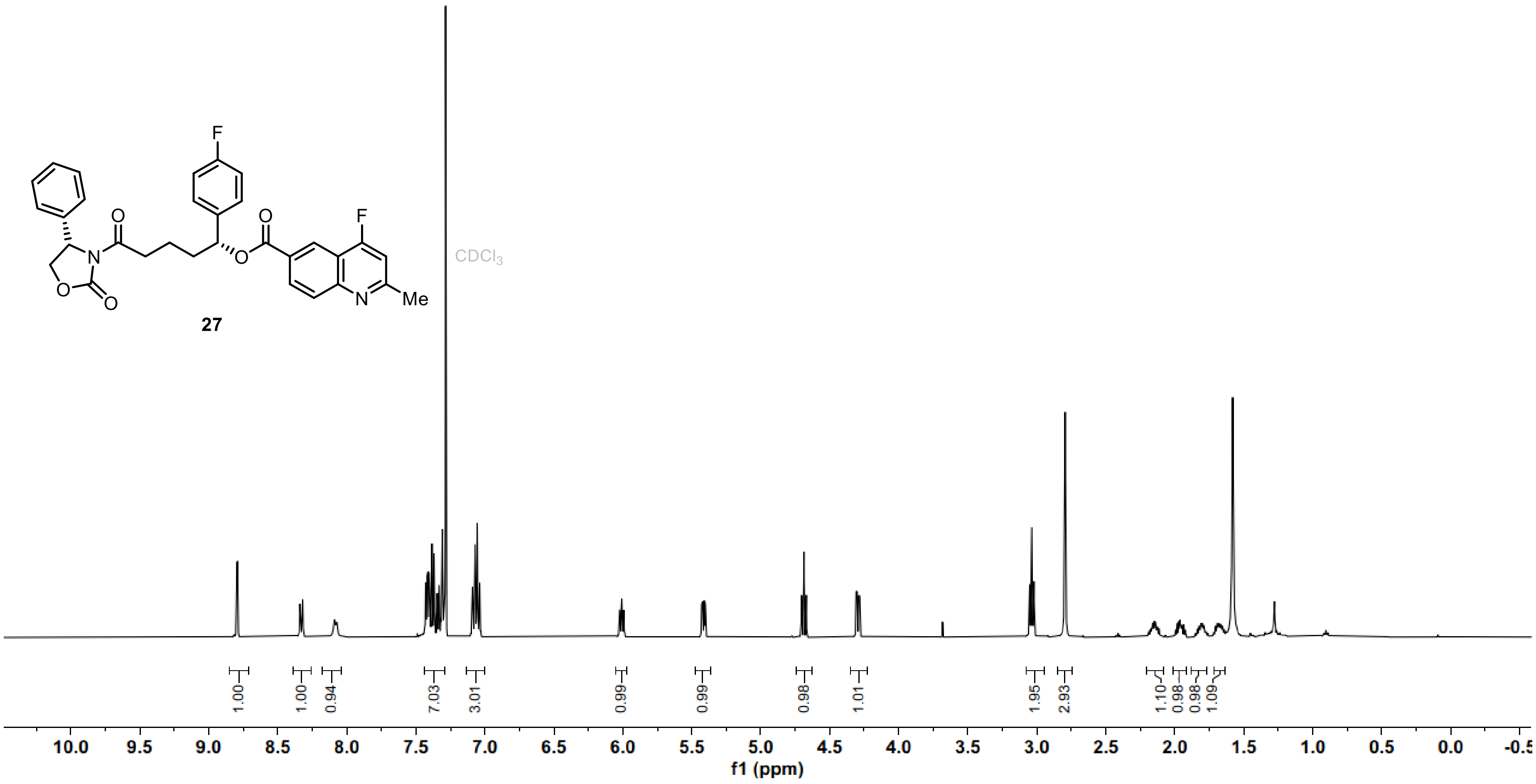

^{13}C NMR of 27

$CDCl_3$, 126 MHz, 25 °C

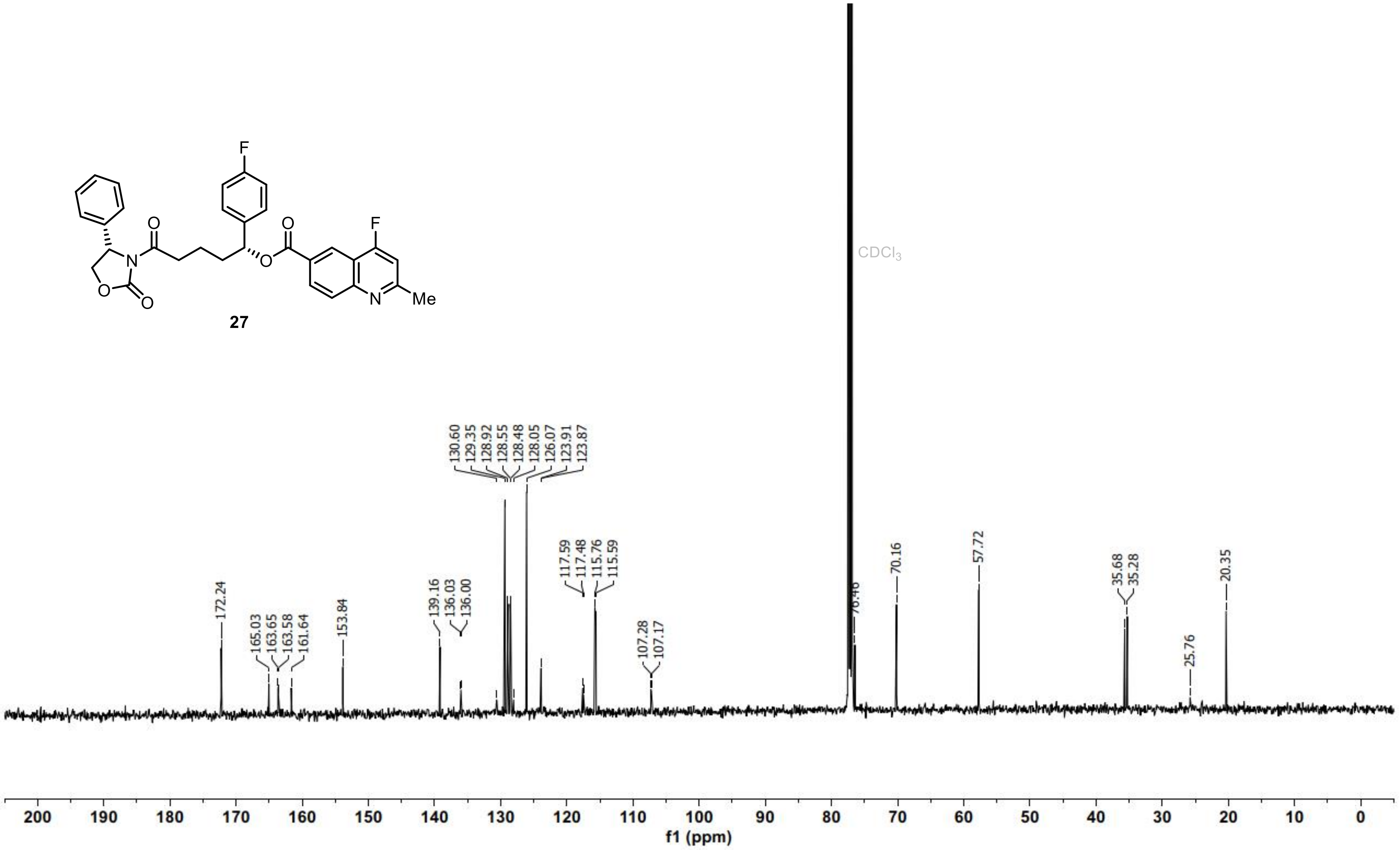

^{19}F NMR of 27

CDCl$_3$, 471 MHz, 25 °C

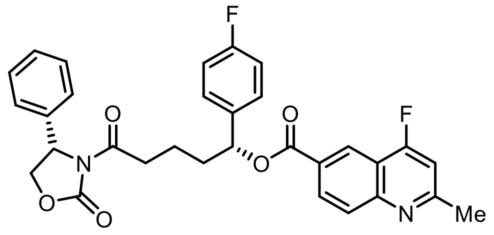

27

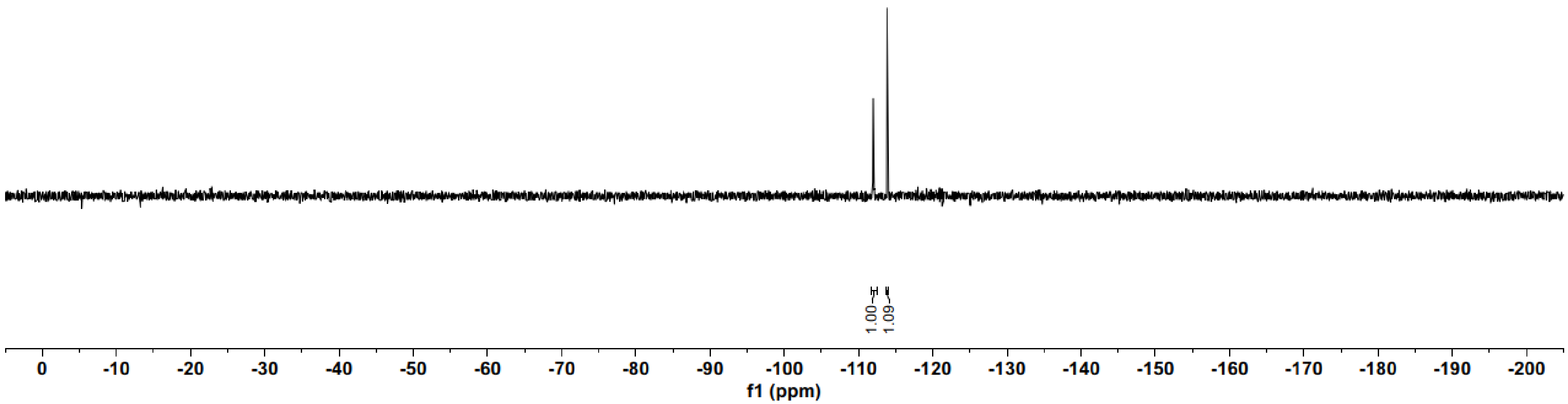

^{1}H NMR of 28

$CDCl_3$, 500 MHz, 25 °C

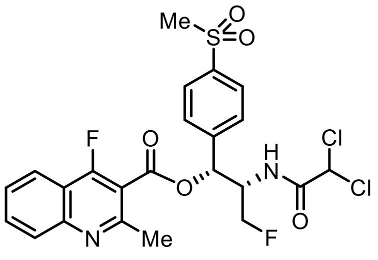

28

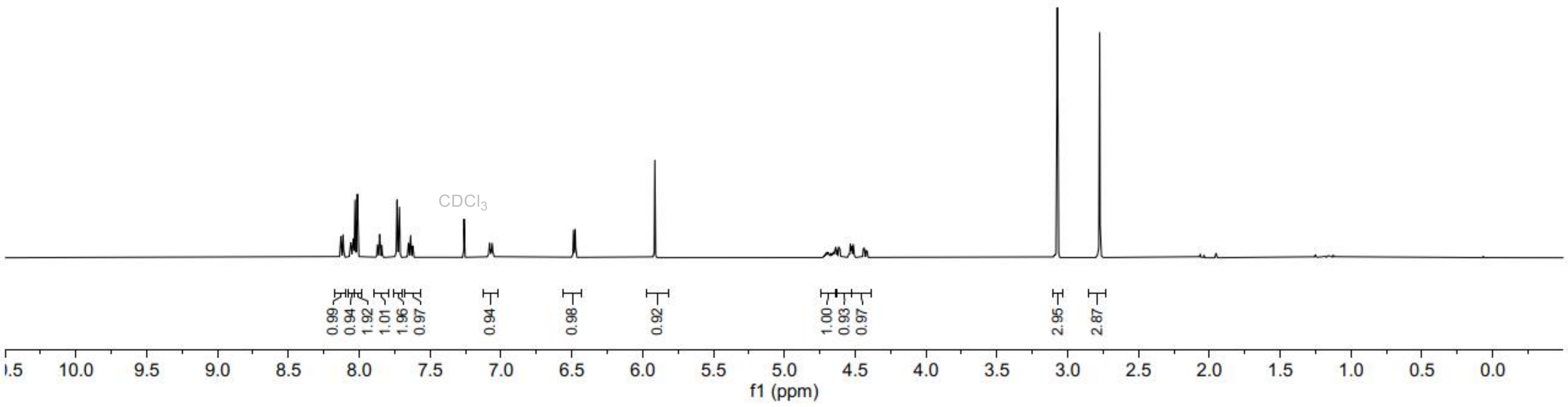

^{13}C NMR of 28

$CDCl_3$, 126 MHz, 25 °C

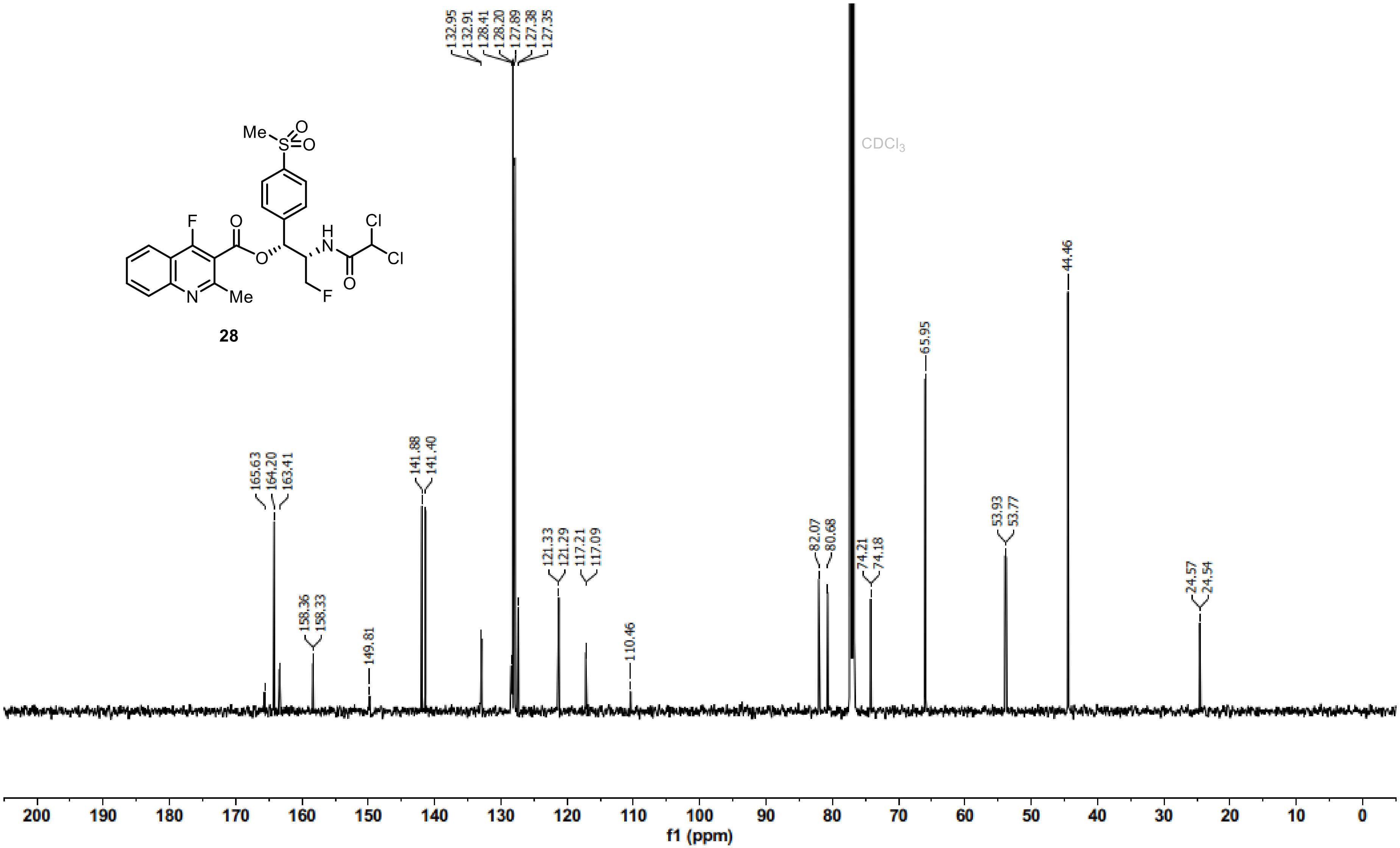

28

^{19}F NMR of 28

CDCl₃, 471 MHz, 25 °C

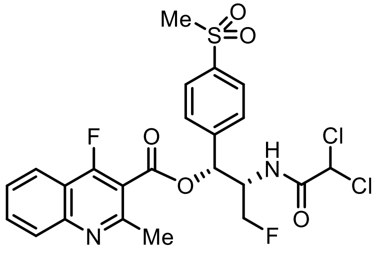

28

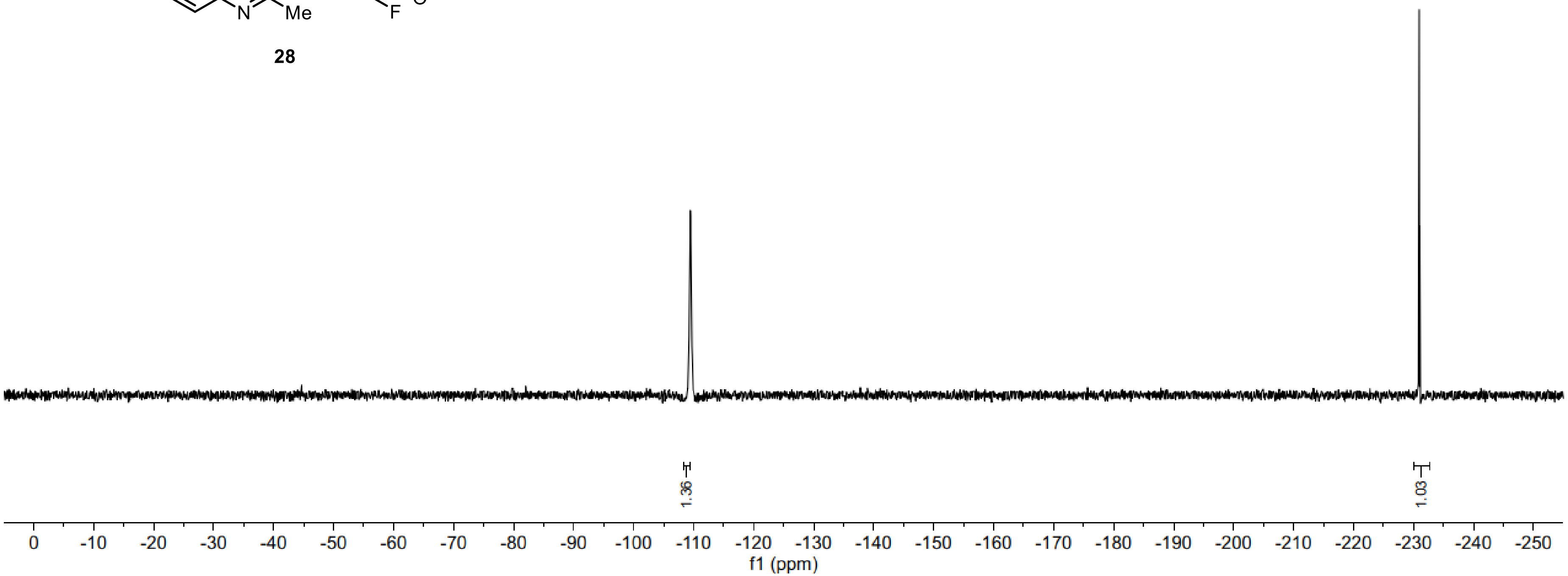

^{1}H NMR of 29

CDCl$_3$, 500 MHz, 25 °C

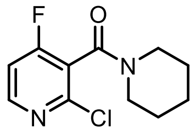

29

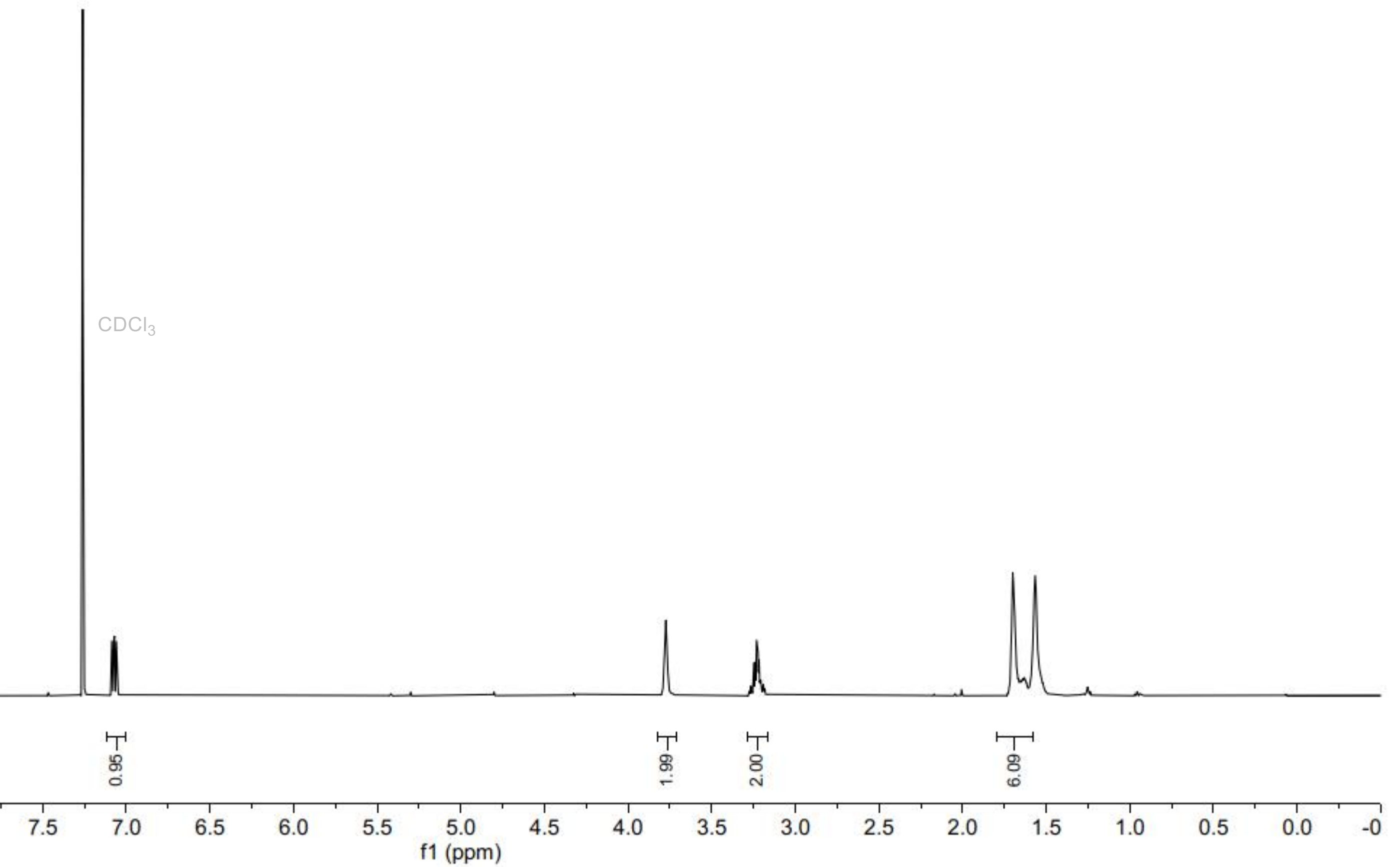

^{13}C NMR of 29

$CDCl_3$, 151 MHz, 25 °C

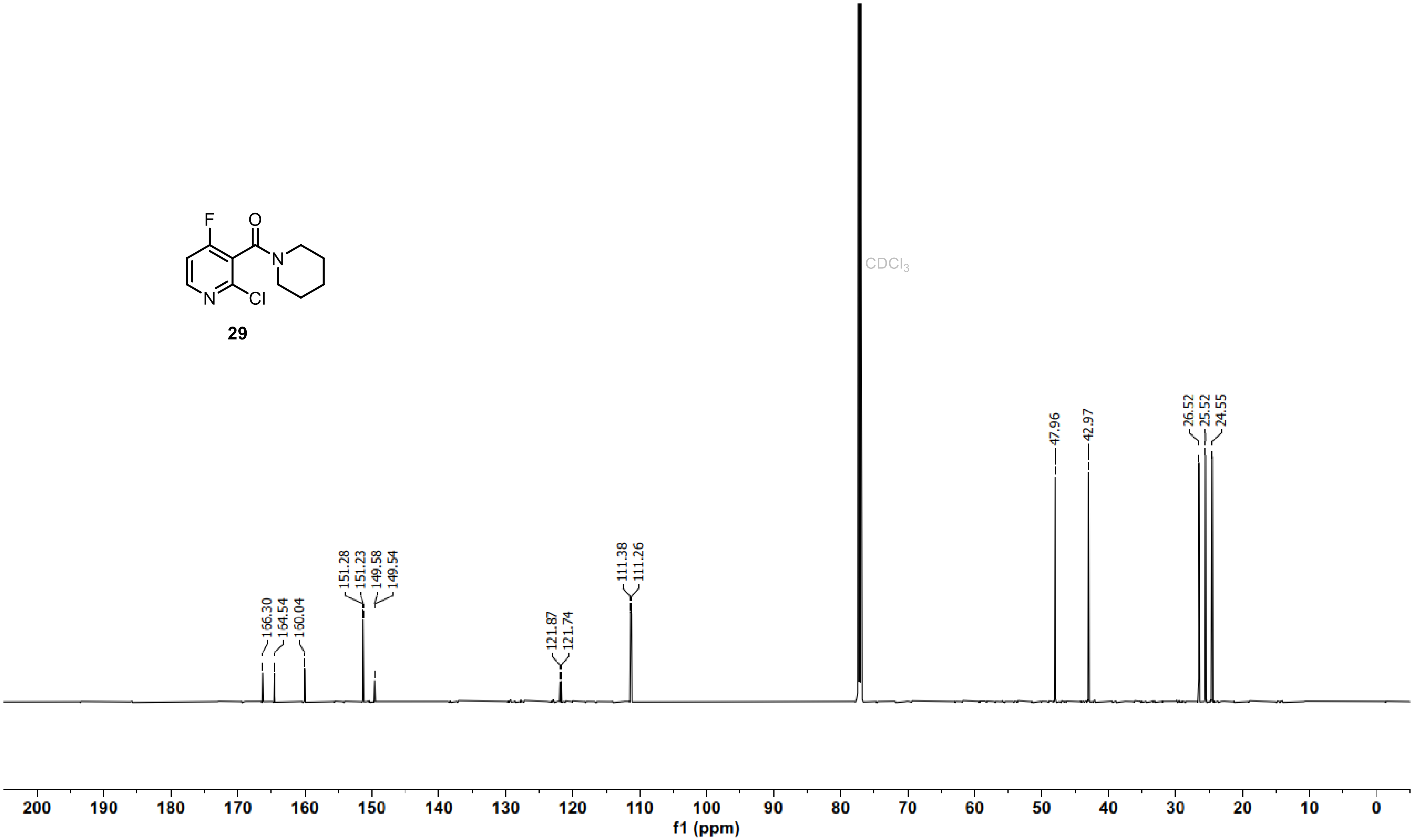

^{19}F NMR of 29

$CDCl_3$, 565 MHz, 25 °C

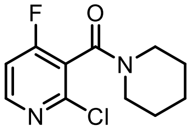

29

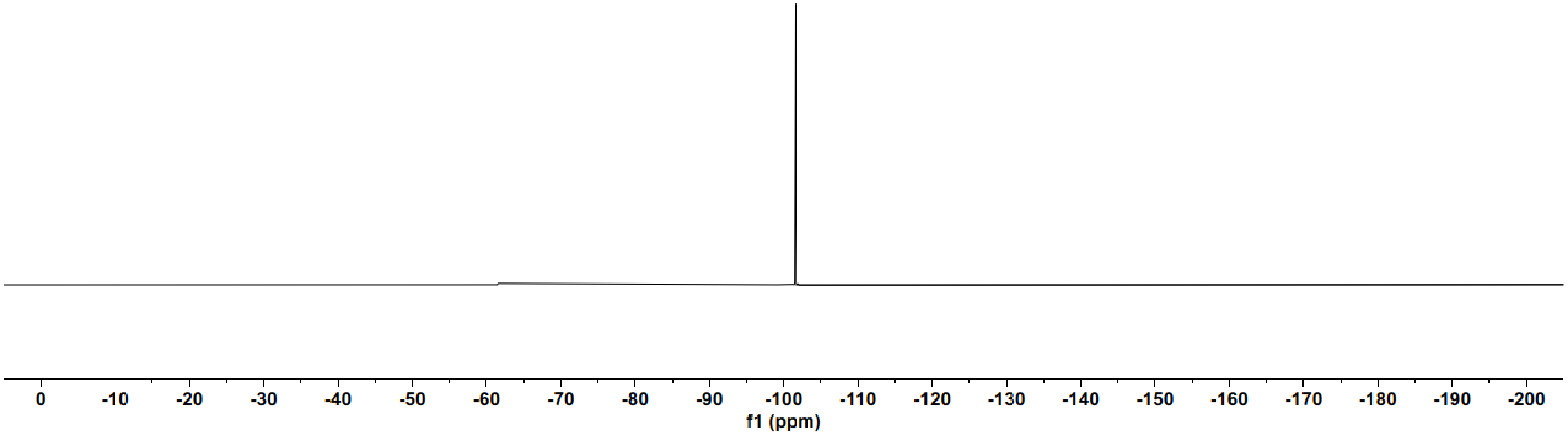

^{1}H NMR of S7

CDCl$_3$, 500 MHz, 25 °C

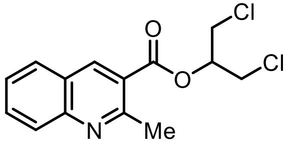

S7

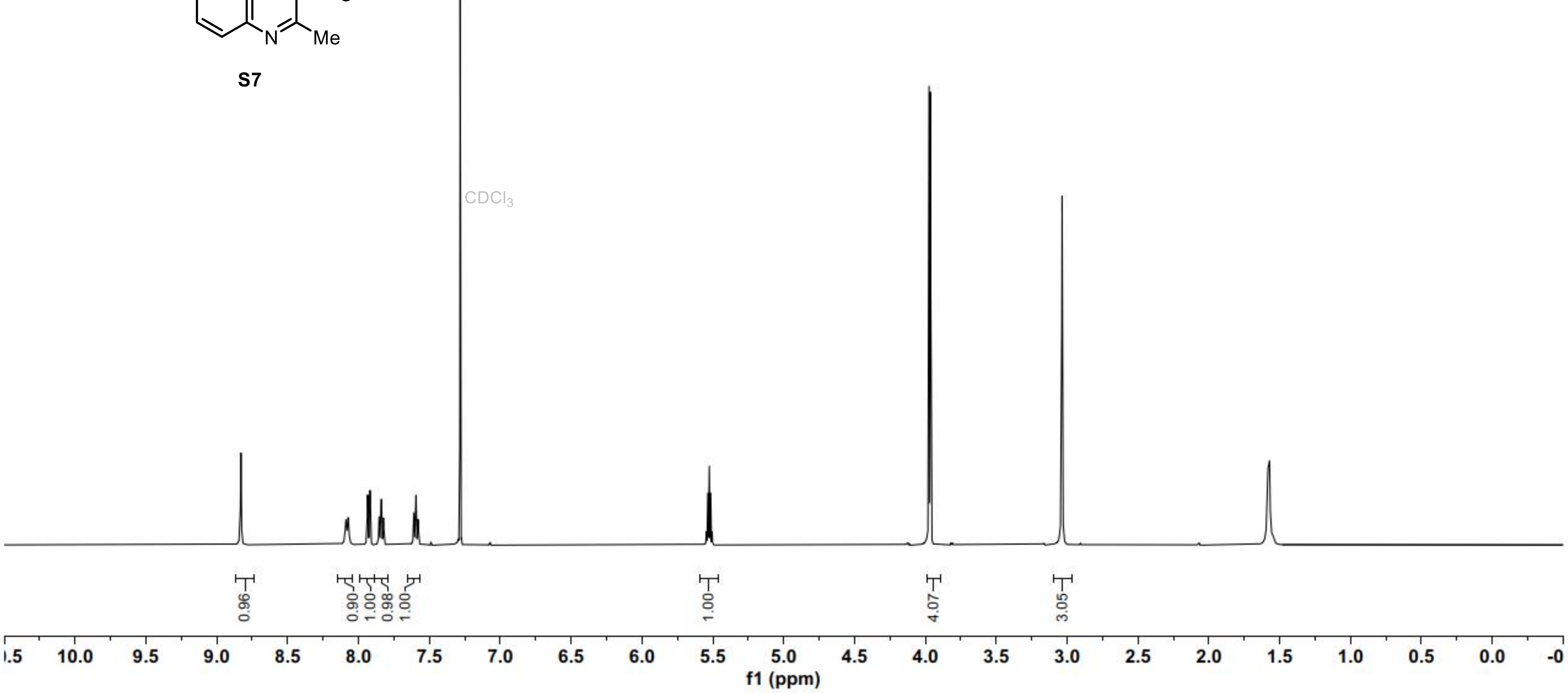

^{13}C NMR of S7

$CDCl_3$, 126 MHz, 25 °C

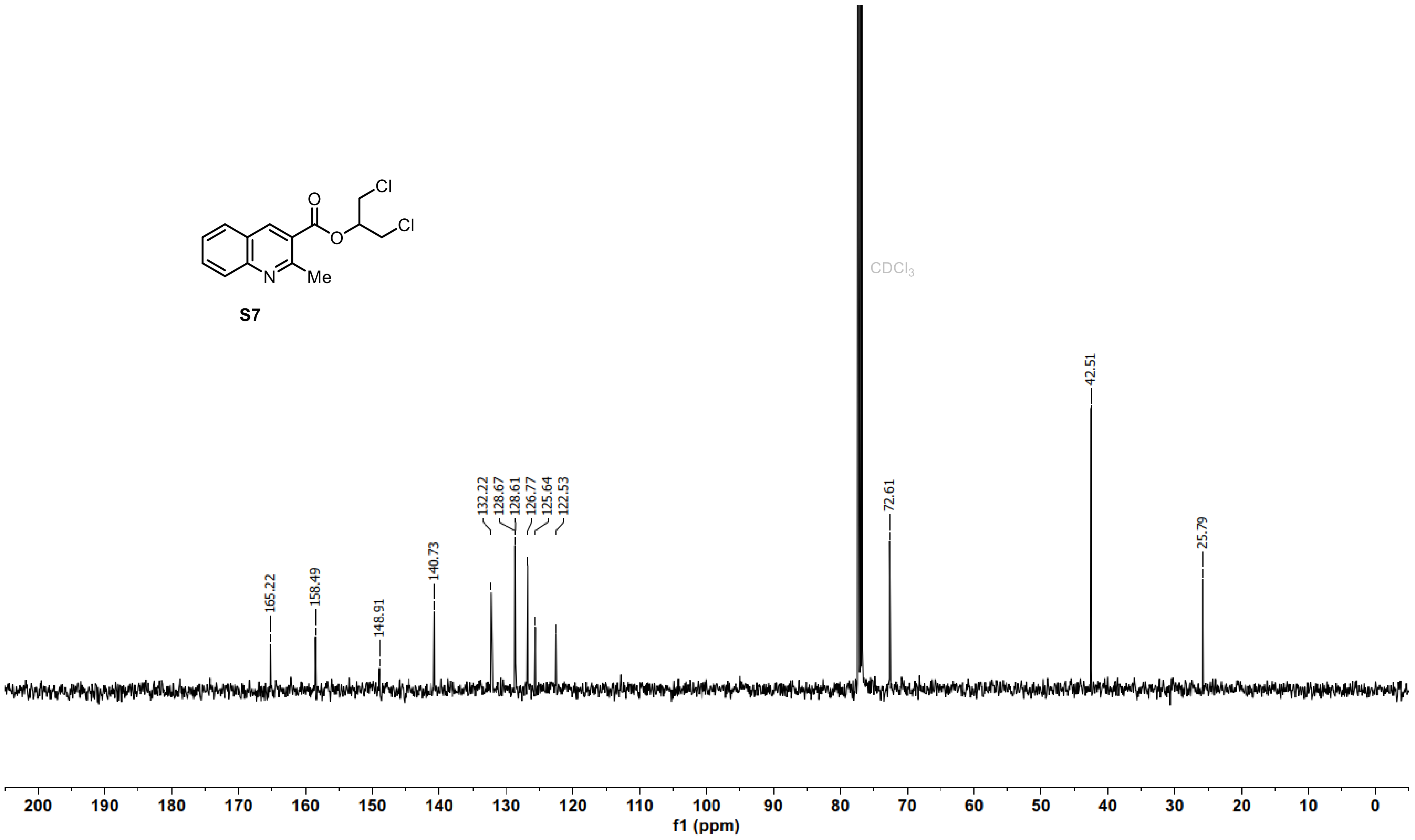

^{1}H NMR of S10

CDCl$_3$, 500 MHz, 25 °C

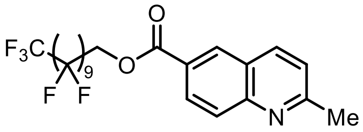

S10

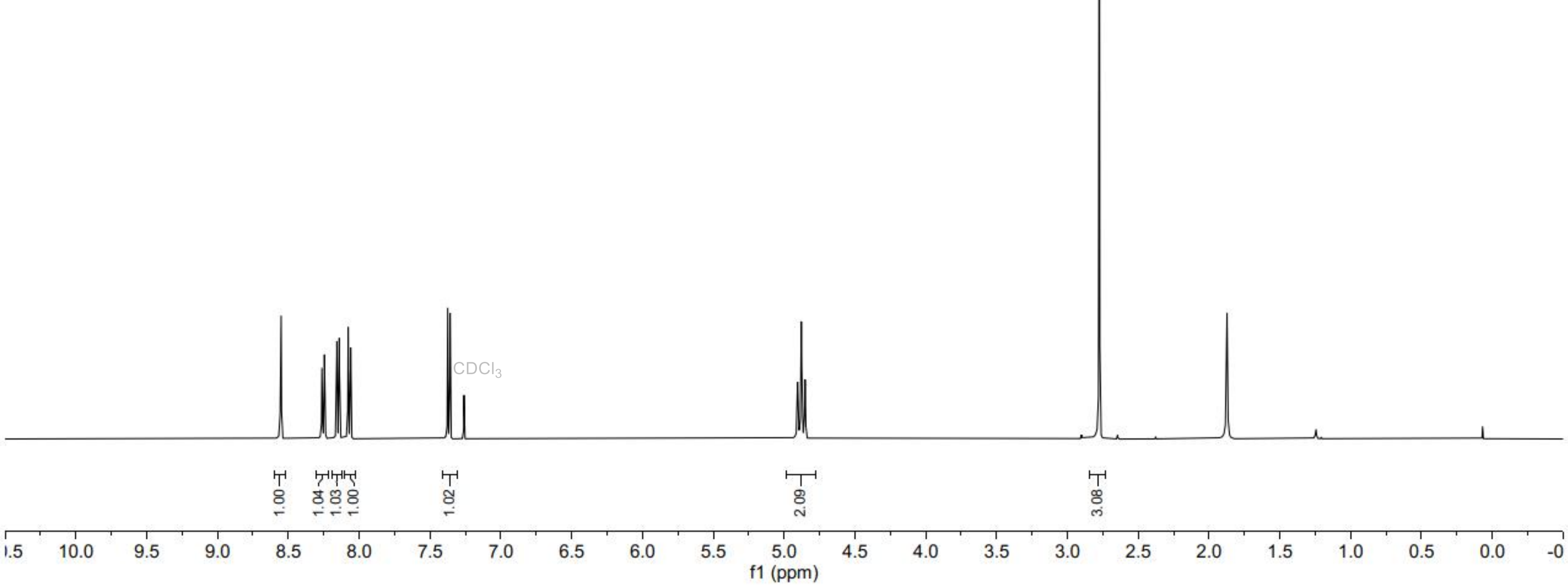

^{13}C NMR of S10

$CDCl_3$, 151 MHz, 25 °C

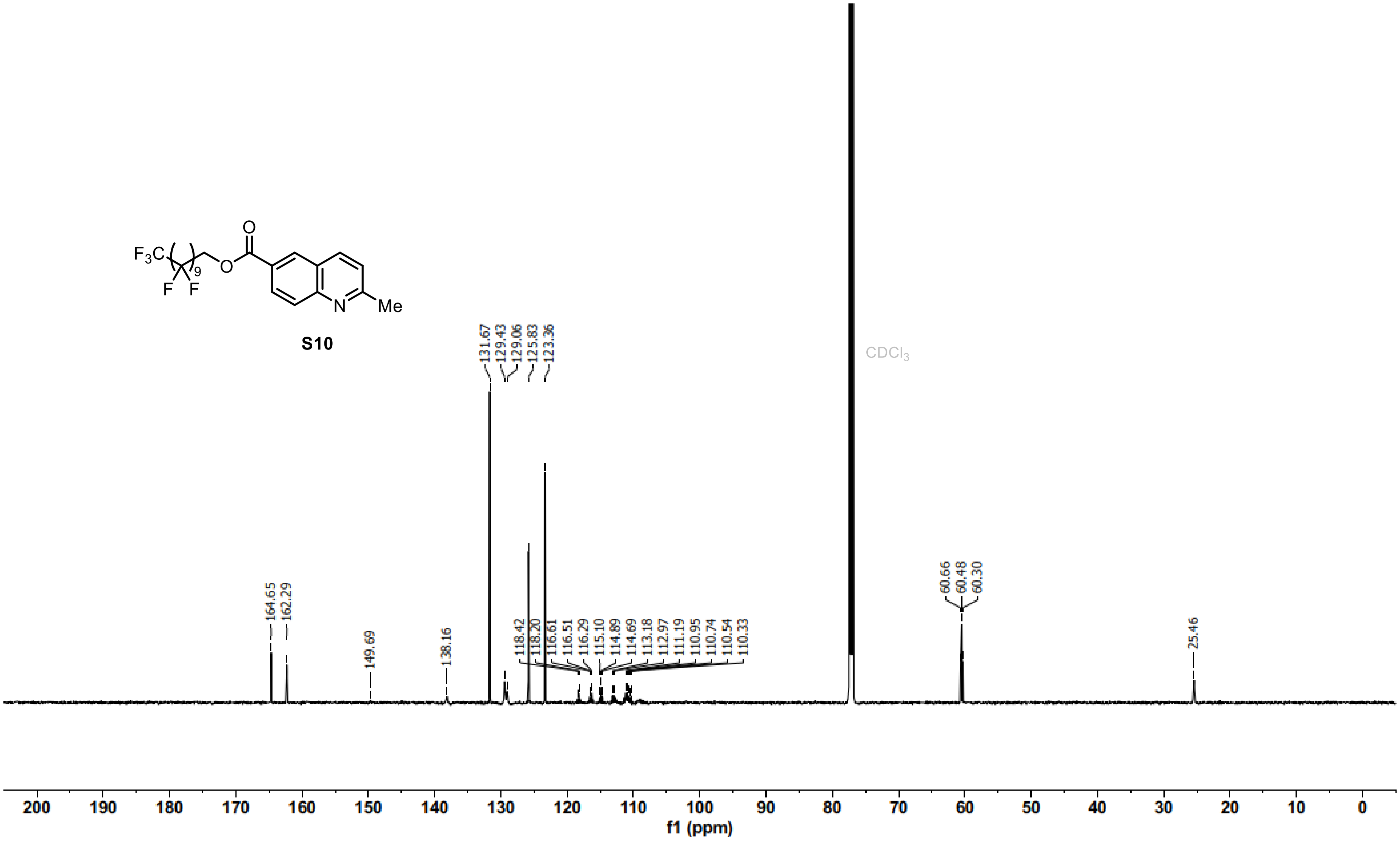

S10

^{19}F NMR of S10

CDCl$_3$, 471 MHz, 25 °C

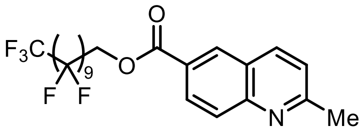

S10

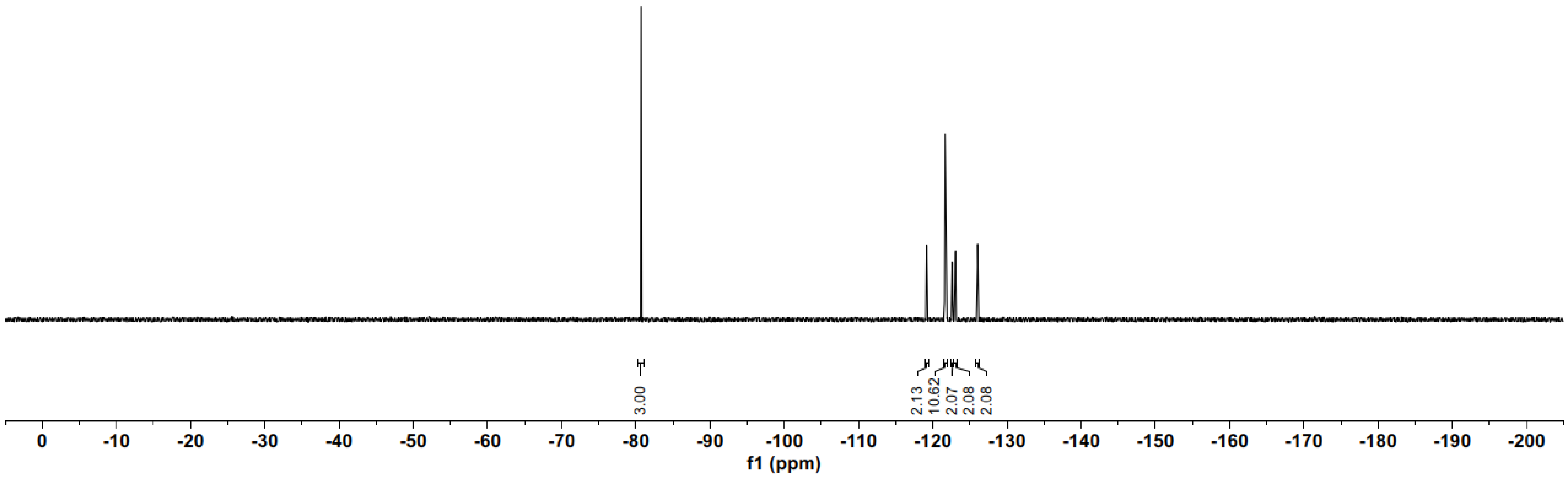

^{1}H NMR of S14

CDCl$_3$, 500 MHz, 25 °C

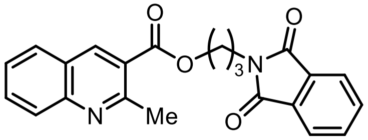

S14

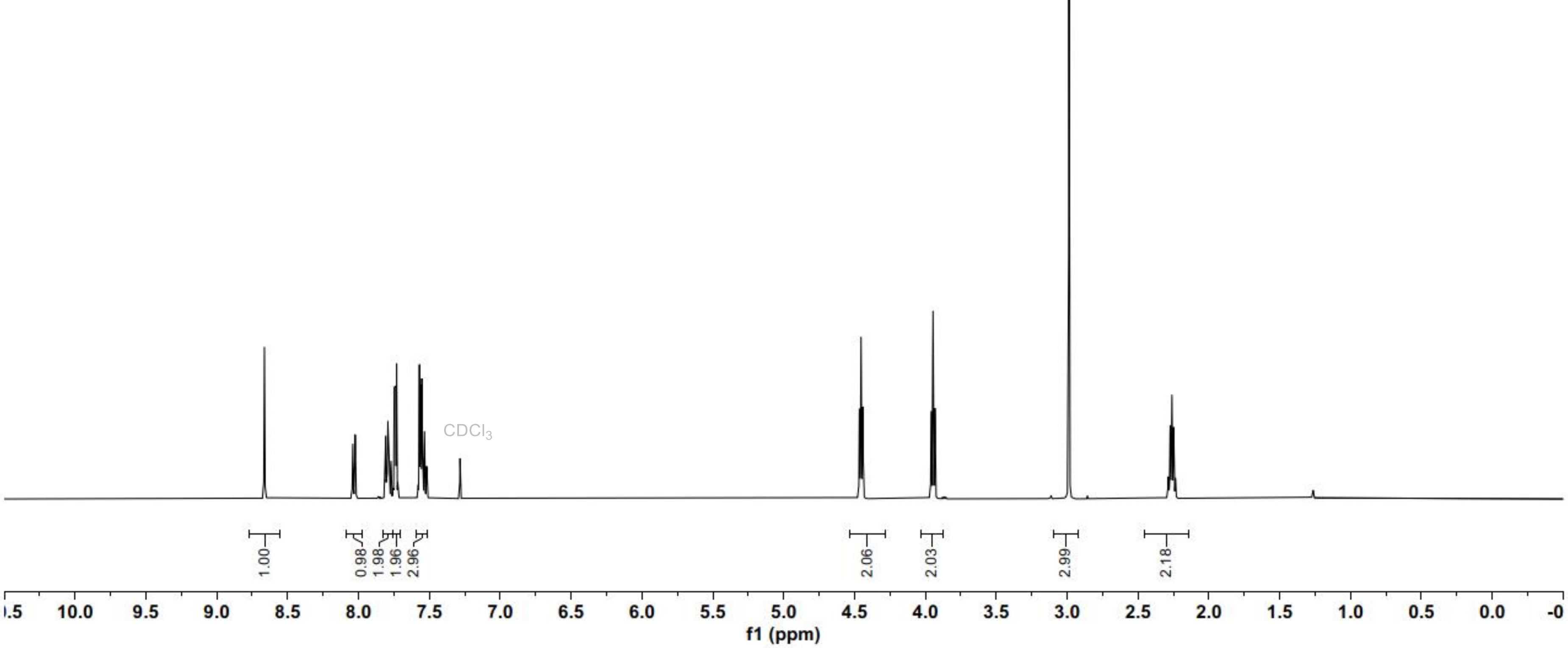

^{13}C NMR of S14

$CDCl_3$, 126 MHz, 25 °C

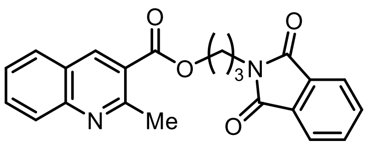

S14

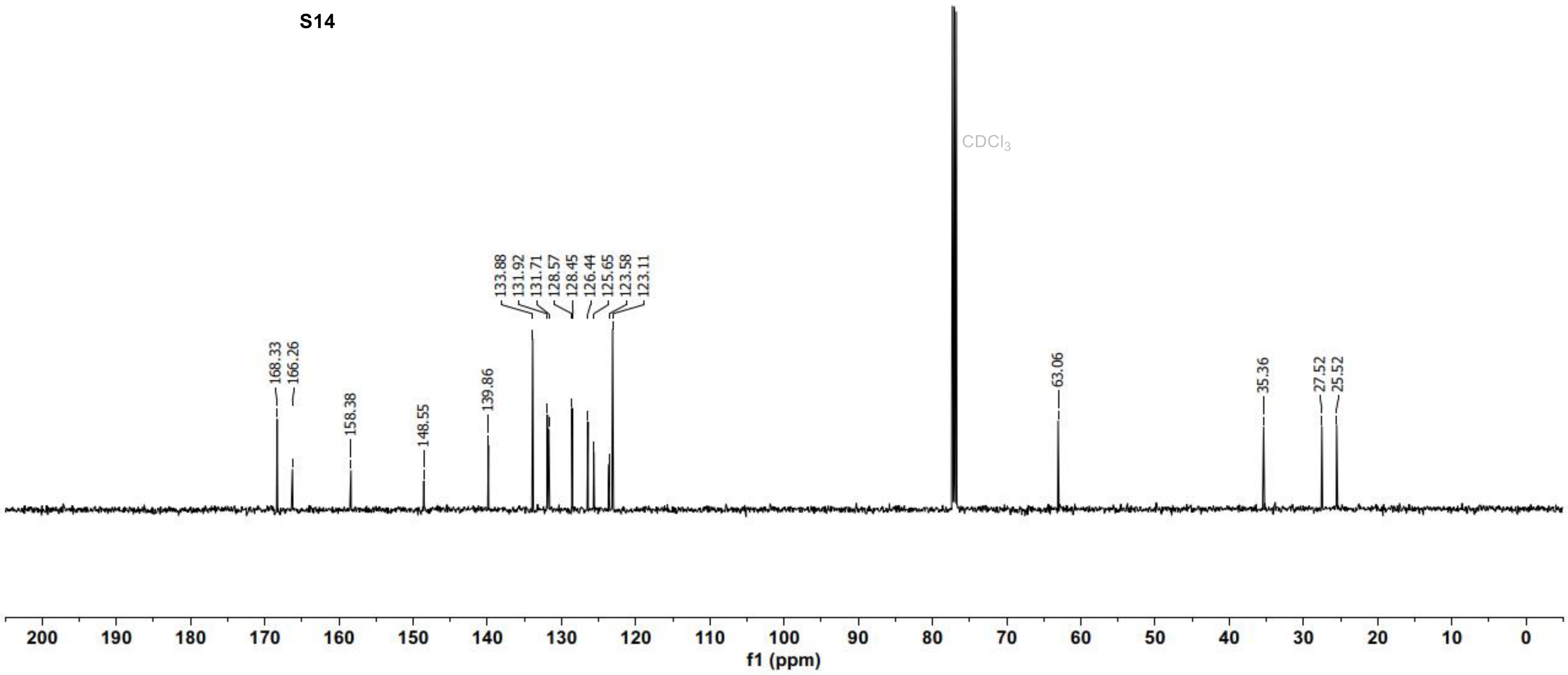

^{1}H NMR of S16

CDCl$_3$, 500 MHz, 25 °C

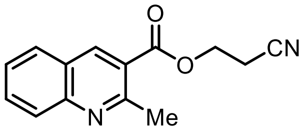

S16

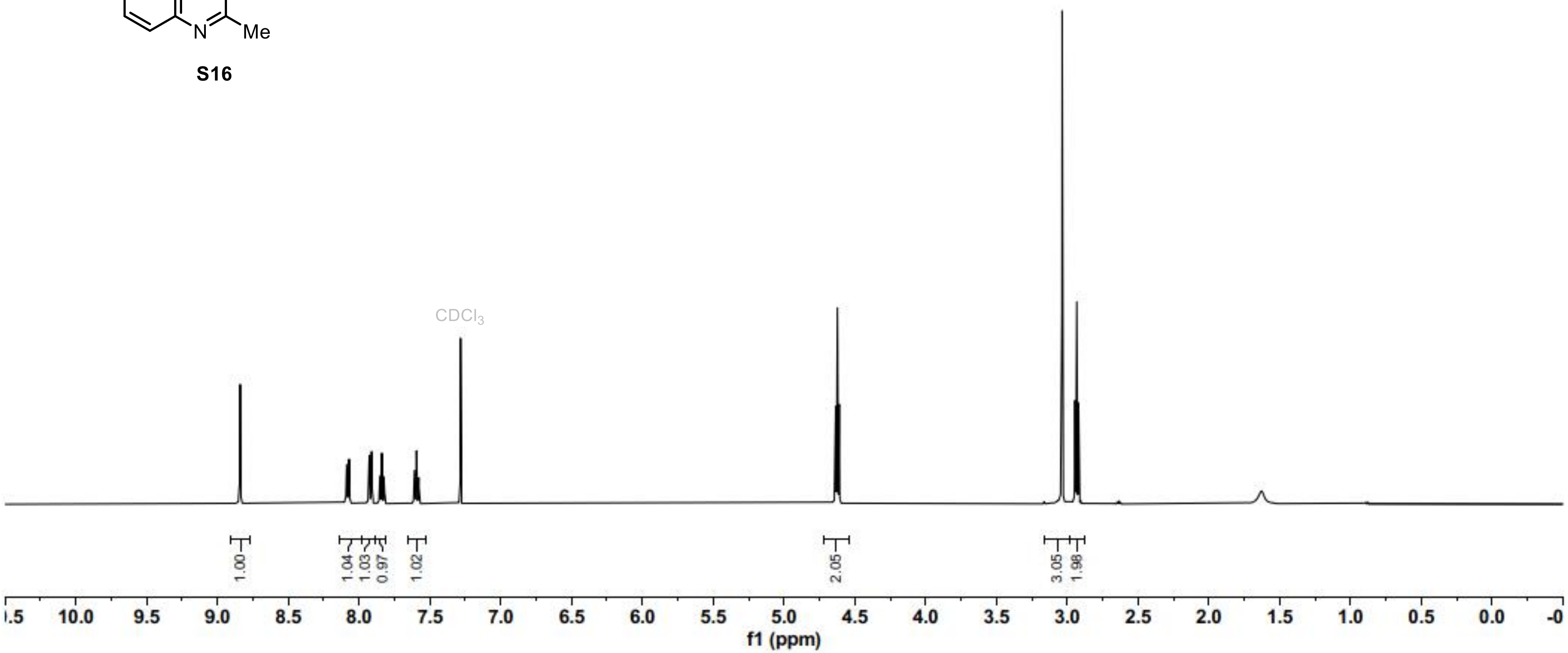

^{13}C NMR of S16

$CDCl_3$, 126 MHz, 25 °C

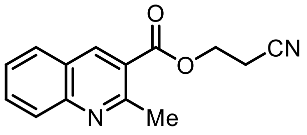

S16

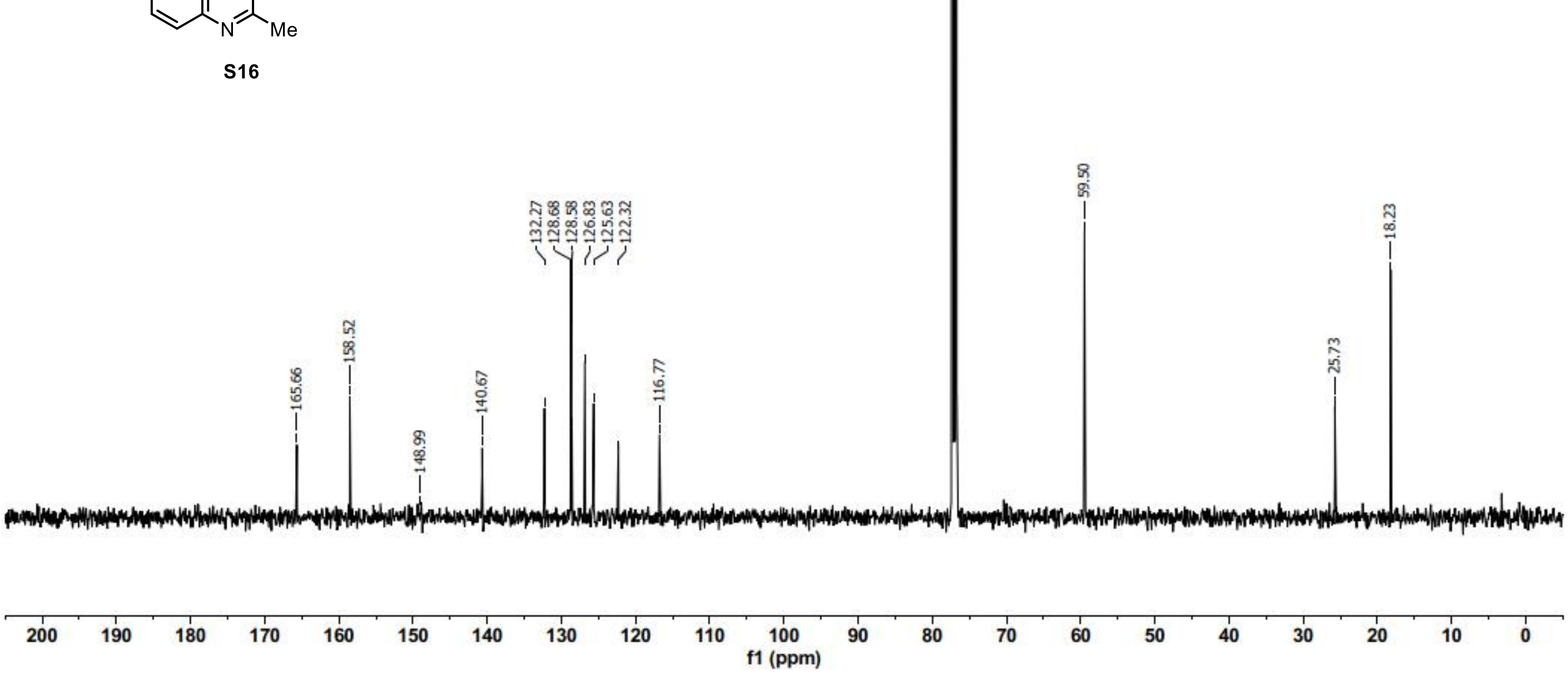

^{1}H NMR of S17

CDCl$_3$, 500 MHz, 25 °C

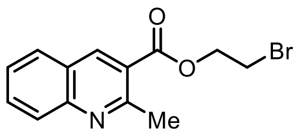

S17

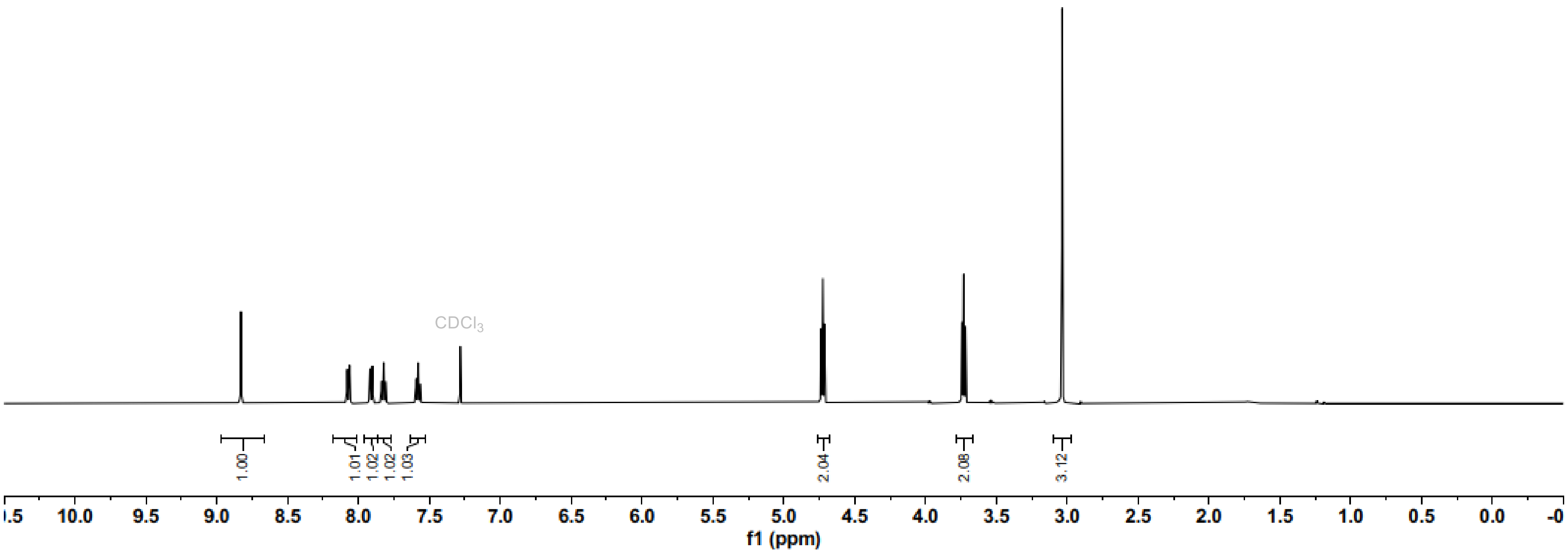

^{13}C NMR of S17

$CDCl_3$, 126 MHz, 25 °C

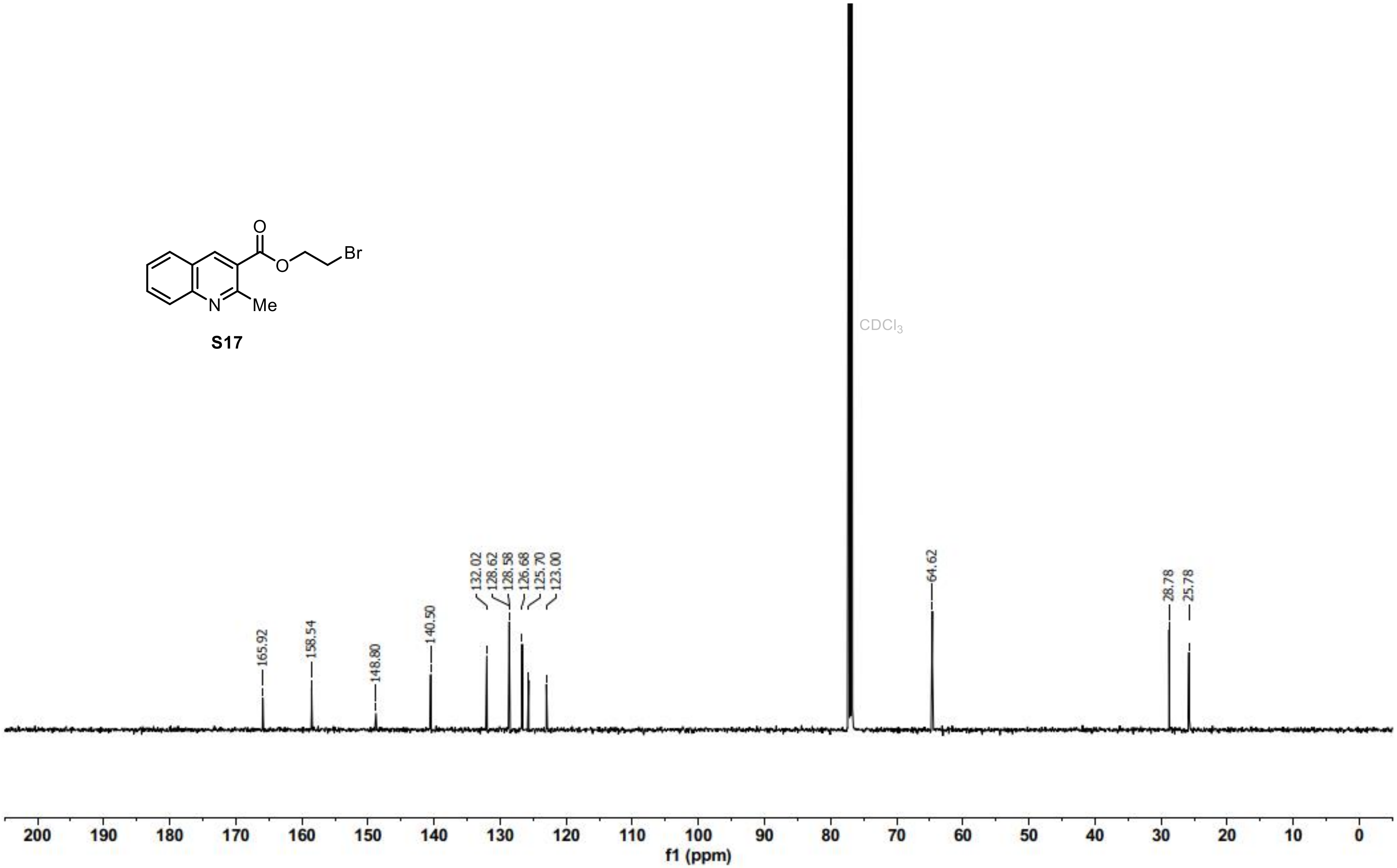

^{1}H NMR of S18

$CDCl_3$, 500 MHz, 25 °C

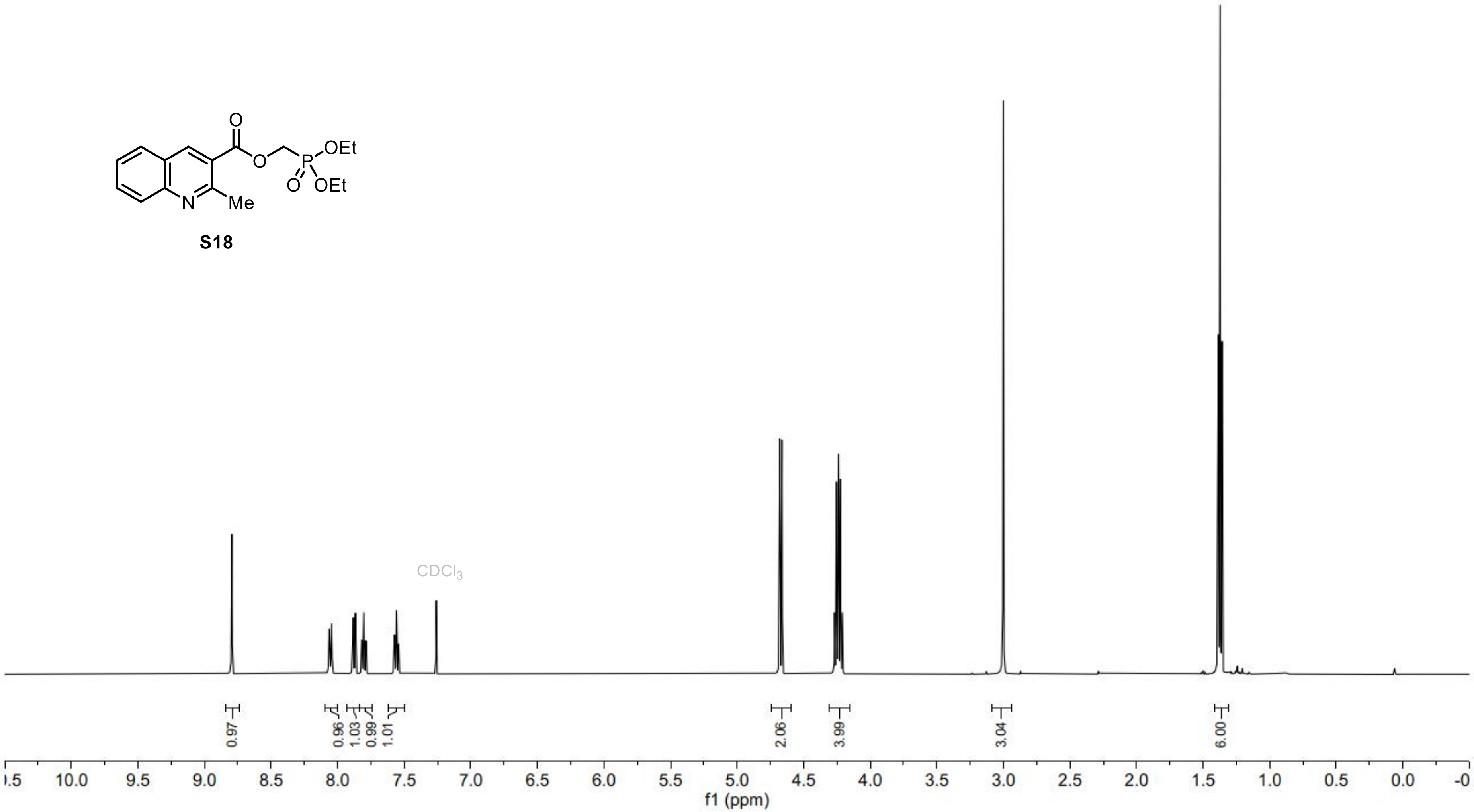

^{13}C NMR of S18

$CDCl_3$, 126 MHz, 25 °C

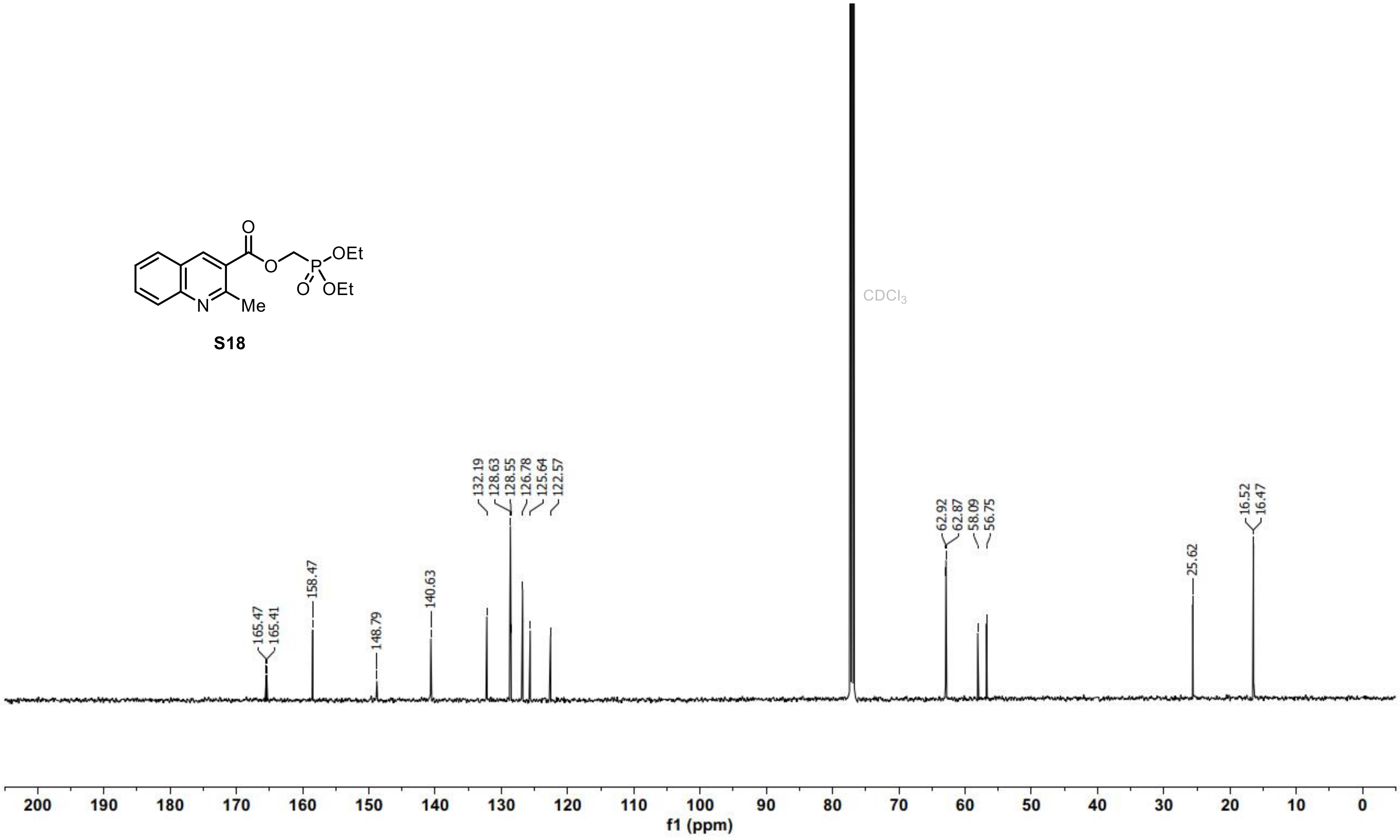

^{31}P NMR of S18

$CDCl_3$, 203 MHz, 25 °C

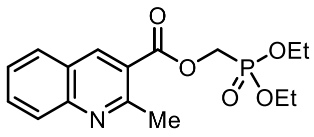

S18

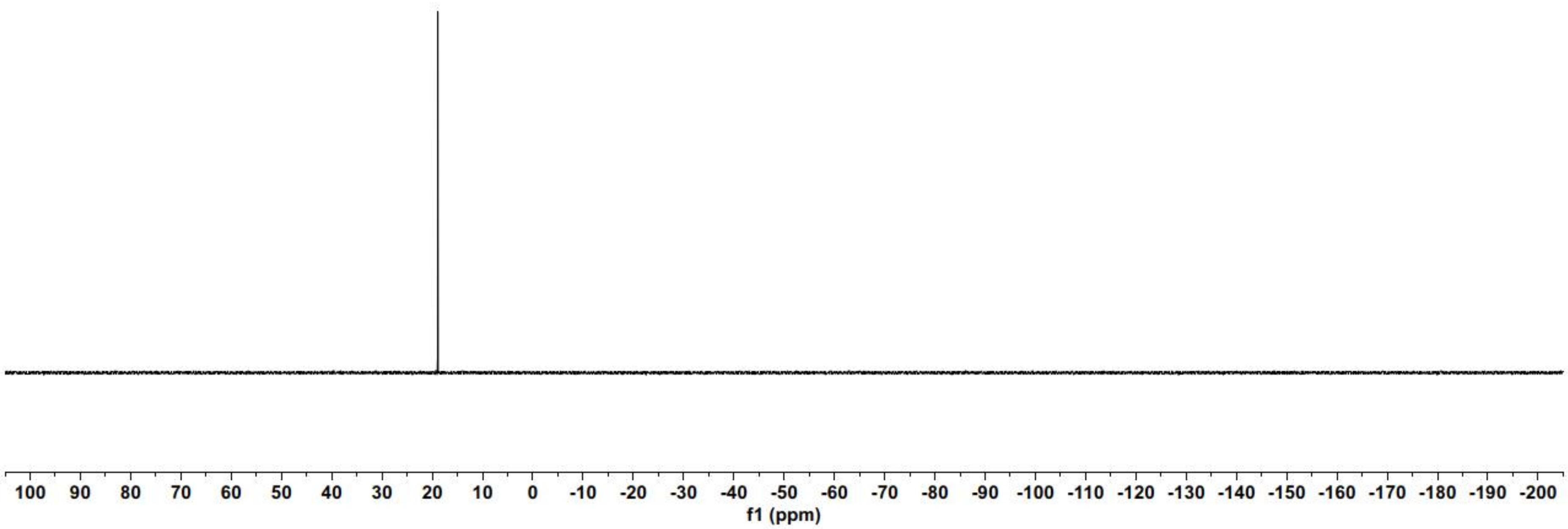

^{1}H NMR of S19

CDCl$_3$, 500 MHz, 25 °C

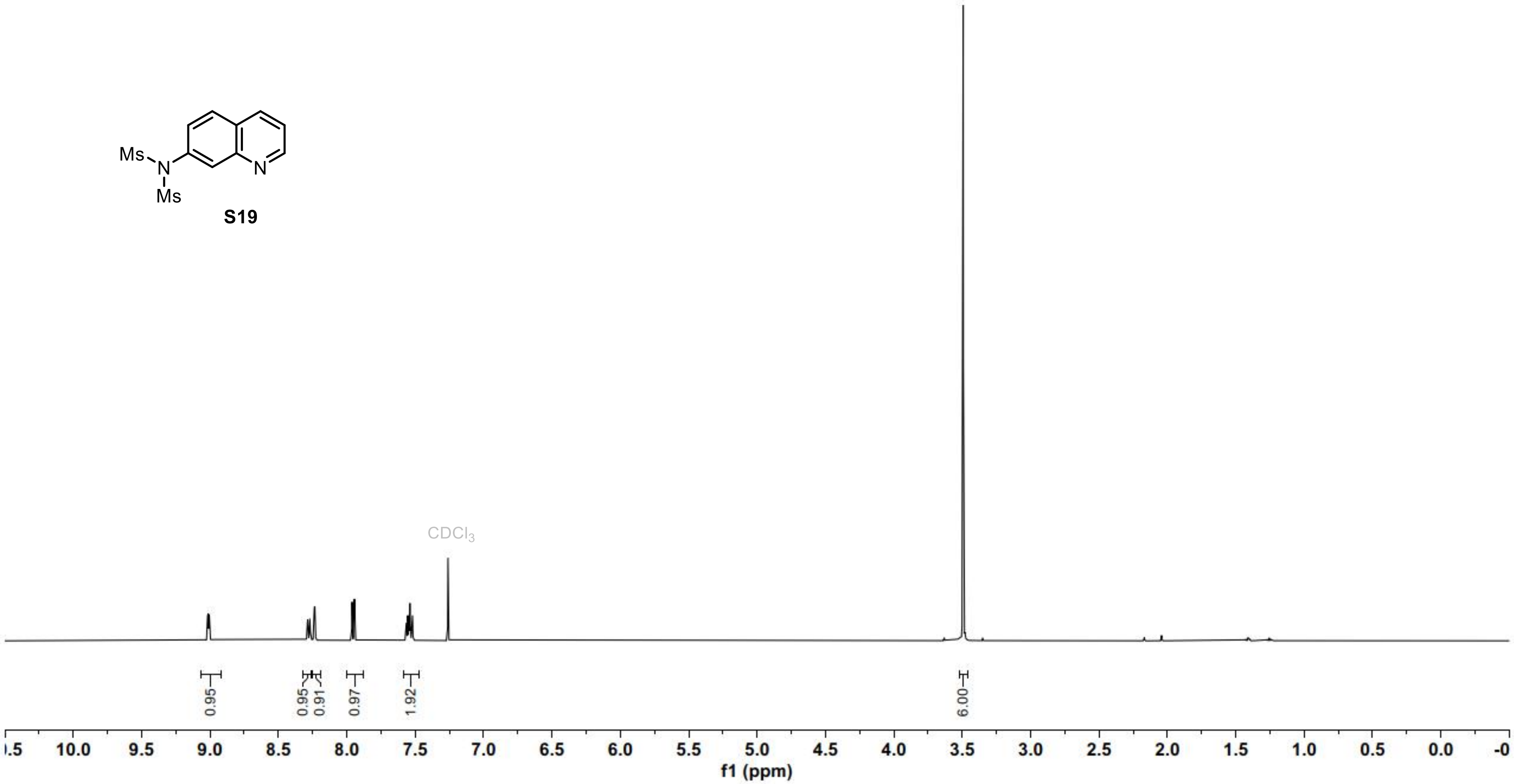

S19

^{13}C NMR of S19

$CDCl_3$, 126 MHz, 25 °C

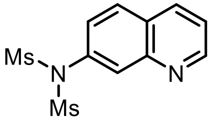

S19

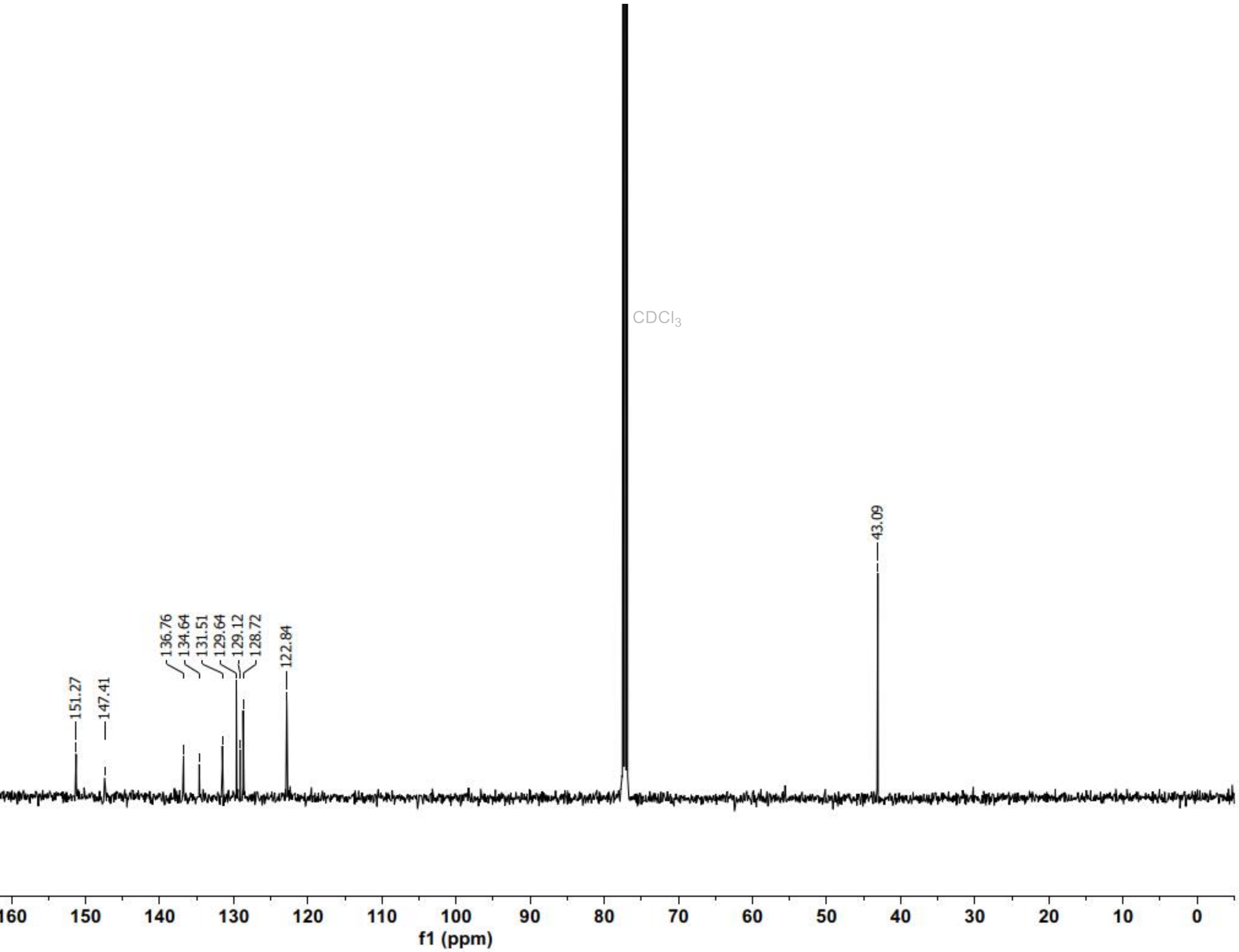

^{1}H NMR of S20

CDCl$_3$, 500 MHz, 25 °C

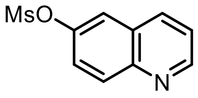

S20

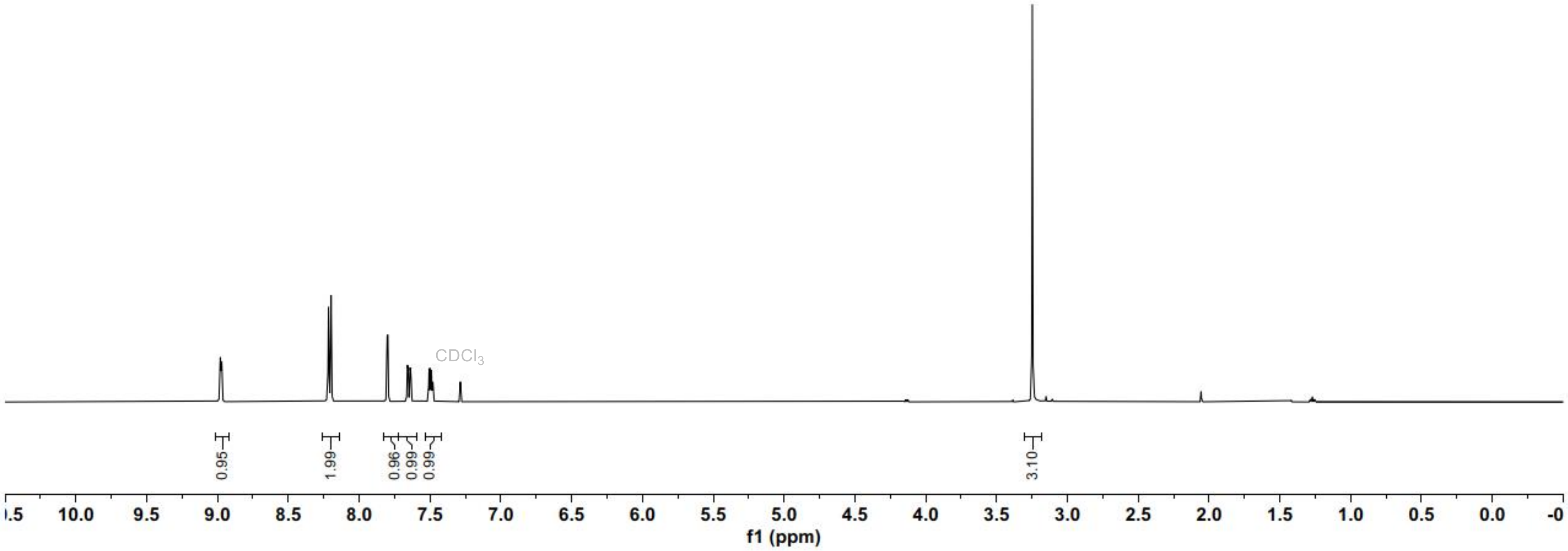

^{13}C NMR of S20

$CDCl_3$, 126 MHz, 25 °C

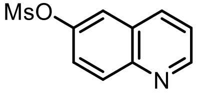

S20

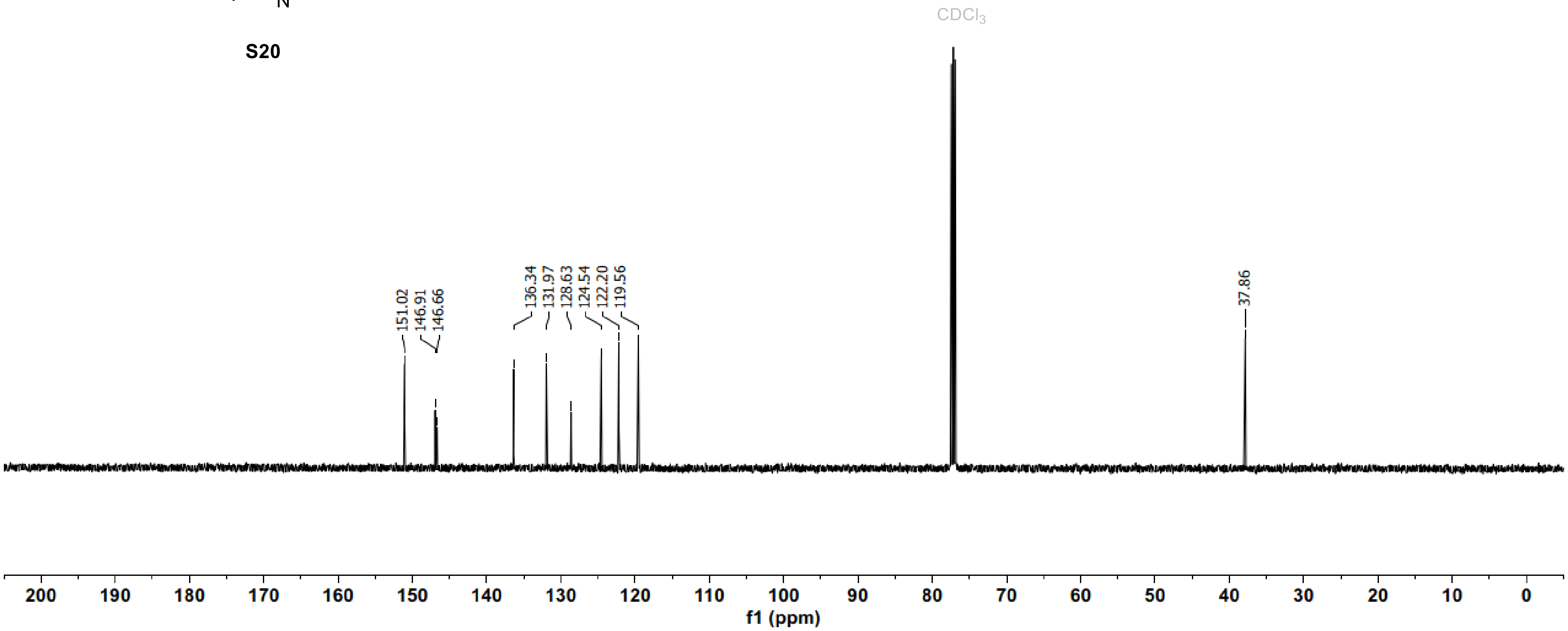

^{1}H NMR of S22

CDCl$_3$, 500 MHz, 25 °C

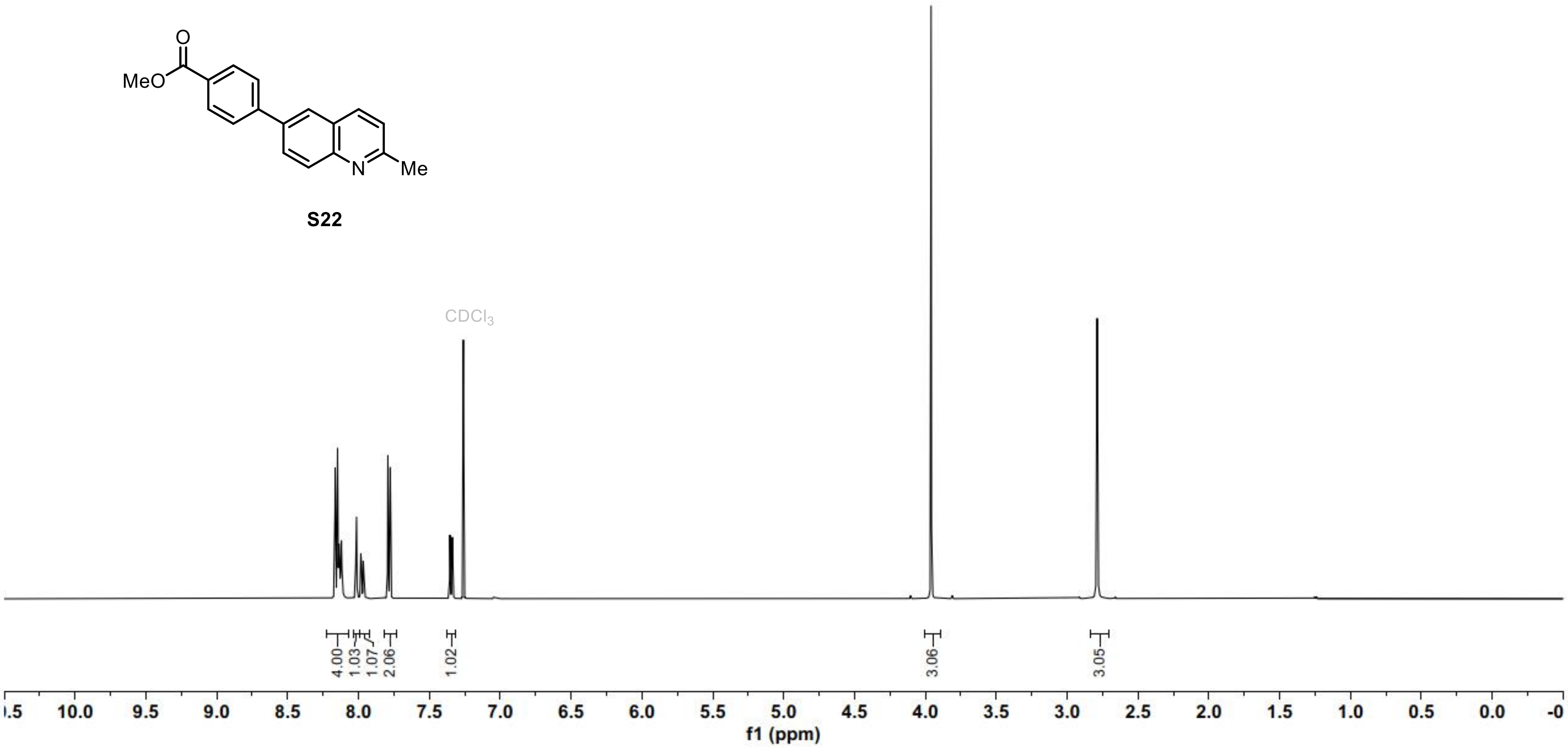

^{13}C NMR of S22

$CDCl_3$, 126 MHz, 25 °C

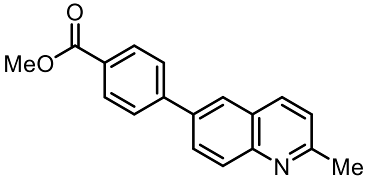

S22

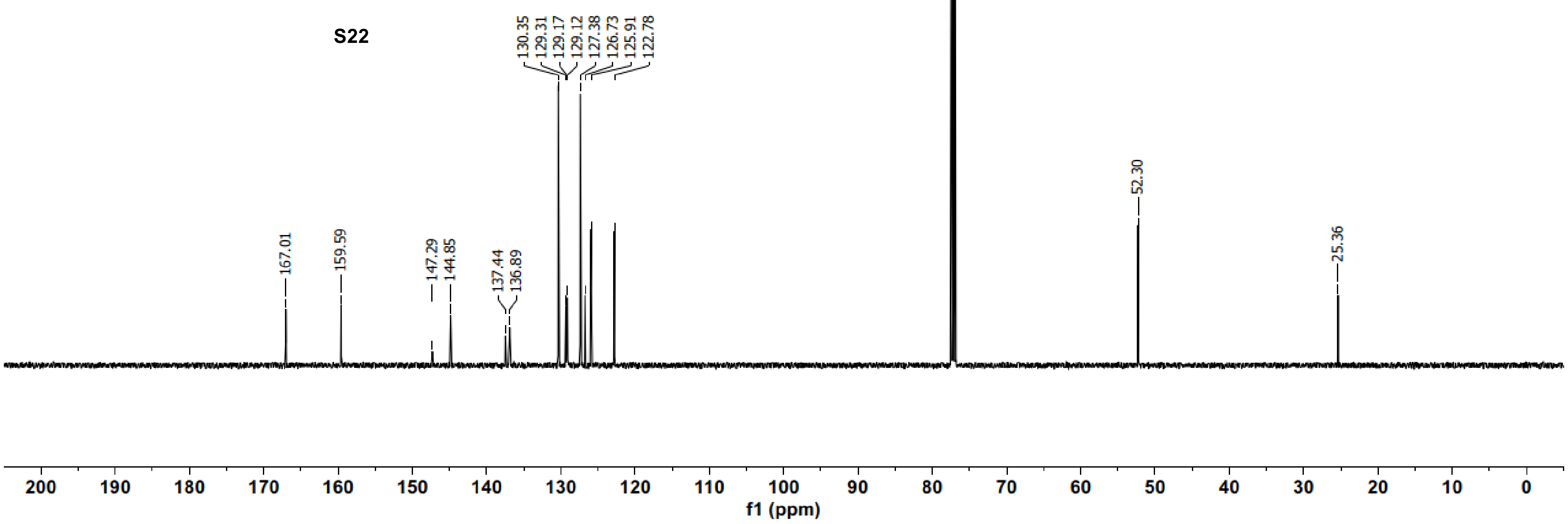

^{1}H NMR of S23

CDCl$_3$, 500 MHz, 25 °C

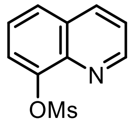

S23

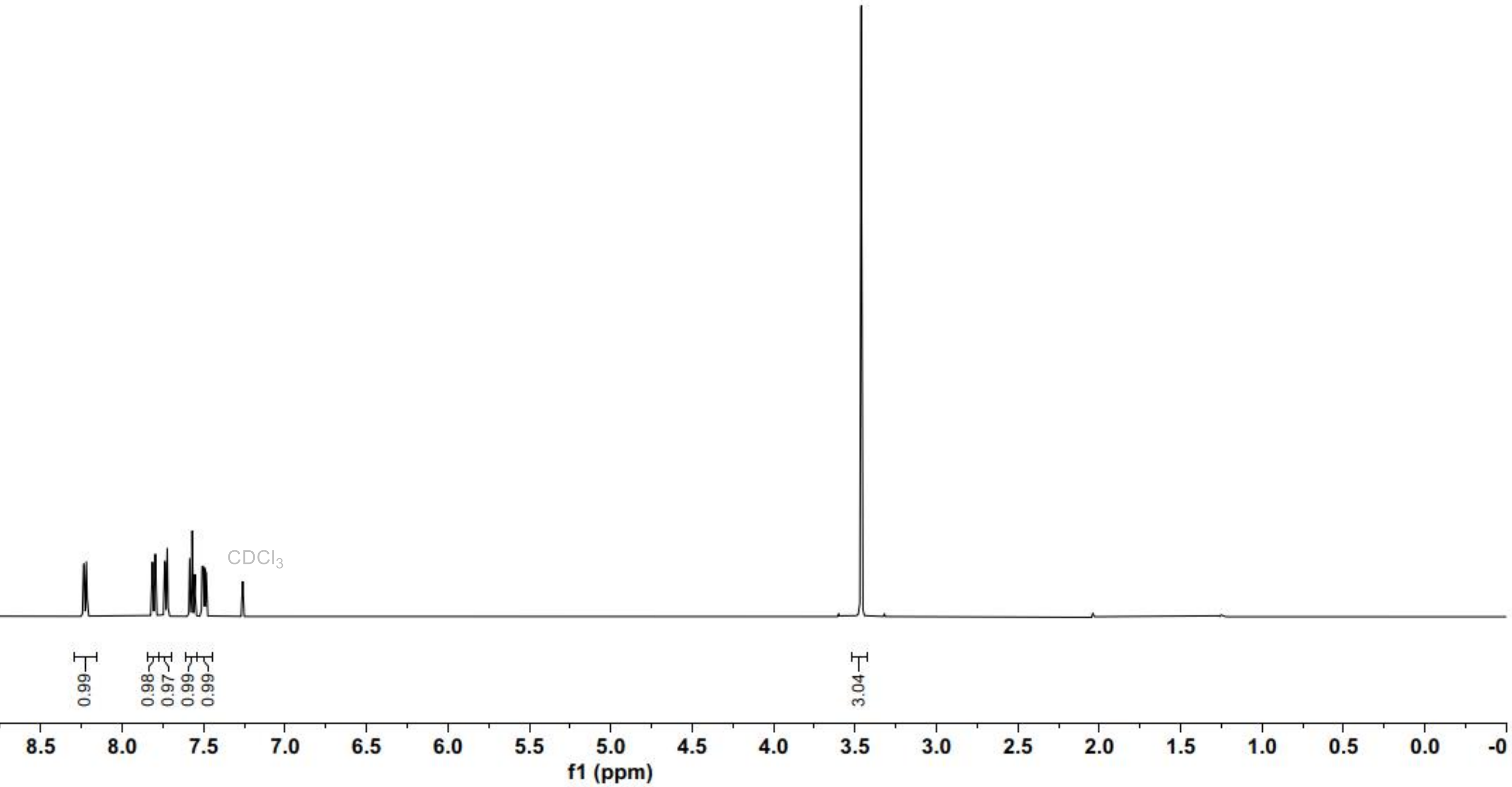

^{13}C NMR of S23

$CDCl_3$, 126 MHz, 25 °C

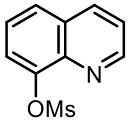

S23

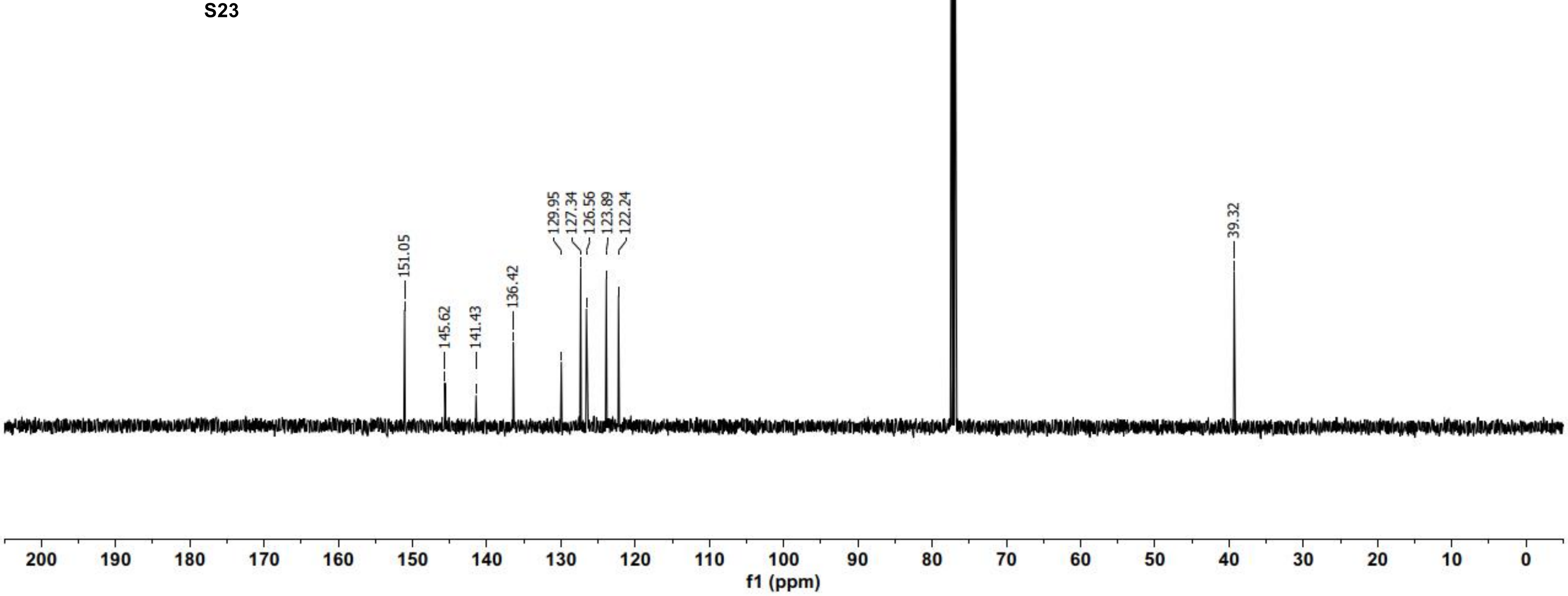

^{1}H NMR of S24

CDCl$_3$, 500 MHz, 25 °C

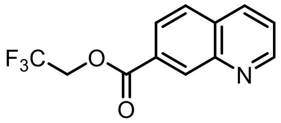

S24

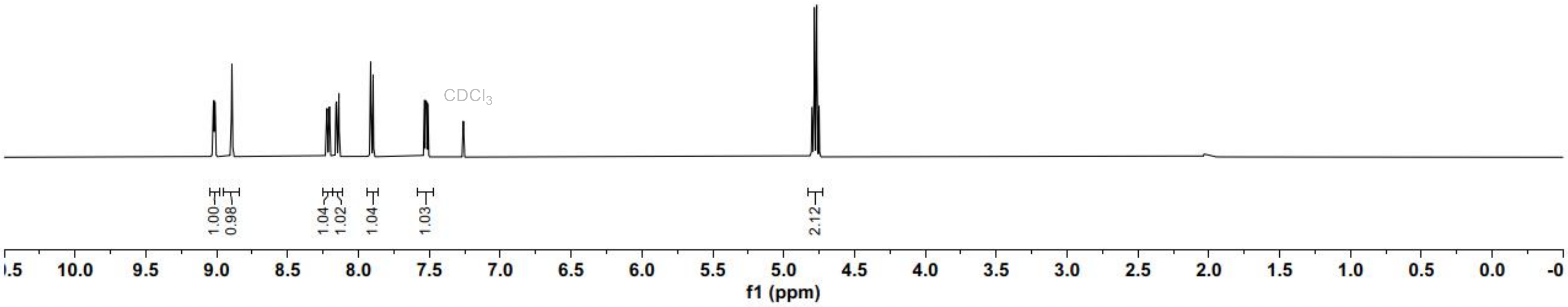

^{13}C NMR of S24

$CDCl_3$, 126 MHz, 25 °C

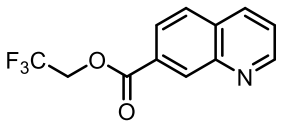

S24

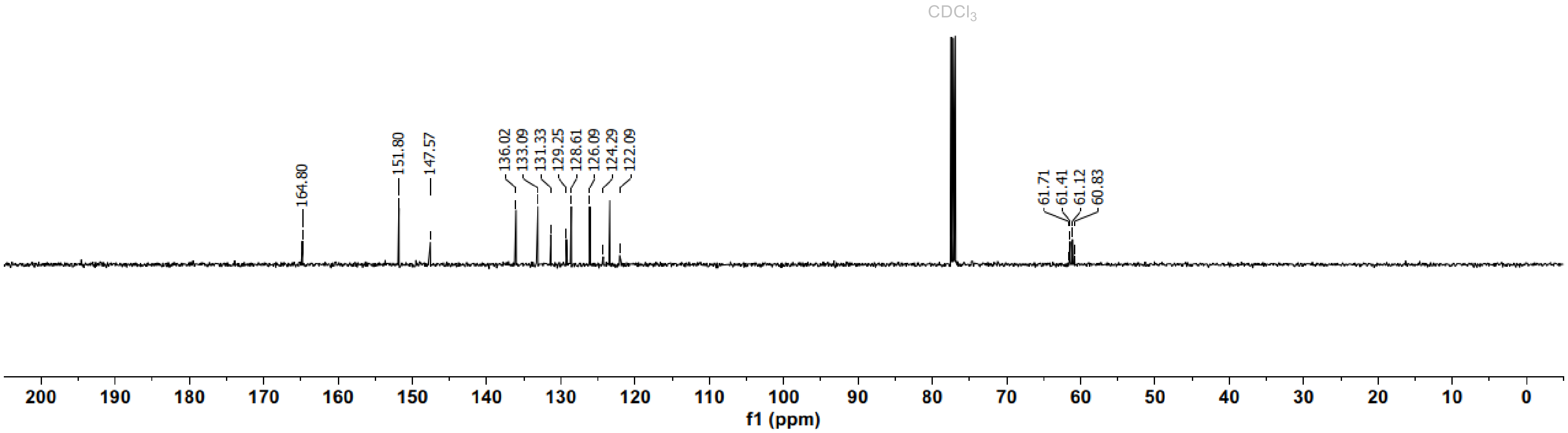

^{19}F NMR of S24

$CDCl_3$, 471 MHz, 25 °C

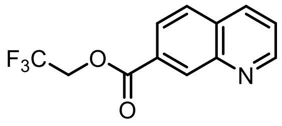

S24

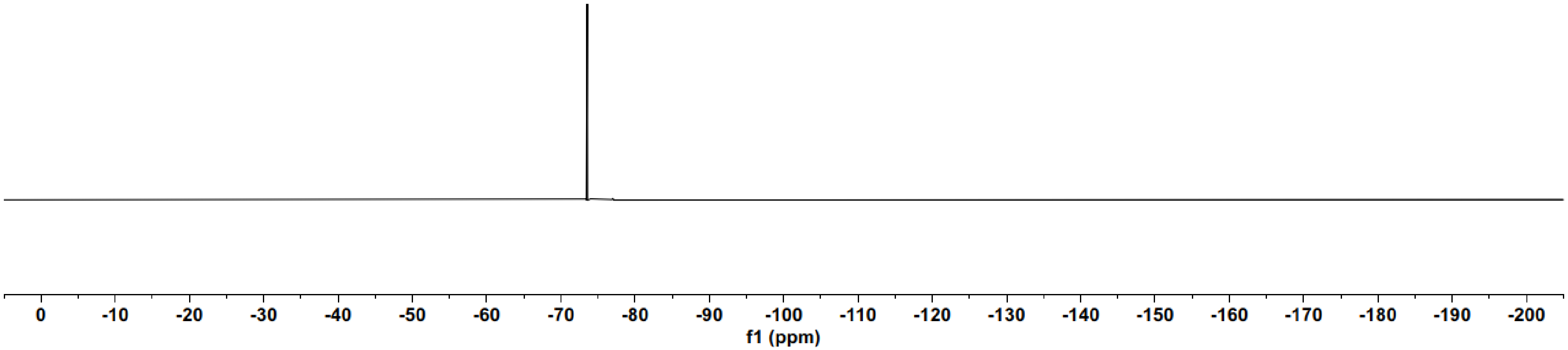

^{1}H NMR of S26

$CDCl_3$, 500 MHz, 25 °C

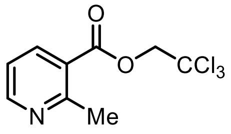

S26

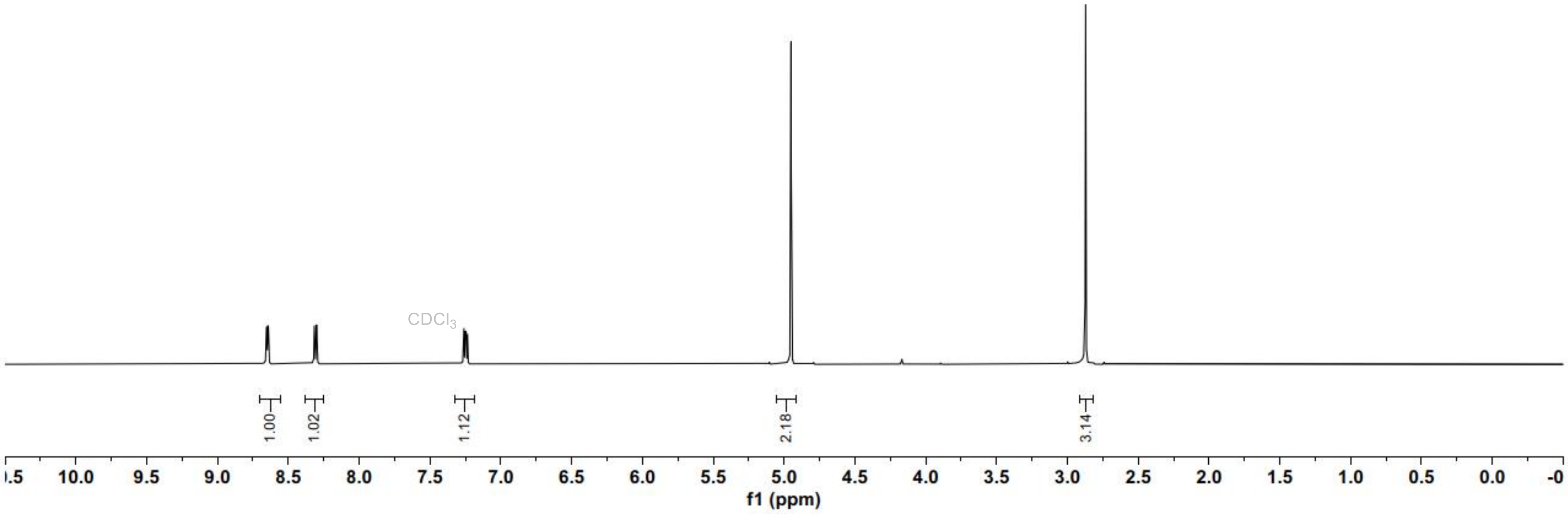

^{13}C NMR of S26

$CDCl_3$, 126 MHz, 25 °C

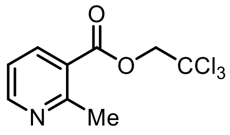

S26

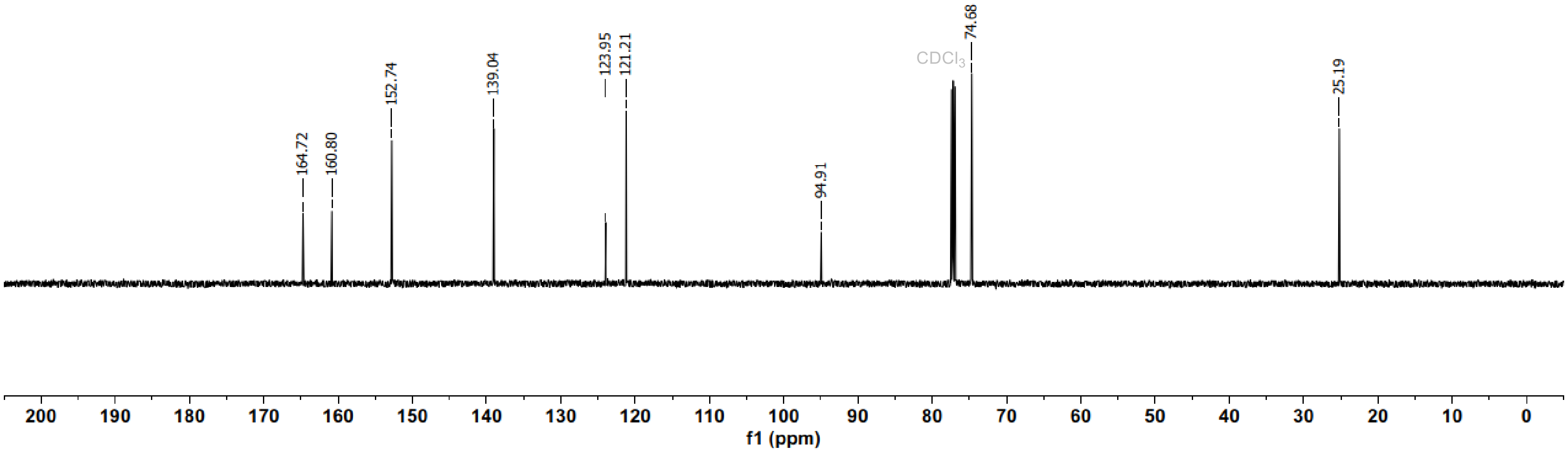

^{1}H NMR of S27

CDCl$_3$, 500 MHz, 25 °C

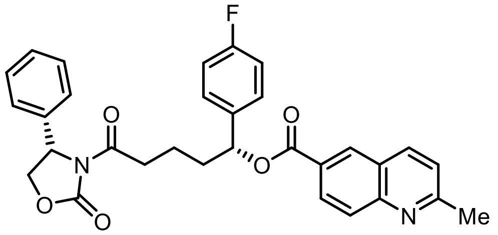

S27

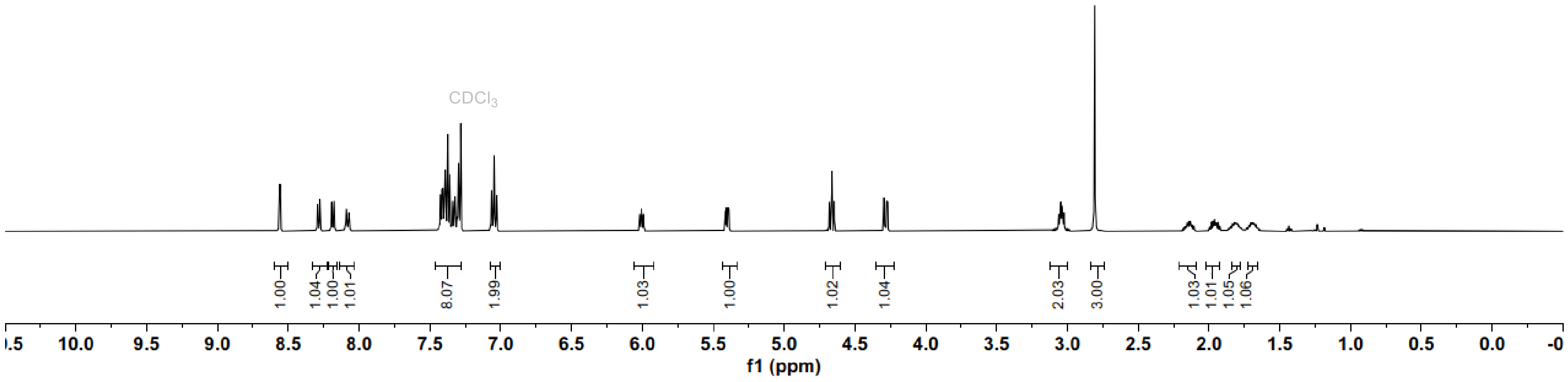

^{13}C NMR of S27

$CDCl_3$, 126 MHz, 25 °C

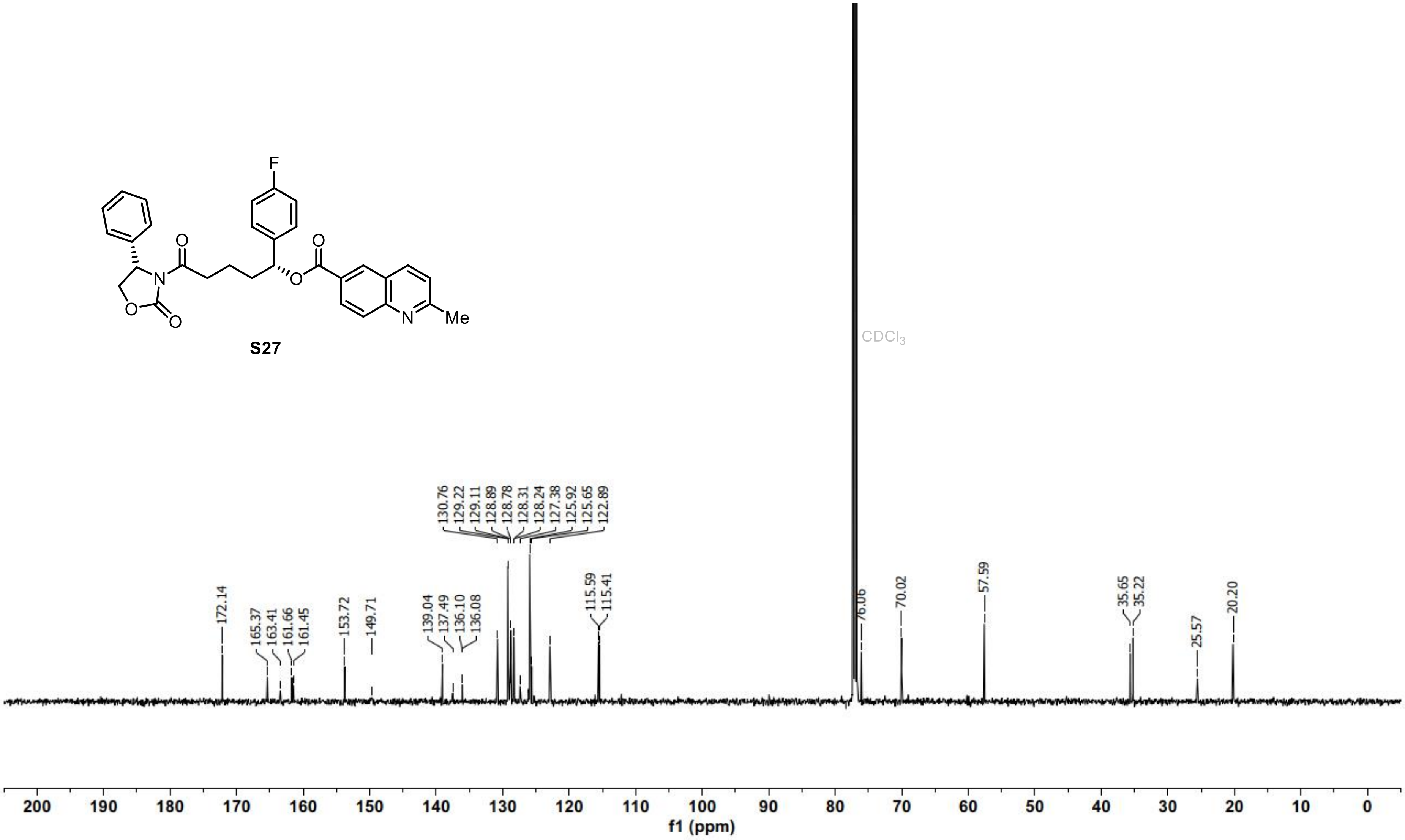

^{19}F NMR of S27

$CDCl_3$, 471 MHz, 25 °C

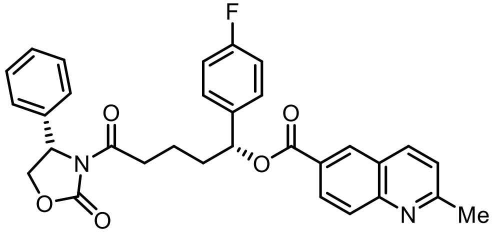

S27

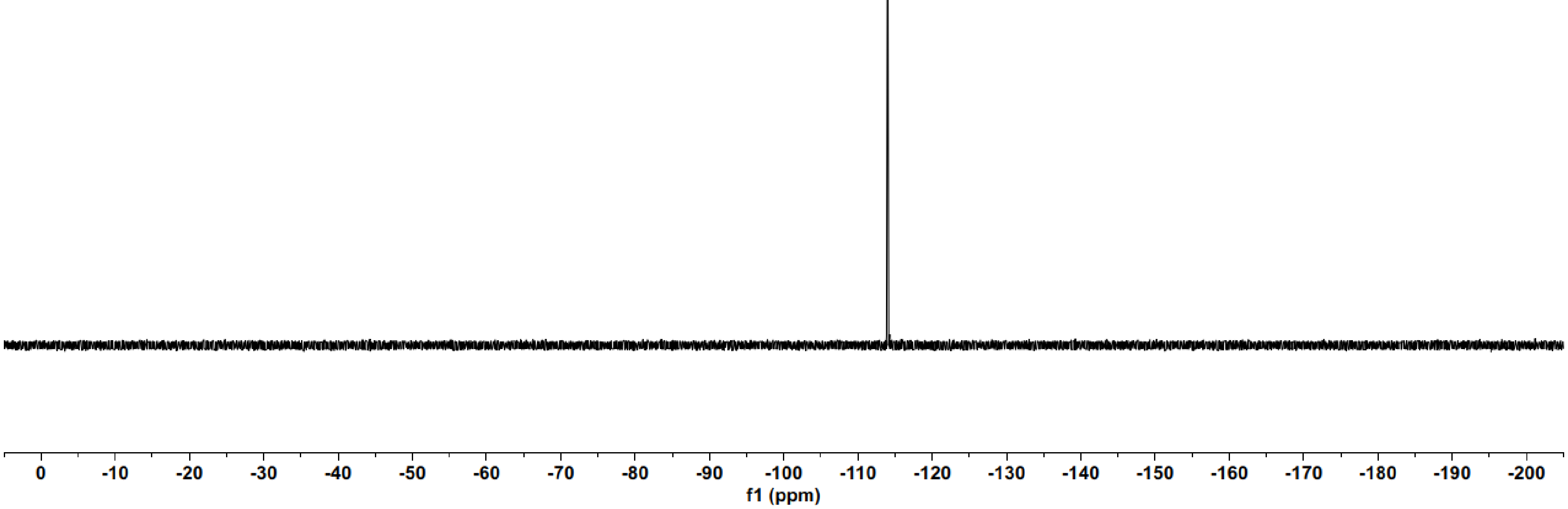

^{1}H NMR of S28

CDCl$_3$, 500 MHz, 25 °C

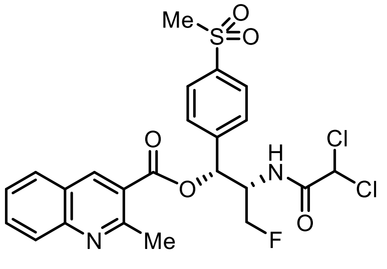

S28

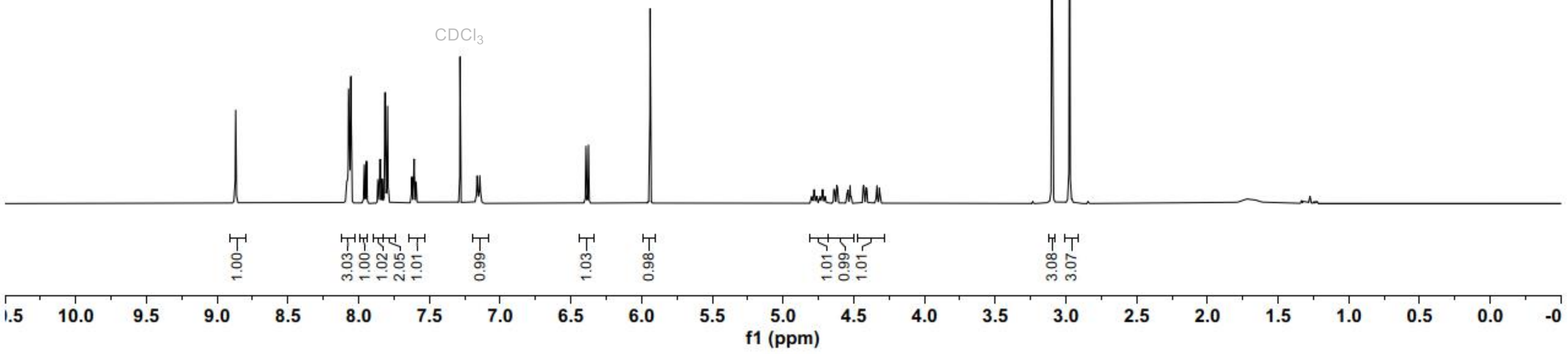

^{13}C NMR of S28

CDCl$_3$, 126 MHz, 25 °C

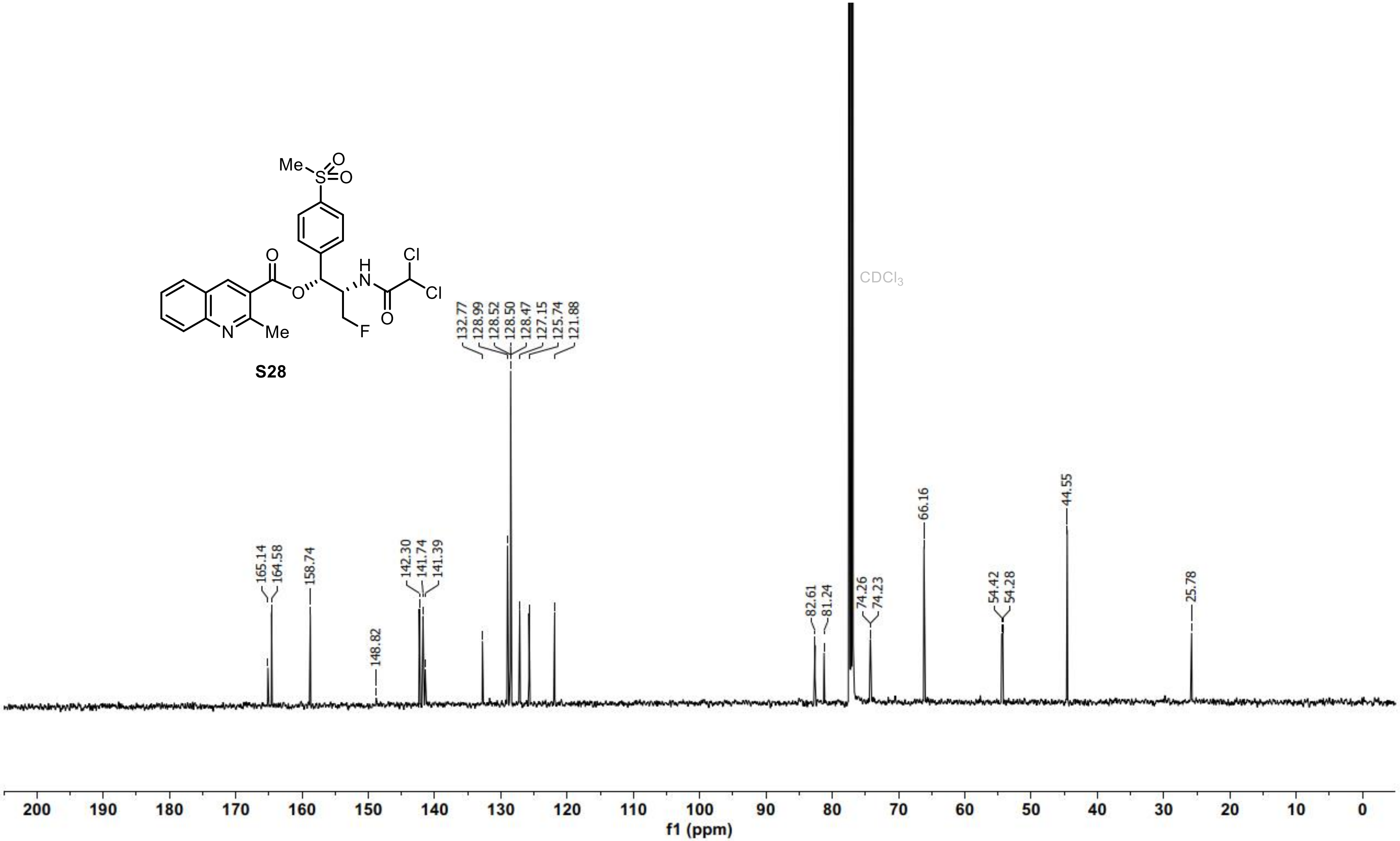

^{19}F NMR of S28

CDCl3, 471 MHz, 25 °C

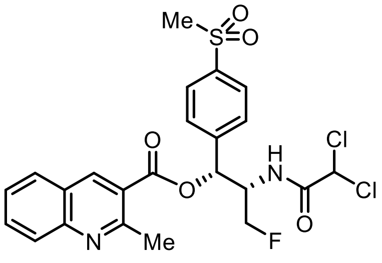

S28

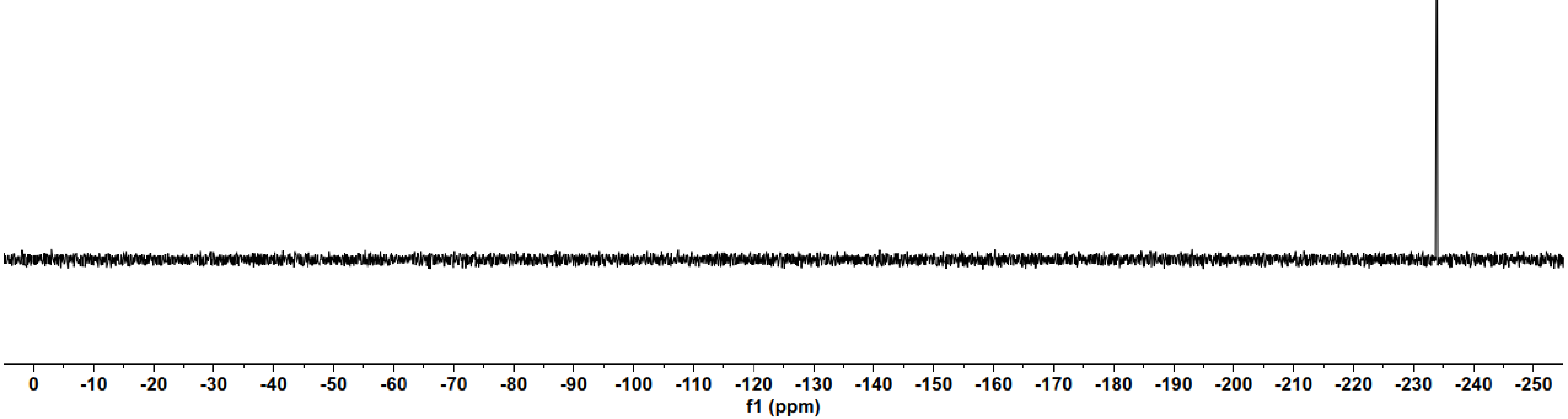

REFERENCES

1. Fulmer, G. R.; Miller, A. J.; Sherden, N. H.; Gottlieb, H. E.; Nudelman, A.; Stoltz, B. M.; Bercaw, J. E.; Goldberg, K. I. NMR chemical shifts of trace Impurities: Common laboratory solvents, oganics, and gases in deuterated solvents relevant to the organometallic chemist. *Organometallics* **2010**, *29*, 2176–2179.

2. Ma, J.; Chen, S.; Bellotti, P.; Wagener, T.; Daniliuc, C.; Houk, K. N.; Glorius, F. Facile access to fused 2D/3D rings via intermolecular cascade dearomative [2+2] cycloaddition/rearrangement reactions of quinolines with alkenes. *Nat. Catal.* **2022**, *5*, 405–413.

3. Boursalian, G. B.; Ngai, M. Y.; Hojczyk, K. N.; Ritter, T. Pd-catalyzed aryl C–H imidation with arene as the limiting reagent. *J. Am. Chem. Soc.* **2013**, *135*, 13278-13281.

4. Boursalian, G. B.; Ham, W. S.; Mazzotti, A. R.; Ritter, T. Charge-transfer-directed radical substitution enables para-selective C–H functionalization. *Nat. Chem.* **2016**, *8*, 810–815.

5. Zhang, F. L.; Li, B.; Houk, K. N.; Wang, Y. F. Application of the spin-center shift in organic synthesis. *JACS Au*, **2022**, *2*, 1032–1042.

6. Neese, F. The ORCA program system. *WIREs Comput. Mol. Sci.* **2012**, *2*, 73–78.

7. Becke, A. D. Density‐functional thermochemistry. III. The role of exact exchange. *J. Chem. Phys*. **1993**, *98*, 5648–5652.

8. Lee, C.; Yang, W.; Parr, R. G. Development of the Colle-Salvetti correlation-energy formula into a functional of the electron density. *Phys. Rev. B* **1988**, *37*, 785–789.

9. Grimme, S.; Antony, J.; Ehrlich, S.; Krieg, H. A consistent and accurate ab initio parametrization of density functional dispersion correction (DFT-D) for the 94 elements H–Pu. *J. Chem. Phys.* **2010**, *132*, 154104.

10. Grimme, S.; Ehrlich, S.; Goerigk, L. Effect of the damping function in dispersion corrected density functional theory. *J. Comput. Chem.* **2011**, 32, 1456–1465.

11. Neese, F.; Wennmohs, F.; Hansen, A.; Becker, U. Efficient, approximate and parallel Hartree–Fock and hybrid DFT calculations. A ‘chain-of-spheres’ algorithm for the Hartree–Fock exchange. *Chem. Phy.* **2009**, *356*, 98–109.

12. Weigend, F. Accurate Coulomb-fitting basis sets for H to Rn. *Phys. Chem. Chem. Phys.* **2006**, *8*, 1057–1065.

13. Weigend, F.; Ahlrichs, R. Balanced basis sets of split valence, triple zeta valence and quadruple zeta valence quality for H to Rn: Design and assessment of accuracy, *Phys. Chem. Chem. Phys.* **2005**, *7*, 3297–3305.

14. Neese, F. The SHARK integral generation and digestion system. *J. Comput. Chem.* doi: 10.1002/jcc.26942 (2022).

15. Barone, V.; Cossi, M. Quantum calculation of molecular energies and energy gradients in solution by a conductor solvent model, *J. Phys. Chem. A* **1998**, *102*, 1995–2001.

16. Glendening, E. D.; Badenhoop, J. K.; Reed, A. E.; Carpenter, J. E., Bohmann, J. A.; Morales, C. M.; Karafiloglou, P.; Landis, C. R.; Weinhold, F. NBO 7.0. Theoretical Chemistry Institute, University of Wisconsin, Madison, WI, 2018; http://nbo7.chem.wisc.edu.

17. Hanwell, M. D.; Curtis, D. E.; Lonie, D. C.; Vandermeersch, T.; Zurek, E.; Hutchison, G. R. Avogadro: an advanced semantic chemical editor, visualization, and analysis platform. *J. Cheminformatics* **2012**, *4*, 17.

18. k. Zhurko, G. A. Chemcraft-graphical program for visualization of quantum chemistry computations. https://chemcraftprog.com.

19. Proctor, R. S.; Phipps, R. J. Recent advances in Minisci-type reactions. *Angew. Chem. Int. Ed.* **2019**, *58*, 13666-13699.

20. Parsaee, F.; Senarathna, M. C.; Kannangara, P. B.; Alexander, S. N.; Arche, P. D. E.; Welin, E. R. Radical philicity and its role in selective organic transformations. *Nat. Rev. Chem.* **2021**, *5*, 486-499.

21. Marcus, R. A. On the theory of oxidation ‐ reduction reactions involving electron transfer. I. *J. Chem. Phys.* **1956**, *24*, 966−978.

22. Pause, L.; Robert, M.; Saveant, J. M. Reductive cleavage of carbon tetrachloride in a polar solvent. An example of a dissociative electron transfer with significant attractive interaction between the caged product fragments. *J. Am. Chem. Soc.* **2000**, *122*, 9829−9835.

23. Saveant, J. M. A simple model for the kinetics of dissociative electron transfer in polar solvents. Application to the homogeneous and heterogeneous reduction of alkyl halides. *J. Am. Chem. Soc.* **1987**, *109*, 6788-6795.

24. Saveant, J. M. Dissociative electron transfer. New tests of the theory in the electrochemical and homogeneous reduction of alkyl halides. *J. Am. Chem. Soc.* **1992**, *114*, 10595−10602.

25. Hammes-Schiffer, S. Proton-coupled electron transfer: Moving together and charging forward. *J. Am. Chem. Soc.*, **2015**, *137*, 8860-8871.

26. Tyburski, R.; Liu, T.; Glover, S. D.; Hammarström, L. Proton-coupled electron transfer guidelines, fair and square. *J. Am. Chem. Soc.*, **2021**, *143*, 560-576.

27. Warren, J. J.; Tronic, T. A.; Mayer, J. M. Thermochemistry of proton-coupled electron transfer reagents and its implications. *Chem. Rev.* **2010**, *110*, 6961−7001.

28. Fier, P. S.; Hartwig, J. F. Selective C-H fluorination of pyridines and diazines inspired by a classic amination reaction. *Science* **2013**, *342*, 956–960.

29. Dedeian, K.; Shi, J.; Shepherd, N.; Forsythe, E.; Morton, D. C. Photophysical and electrochemical properties of heteroleptic tris-cyclometalated iridium (III) complexes. *Inorg. Chem.* **2005**, *44*, 4445-4447.

30. Kim, J.; Sun, X.; van der Worp, B. A.; Ritter, T. Anti-Markovnikov hydrochlorination and hydronitrooxylation of α-olefins. *Nat. Catal.* **2023**, *6*, 196-203.

31. Yang, Q.; Wang, Y. H.; Qiao, Y.; Gau, M.; Carroll, P. J.; Walsh, P. J.; Schelter, E. J. Photocatalytic C–H activation and the subtle role of chlorine radical complexation in reactivity. *Science* **2021**, *372*, 847-852.

32. Kuhn, H. J.; Braslavsky, S. E.; Schmidt, R. Chemical actinometry (IUPAC Technical Report). *Pure Appl. Chem.* **2004**, *76*, 2105–2146.

33. Montalti, M.; Credi, A.; Prodi, L.; Gandolfi, M. T. *Chemical Actinometry. Handbook of Photochemistry*., 3rd Ed. 601–616; Taylor & Francis Group, Boca Raton, 2016.

34. Cismesia, M. A.; Yoon, T. P. Characterizing chain processes in visible light photoredox catalysis. *Chem. Sci.* **2015**, *6*, 5426–5434.

35. Hatchard, C. G.; Parker, C. A. A new sensitive chemical actinometer. II. Potassium ferrioxalate as a standard chemical actinometer. *Proc. Roy. Soc. (London)* **1956***, A235*, 518–536.

36. Bard, A. J. & Faulkner, L. R. Electrochemical Methods: Fundamentals and Applications, John Wiley & Sons, New York, NY, 2001.

37. Roth, H. G., Romero, N. A. & D. A. Nicewicz*,* Experimental and calculated electrochemical potentials of common organic molecules for applications to single-electron redox chemistry. *Synlett* **2016**, *27*, 714.
